# Enantiodivergent [4+2] Cycloaddition of Dienolates by Polyfunctional Lewis Acid/Zwitterion Catalysis

**DOI:** 10.1002/anie.202009093

**Published:** 2020-08-28

**Authors:** Vukoslava Miskov‐Pajic, Felix Willig, Daniel M. Wanner, Wolfgang Frey, René Peters

**Affiliations:** ^1^ Universität Stuttgart Institut für Organische Chemie Pfaffenwaldring 55 70569 Stuttgart Germany

**Keywords:** asymmetric catalysis, Diels–Alder cycloaddition, lactams, lactones, polyfunctional catalysts

## Abstract

Diels–Alder reactions have become established as one of the most effective ways to prepare stereochemically complex six‐membered rings. Different catalysis concepts have been reported, including dienophile activation by Lewis acids or H‐bond donors and diene activation by bases. Herein we report a new concept, in which an acidic prodiene is acidified by a Lewis acid to facilitate deprotonation by an imidazolium–aryloxide entity within a polyfunctional catalyst. A metal dienolate is thus formed, while an imidazolium–ArOH moiety probably forms hydrogen bonds with the dienophile. The catalyst type, readily prepared in few steps in high overall yield, was applied to 3‐hydroxy‐2‐pyrone and 3‐hydroxy‐2‐pyridone as well as cyclopentenone prodienes. Maleimide, maleic anhydride, and nitroolefin dienophiles were employed. Kinetic, spectroscopic, and control experiments support a cooperative mode of action. High enantioselectivity was observed even with unprecedented TONs of up to 3680.

## Graphical abstract

In an efficient approach to catalytic asymmetric [4+2] cycloaddition, acidic prodienes were deprotonated by a betaine species to form a copper dienolate, and the dienophile was activated by hydrogen‐bond formation (see scheme). The polyfunctional catalyst promoted the transformation with high enantioselectivity as well as high turnover numbers.

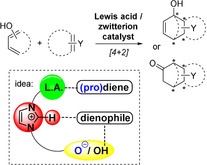

Asymmetric Diels–Alder reactions (DAs) provide a very important tool to enantioselectively construct stereochemically complex six‐membered carbocycles.^[1]^ Attractive features of this reaction type include stereospecificity regarding diene and dienophile components, wide applicability of different substrate types and the possibility to stereoselectively construct a number of stereogenic centers within the formed cyclohexene ring, thus creating structural complexity.^[1]^ Various concepts for catalytic asymmetric DAs have been reported^[1]^ including the use of chiral Lewis acids (LA),^[2]^ primary or secondary amines/ammonium salts,^[3]^ Brønsted acids,^[4]^ hydrogen bond donors,^[5]^ and organic Brønsted bases.^[4]^

With the traditional LA catalysts the dienophile's LUMO energy is decreased (Scheme 1).^[2]^ More rarely, metal centers have also served as tethers to allow for quasi‐intramolecular reaction pathways.^[6]^ In contrast, bases were described to increase the HOMO energy of acidic (pro)dienes.[^1^, ^3^, ^4^]

**Scheme 1:**
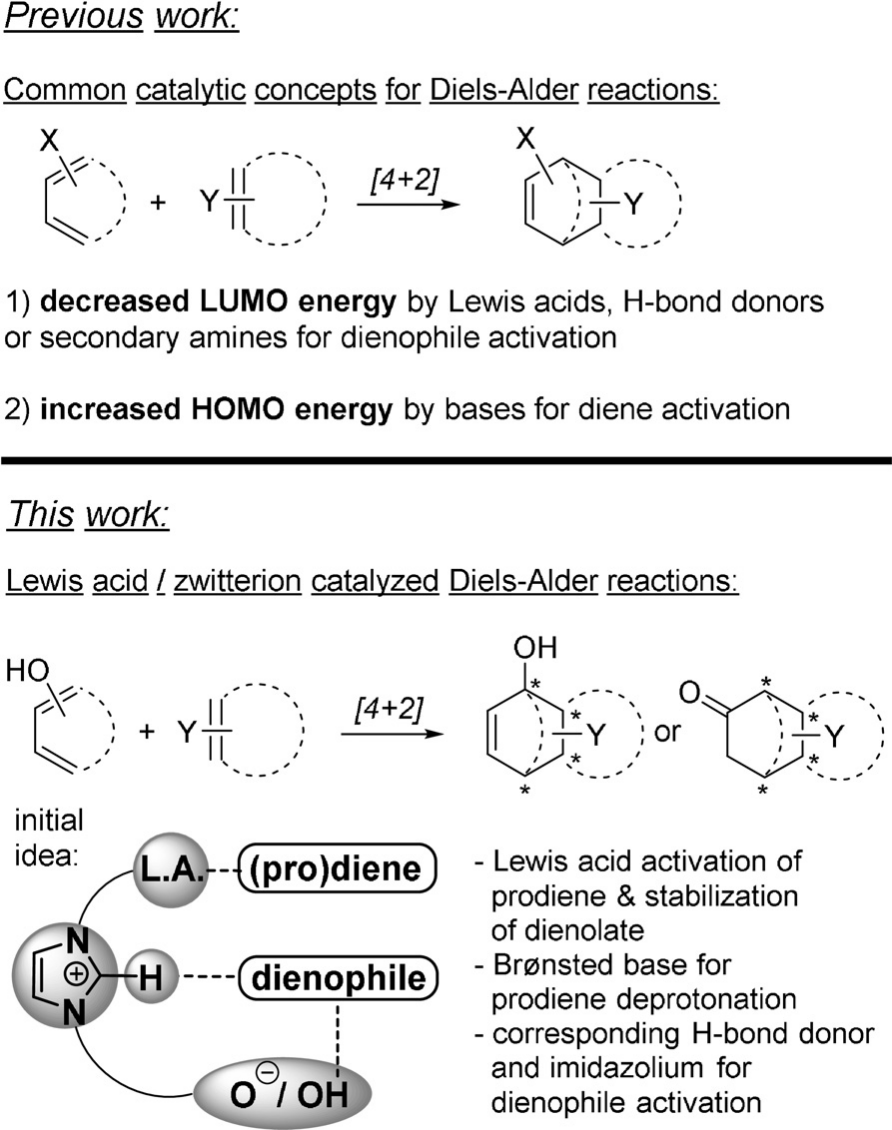
Comparison of previous Diels–Alder approaches with that presented.

In this article, we report the use of polyfunctional catalysts, in which the cooperation of a LA^[7]^ and an aprotic imidazolium aryloxide moiety is utilized to generate the reactive diene.^[8]^ The prodiene is acidified by coordination to the LA and then deprotonated by the aryloxide moiety.^[9]^ The LA should stabilize the generated dienolate. Besides, hydrogen bond activation is crucial in this catalyst system, as control experiments show, most likely for activation of the dienophile.[^10^, ^11^]

For our initial investigations, 3‐hydroxy‐2‐pyrones **1** were studied as diene component to form densely functionalized bicyclic lactones. Similar lactones have been described as valuable building blocks for the synthesis of a number of structurally complex, biologically active natural products such as taxol,^[6b]^ and others.[^12^, ^13^] Pyrones are usually quite reluctant to undergo DAs,^[14]^ unless the electron density is increased by formation of a dienolate.^[15]^ Consequently, chiral Brønsted bases—in particular cinchona alkaloid derivatives—have been reported for highly enantioselective activation.^[16]^ Maleimides **2** were initially investigated as dienophiles, because succinimides not only represent an important structural motif in bioactive compounds,^[17]^ but also because the previously reported methods using a combination of **1** and **2** suffered from moderate enantioselectivity and very limited scope.[^16a^, ^16b^, ^16c^]

Cu^II^/imidazolium/naphthoxide complex **C1 a** (5 mol %)^[8]^ was found to catalyze the reaction of **1 a** and **2A** at room temperature with complete *endo* selectivity, yet low enantioselectivity (entry 1). At −40 °C, the *ee* could be significantly increased to 88 %, while an excellent yield was maintained (entry 2). Lower reaction temperatures gave a decreased enantioselectivity (not shown). Formally replacing the *N*‐Me substituent by the synthetically useful *N*‐benzyl protecting group caused a slightly improved enantioselectivity employing the same conditions (entry 3). Product **3 aB** has been described as valuable precursor to antagonists of neurotransmitter SP, but was previously only accessible with moderate enantioselectivity.^[18]^ A change in configuration of the iminosulfonamide moiety from (1*S*,2*S*) to (1*R*,2*R*) resulted in the formation of the opposite product enantiomer (*ee*=91 %, entry 4). This shows that the configurational outcome is dominated by the iminosulfonamide moiety, rather than the axially chiral part.

It was found that the choice of the sulfonamide unit heavily influences the enantioselectivity. Most residues R^1^ (Me, *p*‐tolyl, C_6_F_5_, 2‐ and 4‐nitrophenyl, 2‐naphthyl) resulted in poor to moderate enantioselectivity (for details see the SI). Nevertheless, with R^1^=1‐naphthyl in **C1 b**, which is accessible in an overall yield of 84 % starting from (*R*)‐BINAM (see SI), the enantiomeric excess increased to 96 % (entry 5). Adding **1 a** by syringe pump further improved the *ee* to 98 % (entry 9).

To our surprise, with **C1 b** the optical antipode of **3 aB** was formed, although the (1*S*,2*S*) iminosulfonamide configuration was used (compare entries 3 & 5). In contrast, the (1*R*,2*R*) configuration for **C1 b** resulted in no switch of the preferred enantiomer, but a significantly decreased *ee* value (entry 6). We suggest that the coordination of **1** at Cu might result in different coordination geometries depending on the sulfonamide residues. Lower loadings still resulted in good results (entries 7,8).

Different pyrones **1** were examined as prodienes under conditions of Table 1, entry 9, using catalysts **C1 a** and **C1 b** (Table 2). Next to **1 a**, also the 4‐Me and 4‐halo substituted substrates were effective and only *endo* products were found (entries 4–6).

**Table 1:** Development and optimization of the title reaction. 
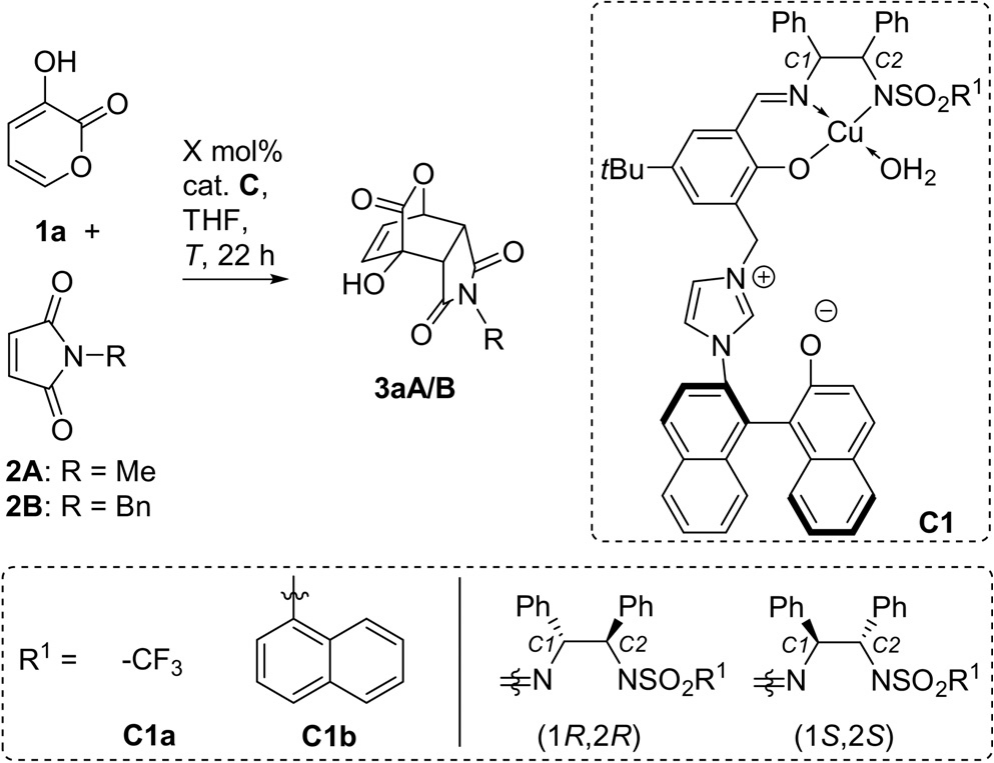

Entry	**C1** (config.)	*X* [mol %]	**2**	*T* [°C]	Yield^[a]^ [%]	*dr* ^[b]^	*ee* ^[c]^ [%]
1	**C1 a** (1*S*,2*S*)	5	**2A**	25	99	>98:2	30
2	**C1 a** (1*S*,2*S*)	5	**2A**	−40	97	>98:2	88
3	**C1 a** (1*S*,2*S*)	5	**2B**	−40	97	>98:2	90
4	**C1 a** (1*R*,2*R*)	5	**2B**	−40	95	>98:2	−91
5	**C1 b** (1*S*,2*S*)	5	**2B**	−40	98	>98:2	−96
6	**C1 b** (1*R*,2*R*)	5	**2B**	−40	96	>98:2	−40
7^[d,e]^	**C1 b** (1*S*,2*S*)	2.5	**2B**	−40	98	>98:2	−96
8^[d,e]^	**C1 b** (1*S*,2*S*)	1	**2B**	−40	95	>98:2	−90
9^[e]^	**C1 b** (1*S*,2*S*)	5	**2B**	−40	98	>98:2	−98

[a] Yield of the isolated product. [b] *Endo*/*exo* ratios were determined by ^1^H NMR spectroscopy of the crude product. [c] The *ee* value of the *endo* isomer was determined by ^1^H NMR spectroscopy using saturated CDCl_3_ solutions of (*R*)‐(+)‐binaphthol.^[16a]^ A minus sign indicates that the antipode of the enantiomer depicted was generated in excess. [d] Reaction time: 18 h. [e] Compound **1 a** was added with a syringe pump.

**Table 2:** Investigation of various 3‐hydroxy‐2‐pyrones and 3‐hydroxy‐2‐pyridones. 
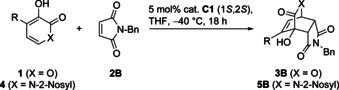

Entry	**C1**	R	**1** or **4**	**3B** or **5B**	Yield [%]^[a]^	*dr* ^[b]^	*ee* [%]^[c]^
1	**C1 a**	H	**1 a**	**3 aB**	97	>98:2	90
2	**C1 b**	H	**1 a**	*ent*‐**3 aB**	98	>98:2	−98
3^[d]^	**C1 b**	H	**1 a**	*ent*‐**3 aB**	96	>98:2	−97
4	**C1 a**	Me	**1 b**	**3 bB**	93	>98:2	93
5	**C1 a**	Cl	**1 c**	**3 cB**	92	>98:2	95
6	**C1 a**	Br	**1 d**	**3 dB**	94	>98:2	93
7^[e]^	**C1 b**	H	**4 a**	*ent*‐**5 aB**	97	>98:2	−94
8^[e]^	**C1 b**	allyl	**4 b**	*ent*‐**5 bB**	98	>98:2	−96
9^[e]^	**C1 a**	allyl	**4 b**	**5 bB**	95	>98:2	95
10^[e]^	**C1 b**	Cl	**4 c**	*ent*‐**5 cB**	97	>98:2	−79
11^[e,f]^	**C1 b**	H	**4 a**	*ent*‐**5 aC**	94	>98:2	−94
12^[e,g]^	**C1 b**	H	**4 a**	*ent*‐**5 aF**	94	>98:2	−95

[a] Yield of the isolated product. [b] *Endo*/*exo* ratios were determined by ^1^H NMR spectroscopy of the crude product. [c] The *ee* value was determined by ^1^H NMR spectroscopy using saturated CDCl_3_ solutions of (*R*)‐(+)‐binaphthol.^[16a]^ A minus sign indicates that the antipode of the enantiomer depicted was generated in excess. [d] The reaction was performed on a 5 mmol scale. [e] The reaction was performed at 25 °C. [f] 4‐O_2_N‐C_6_H_4_ was used for Bn in **2**. [g] H was used for Bn in **2**.

To showcase the practicality of this method, it was performed on a 5 mmol scale and provided excellent results (entry 3). In addition, catalyst recovery of **C1 b** was successful by silica gel filtration.^[8]^ Employing the recovered catalyst provided *ent*‐**3 aB** in diastereopure form in 92 % yield and with 98 % *ee* for the second run and 92 % yield and with 96 % *ee* for the third run.

Besides pyrones **1**, we also examined pyridones **4** (entries 7–12) which are valuable to prepare bioactive compounds like the antiviral drug Oseltamivir.^[19]^ For catalytic asymmetric DAs of pyridones with **2**, there is a single method available providing high enantioselectivity, which is currently limited though to *N*‐mesitylsulfonyl substituted substrates.^[20]^ In our study 2‐nosyl (Ns) protected substrates **4** worked fine as well (entries 7–12). Again, only *endo* isomers were found and different enantiomers were available with catalysts **C1 a** and **C1 b**.^[21]^ The 2‐Ns protecting group could be readily removed as shown for *ent*‐**5 aB** by PhSH/DBU at 25 °C in THF (yield: 94 %, *ee* 94 %).

Different maleimides **2** were also well tolerated (Table 3). Next to unbranched and branched *N*‐alkyl (entries 1,2), a Boc group (entry 3) was accepted which was removable from **3 aE** in 92 % yield (*ee*=92 %) using TFA at 25 °C. Moreover, product formation was also achieved with unprotected maleimide (entry 4 and Table 2, entry 12). In addition, aromatic substituents with various electronic properties were tolerated (entry 5 and SI). The reaction could also be extended to maleic anhydride **6** providing product **7**.^[16j]^ All products were formed in high yields in diastereopure form with high enantioselectivity.^[21]^

**Table 3:** Investigation of maleimide dienophiles **2** and maleic anhydride **6**. 
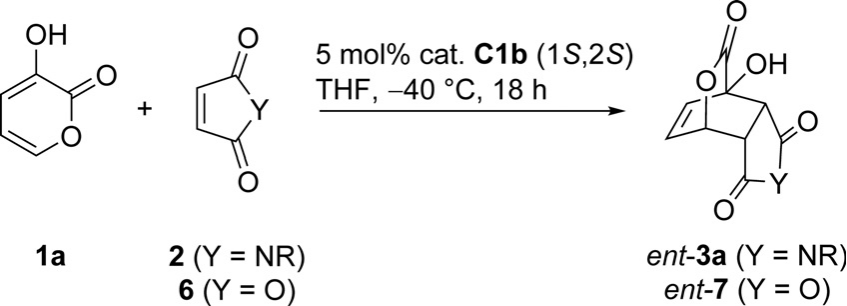

Entry	**2** or **6**	Y	(*ent*‐)**3 a** or **7**	Yield [%]^[a]^	*dr* ^[b]^	*ee* [%]^[c]^
1	**2A**	Me−N	*ent*‐**3 aA**	93	>98:2	93
2	**2D**	*c*Hex−N	*ent*‐**3 aD**	92	>98:2	96
3^[d]^	**2E**	Boc−N	**3 aE**	90	>98:2	−92
4^[e]^	**2F**	H−N	*ent‐* **3 aF**	89	>98:2	90
5	**2G**	Ph−N	*ent*‐**3 aG**	94	>98:2	91
6^[d,f]^	**6**	O	**7**	92	>98:2	−84

[a] Yield of the isolated product. [b] *Endo*/*exo* ratios were determined by ^1^H NMR spectroscopy of the crude product. [c] The *ee* value was determined by ^1^H NMR spectroscopy using saturated CDCl_3_ solutions of (*R*)‐(+)‐binaphthol.^[16a]^ A minus sign indicates that the antipode of the enantiomer depicted was generated in excess. [d] **C1 a** (1*S*,2*S*) was used as the catalyst. [e] The reaction was performed at −20 °C. [f] The reaction was performed at 0 °C.

Other types of C−H acidic prodienes can be applied as well as demonstrated for enone **8** (Scheme 2). With **2B** catalyst **C1 a** produced bicycloheptane **9** exclusively as *exo* diastereomer featuring four contiguous stereocenters (98 % *ee*).^[22]^ Surprisingly, this catalytic asymmetric DA is unknown, although compounds closely related to **9** have been reported as modulators of nuclear hormone receptor function.^[23]^ The unexpected *exo* selectivity might possibly point to a stepwise process in that case.

**Scheme 2:**
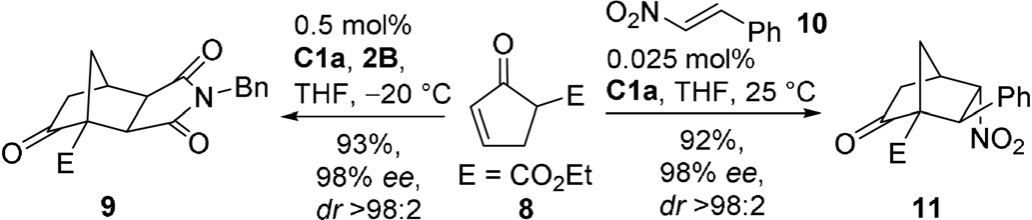
Extension to different substrate types (**C1 a** with 1*S*,2*S* configuration).

In addition, with nitroolefin **10** the cycloaddition proceeded very efficiently (TON 3680, 92 % yield) with just 0.025 mol % **C1 a**. The previously not available *endo* product was obtained with an *ee* of 98 %. Prior to this study, only the *exo* isomer of **11** was accessible with moderate *dr* by chiral amine catalysis with TONs <20.[^24^, ^25^]

To learn more about the role of the aprotic betaine, experiments with several control catalysts were conducted (Table 4). Cs_2_CO_3_ (2.5 mol %) in the absence of a Cu complex formed racemic product **3 aB** in 95 % yield as an *endo*/*exo* mixture (84:16).

**Table 4:** Experiments with control catalyst systems. 
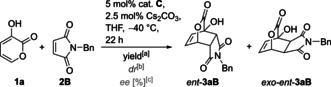

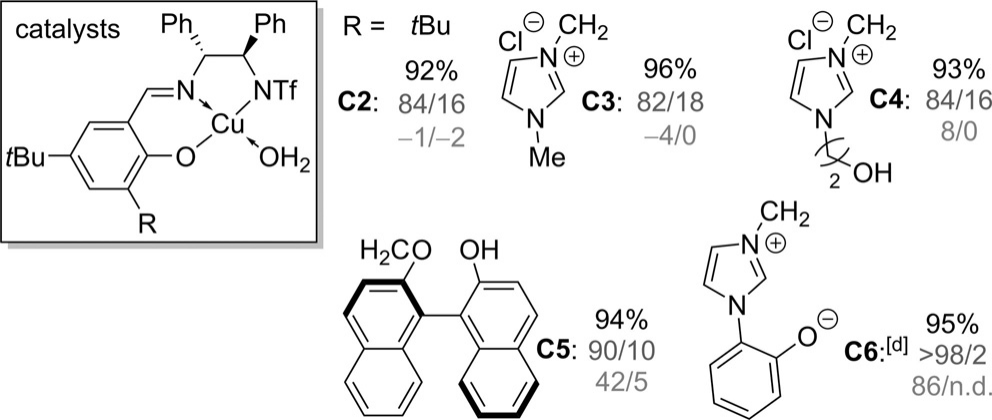

[a] Yield of the isolated product. [b] *Endo*/*exo* ratios were determined by ^1^H NMR spectroscopy of the crude product. [c] The *ee* value was determined by ^1^H NMR spectroscopy using saturated CDCl_3_ solutions of (*R*)‐(+)‐binaphthol.^[16a]^ [d] Without Cs_2_CO_3_.

With control catalysts **C2**–**C4** (5 mol %) bearing either an alkyl moiety R, a simple imidazolium or one with an aliphatic alcohol moiety, the reaction outcome was almost identical as with only Cs_2_CO_3_. The experiments with **C2** and **C4** were also repeated in the absence of base providing yields of 5 % (*ee* n.d.) and 10 % (racemic), respectively. In contrast, using Cs_2_CO_3_ and **C5** equipped with a neutral axially chiral residue bearing an aromatic OH group, stereoselectivity was considerably higher. However, nearly complete diastereoselectivity was only attained with control catalyst **C6** containing an achiral zwitterionic imidazolium/aryloxide moiety.^[26]^ In agreement with the observation that the configuration of the axially chiral binaphthol unit has a minor effect on stereocontrol, enantioselectivity was high with **C6**. Nevertheless, these results demonstrate that the presence of the zwitterionic moiety (achiral or chiral) is necessary to attain high control.^[27]^

Next to the control experiments, kinetic and spectroscopic experiments were performed (for details see SI). Blackmond's reaction progress kinetic analysis (RPKA) was done using ^1^H NMR spectroscopy.^[28]^ By the “same excess” protocol, catalyst robustness and a possible product inhibition was assessed.^[27]^ These experiments show that no significant catalyst decomposition and product inhibition occur in the model reaction.

The empirical rate law was determined by the variable time normalization analysis (VTNA) method introduced by Burés.^[29]^ Again, the model reaction of **1 a** and **2B** was examined at −20 °C in [D_8_]THF using catalyst **C1 b** (1*S*,2*S*). Four reactions with different initial concentrations of **C1 b**, **1 a** and **2B** were used monitoring product *ent*‐**3 aB**. The best fit for the normalization of the time scale axis was achieved for the following rate law [Eq. 1]:

(1) $$r=k\;\left[C1\;b\right]{}^{1.95}\;\left[1\;a\right]{}^{0.07}\;\left[2B\right]{}^{1.00}$$

The reaction rate thus follows a second order kinetic dependence for **C1 b**, a first order for **2B**, and zero order for **1 a**. The latter might be explained by substrate saturation for **1 a**. The second order dependence for **C1 b** indicates that two catalyst molecules are involved in the turnover‐limiting step.^[30]^ There is also a significant positive nonlinear effect for the model reaction of **1 a** and **2B** catalyzed by **C1 b** (5 mol %) in THF at −40 °C. We suggest that a dimeric catalyst might be the active species, because a dimeric structure was also found for **C6** by X‐ray analysis^[31]^ (details are in the SI).

UV/Vis titrations were performed in which **C1 b** was treated with **1 a**. Having added 1.0 equivalent of **1 a**, the resulting spectrum is very similar to the one of the precatalyst, in which a neutral naphthol unit is present. With larger amounts of up to 10 equivalents of **1 a**, the spectra did not significantly change anymore. This suggests that **1 a** is deprotonated by the zwitterionic moiety. In combination with the zero order kinetic dependence for **1 a**, we suggest that **1 a** binds to the Cu center where it gets deprotonated resulting in substrate saturation. A copper dienolate could indeed be found by ESI HRMS (disodium salt: *m*/*z* 1128.2563, calculated: 1128.2564). The interpretation was validated by ^1^H NMR titrations, which were done in [D_8_]THF at −20 °C. Because the catalyst is paramagnetic, no well resolved signals were detected. By addition of 1 equivalent of **1 a**, its signals almost disappeared caused by the paramagnetism. However, with an excess of **1 a** the signals were appearing again, thus suggesting that the catalyst is already saturated with 1 equivalent.

In conclusion, we have reported an efficient way to catalyze [4+2] cycloadditions with acidic prodienes. They are activated by coordination to a LA and deprotonation by a zwitterionic moiety forming a metal dienolate. The acidic OH group generated during this proton transfer and the imidazolium unit are crucial for excellent diastereo‐ and enantioselectivity. In combination with the first order kinetic dependence for maleimide **2B**, it is likely that the cycloaddition step is usually turnover‐limiting. The reaction was found to be applicable to different substrate types and TONs of up to 3680 were achieved. Moreover, the catalyst can be prepared in very high overall yield (**C1 b**: 84%), is stable during catalysis and can be readily recycled by a simple filtration protocol.

## Conflict of interest

The authors declare no conflict of interest.

## Supporting information

As a service to our authors and readers, this journal provides supporting information supplied by the authors. Such materials are peer reviewed and may be re‐organized for online delivery, but are not copy‐edited or typeset. Technical support issues arising from supporting information (other than missing files) should be addressed to the authors.

Supplementary
