# Supplementary material for: Enantiodivergent [4+2] Cycloaddition of Dienolates by Polyfunctional Lewis Acid/Zwitterion Catalysis
Source: Angew Chem Int Ed Engl. 2020 Aug 28;59(45):19873–7. doi: 10.1002/anie.202009093 (PMC7693193; doi:10.1002/anie.202009093)
Supplement: Supplementary file 1 — Supplementary [file ANIE-59-19873-s001.pdf]

Angewandte Chemie
Eine Zeitschrift der Gesellschaft Deutscher Chemiker

Supporting Information

Enantiodivergent [4+2] Cycloaddition of Dienolates by Polyfunctional Lewis Acid/Zwitterion Catalysis

*Vukoslava Miskov-Pajic, Felix Willig, Daniel M. Wanner, Wolfgang Frey, and René Peters**

anie_202009093_sm_miscellaneous_information.pdf

Table of Contents

General Remarks 6

General Procedures (**GP**) 7

General Procedure for the Imine-Synthesis (**GP1**) 7

General Procedure for the Metal Complexation (**GP2**) 7

General Procedure for the Activation of the Complexes (**GP3**) 8

General Procedure for Catalytic Diels–Alder Reactions of 3-Hydroxypyrones (**GP4**) 8

General Procedure for Catalytic Diels–Alder Reactions of 3-Hydroxypyrones in Control Experiments (**GP5**) 9

General Procedure for the Catalytic Diels–Alder Reactions of 3-Hydroxypyridones (**GP6**) 9

General Procedure for the Cycloaddition Reaction with Enone (**8**) (**GP7**) 10

General Procedure for the Determination of *ee* values with (*R*)-BINOL (**GP8**) 10

Substrate Synthesis 11

4-Chloro-3-hydroxy-2-pyrone (**1c**) 11

N-2-Nitrobenzenesulfonyl-4-allyl-3-hydroxy-2-pyridone (**4b**) 11

N-2-Nitrobenzenesulfonyl-4-chloro-3-hydroxy-2-pyridone (**4c**) 12

Ligand Synthesis 12

(*R*)-2-(Imidazol-1-yl)-2′-hydroxy-1,1′-binaphthyl (**K1**) 12

(*R*)-3-(5-(*tert*-Butyl)-3-formyl-2-hydroxybenzyl)-1-(2'-hydroxy-[1,1'-binaphthalen]-2-yl)-1H-imidazol-3-ium chloride (**K2**) 13

3-(5-(*tert*-Butyl)-2-hydroxy-3-((*E*)-(((1*S*,2*S*)-2-(naphthalene-1-sulfonamido)-1,2-diphenylethyl)imino)methyl)benzyl)-1-((*R*)-2'-hydroxy-[1,1'-binaphthalen]-2-yl)-1*H*-imidazol-3-ium-chloride (**L1b-(*S*,*S*)**) 13

3-(5-(*tert*-Butyl)-2-hydroxy-3-((*E*)-(((1*R*,2*R*)-2-(naphthalene-1-sulfonamido)-1,2-diphenylethyl)imino)methyl)benzyl)-1-((*R*)-2'-hydroxy-[1,1'-binaphthalen]-2-yl)-1*H*-imidazol-3-ium-chloride (**L1b-(*R*,*R*)**) 14

3-(5-(*tert*-Butyl)-2-hydroxy-3-((*E*)-(((1*S*,2*S*)-2-(anthrachene-9-sulfonamido)-1,2-diphenylethyl)imino)methyl)benzyl)-1-((*R*)-2'-hydroxy-[1,1'-binaphthalen]-2-yl)-1*H*-imidazol-3-ium-chloride (**L1c-(*S*,*S*)**) 15

3-(5-(*tert*-Butyl)-2-hydroxy-3-((*E*)-(((1*S*,2*S*)-2-(naphthalene-2-sulfonamido)-1,2-diphenylethyl)imino)methyl)benzyl)-1-((*R*)-2'-hydroxy-[1,1'-binaphthalen]-2-yl)-1*H*-imidazol-3-ium-chloride (**L1d-(*S*,*S*)**) 16

3-(5-(*tert*-Butyl)-2-hydroxy-3-((*E*)-(((1*R*,2*R*)-2-(naphthalene-2-sulfonamido)-1,2-diphenylethyl)imino)methyl)benzyl)-1-((*R*)-2'-hydroxy-[1,1'-binaphthalen]-2-yl)-1*H*-imidazol-3-ium-chloride (**L1d-(*R*,*R*)**) 16

3-(5-(*tert*-Butyl)-2-hydroxy-3-((*E*)-(((1*S*,2*S*)-2-(2-nitrobenzenesulfonylamido)-1,2-diphenylethyl)imino)methyl)benzyl)-1-((*R*)-2'-hydroxy-[1,1'-binaphthalen]-2-yl)-1*H*-imidazol-3-ium-chloride (**L1e-(*S*,*S*)**) 17

3-(5-(*tert*-Butyl)-2-hydroxy-3-((*E*)-(((1*R*,2*R*)-2-(2-nitrobenzenesulfonylamido)-1,2-diphenylethyl)imino)methyl)benzyl)-1-((*R*)-2'-hydroxy-[1,1'-binaphthalen]-2-yl)-1*H*-imidazol-3-ium-chloride (**L1e-(*R*,*R*)**) 18

3-(5-(*tert*-Butyl)-2-hydroxy-3-((*E*)-(((1*S*,2*S*)-2-(4-nitrobenzenesulfonylamido)-1,2-diphenylethyl)imino)methyl)benzyl)-1-((*R*)-2'-hydroxy-[1,1'-binaphthalen]-2-yl)-1*H*-imidazol-3-ium-chloride (**L1f-(*S*,*S*)**) 18

3-(5-(*tert*-Butyl)-2-hydroxy-3-((*E*)-(((1*R*,2*R*)-2-(2-nitrobenzenesulfonylamido)-1,2-diphenylethyl)imino)methyl)benzyl)-1-((*R*)-2'-hydroxy-[1,1'-binaphthalen]-2-yl)-1*H*-imidazol-3-ium-chloride (**L1f-(*R*,*R*)**) 19

3-(5-(*tert*-Butyl)-3-((*E*)-(((1*S*,2*S*)-1,2-diphenyl-2-(methylsulfonamido)ethyl)imino)-methyl)-2-hydroxybenzyl)-1-((*R*)-2'-hydroxy-[1,1'-binaphthalen]-2-yl)-1H-imidazol-3-iumchlorid (**L1g-(*S*,*S*)**) . 20

3-(5-(*tert*-Butyl)-3-((*E*)-(((1*R*,2*R*)-1,2-diphenyl-2-(methylsulfonamido)ethyl)imino)-methyl)-2-hydroxybenzyl)-1-((*R*)-2'-hydroxy-[1,1'-binaphthalen]-2-yl)-1H-imidazol-3-iumchlorid (**L1g-(*R*,*R*)**) 20

3-(5-(*tert*-Butyl)-3-((*E*)-(((1*S*,2*S*)-1,2-diphenyl-2-(*p*-tolylsulfonamido)ethyl)imino)-methyl)-2-hydroxybenzyl)-1-((*R*)-2'-hydroxy-[1,1'-binaphthalen]-2-yl)-1*H*-imidazol-3-iumchlorid (**L1h-(*S*,*S*)**). 21

3-(5-(*tert*-Butyl)-3-((*E*)-(((1*R*,2*R*)-1,2-diphenyl-2-(*p*-tolylsulfonamido)ethyl)imino)-methyl)-2-hydroxybenzyl)-1-((*R*)-2'-hydroxy-[1,1'-binaphthalen]-2-yl)-1*H*-imidazol-3-iumchlorid (**L1h-(*R*,*R*)**) 22

3-(5-(*tert*-Butyl)-3-((*E*)-(((1*S*,2*S*)-1,2-diphenyl-2-(pentafluorobenzylsulfonamido)ethyl)-imino)-methyl)-2-hydroxybenzyl)-1-((*R*)-2'-hydroxy-[1,1'-binaphthalen]-2-yl)-1H-imidazol-3-iumchlorid (**L1i-(*S*,*S*)**) 22

3-(5-(*tert*-Butyl)-3-((*E*)-(((1*R*,2*R*)-1,2-diphenyl-2-(pentafluorobenzylsulfonamido)ethyl)-imino)-methyl)-2-hydroxybenzyl)-1-((*R*)-2'-hydroxy-[1,1'-binaphthalen]-2-yl)-1H-imidazol-3-iumchlorid (**L1i-(*R*,*R*)**) 23

Synthesis of the Complexes 24

3-(5-(*tert*-Butyl)-3-((*E*)-(((1*S*,2*S*)-1,2-diphenyl-2-((1-naphthalene)sulfonamido)ethyl)imino)methyl)-2-hydroxybenzyl)-1-(2'-hydroxy-[1,1'-binaphthalen]-2-yl)-1*H*-imidazol-3-ium copper (II)-chloride (**C1b-(*S*,*S*)**) 24

3-(5-(*tert*-Butyl)-3-((*E*)-(((1*R*,2*R*)-1,2-diphenyl-2-((1-naphthalene)sulfonamido)ethyl)imino)methyl)-2-hydroxybenzyl)-1-(2'-hydroxy-[1,1'-binaphthalen]-2-yl)-1*H*-imidazol-3-ium copper (II)-chloride (**C1b-(*R*,*R*)**) 24

3-(5-(*tert*-Butyl)-3-((*E*)-(((1*S*,2*S*)-1,2-diphenyl-2-((2-(anthrachene-9-sulfonamido)ethyl)imino)methyl)-2-hydroxybenzyl)-1-(2'-hydroxy-[1,1'-binaphthalen]-2-yl)-1*H*-imidazol-3-ium copper (II)-chloride (**C1c-(*S*,*S*)**) 25

3-(5-(*tert*-Butyl)-3-((*E*)-(((1*S*,2*S*)-1,2-diphenyl-2-((2-naphthalene)sulfonamido)ethyl)imino)methyl)-2-hydroxybenzyl)-1-(2'-hydroxy-[1,1'-binaphthalen]-2-yl)-1*H*-imidazol-3-ium copper (II)-chloride (**C1d-(*S*,*S*)**) 26

3-(5-(*tert*-Butyl)-3-((*E*)-(((1*R*,2*R*)-1,2-diphenyl-2-((2-naphthalene)sulfonamido)ethyl)imino)methyl)-2-hydroxybenzyl)-1-(2'-hydroxy-[1,1'-binaphthalen]-2-yl)-1*H*-imidazol-3-ium copper (II)-chloride (**C1d-(*R*,*R*)**) 27

3-(5-(*tert*-Butyl)-3-((*E*)-(((1*S*,2*S*)-1,2-diphenyl-2-((2-nitrophenyl)sulfonamido)ethyl)imino)methyl)-2-hydroxybenzyl)-1-(2'-hydroxy-[1,1'-binaphthalen]-2-yl)-1*H*-imidazol-3-ium copper (II)-chloride (**C1e-(*S*,*S*)**) 27

3-(5-(*tert*-Butyl)-3-((*E*)-(((1*R*,2*R*)-1,2-diphenyl-2-((2-nitrophenyl)sulfonamido)ethyl)imino)methyl)-2-hydroxybenzyl)-1-(2'-hydroxy-[1,1'-binaphthalen]-2-yl)-1*H*-imidazol-3-ium copper (II)-chloride (**C1e-(*R*,*R*)**) 28

3-(5-(*tert*-Butyl)-3-((*E*)-(((1*S*,2*S*)-1,2-diphenyl-2-((4-nitrophenyl)sulfonamido)ethyl)imino)methyl)-2-hydroxybenzyl)-1-(2'-hydroxy-[1,1'-binaphthalen]-2-yl)-1*H*-imidazol-3-ium copper (II)-chloride (**C1f-(*S*,*S*)**) 29

3-(5-(*tert*-Butyl)-3-((*E*)-(((1*R*,2*R*)-1,2-diphenyl-2-((4-nitrophenyl)sulfonamido)ethyl)imino)methyl)-2-hydroxybenzyl)-1-(2'-hydroxy-[1,1'-binaphthalen]-2-yl)-1*H*-imidazol-3-ium copper (II)-chloride (**C1f-(*R*,*R*)**) 30

3-(5-(*tert*-Butyl)-2-hydroxy-3-((*E*)-(((1*S*,2*S*)-2-(methylsulfonamido)-1,2-diphenylethyl)imino)methyl)benzyl)-1-((*R*)-2'-hydroxy-[1,1'-binaphthalen]-2-yl)-1H-imidazol-3-ium copper (II) chloride (**C1g-(*S*,*S*)**) 30

3-(5-(*tert*-Butyl)-3-((*E*)-(((1*R*,2*R*)-1,2-diphenyl-2(methylsulfonamido)ethyl)imino)methyl)-2-hydroxybenzyl)-1-(2'-hydroxy-[1,1' binaphthalen]-2-yl)-1*H*-imidazol-3-ium-copper(II)chloride (**C1g-(*R*,*R*)**) 31

3-(5-(*tert*-Butyl)-3-((*E*)-(((1*S*,2*S*)-1,2-diphenyl-2-((4-methylbenzene)sulfonamido)ethyl)imino)methyl)-2-hydroxybenzyl)-1-(2'-hydroxy-[1,1'-binaphthalen]-2-yl)-1*H*-imidazol-3-ium copper (II)-chloride (**C1h-(*S*,*S*)**) 32

3-(5-(*tert*-Butyl)-3-((*E*)-(((1*R*,2*R*)-1,2-diphenyl-2-((4-methylbenzene)sulfonamido)ethyl)imino)methyl)-2-hydroxybenzyl)-1-(2'-hydroxy-[1,1'-binaphthalen]-2-yl)-1*H*-imidazol-3-ium copper (II)-chloride (**C1h-(*R*,*R*)**) 33

3-(5-(*tert*-Butyl)-3-((*E*)-(((1*S*,2*S*)-1,2-diphenyl-2-((perfluorophenyl)sulfonamido)ethyl)imino)methyl)-2-hydroxybenzyl)-1-(2'-hydroxy-[1,1'-binaphthalen]-2-yl)-1*H*-imidazol-3-ium copper (II)-chloride (**C1i-(*S*,*S*)**) 33

3-(5-(*tert*-Butyl)-3-((*E*)-(((1*R*,2*R*)-1,2-diphenyl-2-((perfluorophenyl)sulfonamido)ethyl)imino)methyl)-2-hydroxybenzyl)-1-(2'-hydroxy-[1,1'-binaphthalen]-2-yl)-1*H*-imidazol-3-ium copper (II)-chloride (**C1i-(*R*,*R*)**) 34

Screening of the Sulfonyl Residues of Catalyst **C1**. 36

Investigation of Maleimide Dienophiles (**2**) and Maleic Anhydride (**6**) in the Diels-Alder Reaction with 3-Hydroxypyrone (**1a**). 37

Catalytic Diels–Alder Reactions of 3-Hydroxypyrone in Control Experiments........ 38

Catalytic Diels–Alder Reactions of 3-Hydroxypyrone in Control Experiments........ 39

Characterization of Diels-Alder Adducts 40

(3a*S*,4*S*,7*S*,7a*R*)-4,7-Ethenopyranol [3,4-*c*] pyrrole-1,3,6(2*H*)-trione-3a,4,7,7a-tetrahydro-7-hydroxy-2-methyl (***ent*-3aA**)........ 40

(3a*S*,4*S*,7*S*,7a*R*)-4,7-Ethenopyranol [3,4-*c*] pyrrole-1,3,6(2*H*)-trione-3a,4,7,7a-tetrahydro-7-hydroxy-2-(phenylmethyl) (***ent*-3aB**) 40

(3a*S*,4*S*,7*S*,7a*R*)-4,7-Ethenopyranol [3,4-*c*] pyrrole-1,3,6(2*H*)-trione-3a,4,7,7a-tetrahydro-7-hydroxy-2-(4-nitrophenyl) (***ent*-3aC**)........ 41

(3a*S*,4*S*,7*S*,7a*R*)-4,7-Ethenopyranol [3,4-*c*] pyrrole-1,3,6(2*H*)-trione-3a,4,7,7a-tetrahydro-7-hydroxy-2-cyclohexyl (***ent*-3aD**) 41

(3a*R*,4*R*,7*R*,7a*S*)-4,7-Ethenopyranol [3,4-*c*] pyrrole-1,3,6(2*H*)-trione-3a,4,7,7a-tetrahydro-7-hydroxy-2-*tert*-Butyloxycarbonyl (**3aE**) 42

(3a*R*,4*R*,7*R*,7a*S*)-4,7-Ethenopyranol [3,4-*c*] pyrrole-1,3,6(2*H*)-trione-3a,4,7,7a-tetrahydro-7-hydroxy (**3aF**) 42

(3a*S*,4*S*,7*S*,7a*R*)-4,7-Ethenopyranol [3,4-*c*] pyrrole-1,3,6(2*H*)-trione-3a,4,7,7a-tetrahydro-7-hydroxy-2-phenyl (***ent*-3aG**) 43

(3a*S*,4*S*,7*S*,7a*R*)-4,7-Ethenopyranol [3,4-*c*] pyrrole-1,3,6(2*H*)-trione-3a,4,7,7a-tetrahydro-7-hydroxy-2-(2,6-dimethoxyphenyl) (***ent*-3aH**) 44

(3a*S*,4*S*,7*S*,7a*R*)-4,7-Ethenopyranol [3,4-*c*] pyrrole-1,3,6(2*H*)-trione-3a,4,7,7a-tetrahydro-7-hydroxy-2-(3-chlorophenyl) (***ent*-3aI**) 44

(3a*S*,4*S*,7*S*,7a*R*)-4,7-Ethenopyranol [3,4-*c*] pyrrole-1,3,6(2*H*)-trione-3a,4,7,7a-tetrahydro-7-hydroxy-2-(4-(trifluoromethyl)phenyl) (***ent*-3aJ**) 45

(3a*S*,4*S*,7*S*,7a*R*)-4,7-Ethenopyranol [3,4-*c*] pyrrole-1,3,6(2*H*)-trione-3a,4,7,7a-tetrahydro-7-hydroxy-2-(3-nitrophenyl) (***ent*-3aK**) 45

(3a*S*,4*S*,7*S*,7a*R*)-4,7-Ethenopyranol [3,4-*c*] pyrrole-1,3,6(2*H*)-trione-3a,4,7,7a-tetrahydro-7-hydroxy-2-(4-chlorophenyl) (***ent*-3aL**) 46

(3a*S*,4*S*,7*S*,7a*R*)-4,7-Ethenopyranol [3,4-*c*] pyrrole-1,3,6(2*H*)-trione-3a,4,7,7a-tetrahydro-7-hydroxy-2-(2,4,6-trimethylphenyl) (***ent*-3aM**) 47

(3a*R*,4*R*,7*S*,7a*S*)-4,7-Ethenopyranol-(8-methyl) [3,4-*c*] pyrrole-1,3,6(2*H*)-trione-3a,4,7,7a-tetrahydro-7-hydroxy-2-(phenylmethyl) (**3bB**) 47

(3a*R*,4*S*,7*R*,7a*S*)-4,7-Ethenopyranol-(8-chloro) [3,4-*c*] pyrrole-1,3,6(2*H*)-trione-3a,4,7,7a-tetrahydro-7-hydroxy-2-(phenylmethyl) (**3cB**) 48

(3a*R*,4*S*,7*R*,7a*S*)-4,7-Ethenopyranol-(8-bromo) [3,4-*c*] pyrrole-1,3,6(2*H*)-trione-3a,4,7,7a-tetrahydro-7-hydroxy-2-(phenylmethyl) (**3dB**) 48

(3a*S*,4*S*,7*S*,7a*R*)-4,7-Etheno-1*H*-pyrrolo [3,4-*c*] pyridine-1,3,6(2*H,* 3a*H*)-trione-4,5,7,7a-tetrahydro-7-hydroxy-2-(methylphenyl)-5-[(2-nitrophenyl)sulfonyl] (***ent*-5aB**) 49

(3a*S*,4*S*,7*S*,7a*R*)-4,7-Etheno-1*H*-pyrrolo [3,4-*c*] pyridine-1,3,6(2*H,* 3a*H*)-trione-4,5,7,7a-tetrahydro-7-hydroxy-2-(4-nitrophenyl)-5-[(2-nitrophenyl)sulfonyl] (***ent*-5aC**) 49

(3a*S*,4*S*,7*S*,7a*R*)-4,7-Etheno-1*H*-pyrrolo [3,4-*c*] pyridine-1,3,6(2*H,* 3a*H*)-trione-4,5,7,7a-tetrahydro-7-hydroxy-5-[(2-nitrophenyl)sulfonyl] (***ent*-5aF**) 50

(3a*S*,4*S*,7*R*,7a*R*)-4,7-Etheno-1*H*-pyrrolo[3,4-*c*] pyridine-1,3,6(2*H,* 3a*H*)-trione-4,5,7,7a-tetrahydro-7-hydroxy-8-allyl-2-(methylphenyl)-5-[(2-nitrophenyl)sulfonyl] (***ent*-5bB**) 50

(3a*S*,4*R*,7*S*,7a*R*)-4,7-Etheno-1*H*-pyrrolo[3,4-*c*] pyridine-1,3,6(2*H,* 3a*H*)-trione-4,5,7,7a-tetrahydro-7-hydroxy-8-chloro-2-(methylphenyl)-5-[(2-nitrophenyl)sulfonyl] (***ent*-5cB**) 51

(3a*S*,4*S*,7*S*,7a*R*)-4,7-Etheno-1*H*-pyrrolo[3,4-*c*] pyridine-1,3,6(2*H,* 3a*H*)-trione-4,5,7,7a-tetrahydro-7-hydroxy-2-(methylphenyl) (***ent*-5dB**) 52

(3a*R*,4*R*,7*R*,7a*S*)-4,7-Ethenopyranol [3,4-*c*] pyrran-1,3,6(2*H*)-trione-3a,4,7,7a-tetrahydro-7-hydroxy (**7**) 52

Ethyl (3a*R*,4*S*,7*R*,7a*R*)-4,7-Methano-1*H*-isoindole-(2*H*,7*H*)-1,3,5-trione, tetrahydro- 2-(phenylmethyl)-4-carboxylate (**9**) 53

Ethyl (1*S*,2*S*,3*S*,4*R*)-3-Nitro-6-oxo-2-phenylbicyclo[2.2.1]heptane-1-carboxylate (**11**) 54

Confirming the Configurational Outcome with 2D-NMR Experiments of Compound **11**. 55

Derivatisation of the Catalytic Product **11**. 57

Ethyl (1*S*,2*S*,3*S*,4*R*,6*R*)-3-Nitro-6-hydroxy-2-phenylbicyclo[2.2.1]heptane-1-carboxylate (**11a**) 57

Catalyst Recycling 58
Mechanistic Study 59
Mass Spectrometric Experiments 59
^{1}H NMR Titration Experiments 60
UV-Vis Titration Experiments 60
Kinetic Experiments 61
Probing Catalyst Robustness and Product Influence 61
Raw Data and Calculated Concentrations for the “Same-Excess”-and “Product Addition”-Experiments 62
Kinetic Experiments–Determination of Reaction Orders Using Variable Time Normalization Graphical Analysis (VTNA) 63
Raw Data, Calculated Concentrations and Processed Data for the VTNA. 65
Investigation of a Possible Non-Linear-Effect 67
Crystallographic Data 68
Catalyst **C6** 68
NMR Data 69
Determination of Enantiomeric Excess 112
References 121

General Remarks

All reactions were performed in oven-dried glassware (stored in an oven, at 150 °C) and under a positive pressure of nitrogen, unless otherwise indicated. Technical grade solvents (dichloromethane (DCM), petroleum ether (PE), ethyl acetate (EE), diethylether (Et_2O), tetrahydrofuran (THF) and toluene) were distilled before use. Dry solvents like DCM, Et_2O, toluene, THF and acetonitrile were taken from solvent purification systems (MBraun MB SPS-800). Purchased chemicals were used without further purification. Analytical thin layer chromatography (TLC) was performed with silica gel 60F-254 TLC plates and compound spots were visualized by fluorescence quenching under UV light (254 nm) or by staining with $KMnO_4$/NaOH. Purification by flash-chromatography was performed on silica gel 60 (40-63 µm particle size), using a forced flow of eluent. All catalytic reactions were performed in oven dried Schlenk vials under a positive pressure of nitrogen unless otherwise indicated. In reactions where low temperatures were necessary a cryostatic temperature regulator was used. *n*-Heptane and *i*-propanol for HPLC were purchased in HPLC-quality and used without further purification.

N-Methyl maleimide **2A**, maleimide **2F**, *N*-Phenyl maleimide **2G**, maleic anhydride **6** and *trans*-β-nitrostyrene **10** were purchased from commercial supplier (Sigma-Aldrich) and were used without further purification. All other maleimides were synthesized according to literature known procedures.[1] 2-Pyrones **1a**[2], **1b**[3], **1d**[4] and pyridone **4a**[5] were prepared according to literature methods.

NMR data were recorded on *Bruker Avance* spectrometers operating at Larmor frequencies of 700, 500, 400 or 300 MHz (^{1}H), 176, 125, 100 or 75 MHz (^{13}C) and 376 MHz (^{19}F). Chemical shifts *δ* are referred in terms of ppm. *J*-Coupling constants are given in Hz. The following abbreviations classify the multiplicity: *s* (singlet), *d* (doublet), *t* (triplet), *q* (quartet), *quint* (quintet), *sept* (septet), *m* (multiplet), *dd* (doublet of doublets), *dt* (doublet of triplets), *td* (triplet of doublets) and *br* (broad signal). Infrared spectra were recorded by the IR service of the University of Stuttgart on an FT-IR spectrometer (*Bruker Alpha FT-IR*) with an ATR unit and the signals are given by wavenumbers (cm^{-1}). Optical rotation was measured on a *Perkin Elmer 241 Polarimeter* operating at the sodium D line (λ = 589 nm) and mercury lines (λ_1 = 578 nm and λ_2 = 546 nm) with a 100 mm path cell length. Melting points were measured using a melting point apparatus (*Büchi 535*) in open glass capillaries. Mass spectra were measured on a *Finnigan MAT 95* for CI and EI and a *Bruker MicroTOFQ* for ESI and obtained from the MS service of the University of Stuttgart. The UV-Vis spectra were recorded with a *Lambda 365-Spectrometer* (*PerkinElmer*). Single crystal X-ray analysis was performed by Dr. Wolfgang Frey, University of Stuttgart, on a *Bruker Kappa APEXII Duo* (Cu K_α 1.54178Å and Mo K_α 0.71073 Å). Enantiomeric excesses (*ee*) were determined by high performance liquid chromatography (HPLC) or NMR spectroscopy using the (*R*)-BINOL. The applied method is given in the description of the corresponding product.

The catalysts **C1a**, **C2**, **C3**, **C4**, **C5** and **C6** were synthesized according to our previously described protocols.[6]

General Procedures (GP)

General Procedure for the Imine-Synthesis (GP1)

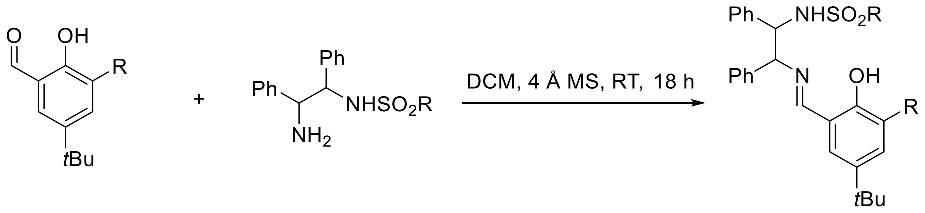

The corresponding ligands were synthesized following the literature procedure.[6] The corresponding aldehyde (1.0 equiv.) and the corresponding amine (1.0 equiv.) were dissolved in dry DCM (5 mL / mmol) in the presence of molecular sieves (4 Å) under nitrogen atmosphere and the reaction mixture was stirred for 18 h. After that, the reaction mixture was filtered through celite, the filter cake washed with dry DCM (5 mL/1 mmol) and the solvent removed under reduced pressure. The resulting yellow solid was dissolved in a small amount of dry DCM (0.2 mL/mmol) and added to *n*-pentane (2 mL/mmol). The formed precipitate was filtered to afford the pure ligand as a yellow solid.

General Procedure for the Metal Complexation (GP2)

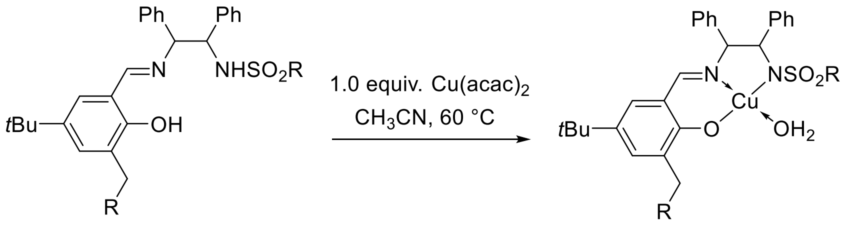

Based on a literature protocol,[6] the corresponding ligand (1.0 equiv.) was dissolved in dry acetonitrile (5 mL / 0.01 mmol) and the corresponding metal source ($Cu(acac)_2$, 1.0 equiv.) was added and the mixture was stirred at 60 °C for 16 h. The solution was filtered over celite and the filter cake washed with DCM. Subsequently, the solvent was removed under reduced pressure. The residue was dissolved in a small amount of DCM and the product was precipitated by adding *n*-pentane to the solution. The suspension was centrifuged, the supernatant solution was decanted off and the residue was then dried under high vacuum to afford the corresponding pre-catalyst.

General Procedure for the Activation of the Complexes (GP3)

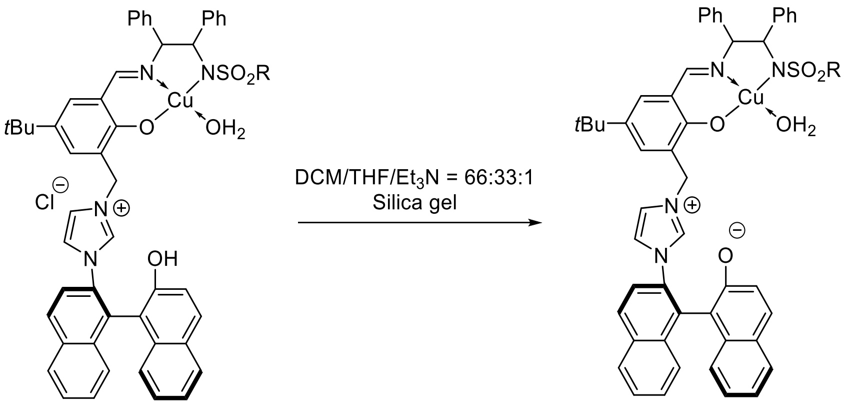

Based on a literature protocol,[6] the complexes were dissolved in a solvent mixture of DCM/THF/Et_3N (66/33/1) and the solution was filtered over a small silica pad in a glass frit and eluted with the same mixture. The volatiles were removed under reduced pressure and the product (activated catalyst) was dissolved in a small amount of DCM (0.1 mL), precipitated in *n*-pentane (5 mL), filtered and dried under high vacuum for 2 h. The activated catalyst could be used without further purification in the catalytic DA reactions.

General Procedure for Catalytic Diels−Alder Reactions of 3-Hydroxypyrones (GP4)

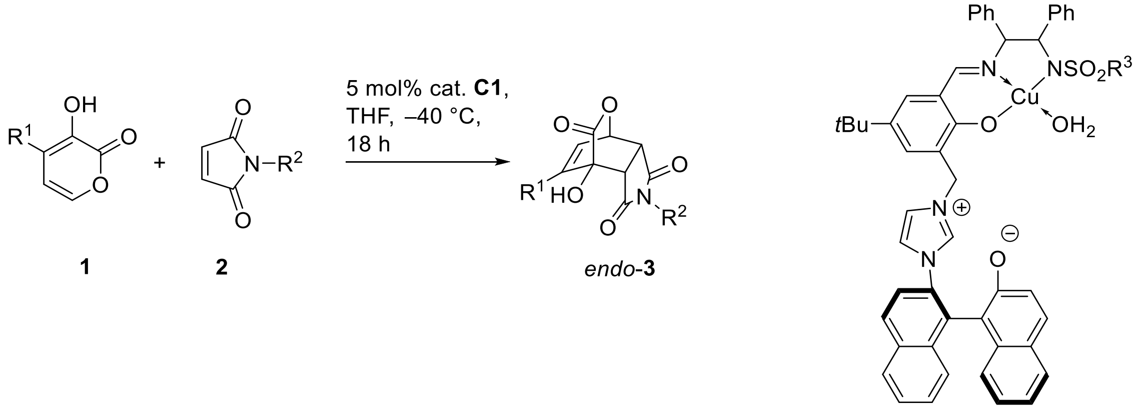

1 **2** *endo*-**3** **C1**

To an oven-dried Schlenk vial containing activated catalyst **C1** (0.005 mmol, 5 mol%) and *N*-substituted maleimide **2** (0.105 mmol, 1.05 equiv.) dry THF (0.1 mL) was added. The solution was placed in a cryobath at −40 °C and allowed to stir for 10 min under nitrogen atmosphere. The corresponding diene **1** (0.1 mmol, 1.0 equiv.) was then added using a syringe pump over a period of 12 h as a stock solution (in 0.1 mL THF) followed by additional solvent (0.05 mL) to avoid a loss of the material on the glass wall. After the addition period was completed the reaction mixture was stirred for six hours. Afterwards the reaction mixture was filtered through a short pad of silica to remove the catalyst using a mixture of petroleum ether/ethyl acetate 1:1 as eluent. The crude product was purified by flash column chromatography (PE : EE = 2:1) to yield the pure product.

General Procedure for Catalytic Diels−Alder Reactions of 3-Hydroxypyrones in Control Experiments (GP5)

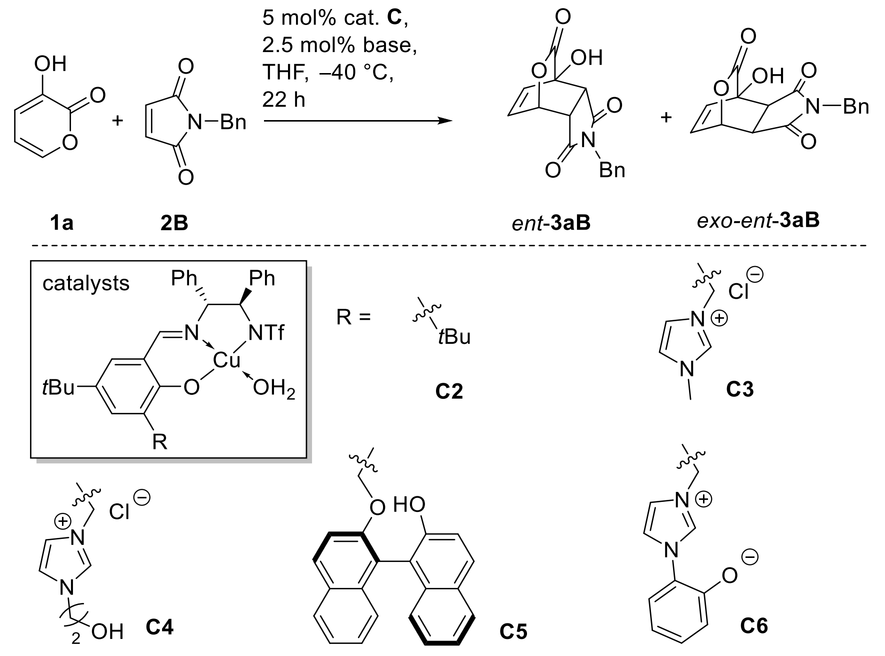

To an oven-dried Schlenk vial containing the corresponding catalyst (**C2-C5**) (0.05 mmol, 5 mol%), the corresponding base (0.0025 mmol, 2.5 mol%) and maleimide **2B** (0.105 mmol, 1.05 equiv.) THF (0.1 mL) was added. The reaction mixture was placed in a cryobath at −40 °C and allowed to stir for 10 min under nitrogen atmosphere. The diene **1a** (0.1 mmol, 1.0 equiv.) was then added using a syringe pump over the period of 12 h as a stock solution (in 0.1 mL THF, plus additional solvent 0.05 mL to avoid loss of the material on the glass wall. After the addition period was completed the reaction mixture was stirred for additional 10 hours. Afterwards the reaction mixture was filtered through a short pad of silica to remove the catalyst using a mixture of petroleum ether/ethyl acetate (1:1) as eluent. The crude product was purified by flash column chromatography (PE : EE = 2:1) to yield the pure product.

General Procedure for the Catalytic Diels−Alder Reactions of 3-Hydroxypyridones (GP6)

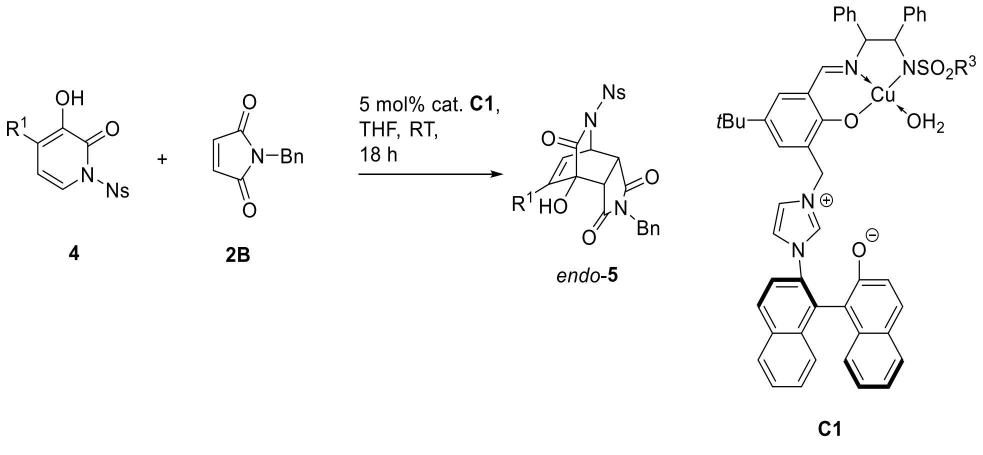

To an oven-dried Schlenk vial containing activated catalyst **C1** (0.005 mmol, 5.0 mol%), and maleimide **2B** (0.105 mmol, 1.05 equiv.) 0.1 mL of dried THF was added at room temperature. The solution was allowed to stir for 10 min under nitrogen atmosphere. The corresponding diene **4** (0.1 mmol, 1.0 equiv.) was then added using a syringe pump over the period of 12 h as a stock solution (in 0.1 mL THF) followed by additional solvent (0.05 mL) to avoid a loss of the material on the glass wall. After the addition period was completed the reaction mixture was stirred for additional six hours. Afterwards the reaction mixture was filtered through a short pad of silica to remove the catalyst using a mixture of petroleum ether/ethyl acetate 1:1 as eluent. The crude product was purified by flash column chromatography (PE : EE = 2:1) to yield the pure product.

General Procedure for the Cycloaddition Reaction with Enone (8) (GP7)

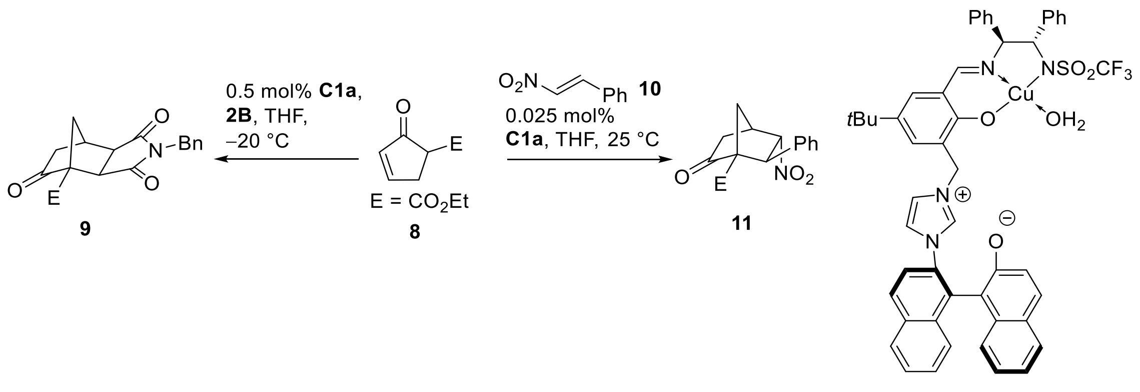

C1a-(*S*,*S*)

The activated catalyst **C1a-(*S*,*S*)** was added as a stock solution (0.05 mL) in anhydrous THF to a catalysis tube containing *N*-benzylmaleimide **2B** (18.7 mg, 0.10 mmol, 1.0 equiv.) or nitroolefin **10** (14.9 mg, 0.1 mmol, 1.0 equiv.) and enone **8** (18.4 mg, 0.12 mmol, 1.2 equiv.) under nitrogen atmosphere. The reaction mixture was stirred for 20 h. Afterwards the reaction mixture was diluted with a solvent mixture of petroleum ether/ethyl acetate (1/1, 2 mL), filtered through a small pad of silica to remove the catalyst from the reaction mixture and the crude product was eluted with additional petroleum ether/ethyl acetate (1/1, 10 mL). After removal of solvent under reduced pressure, the crude product was purified via column chromatography with petroleum ether/ethyl acetate as eluent (4/1) to yield the pure product.

General Procedure for the Determination of *ee* values with (*R*)-BINOL (GP8)

To an NMR tube filled with 0.4 mL of saturated $CDCl_3$ solution of (*R*)-BINOL was added the corresponding cycloaddition product (1.0 mg, dissolved in 0.1 mL of $CDCl_3$). The ^1H NMR spectra were recorded at 500 or 700 MHz. The enantiomeric excesses were determined by integration of characteristic signals of the (+) and (–)-enantiomers.[7]

Substrate Synthesis

4-Chloro-3-hydroxy-2-pyrone (1c)

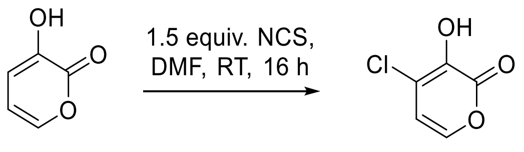

To a solution of 3-hydroxy-2-pyrone **1a** (0.40 g, 3.57 mmol) in DMF (10 mL) was added NCS (0.71 g, 5.36 mmol, 1.50 equiv.) portionwise over the period of 15 min. The reaction mixture was stirred for 16 h at room temperature and diluted with 10 mL of H_2O and extracted with EtOAc (3 x 10 mL). The combined organic layers were dried over Na_2SO_4, filtrated and concentrated *in vacuo*. The residue was purified by column chromatography (using DCM as eluent) to afford pyrone **1c** (261.5 mg, 1.78 mmol, 50%) as a yellow crystalline solid.

$C_5H_3ClO_3$, *M*: 146.53 g/mol. **^{1}H NMR (300 MHz, $CDCl_3$):** *δ* (ppm) = 7.07 (*d*, *J* = 5.7 Hz, 1H, CH=C*H*-O), 7.05-6.76 (*br*, 1H, O*H*), 6.27 (*d*, *J* = 5.7 Hz, 1H, C*H*=CH-O).

The NMR spectra is in agreement to the one reported in the literature.[8]

N-2-Nitrobenzenesulfonyl-4-allyl-3-hydroxy-2-pyridone (4b)

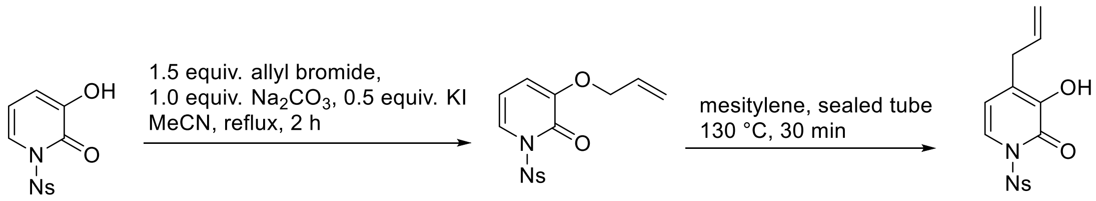

Following the literature procedure,[9] to a mixture of **4a** (592.5 mg, 2.0 mmol, 1.0 equiv.), Na_2CO_3 (212.0 mg, 2.0 mmol, 1.0 equiv.) and KI (83 mg, 1.0 mmol, 0.5 equiv.) in MeCN (5 mL) was added allyl bromide (259.6 µL, 3.0 mmol, 1.1 equiv.), and the reaction mixture was heated at reflux for two hours. After cooling to room temperature, water (10 mL) was added and the mixture was extracted with EtOAc (3 x 10 mL). The combined organic phases were dried over $MgSO_4$ and concentrated under reduced pressure. The obtained *O*-allyl pyridone was dissolved in 5 mL of mesitylene in a pressure tube and heated at 130 °C for 30 min. After cooling to room temperature the reaction mixture was directly purified by column chromatography (PE : EE = 2 : 1). **4b** was isolated as a light-yellow solid (98.9 mg, 0.29 mmol, 35%, over two steps).

$C_{14}H_{12}N_2O_6S$, *M*: 336.32 g/mol. **m.p.** = 181 °C. **^{1}H NMR (400 MHz, $CDCl_3$):** *δ* (ppm) = 8.70-8.50 (*m*, *br*, 2H, Ar*H*, O*H*), 7.96-7.84 (*m*, 3H, Ar*H*), 7.41 (*d*, *J* = 7.8 Hz, 1H, NC*H*=CH), 6.23 (*d*, *J* = 7.9 Hz, 1H, NCH=C*H*), 5.95-5.80 (*m*, 1H, C*H*=CH_{2allyl}), 5.12 (*d*, *J* = 17.3 Hz, 1H, CH=CH_{2allyl}), 5.05 (*d*, *J* = 10.3 Hz, 1H, CH=CH_{2allyl}), 3.26 (*d*, *J* = 6.6 Hz, 2H, OCH_2). **^{13}C NMR (176 MHz, $CDCl_3$):** *δ* (ppm) = 157.0, 149.3, 144.8, 137.0, 136.9, 134.9, 132.4, 130.5, 128.7, 125.5, 121.3, 116.9, 109.3, 33.6. **IR (solid)** $\tilde{\nu}$ = 3289, 3103, 2979, 1638, 1616, 1541, 1440,

1362, 1284, 1237, 1179, 1128, 1054, 927, 852, 781, 737, 657, 593, 556 cm^{-1}. **HRMS (ESI)**: m/z calculated for $[C_{14}H_{12}N_2O_6SNa]^+$: 359.0308, found: 359.0308.

N-2-Nitrobenzenesulfonyl-4-chloro-3-hydroxy-2-pyridone (4c)

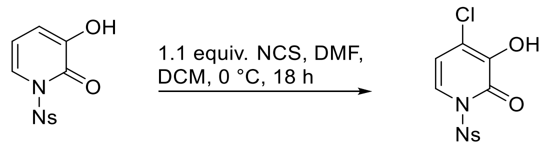

4a **4c**

To a solution of *N*-2-nitrobenzenesulfonyl-3-hydroxy-2-pyridone **4a** (0.50 g, 1.67 mmol) in DMF (10 mL) was added NCS (247.9 mg, 1.85 mmol, 1.1 equiv.) portionwise. The reaction mixture was stirred for 18 h at 0 °C and diluted with 15 mL of H_2O and extracted with EtOAc (3 x 10 mL). The combined organic layers were dried over $MgSO_4$, filtrated and concentrated *in vacuo*. The residue was purified by column chromatography (PE : EE = 2:1) to afford pyridone **4c** (247.5 mg, 0.95 mmol, 56%) as a yellow crystalline solid.

$C_{11}H_7ClN_2O_6S$, *M*: 330.69 g/mol. **m.p**. = 187 °C. **^{1}H NMR (400 MHz, THF-d$_8$):** δ (ppm) = 9.52 (*s*, 1H, O*H*), 8.62 (*d*, *J* = 6.6 Hz, 1H, Ar*H*), 8.01-7.84 (*m*, 3H, Ar*H*), 7.47 (*d*, *J* = 8.1 Hz, NC*H*=CH), 6.43 (*d*, *J* = 8.1 Hz, 1H, NCH=C*H*). **^{13}C NMR (100 MHz, THF-d$_8$):** δ (ppm) = 156.4, 149.3, 145.1, 137.2, 137.0, 132.6, 130.1, 125.7, 123.5, 121.6, 109.4. **IR (solid)** $\tilde{\nu}$ = 3304, 3110, 1957, 1646, 1542, 1442, 1383, 1361, 1266, 1184, 1148, 1125, 1025, 945, 896, 853, 782, 739, 657, 588, 563 cm^{-1}. **HRMS (ESI)**: m/z calculated for $[C_{11}H_7ClN_2O_6SNa]^+$: 352.9606, found: 352.9607.

Ligand Synthesis

(*R*)-2-(Imidazol-1-yl)-2′-hydroxy-1,1′-binaphthyl (K1)

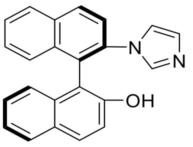

K1

Imidazole derivative **K1** was synthesized using a modification of the procedure by Crabtree *et al.*[10] To (*R*)-2,2-diamino-1,1'-binaphthyl ((*R*)-BINAM) (500 mg, 1.76 mmol, 1.0 equiv.) 10 mL of demineralized water was added, and then 478 µL of concentrated HBr (5.0 equiv.). The mixture was stirred for 5 min, and 40% aqueous glyoxal (1.0 mL, 8.79 mmol, 5.0 equiv.) and paraformaldehyde (264 mg, 8.79 mmol, 5.0 equiv.) were added followed by addition of 10 mL of 1,4-dioxane. The mixture was heated with stirring to 80 °C, and ammonium chloride (470.3 mg, 8.79 mmol, 5.0 equiv.) was added. The solution was refluxed for 5 h and cooled to room temperature. A saturated aqueous solution of K_2CO_3 (10 mL) was added, and the mixture was extracted with dichloromethane (3 x 10 mL). The organic phases were combined, dried over Na_2SO_4, and filtered. The solvent was evaporated under reduced pressure, and the crude residue was purified by column chromatography on silica gel (dried in an oven overnight, at 150 °C) using a mixture of acetone : MeOH = 20:1. Pure product was obtained in 92% yield

(544 mg, 1.62 mmol) after additional precipitation in *n*-pentane (30 mL) from a DCM solution (5 mL).

$C_{23}H_{16}N_2O$, *M*: 336.39 g/mol. **^{1}H NMR (300 MHz, $CDCl_3$)**: *δ* (ppm) = 8.06 (*d*, *J* = 8.6 Hz, 1H, Ar*H*), 7.98 (*d*, *J* = 8.6 Hz, 1H, Ar*H*), 7.86-7.78 (*m*, 2H, Ar*H*), 7.62-7.48 (*m*, 3H, Ar*H*), 7.45-7.32 (*m*, 2H, Ar*H*), 7.31-7.22 (*m*, 1H, Ar*H*), 7.22-7.12 (*m*, 1H, Ar*H*), 6.90-6.79 (*m*, 3H, Ar*H*, NC*H*), 6.74 (*d*, *J* = 8.6 Hz, 1H, NC*H*).

The NMR spectra is in agreement to the one reported in the literature.[10]

(*R*)-3-(5-(*tert*-Butyl)-3-formyl-2-hydroxybenzyl)-1-(2'-hydroxy-[1,1'-binaphthalen]-2-yl)-1H-imidazol-3-ium chloride (K2)

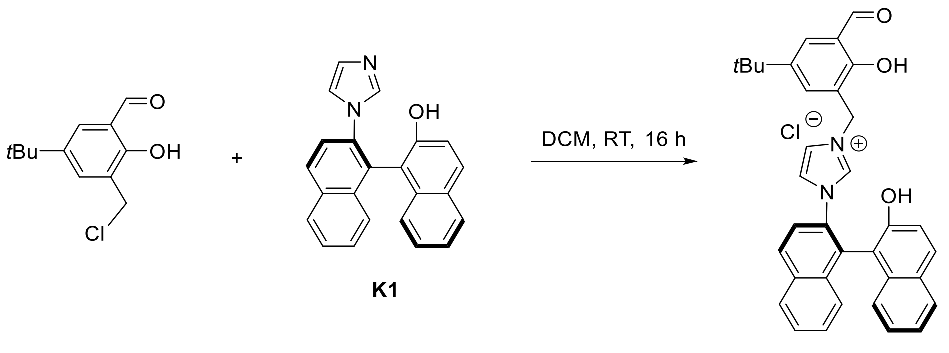

According to a literature protocol,[6] 3-(Chloromethyl)-5-(*tert*-butyl)-2-hydroxybenzaldehyde (202.2 mg, 0.89 mmol, 1.0 equiv.) was dissolved in 5 mL DCM and added to a solution of **K1** (300.0 mg, 0.89 mmol, 1.0 equiv.) in DCM (5 mL). The solution was stirred at room temperature for 16 h. The solvent was removed under reduced pressure, and the residue was redissolved in a small amount of dichloromethane (2 mL) and added to a stirred solution of diethylether (20 mL) to cause precipitation. The precipitate was filtered, washed with diethylether (10 mL) and resulting solid was dried in vacuo. The imidazolium salt **K2** was isolated as a beige solid (489.9 mg, 0.87 mmol, 98%).

$C_{35}H_{31}ClN_2O_3$, ***M***: 563.09 g/mol. **^{1}H NMR (300 MHz, $CDCl_3$):** *δ* (ppm) = 11.11 (*s*, 1H, C*H*O), 9.85 (*s*, 1H, ArO*H*), 9.82 (*s*, 1H, ArO*H*), 8.34 (*d*, *J* = 2.3 Hz, 1H, NC*H*N), 8.07 (*d*, *J* = 8.7 Hz, 1H, Ar*H*), 7.98 (*d*, *J* = 8.5 Hz, 1H, Ar*H*), 7.76-7.34 (*m*, 9H, Ar*H*), 7.22-7.02 (*m*, 3H, Ar*H*), 6.85-6.70 (*m*, 2H, NC*H*), 5.80-5.56 (*m*, 2H, $CH_2N_{imidazole}$), 1.34 (*s*, 9H, CH_3).

The NMR spectra is in agreement to the one reported in the literature.[6]

3-(5-(*tert*-Butyl)-2-hydroxy-3-((*E*)-(((1*S*,2*S*)-2-(naphthalene-1-sulfonamido)-1,2-diphenylethyl)imino)methyl)benzyl)-1-((*R*)-2'-hydroxy-[1,1'-binaphthalen]-2-yl)-1*H*-imidazol-3-ium-chloride (L1b-(*S*,*S*))

Ligand **L1b-(*S*,*S*)** was synthesized according to **GP1** using aldehyde **K2** (20.0 mg, 0.035 mmol, 1.0 equiv.) and *N*-((1*S*,2*S*)-2-Amino-1,2-diphenylethyl)-naphthalene-1-sulfonamide (14.3 mg, 0.035 mmol, 1.0 equiv.). The product was isolated as a yellow solid (32.7 mg, 0.034 mmol, 96%).

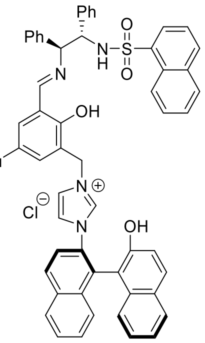

L1b-(*S*,*S*)

$C_{59}H_{51}ClN_4O_4S$, *M*: 947.59 g/mol. **m.p.** = 218 °C. **$[\alpha]^{20}_D$** (c = 1.00 mg/mL, DCM): −40.7. **^{1}H NMR (400 MHz, $CDCl_3$):** *δ* (ppm) = 13.68-12.81 (*br*, 1H, ArO*H*), 10.89-9.57 (*br*, 1H, ArO*H*), 9.30 (*s*, 1H, C*H*N), 8.49 (*d*, *J* = 8.8 Hz, 1H, Ar*H*), 8.01 (*d*, *J* = 7.6 Hz, 1H, Ar*H*), 7.89 (*d*, *J* = 7.4 Hz, 1H, Ar*H*), 7.87-7.65 (*m*, 7H, Ar*H*), 7.65-7.29 (*m*, 8H, Ar*H*), 7.22-6.87 (*m*, 13H, Ar*H*), 6.83 (*m*, 2H, N-C*H*-CH-N), 6.59 (*s*, 1H, NC*H*N), 5.52 (*d*, *J* = 14.0 Hz, 1H, ArC*H*$_2$-N), 5.13 (*d*, *J* = 14.0 Hz, 1H, ArC*H*$_2$N), 4.88 (*d*, *J* = 8.1 Hz, 1H, C=N-C*H*Ph), 4.72 (*d*, 1H, SO$_2$NH-C*H*Ph), 1.11 (*s*, 9H, C(C*H*$_3$)$_3$). **^{13}C NMR (176 MHz, $CDCl_3$):** *δ* = 167.1, 157.1, 153.8, 141.8, 139.0, 138.4, 136.7, 135.5, 134.1, 133.8, 133.7, 133.6, 133.1, 132.5, 132.1, 131.7, 130.7, 129.9, 129.8, 129.0, 128.5, 128.3, 128.2, 128.1, 128.0, 127.8, 127.7, 127.7, 127.6, 127.5, 127.0, 126.8, 126.3, 124.4, 123.6, 123.5, 123.0, 122.5, 122.3, 122.1, 120.4, 120.2, 117.9, 113.5, 64.4, 48.6, 34.0, 31.4. **IR (solid):** $\tilde{\nu}$ = 3062, 2963, 1626, 1597, 1508, 1434, 1345, 1324, 1161, 1134, 910, 819, 771, 731, 699, 587 cm^{-1}. **HRMS (ESI)**: m/z calculated for $[C_{59}H_{51}N_4O_4S]^+$: 911.3626, found: 911.3627.

3-(5-(*tert*-Butyl)-2-hydroxy-3-((*E*)-(((1*R*,2*R*)-2-(naphthalene-1-sulfonamido)-1,2-diphenylethyl)imino)methyl)benzyl)-1-((*R*)-2'-hydroxy-[1,1'-binaphthalen]-2-yl)-1*H*-imidazol-3-ium-chloride (L1b-(*R*,*R*))

Ligand **L1b-(*R*,*R*)** was synthesized according to **GP1** using aldehyde **K2** (20.0 mg, 0.035 mmol, 1.0 equiv.) and *N*-((1*R*,2*R*)-2-Amino-1,2-diphenylethyl)-naphthalene-1-sulfonamide (14.3 mg, 0.035 mmol, 1.0 equiv.). The product was isolated as a yellow solid (29.6 mg, 0.031 mmol, 88%).

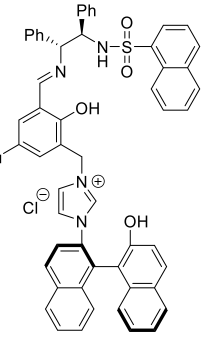

L1b-(*R*,*R*)

$C_{59}H_{51}ClN_4O_4S$, *M*: 947.59 g/mol. **m.p.** = 208 °C. **$[\alpha]^{20}_D$** (c = 1.00 mg/mL, DCM): 73.4. **^{1}H NMR (400 MHz, $CDCl_3$):** *δ* (ppm) = 14.24 (*br. s*, 1H, ArO*H*), 10.91-10.14 (*br*, 1H, ArO*H*), 9.78 (*s*, 1H, C*H*N), 9.44-9.25 (*br*, 1H, SO$_2$N*H*), 8.62 (*d*, *J* = 8.5 Hz, 1H, Ar*H*), 8.24 (*s*, 1H, Ar*H*), 8.11 (*s*, 1H, Ar*H*), 7.99 (*d*, *J* = 7.6 Hz, 1H, Ar*H*), 7.78 (*d*, *J* = 8.8 Hz, 1H, Ar*H*), 7.67-7.58 (*m*, 4H, Ar*H*), 7.55-7.47 (*m*, 2H, Ar*H*), 7.43-7.33 (*m*, 4H, Ar*H*), 7.20-6.77 (*m*, 14H, Ar*H*, 1H, NC*H*N), 6.59 (*d*, *J* = 9.0 Hz, 1H, N-C*H*-CH-N), 6.46 (*d*, *J* = 8.0 Hz, 1H, N-C*H*-CH-N), 6.40 (*t*, *J* = 6.7 Hz, 2H, Ar*H*), 5.75 (*d*, *J* = 14.0 Hz, 1H, ArC*H*$_2$N), 5.61 (*d*, *J* = 13.0 Hz, 1H, ArC*H*$_2$N), 5.36 (*d*, *J* = 10.0 Hz, 1H, HC=N-C*H*Ph), 4.80 (*t*, *J* = 10.0 Hz, 1H, HNC*H*Ph), 1.27 (*s*, 9H, C(C*H*$_3$)$_3$).

^{13}C NMR (176 MHz, CDCl$_3$): δ = 167.2, 157.4, 135.4, 141.2, 139.9, 137.1, 136.2, 133.7, 133.5 133.4, 133.3, 133.0, 131.9, 131.4, 130.4, 130.3, 129.9, 129.7, 128.8, 128.4, 128.3, 128.2, 128.1, 128.0, 127.9, 127.6, 127.5, 127.2, 127.0, 126.9, 126.8, 126.6, 126.4, 126.2, 126.1, 126.0, 125,2, 123.9, 123.8, 123.5, 123.3, 123.0, 122.8, 122.7, 121.9, 121.3, 120.2, 118.2, 117.8, 114.6, 114.1, 72.8, 64.6, 58.5, 48.5, 34.2, 31.5, 18.5. **IR (solid):** $\tilde{\nu}$ = 3059, 2958, 1626, 1599, 1319, 1159, 1132, 909, 817, 771, 729, 698, 587 cm^{-1}. **HRMS (ESI)**: m/z calculated for [$C_{59}H_{51}N_4O_4S$]$^+$: 911.3626, found: 911.3630.

3-(5-(*tert*-Butyl)-2-hydroxy-3-((*E*)-(((1*S*,2*S*)-2-(anthrachene-9-sulfonamido)-1,2-diphenylethyl)imino)methyl)benzyl)-1-((*R*)-2'-hydroxy-[1,1'-binaphthalen]-2-yl)-1*H*-imidazol-3-ium-chloride (L1c-(*S*,*S*))

Ligand **L1c-(*S*,*S*)** was synthesized according to **GP1** using aldehyde **K2** (17.2 mg, 0.031 mmol, 1.0 equiv.) and *N*-((1*S*,2*S*)-2-Amino-1,2-diphenylethyl)-anthrachene-9-sulfonamide (13.86 mg, 0.031 mmol, 1.0 equiv.). The product was isolated as a yellow solid (26.3 mg, 0.026 mmol, 85%).

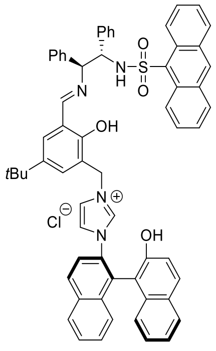

L1c-(*S*,*S*)

$C_{63}H_{53}ClN_4O_4S$, *M*: 997.64 g/mol. **m.p**. = 246 °C. **[α]$^{20}_{D}$** (c = 1.00 mg/mL, DCM): –32.0. **^{1}H NMR (700 MHz, (CD$_3$)$_2$SO):** δ (ppm) = 12.99 (*s*, 1H, ArO*H*), 9.95 (*s*, 1H, ArO*H*), 9.15 (*d*, *J* = 9.4 Hz, 2H, *H*C=N), 8.98 (*s*, 1H, Ar*H*), 8.94 (*d*, *J* = 5.8 Hz, 1H, SO$_2$N*H*), 8.63 (*s*, 1H, Ar*H*), 8.56 (*s*, 1H, Ar*H*), 8.28 (*d*, *J* = 9.1 Hz, 1H, Ar*H*), 8.15 (*d*, *J* = 9.1 Hz, 1H, Ar*H*), 7.94 (*d*, *J* = 8.5 Hz, 2H, Ar*H*), 7.84 (*d*, *J* = 8.5 Hz, 1H, Ar*H*), 7.66 (*t*, *J* = 8.5 Hz, 1H, Ar*H*), 7.58-7.55 (*m*, 3H, Ar*H*), 7.52-7.49 (*m*, 2H, Ar*H*), 7.46-7.39 (*m*, 4H, Ar*H*), 7.22 (*m*, 3H, Ar*H*), 7.16-7.00 (*m*, 10H, Ar*H*), 6.74-6.68 (*m*, 4H, Ar*H,* NC*H*), 6.65-6.61 (*m*, 2H, Ar*H,* NC*H*), 5.15 (*q*, *J* = 29.0, 14.1 Hz, 2H, $N_{imidazole}CH_2$), 4.85 (*t*, *J* = 9.8 Hz, 1H, SO$_2$NHC*H*Ph), 4.61 (*d*, *J* = 9.8 Hz, 1H, C=NC*H*Ph), 1.23 (*s*, 9H, C(C*H*$_3$)$_3$). **^{13}C NMR (700 MHz, (CD$_3$)$_2$SO)):** δ (ppm) = 167.8, 167.2, 156.8, 153.5, 141.0, 139.8, 138.6, 136.8, 135.9, 135.1, 135.0, 134.0, 133.5, 133.3, 132.6, 132.5, 131.20, 131.17, 131.07, 130.9, 130.6, 130.5, 130.3, 129.8, 129.6, 129.5, 128.9, 128.8, 128.5, 128.25, 128.23, 128.1, 127.9, 127.8, 127.4, 127.3, 127.2, 127.0, 126.9, 125.5, 125.3, 124.0, 123.9, 123.4, 123.2, 122.8, 120.2, 118.5, 118.4, 118.0, 112.6, 77.6, 63.2, 48.0, 40.5, 34.1, 31.6. **IR (solid):** $\tilde{\nu}$ = 3061, 2957, 2924, 1717, 1625, 1601, 1546, 1510, 1478, 1454, 1434, 1364, 1344, 1320, 1275, 1203, 1158, 1145, 1098, 1064, 1027, 945, 908, 818, 778, 734, 698, 671, 581, 516 cm^{-1}. **HRMS (ESI)**: m/z calculated for [$C_{63}H_{53}N_4O_4S$]$^+$: 961.3782, found: 961.3780.

3-(5-(*tert*-Butyl)-2-hydroxy-3-((*E*)-(((1*S*,2*S*)-2-(naphthalene-2-sulfonamido)-1,2-diphenylethyl)imino)methyl)benzyl)-1-((*R*)-2'-hydroxy-[1,1'-binaphthalen]-2-yl)-1*H*-imidazol-3-ium-chloride (L1d-(*S*,*S*))

Ligand **L1d-(*S*,*S*)** was synthesized according to **GP1** using aldehyde **K2** (35.0 mg, 0.062 mmol, 1.0 equiv.) and *N*-((1*S*,2*S*)-2-Amino-1,2-diphenylethyl)-naphthalene-2-sulfonamide (25.02 mg, 0.062 mmol, 1.0 equiv.). The product was isolated as a yellow solid (55.8 mg, 0.059 mmol, 95%).

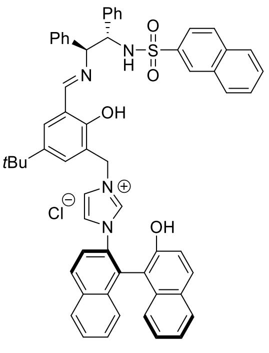

L1d-(*S*,*S*)

$C_{59}H_{51}ClN_4O_4S$, *M*: 947.59 g/mol. **m.p**. = 190 °C. **$[\alpha]^{20}_{D}$** (c = 1.00 mg/mL, DCM): –2.2. **^{1}H NMR (400 MHz, $CDCl_3$):** δ (ppm) = 14.08-12.35 (*br*, 1H, ArO*H*), 10.45-9.48 (*br*, 1H, ArO*H*), 9.30 (*s*, 1H, *H*C=N), 8.50 (*s*, 1H, SO_2N*H*), 7.93 (*s*, 1H, Ar*H*), 7.80-7.55 (*m*, 6H, Ar*H*), 7.49 (*t*, *J* = 7.5 Hz, 1H, Ar*H*), 7.44-7.21 (*m*, 10H, Ar*H*), 7.13-7.01 (*m*, 3H, Ar*H*), 7.01-6.81 (*m*, 14H, Ar*H,* NC*H*), 6.42 (*s*, 1H, NC*H*), 5.29 (*d*, *J* = 13.7 Hz, 1H, SO_2NHC*H*Ph,), 4.94-4.62 (*m*, 3H, C=NC*H*Ph, $N_{imidazole}CH_2$), 1.00 (*s*, 9H, $C(CH_3)_3$). **^{13}C NMR (176 MHz, $CDCl_3$):** δ (ppm) = 167.6, 157.1, 153.7, 141.9, 139.3, 138.8, 138.2, 136.9, 134.1, 134.1, 133.7, 133.2, 131.9, 131.8, 130.9, 130.0, 129.9, 129.0, 128.8, 128.6, 128.4, 128.4, 128.3, 128.0, 127.9, 127.8, 127.7, 127.6, 127.3, 127.2, 127.1, 127.0, 123.6, 123.2, 122.7, 122.3, 122.2, 122.0, 120.5, 120.2, 118.1, 113.6, 76.6, 64.5, 48.7, 34.1, 31.5. **IR (solid):** $\tilde{\nu}$ = 3057, 2959, 1625, 1599, 1545, 1506, 1478, 1454, 1433, 1320, 1273, 1201, 1153, 1130, 1096, 1074, 1028, 955, 908, 858, 816, 773, 747, 728, 698, 661, 643, 626, 593, 562, 546, 480 cm^{-1}. **HRMS (ESI)**: m/z calculated for $[C_{59}H_{51}N_4O_4S]^+$: 911.3626, found: 911.3627.

3-(5-(*tert*-Butyl)-2-hydroxy-3-((*E*)-(((1*R*,2*R*)-2-(naphthalene-2-sulfonamido)-1,2-diphenylethyl)imino)methyl)benzyl)-1-((*R*)-2'-hydroxy-[1,1'-binaphthalen]-2-yl)-1*H*-imidazol-3-ium-chloride (L1d-(*R*,*R*))

Ligand **L1d-(*R*,*R*)** was synthesized according to **GP1** using aldehyde **K2** (35.0 mg, 0.062 mmol, 1.0 equiv.) and *N*-((1*R*,2*R*)-2-Amino-1,2-diphenylethyl)-naphthalene-2-sulfonamide (25.02 mg, 0.062 mmol, 1.0 equiv.). The product was isolated as a yellow solid (54.9 mg, 0.058 mmol, 94%).

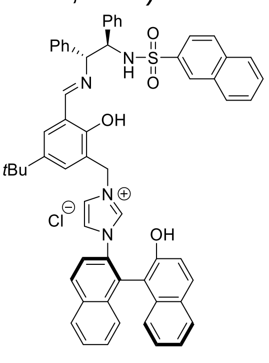

L1d-(*R*,*R*)

$C_{59}H_{51}ClN_4O_4S$, *M*: 947.59 g/mol. **m.p**. = 195 °C. **[α]$^{20}_{D}$** (c = 1.00 mg/mL, DCM): 0.90. **^{1}H NMR (400 MHz, $CDCl_3$):** *δ* (ppm) = 14.93-13.58 (*br*, 1H, ArO*H*), 10.81-10.06 (*br*, 1H, ArO*H*), 9.85 (*s*, 1H, *H*C=N), 9.10 (*br. s*, 1H, SO_2N*H*), 8.51 (*s*, 1H, Ar*H*), 7.99 (*s*, 1H, Ar*H*), 7.85-7.40 (*m*, 12H, Ar*H*), 7.33 (*t*, *J* = 7.2 Hz, 1H, Ar*H*), 7.22-6.88 (*m*, 14H, Ar*H*), 6.85 (*d*, *J* = 8.3 Hz, 1H, Ar*H*), 6.69 (*s*, 1H, Ar*H*), 6.53 (*m*, 3H, N-C*H*, Ar*H*), 5.57-5.14 (*m*, 3H, SO_2NHC*H*Ph*,* $N_{imidazole}CH_2$), 5.05 (*t*, *J* = 9.1 Hz, 1H, Ar*H,* C=NC*H*Ph), 1.26 (*s*, 9H, C(CH_3)$_3$). **^{13}C NMR (176 MHz, $CDCl_3$):** *δ* (ppm) = 153.7, 141.7, 140.0, 138.4, 137.9, 137.2, 134.1, 133.9, 133.7, 133.4, 133.3, 132.7, 132.0, 131.8, 130.7, 129.8, 129.3, 129.1, 129.0, 128.6, 128.5, 128.4, 128.3, 128.3, 128.1, 127.9, 127.8, 127.7, 127.6, 127.6, 127.5, 127.4, 127.2, 126.8, 126.7, 126.1, 123.6, 123.2, 123.1, 122.3, 122.2, 122.1, 121.2, 120.1, 118.1, 113.9, 73.6, 64.5, 62.8, 48.0, 34.4, 31.6. **IR (solid):** $\tilde{\nu}$ = 3057, 2959, 1626, 1599, 1545, 1506, 1478, 1454, 1433, 1343, 1321, 1273, 1201, 1153, 1130, 1096, 1074, 1028, 955, 908, 858, 815, 747, 728, 698, 661, 643, 616, 562, 545, 481 cm^{-1}. **HRMS (ESI)**: m/z calculated for [$C_{59}H_{51}N_4O_4S$]$^+$: 911.3626, found: 911.3628.

3-(5-(*tert*-Butyl)-2-hydroxy-3-((*E*)-(((1*S*,2*S*)-2-(2-nitrobenzenesulfonylamido)-1,2-diphenylethyl)imino)methyl)benzyl)-1-((*R*)-2'-hydroxy-[1,1'-binaphthalen]-2-yl)-1*H*-imidazol-3-ium-chloride (L1e-(*S*,*S*))

Ligand **L1e-(*S*,*S*)** was synthesized according to **GP1** using aldehyde **K2** (30.0 mg, 0.053 mmol, 1.0 equiv.) and *N*-((1*S*,2*S*)-2-Amino-1,2-diphenylethyl)-2-nitrophenylsulfonamide (21.17 mg, 0.053 mmol, 1.0 equiv.). The product was isolated as a yellow solid (43.4 mg, 0.046 mmol, 87%).

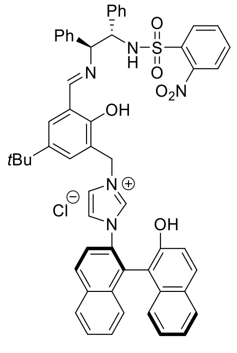

L1e-(*S*,*S*)

$C_{55}H_{48}ClN_5O_6S$, *M*: 942.52 g/mol. **m.p**. = 246 °C. **[α]$^{20}_{D}$** (c = 1.00 mg/mL, DCM): 21.6. **^{1}H NMR (400 MHz, $CDCl_3$):** *δ* (ppm) =13.69-13.62 (*br*, 1H, ArO*H*), 10.41-9.85 (*br*, 1H, ArO*H*), 9.56 (*s*, 1H, *H*C=N), 8.59 (*s*, 1H, N*H*), 7.96 (*d*, *J* = 8.5 Hz, 1H, Ar-*H*), 7.91 (*d*, *J* = 8.3 Hz, 1H, Ar*H*), 7.82-7.70 (*m*, 3H, Ar*H*), 7.69-7.45 (*m*, 5H, Ar*H*), 7.44-7.32 (*m*, 2H, Ar*H*), 7.25-7.06 (*m*, 12H, Ar*H*), 7.06-6.95 (*m*, 4H, Ar*H*), 6.84 (*d*, *J* = 8.6 Hz, 1H, NC*H*), 6.79 (*t*, *J* = 7.7 Hz, 1H, NC*H*), 6.68 (*s*, 1H, NC*H*), 5.57 (*d*, *J* = 14.4 Hz, 1H, $N_{imidazole}CH_2$), 5.30 (*d*, *J* = 14.4 Hz, 1H, $N_{imidazole}CH_2$), 5.02 (*t*, *J* = 8.1 Hz, 1H, NsNHC*H*), 4.85 (*t*, *J* = 8.1 Hz, 1H, C*H*N=), 1.09 (*s*, 9H, C(CH_3)$_3$). **^{13}C NMR (176 MHz, $CDCl_3$):** *δ* (ppm) = 146.9, 142.3, 139.0, 138.6, 137.0, 134.6, 134.1, 133.6, 133.2, 132.4, 132.1, 131.7, 130.8, 130.3, 130.1, 130.0, 128.6, 128.5, 128.4, 128.3, 128.1, 128.0, 127.9, 127.7, 127.0, 123.9, 123.6, 123.2, 122.8, 122.5, 122.1, 120.5, 120.3, 117.8, 113.8, 64.7, 48.7, 34.1, 31.3. **IR (solid):** $\tilde{\nu}$ = 3056, 2956, 2866, 1625, 1599, 1537, 1509, 1478, 1454, 1432, 1342, 1273, 1226, 1162, 1096, 1063, 1027, 907, 816, 726, 697, 641, 581, 559, 528 cm^{-1}. **HRMS (ESI)**: m/z calculated for [$C_{55}H_{48}N_5O_6S$]$^+$: 906.3320, found: 906.3326.

3-(5-(*tert*-Butyl)-2-hydroxy-3-((*E*)-(((1*R*,2*R*)-2-(2-nitrobenzenesulfonylamido)-1,2-diphenylethyl)imino)methyl)benzyl)-1-((*R*)-2'-hydroxy-[1,1'-binaphthalen]-2-yl)-1*H*-imidazol-3-ium-chloride (L1e-(*R*,*R*))

Ligand **L1e-(*R*,*R*)** was synthesized according to **GP1** using aldehyde **K2** (30.0 mg, 0.053 mmol, 1.0 equiv.) and *N*-((1*R*,2*R*)-2-Amino-1,2-diphenylethyl)-2-nitrophenylsulfonamide (21.17 mg, 0.053 mmol, 1.0 equiv.). The product was isolated as a yellow solid (44.4 mg, 0.047 mmol, 89%).

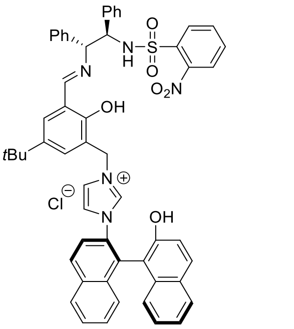

L1e-(*R*,*R*)

$C_{55}H_{48}ClN_5O_6S$, *M*: 942.52 g/mol. **m.p.** = 245 °C. **$[\alpha]^{20}_D$** (c = 1.00 mg/mL, DCM): 37.2. **^{1}H NMR (400 MHz, $CDCl_3$):** δ (ppm) = 13.69-13.62 (*br*, 1H, ArO*H*), 10.27-9.99 (*br*, 1H, ArO*H*), 9.47 (*br s*, 1H, *H*C=N), 8.32 (*br*. *s*, 1H, N*H*), 7.96 (*d*, *J* = 8.5 Hz, 1H, Ar*H*), 7.97 (*m*, 2H, Ar*H*), 7.89-7.79 (*m*, 2H, Ar*H*), 7.76-7.60 (*m*, 4H, Ar*H*), 7.58-7.49 (*m*, 2H, Ar*H*), 7.44-7.20 (*m*, 6H, Ar*H*), 7.19-7.01 (*m*, 11H, Ar*H*), 6.96-6.86 (*m*, 3H, Ar*H*, NC*H*), 6.79 (*m*, *J* = 7.7 Hz, 2H, NC*H*), 5.59-5.36 (*m*, 2H, $N_{imidazole}CH_2$), 5.01 (*s,* 2H, NsNHC*H*, C*H*N=), 1.23 (*s*, 9H, $C(CH_3)_3$). **^{13}C NMR (176 MHz, $CDCl_3$):** δ (ppm) = 138.9, 137.6, 136.9, 134.4, 134.1, 133.5, 133.2, 132.9, 132.6, 132.5, 132.3, 131.7, 130.7, 130.4, 130.0, 129.8, 128.6, 128.4, 128.4, 128.3, 128.2, 128.1, 128.0, 127.9, 127.8, 127.6, 126.8, 124.0, 123.5, 123.1, 122.8, 122.6, 122.2, 120.8, 120.3, 120.2, 118.0, 113.7, 75.8, 64.8, 48.6, 34.3, 31.5. **IR (solid):** $\tilde{\nu}$ = 3140, 3031, 2960, 2867, 1626, 1599, 1538, 1478, 1454, 1433, 1344, 1274, 1226, 1202, 1165, 1097, 1064, 1027, 909, 839, 817, 729, 699, 653, 627, 561, 531 cm^{-1}. **HRMS (ESI)**: m/z calculated for $[C_{55}H_{48}N_5O_6S]^+$: 906.3320, found: 906.3317.

3-(5-(*tert*-Butyl)-2-hydroxy-3-((*E*)-(((1*S*,2*S*)-2-(4-nitrobenzenesulfonylamido)-1,2-diphenylethyl)imino)methyl)benzyl)-1-((*R*)-2'-hydroxy-[1,1'-binaphthalen]-2-yl)-1*H*-imidazol-3-ium-chloride (L1f-(*S*,*S*))

Ligand **L1f-(*S*,*S*)** was synthesized according to **GP1** using aldehyde **K2** (30.0 mg, 0.053 mmol, 1.0 equiv.) and *N*-((1*S*,2*S*)-2-Amino-1,2-diphenylethyl)-4-nitrophenylsulfonamide (21.17 mg, 0.053 mmol, 1.0 equiv.). The product was isolated as a yellow solid (44.9 mg, 0.048 mmol, 90%).

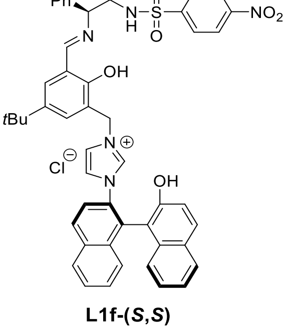

L1f-(*S*,*S*)

$C_{55}H_{48}ClN_5O_6S$, *M*: 942.52 g/mol. **m.p.** = 245 °C. **$[\alpha]^{20}_D$** (c = 1.00 mg/mL, DCM): 50.1. **^{1}H NMR (400 MHz, CD_3CN):** δ (ppm) = 13.95-12.35 (*br*, 1H, ArO*H*), 10.08-8.85 (*br*, 1H, ArO*H*), 9.56 (*s*, 1H, *H*C=N), 8.71 (*s*, 1H, N*H*), 8.61 (*s*, 1H, O*H*), 8.18 (*d*, *J* = 9.0 Hz, 1H, Ar*H*), 8.06 (*d*, *J* = 8.6 Hz, 1H, Ar*H*), 7.83-7.75 (*m*, 4H, Ar*H*), 7.67 (*d*, *J* = 8.9 Hz, 1H, Ar*H*), 7.60 (*t*, *J* = 7.2 Hz, 1H, Ar*H*), 7.52 (*d*, *J* = 8.1 Hz, 1H, Ar*H*), 7.42 (*s*, 2H, Ar*H*), 7.37 (*t*, *J* = 8.3 Hz, 1H, Ar*H*), 7.34-6.95 (*m*, 18H, Ar*H,* NC*H*), 6.74 (*d*, *J* = 8.5 Hz, 1H, NC*H*), 5.18 (*d*, *J* = 13.9 Hz, 1H, $N_{imidazole}CH_2$), 5.09-4.95 (*m*, 3H, $N_{imidazole}CH_2$, *p*-NsNHC*H,* C*H*N=), 1.24 (*s*, 9H, $C(CH_3)_3$). **^{13}C NMR (176 MHz, CD_2Cl_2):** δ (ppm) = 167.8, 157.8, 154.0, 148.9, 147.9, 142.1, 139.5, 139.1, 136.8, 134.5, 133.9, 133.3, 132.6, 132.1, 130.9, 130.3, 128.8, 128.8, 128.7, 128.6, 128.5, 128.4, 128.1, 127.9, 127.7, 127.5, 127.4, 123.7, 123.6, 123.2, 122.9, 122.2, 120.1, 119.8, 118.6, 113.5, 76.24, 64.95, 49.43, 34.25, 31.40. **IR (solid):** $\tilde{\nu}$ = 3056, 2860, 1625, 1600, 1525, 1477, 1454, 1432, 1345, 1309, 1273, 1201, 1160, 1093, 1049, 1027, 952, 937, 853, 816, 774, 747, 735, 698, 684, 645, 625, 610, 557, 464 cm^{-1}. **HRMS (ESI)**: m/z calculated for $[C_{55}H_{48}N_5O_6S]^+$: 906.3320, found: 906.3319.

3-(5-(*tert*-Butyl)-2-hydroxy-3-((*E*)-(((1*R*,2*R*)-2-(2-nitrobenzenesulfonylamido)-1,2-diphenylethyl)imino)methyl)benzyl)-1-((*R*)-2'-hydroxy-[1,1'-binaphthalen]-2-yl)-1*H*-imidazol-3-ium-chloride (L1f-(*R*,*R*))

Ligand **L1f-(*R*,*R*)** was synthesized according to **GP1** using aldehyde **K2** (30.0 mg, 0.053 mmol, 1.0 equiv.) and *N*-((1*R*,2*R*)-2-Amino-1,2-diphenylethyl)-4-nitrophenylsulfonamide (21.17 mg, 0.053 mmol, 1.0 equiv.). The product was isolated as a yellow solid (42.3 mg, 0.045 mmol, 85%).

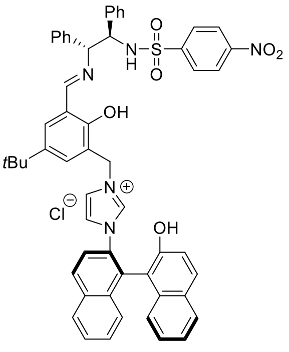

L1f-(*R*,*R*)

$C_{55}H_{48}ClN_5O_6S$, *M*: 942.52 g/mol. **m.p.** = 242 °C. **$[\alpha]^{20}_D$** (c = 1.00 mg/mL, DCM): 44.1. **^{1}H NMR (400 MHz, $CDCl_3$):** δ (ppm) = 14.75-13.70 (*br*, 1H, ArO*H*), 10.65-9.91 (*br*, 1H, ArO*H*), 9.73 (*s*, 1H, *H*C=N), 9.60 (*br. s*, 1H, N*H*), 8.46 (*s*, 1H, O*H*), 7.76 (*t*, *J* = 8.2 Hz, 2H, Ar*H*), 7.66-7.49 (*m*, 5H, Ar*H*), 7.48-7.40 (*m*, 2H, Ar*H*), 7.37 (*d*, *J* = 8.6 Hz, 1H, Ar*H*), 7.28-6.77 (*m*, 16H, Ar*H*), 6.76-6.57 (*m*, 4H, Ar*H*, NC*H*), 5.39-5.13 (*m*, 3H, $N_{imidazole}CH_2$, *p*-NsNHC*H,*), 5.11-4.99 (*m*, 1H, C*H*N=), 1.16 (*s*, 9H, $C(CH_3)_3$). **^{13}C NMR (100 MHz, $CDCl_3$):** δ (ppm) = 167.7, 157.5, 153.3, 148.5, 147.3, 141.6, 139.9, 138.3, 137.5, 134.1, 133.7, 133.2, 132.9, 132.4, 131.8, 130.8, 129.5, 128.7, 128.6, 128.5, 128.4, 128.3, 128.2, 127.8, 127.6, 127.2, 127.0, 123.6, 123.2, 122.7, 122.1, 121.6, 120.5, 120.0, 118.5, 114.1, 73.7, 64.7, 48.2, 34.2, 31.6. **IR (solid):** $\tilde{\nu}$ = 3064, 2958, 2925, 2859, 1736, 1627, 1604, 1528, 1455, 1347, 1310, 1276, 1163, 1093, 1053, 1028, 937, 910, 854, 818, 736, 700, 685, 610, 557, 464 cm^{-1}. **HRMS (ESI)**: m/z calculated for $[C_{55}H_{48}N_5O_6S]^+$: 906.3320, found: 906.3318.

3-(5-(*tert*-Butyl)-3-((*E*)-(((1*S*,2*S*)-1,2-diphenyl-2-(methylsulfonamido)ethyl)imino)-methyl)-2-hydroxybenzyl)-1-((*R*)-2'-hydroxy-[1,1'-binaphthalen]-2-yl)-1H-imidazol-3-iumchlorid (L1g-(*S*,*S*))

Ligand **L1g-(*S*,*S*)** was synthesized according to **GP1** using aldehyde **K2** (17.82 mg, 0.032 mmol, 1.0 equiv.) and *N*-((1*S*,2*S*)-2-Amino-1,2-diphenylethyl)-methanesulfonamide (9.19 mg, 0.032 mmol, 1.0 equiv.). The product was isolated as a yellow solid (26.3 mg, 0.0315 mmol, 99%).

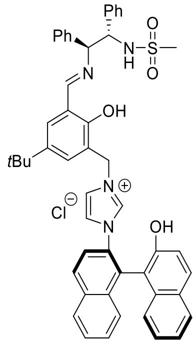

L1g-(*S*,*S*)

$C_{50}H_{47}ClN_4O_4S$. ***M*:** 835.46 g/mol. **m.p.** = 189 °C. $[\alpha]_D^{20}$ (c = 1.00 mg/mL, DCM): 28.0. **^{1}H NMR (400 MHz, $CDCl_3$)**: δ = 13.61 (*br*, 1H, ArO*H*), 9.57 (*br*, 1H, ArO*H*), 9.50 (*s*, 1H, C*H*N), 8.28 (*s*, 1H, NC*H*N), 8.08 (*d*, 1H, *J* = 8.8 Hz, Ar*H*), 7.98 (*d*, 1H, *J* = 8.2 Hz, Ar*H*), 7.68-7.51 (*m*, 4H, Ar*H*), 7.93-7.90 (*m*, 1H, Ar*H*), 7.76-7.64 (*m*, 2H, Ar*H*), 7.63-7.54 (*m*, 2H, Ar*H*), 7.45-7.37 (*m*, 2H, Ar*H*), 7.34-7.28 (*m*, 2H, Ar*H*), 7.25-7.12 (*m*, 9H, Ar*H*, N*H*CCHN), 7.07 (*s*, 1H, Ar*H*), 6.07 (*m*, 1H, NHCC*H*N), 6.71 (*s*, 1H, Ar*H*), 5.73 (*d*, 1H, *J* = 13.7 Hz, ArCH*H*), 5.44 (*d*, 1H, *J* = 13.9 Hz, ArCH*H*), 5.02 (*m*, 2H, CNC*H*Ph, PhC*H*NHSO$_2$Me), 2.40 (*s*, 3H, SO$_2$C*H$_3$*), 1.23 (*s*, 9H, C(C*H$_3$*)$_3$). **^{13}C NMR (100 MHz, $CDCl_3$)**: δ = 167.3, 157.4, 153.3, 141.7, 139.3, 138.7, 136.7, 134.0, 133.5, 133.1, 132.6, 132.1, 131.8, 130.7, 130.0, 129.6, 128.7, 128.6, 128.5, 128.5, 128.4, 128.3, 128.3, 127.9, 127.9, 127.8, 127.7, 127.6, 127.0, 123.5, 123.2, 122.7, 122.3, 122.1, 120.8,119.6, 118.0, 113.3, 75.1, 64.3, 48.7, 41.6, 34.1, 31.4. **IR (solid)**: $\tilde{\nu}$ = 3061, 2960, 1627, 1600, 1546, 1508, 1479, 1455, 1433, 1344, 1319, 1275, 1204, 1150, 1098, 1066, 975, 910, 819, 751, 731, 700 cm^{-1}. **HRMS (ESI)** *m/z*: calculated for $[C_{50}H_{47}N_4O_4S]^+$ 799.3313, found: 799.3312.

3-(5-(*tert*-Butyl)-3-((*E*)-(((1*R*,2*R*)-1,2-diphenyl-2-(methylsulfonamido)ethyl)imino)-methyl)-2-hydroxybenzyl)-1-((*R*)-2'-hydroxy-[1,1'-binaphthalen]-2-yl)-1H-imidazol-3-iumchlorid (L1g-(*R*,*R*))

Ligand **L1g-(*R*,*R*)** was synthesized according to **GP1** using aldehyde **K2** (20.2 mg, 0.036 mmol, 1.0 equiv.) and *N*-((1*R*,2*R*)-2-Amino-1,2-diphenylethyl)-methanesulfonamide (10.4 mg, 0.036 mmol, 1.0 equiv.). The product was isolated as a yellow solid (28.1 mg, 0.032 mmol, 86%).

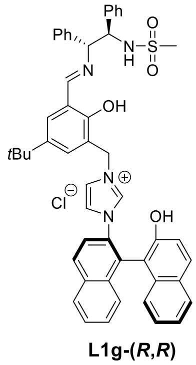

L1g-(*R*,*R*)

$C_{50}H_{47}ClN_4O_4S$. *M*: 835.46 g/mol. **m.p.** = 187 °C. $[\alpha]_D^{20}$ (c = 1.8 mg/mL, DCM): 108.4. **^{1}H NMR (400 MHz, $CDCl_3$)**: δ = 14.06 (*br*, 1H, ArO*H*), 10.14 (*br*, 1H, ArO*H*), 9.42 (*s*, 1H, C*H*N), 8.40 (*br*, 1H, N*H*SO_2CH_3), 8.30 (*s*, 1H, NC*H*N), 8.07 (*d*, 1H, *J* = 8.8 Hz, Ar*H*), 8.01-7.95 (*m*, 2H, Ar*H*), 7.68-7.51 (*m*, 5H, Ar*H*), 7.43-7.37 (*m*, 4H, Ar*H*), 7.22-7.07 (*m*, 9H, Ar*H*), 7.04-6.95 (*m*, 3H, Ar*H*, N*H*CCHN), 6.82-6.78 (*m*, 1H, NHCC*H*N), 6.70 (*s*, 1H, Ar*H*), 5.50 (*d*, 1H, *J* = 13.1 Hz, ArCH*H*), 5.35 (*d*, 1H, *J* = 13.7 Hz, ArCH*H*), 5.25 (*d*, 1H, *J* = 9.4 Hz, CNC*H*Ph), 4.92 (*t*, 1H, *J* = 9.6 Hz, PhC*H*NHSO_2Me), 2.28 (*s*, 3H, SO_2CH_3), 1.27 (*s*, 9H, C(C*H*$_3$)$_3$). **^{13}C NMR (100 MHz, $CDCl_3$)**: δ = 167.1, 157.4, 153.3, 141.4, 139.6, 138.8, 137.2, 134.1, 133.5, 133.4, 133.1, 132.7, 131.8, 130.5, 129.8, 129.0, 128.7, 128.6, 128.4, 128.3, 128.3, 127.9, 127.9, 127.7, 127.6, 127.5, 127.0, 126.7, 126.6, 123.5, 122.8, 122.8, 122.2, 122.0, 121.0, 119.8, 117.7, 113.5, 72.9, 64.3, 48.1, 41.7, 34.3, 31.5. **IR (solid)**: $\tilde{\nu}$ = 3061, 2964, 1627, 1600, 1546, 1507 ,1479, 1456, 1434, 1319, 1276, 1204, 1150, 1098, 1067, 976, 912, 818, 752, 735, 702 cm^{-1}. **HRMS (ESI)** *m/z*: calculated for $[C_{50}H_{47}N_4O_4S]^+$ 799.3313, found: 799.3303.

3-(5-(*tert*-Butyl)-3-((*E*)-(((1*S*,2*S*)-1,2-diphenyl-2-(*p*-tolylsulfonamido)ethyl)imino)-methyl)-2-hydroxybenzyl)-1-((*R*)-2'-hydroxy-[1,1'-binaphthalen]-2-yl)-1*H*-imidazol-3-iumchlorid (L1h-(*S*,*S*))

Ligand **L1h-(*S*,*S*)** was synthesized according to **GP1** using aldehyde **K2** (19.9 mg, 0.035 mmol, 1.0 equiv.) and *N*-((1*S*,2*S*)-2-Amino-1,2-diphenylethyl)-4-methylphenylsulfonamide (12.8 mg, 0.035 mmol, 1.0 equiv.). The product was isolated as a yellow solid (29.1 mg, 0.032 mmol, 91%).

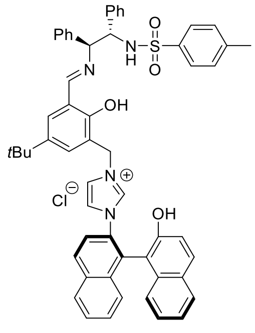

L1h-(*S*,*S*)

$C_{56}H_{51}ClN_4O_4S$. *M*: 911.56 g/mol. **m.p.**= 149 °C. $[\alpha]_D^{20}$ (c = 0.75 mg/mL, DCM): −10.0. **^{1}H NMR (400 MHz, $CDCl_3$)**: δ = 13.57 (*br*, 1H, ArO*H*), 9.99 (*br*, 1H, ArO*H*), 9.62 (*s*, 1H, C*H*N), 8.31 (*s*, 1H, NC*H*N), 8.02 (*d*, 1H, *J* = 8.7 Hz, Ar*H*), 7.97 (*d*, 1H, *J* = 8.4 Hz, Ar*H*), 7.89 (*d*, 1H, *J* = 1.9 Hz, Ar*H*), 7.77-7.68 (*m*, 3H, Ar*H*), 7.63-7.58 (*m*, 1H, Ar*H*), 7.54 (*d*, 1H, *J* = 8.7 Hz, Ar*H*), 7.47-7.32 (*m*, 4H, Ar*H*), 7.29-7.21 (*m*, 2H, Ar*H*), 7.18-7.10 (*m*, 6H, Ar*H*), 7.07-6.82 (*m*, 10H, Ar*H*, N*H*CC*H*N), 6.75-6.72 (*m*, 1H, Ar*H*), 6.30 (*br*, 1H, N*H*Tos), 5.81 (*d*, 1H, *J* = 13.8 Hz, ArCH*H*), 5.48 (*d*, 1H, *J* = 13.8 Hz, ArCH*H*), 4.82-4.76 (*m*, 2H, CNC*H*Ph, PhC*H*NTos), 2.21 (*s*, 3H, *ArC*H$_3$*)*,1.24 (*s*, 9H, C(C*H*$_3$)$_3$). **^{13}C NMR (100 MHz, $CDCl_3$)**: δ = 167.3, 157.4, 153.5, 142.2, 141.9, 139.0, 137.9, 136.9, 134.1, 133.6, 133.1, 132.5, 132.3, 131.8, 130.7, 129.9, 129.9, 129.1, 128.8, 128.4, 128.3, 128.3, 128.2, 128.1, 128.0, 127.9, 127.7, 127.6, 127.4, 127.3, 127.1, 126.9, 126.6, 126.6, 123.5, 123.1, 122.7, 122.2, 120.7, 120.0,118.0, 113.5, 76.0, 64.2, 48.7, 34.1, 31.4, 21.3. **IR (solid)**: $\tilde{\nu}$ = 3060, 2960, 2868, 1626, 1599, 1546, 1508, 1479, 1455, 1433, 1364, 1344, 1324, 1275, 1204, 1156, 1094, 1066, 1028, 909, 816, 750, 729, 699 cm^{-1}. **HRMS (ESI)** *m/z*: calculated for $[C_{56}H_{51}N_4O_4S]^+$ 875.3626, found: 875.3622.

3-(5-(*tert*-Butyl)-3-((*E*)-(((1*R*,2*R*)-1,2-diphenyl-2-(*p*-tolylsulfonamido)ethyl)imino)-methyl)-2-hydroxybenzyl)-1-((*R*)-2'-hydroxy-[1,1'-binaphthalen]-2-yl)-1*H*-imidazol-3-iumchlorid (L1h-(*R*,*R*))

Ligand **L1h-(*R*,*R*)** was synthesized according to **GP1** using aldehyde **K2** (16.4 mg, 0.029 mmol, 1.0 equiv.) and *N*-((1*R*,2*R*)-2-Amino-1,2-diphenylethyl)-4-methylphenylsulfonamide (10.6 mg, 0.029 mmol, 1.0 equiv.). The product was isolated as a yellow solid (24.8 mg, 0.027 mmol, 94%).

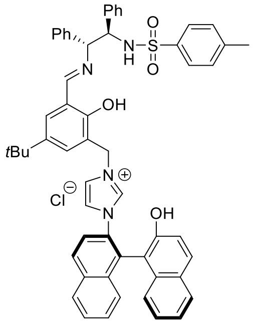

L1h-(*R*,*R*)

$C_{56}H_{51}ClN_4O_4S$. ***M*:** 911.56 g/mol. **m.p.** = 152 °C. $[\alpha]_D^{20}$ (c = 1.7 mg/mL, DCM): 113.5. **^{1}H NMR (400 MHz, $CDCl_3$)**: *δ* = 14.25 (*br*, 1H, ArO*H*), 10.38 (*br*, 1H, ArO*H*), 8.98 (*br*, 1H, N*H*Tos), 8.21 (*s*, 1H, C*H*N), 8.38 (*s*, 1H, NC*H*N), 8.00 (*s*, 1H, Ar*H*), 7.88-7.84 (*m*, 2H, Ar*H*), 7.72-7.60 (*m*, 3H, Ar*H*), 7.57-7.52 (*m*, 1H, Ar*H*), 7.46-7.38 (*m*, 3H, Ar*H*), 7.35-7.30 (*m*, 1H, Ar*H*), 7.20-6.94 (*m*, 12H, Ar*H*), 6.83-6.72 (*m*, 4H, Ar*H*), 6.63 (*m*, 1H, Ar*H*), 6.41-6.37 (*m*, 2H, N*H*CC*H*N), 5.47 (*d*, 1H, *J* = 13.6 Hz, ArCH*H*), 5.34 (*d*, 1H, *J* = 13.6 Hz, ArCH*H*), 5.26 (*d*, 1H, *J* = 10.4 Hz, CNC*H*Ph), 4.92 (*t*, 1H, *J* = 9.9 Hz, PhC*H*NTos), 2.00 (*s*, 3H, ArC*H*$_3$), 1.26 (*s*, 9H, C(C*H*$_3$)$_3$). **^{13}C NMR (100 MHz, $CDCl_3$)**: *δ* = 167.1, 157.6, 153.6, 141.5, 141.3, 139.8, 138.3, 137.6, 137.4, 134.0, 133.6, 133.3, 133.1, 132.5, 131.8, 130.5, 129.8, 129.1, 128.6, 128.4, 128.3, 128.3, 128.2, 128.2, 127.7, 127.6, 127.5, 127.4, 127.3, 127.1, 126.9, 126.6, 126.5, 123.4, 123.0, 122.9, 122.2, 121.9, 121.0, 120.0, 117.9, 113.8, 73.4, 64.3, 48.0, 34.2, 31.5, 21.1. **IR (solid)**: $\tilde{\nu}$ =3061, 2956, 2925, 1626, 1599, 1545, 1496, 1456, 1327, 1276, 1207, 1158, 1094, 816, 751, 700 cm^{-1}. **HRMS (ESI)** *m/z*: calculated for $[C_{56}H_{51}N_4O_4S]^+$ 875.3626, found: 875.3628.

3-(5-(*tert*-Butyl)-3-((*E*)-(((1*S*,2*S*)-1,2-diphenyl-2-(pentafluorobenzylsulfonamido)ethyl)-imino)-methyl)-2-hydroxybenzyl)-1-((*R*)-2'-hydroxy-[1,1'-binaphthalen]-2-yl)-1H-imidazol-3-iumchlorid (L1i-(*S*,*S*))

Ligand **L1i-(*S*,*S*)** was synthesized according to **GP1** using aldehyde **K2** (26.5 mg, 0.047 mmol, 1.0 equiv.) and *N*-((1*S*,2*S*)-2-Amino-1,2-diphenylethyl)-perfluorophenylsulfonamide (20.8 mg, 0.047 mmol, 1.0 equiv.). The product was isolated as a yellow solid (32.4 mg, 0.033 mmol, 70%).

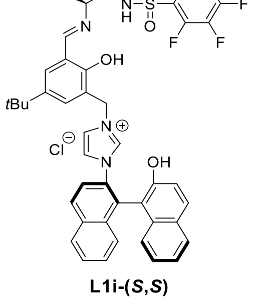

L1i-(*S*,*S*)

$C_{55}H_{44}ClF_5N_4O_4S$. ***M*:** 987.48 g/mol. **m.p.** = 216 °C. $[\alpha]_D^{20}$ (c = 1.00 mg/mL, DCM): 48.4. **^{1}H NMR (300 MHz, $CDCl_3$)**: δ = 13.83 (*br*, 1H, ArO*H*), 9.34 (*br*, 1H, ArO*H*), 9.07 (*s*, 1H, C*H*N), 8.62 (*s*, 1H, NC*H*N), 8.00 (*d*, 1H, *J* = 8.7 Hz, Ar*H*), 7.90 (*d*, 1H, *J* = 8.3 Hz, Ar*H*), 7.73-7.49 (*m*, 5H, Ar*H*), 7.40-7.03 (*m*, 14H, Ar*H*), 7.00-6.92 (*m*, 3H, Ar*H*), 6.83 (*m*, 1H, NHCC*H*N), 6.65 (*s*, 1H, N*H*CCHN), 5.45 (*d*, 1H, *J* = 13.9 Hz, ArCH*H*), 5.35 (*d*, 1H, *J* = 9.7 Hz, CNC*H*Ar), 5.11 (*d*, 1H, *J* = 13.9 Hz, ArCH*H*), 4.99 (*d*, 1H, *J* = 9.7 Hz, PhC*H*NHSO$_2$Ar), 1.10 (*s*, 9H, C(C*H$_3$*)$_3$). **^{19}F NMR (376 MHz, $CDCl_3$)**: δ = –134.9 (*m*), –148.2 (*m*), –160.2 (*m*). **^{13}C NMR (100 MHz, $CDCl_3$)**: δ = 167.5, 157.6, 153.4, 143.1 (*dd*, *J* = 12.3, 255.4 Hz, 2C, CF), 142.9 (*dt*, *J* = 13.1, 260.4 Hz, 1C, CF), 141.7, 138.7, 137.1 (*dt*, *J* = 17.2, 255.0 Hz, 2C, CF), 136.9, 136.1, 134.0, 133.5, 133.0, 132.1, 132.0, 131.7, 130.8, 130.0, 128.5, 128.4, 128.3, 128.3, 128.2, 128.2, 128.1, 128.0, 128.0, 127.9, 127.8, 127.7, 127.6, 127.5, 126.9, 123.4, 123.2, 122.5, 122.4, 122.0, 120.3, 119.6, 118.1, 117.2 (*t*, *J* = 14.3 Hz, 1C, ArCSO$_2$), 113.0, 74.3, 64.8, 48.8, 34.0, 31.3. **IR (solid)**: $\tilde{\nu}$ = 3054, 2961, 1627, 1601, 1519, 1497, 1455, 1434, 1363, 1346, 1296, 1275, 1171, 1098, 991, 818, 749, 700, 645, 627, 604 cm^{-1}. **HRMS (ESI)** *m/z*: calculated for $[C_{55}H_{44}F_5N_4O_4S]^+$ 951.2998, found: 951.3006.

3-(5-(*tert*-Butyl)-3-((*E*)-(((1*R*,2*R*)-1,2-diphenyl-2-(pentafluorobenzylsulfonamido)ethyl)-imino)-methyl)-2-hydroxybenzyl)-1-((*R*)-2'-hydroxy-[1,1'-binaphthalen]-2-yl)-1H-imidazol-3-iumchlorid (L1i-(*R*,*R*))

Ligand **L1i-(*R*,*R*)** was synthesized according to **GP1** using aldehyde **K2** (29.1 mg, 0.046 mmol, 1.0 equiv.) and *N*-((1*R*,2*R*)-2-Amino-1,2-diphenylethyl)-perfluorophenylsulfonamide (20.5 mg, 0.046 mmol, 1.0 equiv.) The product was isolated as a yellow solid (25.1 mg, 0.025 mmol, 55%).

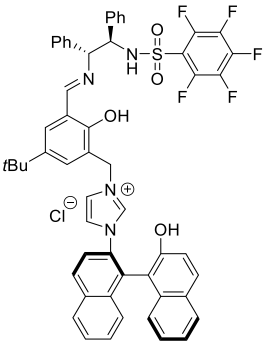

L1i-(*R*,*R*)

$C_{55}H_{44}ClF_5N_4O_4S$. ***M*:** 987.48 g/mol. **m.p.** = 216 °C. $[\alpha]_D^{20}$ (c = 1.6 mg/mL, DCM): 123.0. **^{1}H NMR (400 MHz, $CDCl_3$)**: δ = 14.25 (*br*, 1H, ArO*H*), 10.20-9.80 (*br*, 2H, ArO*H*, N*H*SO$_2$Ar), 9.53 (*s*, 1H, C*H*N), 8.21 (*s*, 1H, NC*H*N), 8.06-7.94 (*m*, 2H, Ar*H*), 7.71-7.47 (*m*, 5H, Ar*H*), 7.43-7.33 (*m*, 3H, Ar*H*), 7.24-6.97 (*m*, 10H, Ar*H*), 6.95-6.78 (*m*, 5H, Ar*H*, N*H*CC*H*N), 6.75 (*s*, 1H, Ar*H*), 5.71 (*d*, 1H, *J* = 13.9 Hz, ArCH*H*), 5.58 (*d*, 1H, *J* = 10.4 Hz, CNC*H*Ar), 5.46 (*d*, 1H, *J* = 13.3 Hz, ArCH*H*), 4.97 (*d*, 1H, *J* = 10.2 Hz, PhC*H*NHSO$_2$Ar), 1.20 (*s*, 9H, C(C*H$_3$*)$_3$). **^{19}F NMR (376 MHz, $CDCl_3$)**: δ = –134.6 (*m*), –148.8 (*m*), –160.5 (*m*). **^{13}C NMR (176 MHz, $CDCl_3$)**: δ = 164.9, 155.1, 150.9, 141.5 (*d*, *J* = 257.0 Hz, 2C, CF), 140.1 (*dt*, *J* = 14.3, 261.8 Hz, 1C, CF), 139.2, 136.2, 134.6 (*dt*, *J* = 13.4, 255.6 Hz, 2C, CF), 134.3, 133.6, 131.5, 130.9, 130.5, 129.6, 129.5, 129.1, 128.3, 127.5, 127.4, 126.0, 125.9, 125.8, 125.7, 125.5, 125.4, 125.3, 125.2, 125.1, 125.0, 124.4, 120.8, 120.6, 120.0, 119.9, 119.5, 117.8, 117.1, 115.6, 114.6 (*t*, *J* = 14.0 Hz, Ar*C*SO$_2$), 110.5, 71.7, 62.3, 46.2, 31.5, 28.7. **IR (solid)**: $\tilde{\nu}$ = 3065, 2960, 1627, 1600, 1518, 1497, 1455, 1434, 1364, 1346, 1296, 1275, 1171, 1098, 991, 817, 750, 699, 645, 603 cm^{-1}. **HRMS (ESI)** *m/z*: calculated for $[C_{55}H_{44}F_5N_4O_4S]^+$ 951.2998, found: 951.3023.

Synthesis of the Complexes

3-(5-(*tert*-Butyl)-3-((*E*)-(((1*S*,2*S*)-1,2-diphenyl-2-((1-naphthalene)sulfonamido)ethyl)imino)methyl)-2-hydroxybenzyl)-1-(2'-hydroxy-[1,1'-binaphthalen]-2-yl)-1*H*-imidazol-3-ium copper (II)-chloride (C1b-(*S*,*S*))

The synthesis was carried out according to **GP2** using ligand **L1b-(*S*,*S*)** (100.0 mg, 0.105 mmol, 1.0 equiv.) and Cu(acac)$_2$ (27.62 mg, 0.105 mmol, 1.0 equiv.). The pre-catalyst **C1b-(*S*,*S*)** was obtained as a pale green solid (104.6 mg, 0.102 mmol, 97%).

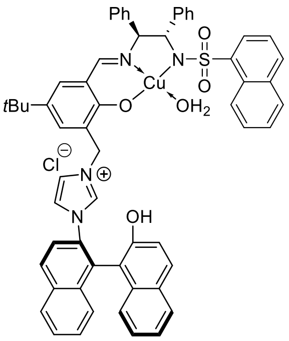

C1b-(*S*,*S*)

$C_{59}H_{51}ClCuN_4O_5S$, ***M***: 1027.12 g/mol. **m.p.** = 180 °C. **[α]$^{20}_D$** (c = 1.00 mg/mL, DCM): –49.0. **^{1}H NMR:** paramagnetic species. **IR (solid):** $\tilde{\nu}$ = 3057, 2961, 2155, 1625, 1542, 1434, 1344, 1274, 1117, 730 cm^{-1}. **HRMS (ESI)** m/z: calculated for [$C_{59}H_{49}CuN_4O_4S$]$^+$: 972.2765. Found: 972.2742. **Elemental Analysis** calculated for [$C_{59}H_{49}ClCuN_4O_4S$ x $2H_2O$]: C 67.80, H 5.11, N 5.36, found: C 67.62, H 5.44, N 5.05.

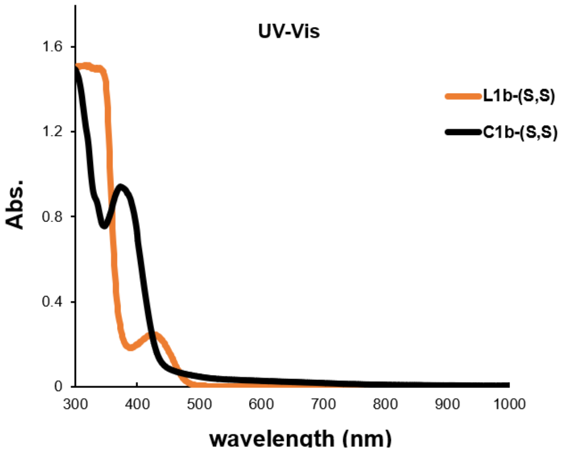

3-(5-(*tert*-Butyl)-3-((*E*)-(((1*R*,2*R*)-1,2-diphenyl-2-((1-naphthalene)sulfonamido)ethyl)imino)methyl)-2-hydroxybenzyl)-1-(2'-hydroxy-[1,1'-binaphthalen]-2-yl)-1*H*-imidazol-3-ium copper (II)-chloride (C1b-(*R*,*R*))

The synthesis was carried out according to **GP2** using ligand **L1b-(*R*,*R*)** (20.0 mg, 0.021 mmol, 1.0 equiv.) and Cu(acac)$_2$ (5.52 mg, 0.021 mmol, 1.0 equiv.). The pre-catalyst **C1b-(*R*,*R*)** was obtained as a pale green solid (19.8 mg, 0.019 mmol, 92%).

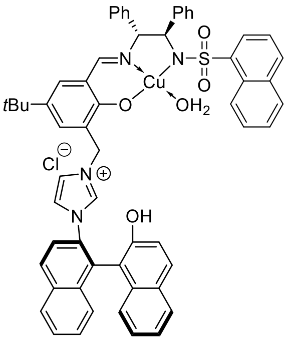

C1b-(*R*,*R*)

$C_{59}H_{51}ClCuN_4O_5S$, ***M***: 1027.12 g/mol. **m.p.** = 175 °C. **[α]$^{20}_{D}$** (c = 1.00 mg/mL, DCM): 91.6. **^{1}H NMR:** paramagnetic species. **IR (solid):** $\tilde{\nu}$ = 3060, 2956, 2668, 1625, 1543, 1452, 1434, 1274, 1118, 802, 769, 730 cm^{-1}. **HRMS (ESI)** m/z: calculated for $[C_{59}H_{49}CuN_4O_4S]^+$: 972.2765. Found: 972.2760.

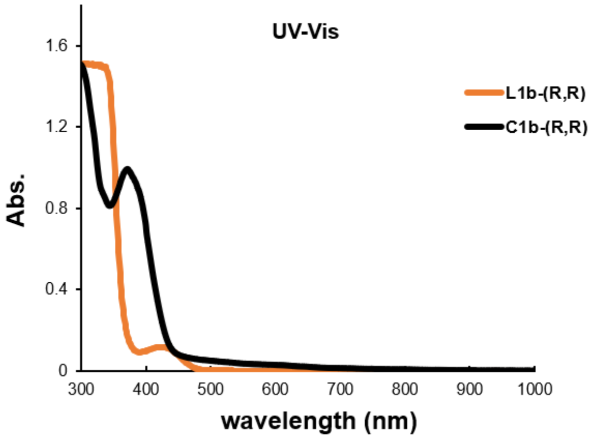

3-(5-(*tert*-Butyl)-3-((*E*)-(((1*S*,2*S*)-1,2-diphenyl-2-((2-(anthrachene-9-sulfonamido)ethyl)imino)methyl)-2-hydroxybenzyl)-1-(2'-hydroxy-[1,1'-binaphthalen]-2-yl)-1*H*-imidazol-3-ium copper (II)-chloride (C1c-(*S*,*S*))

The synthesis was carried out according to **GP2**. The ligand **L1c-(*S*,*S*)** (18.7 mg, 0.018 mmol, 1.0 equiv.) and $Cu(acac)_2$ (4.91 mg, 0.018 mmol, 1.0 equiv.). After work-up the pre-catalyst **C1c-(*S*,*S*)** (18.4 mg, 0.017 mmol, 95%) was obtained as light green solid.

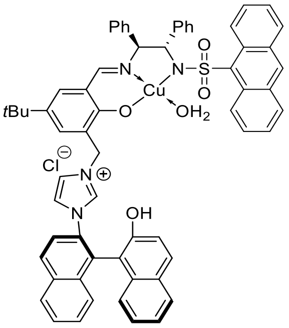

C1c-(*S*,*S*)

$C_{63}H_{53}ClCuN_4O_5S$, ***M***: 1077.18 g/mol. **m.p.** = 216 °C. **[α]**$^{20}_{D}$ (c = 1.00 mg/mL, DCM): –36.5. **^{1}H NMR:** paramagnetic species. **^{13}C NMR:** paramagnetic species. **IR (solid):** $\tilde{\nu}$ = 3135, 3058, 2957, 2924, 2854, 1626, 1543, 1494, 1449, 1393, 1364, 1346, 1275, 1221, 1126, 1098, 1026, 994,950, 909, 817, 780, 734, 699, 646, 587 cm^{-1}. **HRMS (ESI)** m/z: calculated for $[C_{63}H_{51}CuN_4O_4S]^+$: 1022.2922. Found: 1022.2920.

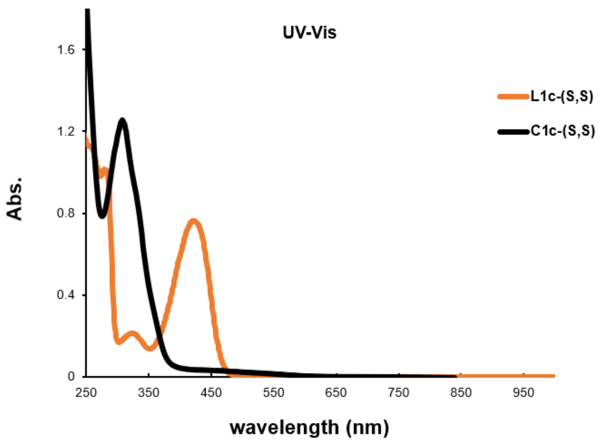

3-(5-(*tert*-Butyl)-3-((*E*)-(((1*S*,2*S*)-1,2-diphenyl-2-((2-naphthalene)sulfonamido)ethyl)imino)methyl)-2-hydroxybenzyl)-1-(2'-hydroxy-[1,1'-binaphthalen]-2-yl)-1*H*-imidazol-3-ium copper (II)-chloride (C1d-(*S*,*S*))

The synthesis was carried out according to **GP2** using the ligand **L1d-(*S*,*S*)** (30.0 mg, 0.032 mmol, 1.0 equiv.) and Cu(acac)$_2$ (8.29 mg, 0.032 mmol, 1.0 equiv.). The pre-catalyst **C1d-(*S*,*S*)** was obtained as a pale green solid (29.9 mg, 0.029 mmol, 91%).

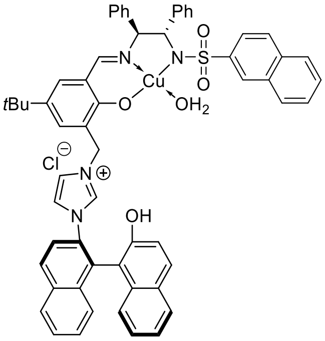

C1d-(*S*,*S*)

$C_{59}H_{51}ClCuN_4O_5S$, ***M***: 1027.12 g/mol. **m.p.** = 184 °C. **$[\alpha]^{20}_D$** (c = 1.00 mg/mL, DCM): –35.4. **^{1}H NMR:** paramagnetic species. **^{13}C NMR:** paramagnetic species. **IR (solid):** $\tilde{\nu}$ = 3057, 2957, 2925, 1626, 1543, 1451, 1435, 1393, 1365, 1345, 1274, 1221, 1122, 1097, 1079, 1027, 996, 936, 816, 748, 698, 662, 618, 550, 503, 476 cm^{-1}. **HRMS (ESI)** m/z: calculated for $[C_{59}H_{49}CuN_4O_4S]^+$: 972.2765. Found: 972.2795.

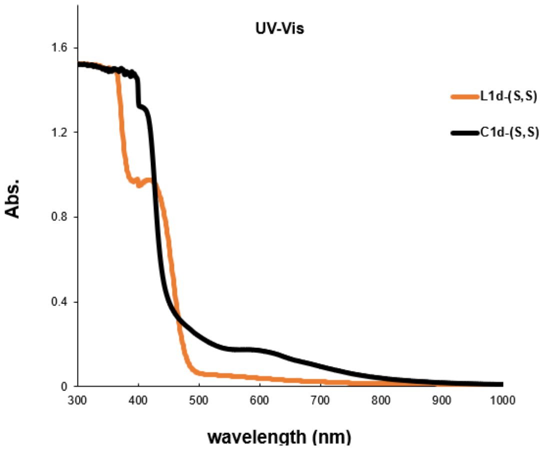

3-(5-(*tert*-Butyl)-3-((*E*)-(((1*R*,2*R*)-1,2-diphenyl-2-((2-naphthalene)sulfonamido)ethyl)imino)methyl)-2-hydroxybenzyl)-1-(2'-hydroxy-[1,1'-binaphthalen]-2-yl)-1*H*-imidazol-3-ium copper (II)-chloride (C1d-(*R*,*R*))

The synthesis was carried out according to **GP2** using ligand **L1d-(*R*,*R*)** (30.0 mg, 0.032 mmol, 1.0 equiv.) and Cu(acac)$_2$ (8.29 mg, 0.032 mmol, 1.0 equiv.). The pre-catalyst **C1d-(*R*,*R*)** was obtained as a pale green solid (31.2 mg, 0.03 mmol, 95%).

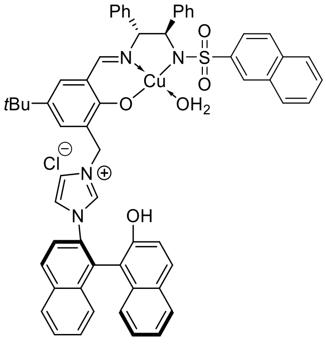

C1d-(*R*,*R*)

$C_{59}H_{51}ClCuN_4O_5S$, ***M***: 1027.12 g/mol. **m.p**. = 214 °C. **$[\alpha]^{20}_D$** (c = 1.00 mg/mL, DCM): 59.0. **^{1}H NMR:** paramagnetic species. **^{13}C NMR:** paramagnetic species. **IR (solid):** $\tilde{\nu}$ = 3055, 2950, 2920, 1627, 1542, 1450, 1437, 1392, 1366, 1346, 1271, 1221, 1123, 1095, 1071, 1028, 997, 934, 816, 749, 699, 660, 620, 555, 510, 471 cm^{-1}. **HRMS (ESI)** m/z: calculated for $[C_{59}H_{49}CuN_4O_4S]^+$: 972.2765. Found: 972.2795.

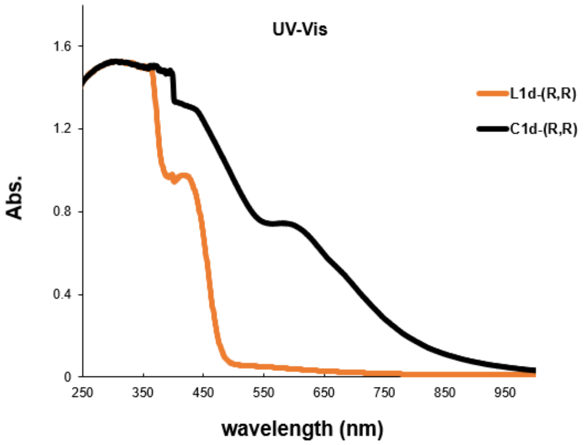

3-(5-(*tert*-Butyl)-3-((*E*)-(((1*S*,2*S*)-1,2-diphenyl-2-((2-nitrophenyl)sulfonamido)ethyl)imino)methyl)-2-hydroxybenzyl)-1-(2'-hydroxy-[1,1'-binaphthalen]-2-yl)-1*H*-imidazol-3-ium copper (II)-chloride (C1e-(*S*,*S*))

The synthesis was carried out according to **GP2** using ligand **L1e-(*S*,*S*)** (20.0 mg, 0.021 mmol, 1.0 equiv.) and Cu(acac)$_2$ (5.55 mg, 0.015 mmol, 1.0 equiv.). The pre-catalyst **C1e-(*S*,*S*)** was obtained as a pale green solid (14.9 mg, 0.020 mmol, 95%).

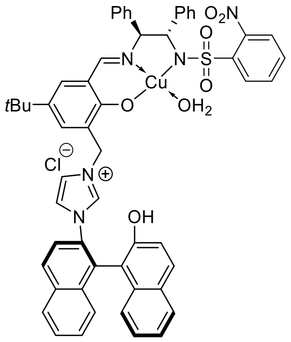

C1e-(*S*,*S*)

$C_{55}H_{48}ClCuN_5O_7S$, ***M***: 1022.06 g/mol. **m.p**. = 236 °C. **[α]$^{20}_{D}$** (c = 1.00 mg/mL, DCM): –37.0. **^{1}H NMR:** paramagnetic species. **^{13}C NMR:** paramagnetic species. **IR (solid):** $\tilde{\nu}$ = 3139, 3059, 3026, 2958, 2926, 2868, 1721, 1625, 1538, 1497, 1452, 1424, 1369, 1293, 1221, 1150, 1126, 1096, 1062, 1027, 996, 910, 816, 735, 700, 654, 631, 602, 552 cm^{-1}. **HRMS (ESI)** m/z: calculated for $[C_{55}H_{46}CuN_5O_6S]^+$: 967.2459, found: 967.2453.

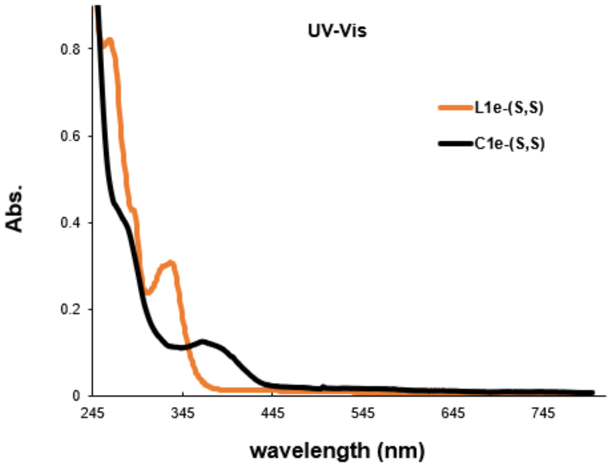

3-(5-(*tert*-Butyl)-3-((*E*)-(((1*R*,2*R*)-1,2-diphenyl-2-((2-nitrophenyl)sulfonamido)ethyl)imino)methyl)-2-hydroxybenzyl)-1-(2'-hydroxy-[1,1'-binaphthalen]-2-yl)-1*H*-imidazol-3-ium copper (II)-chloride (C1e-(*R*,*R*))

The synthesis was carried out according to **GP2** using ligand **L1e-(*R*,*R*)** (20.0 mg, 0.021 mmol, 1.0 equiv.) and $Cu(acac)_2$ (5.55 mg, 0.015 mmol, 1.0 equiv.). The pre-catalyst **C1e-(*R*,*R*)** was obtained as a pale green solid (14.9 mg, 0.020 mmol, 95%).

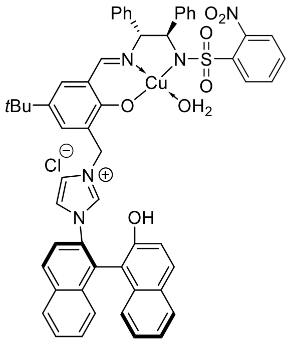

C1e-(*R*,*R*)

$C_{55}H_{48}ClCuN_5O_7S$, ***M***: 1022.06 g/mol. **m.p**. = 232 °C. **[α]$^{20}_{D}$** (c = 1.00 mg/mL, DCM): 24.0. **^{1}H NMR:** paramagnetic species. **^{13}C NMR:** paramagnetic species. **IR (solid):** $\tilde{\nu}$ = 3060, 3026,

2960, 2926, 1625, 1538, 1451, 1435, 1392, 1366, 1277, 1146, 1125, 1097, 911, 818, 730, 600, 582 cm^{-1}. **HRMS (ESI)** m/z: calculated for $[C_{55}H_{46}CuN_5O_6S]^+$: 967.2459. Found: 967.2454.

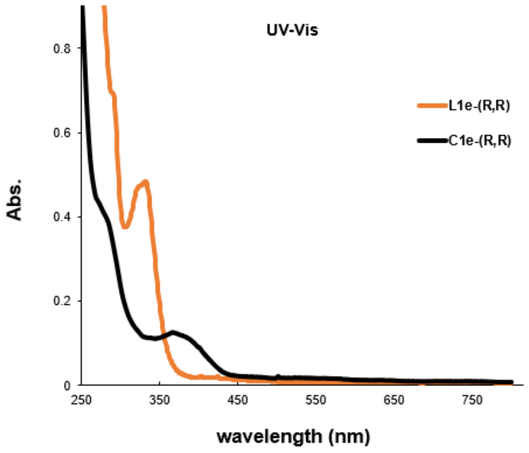

3-(5-(*tert*-Butyl)-3-((*E*)-(((1*S*,2*S*)-1,2-diphenyl-2-((4-nitrophenyl)sulfonamido)ethyl)imino)methyl)-2-hydroxybenzyl)-1-(2'-hydroxy-[1,1'-binaphthalen]-2-yl)-1*H*-imidazol-3-ium copper (II)-chloride (C1f-(*S*,*S*))

The synthesis was carried out according to **GP2** using ligand **L1f-(*S*,*S*)** (25.0 mg, 0.026 mmol, 1.0 equiv.) and $Cu(acac)_2$ (6.94 mg, 0.026 mmol, 1.0 equiv.). The pre-catalyst **C1f-(*S*,*S*)** was obtained as a pale green solid (25.1 mg, 0.025 mmol, 96%).

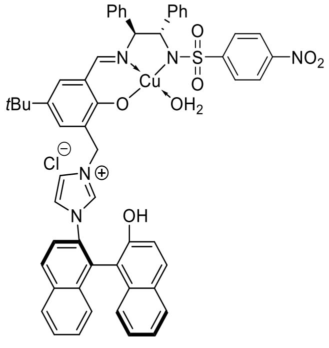

C1f-(*S*,*S*)

$C_{55}H_{48}ClCuN_5O_7S$, *M*: 1022.06 g/mol. **m.p.** = 180 °C. **$[\alpha]^{20}_D$** (c = 1.00 mg/mL, DCM): –49.2. **^{1}H NMR:** paramagnetic species. **^{13}C NMR:** paramagnetic species. **IR (solid):** $\tilde{\nu}$ = 3057, 2929, 1721, 1625, 1590, 1542, 1522, 1500, 1452, 1424, 1391, 1367, 1346, 1288, 1247, 1221, 1181, 1143, 1097, 1063, 1027, 994, 946, 910, 837, 814, 747, 734, 692, 619, 551 cm^{-1}. **HRMS (ESI)** m/z: calculated for $[C_{55}H_{46}CuN_5O_6S]^+$: 967.2459. Found: 967.2454.

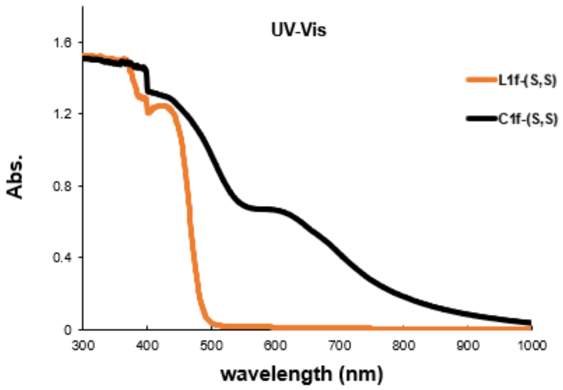

3-(5-(*tert*-Butyl)-3-((*E*)-(((1*R*,2*R*)-1,2-diphenyl-2-((4-nitrophenyl)sulfonamido)ethyl)imino)methyl)-2-hydroxybenzyl)-1-(2'-hydroxy-[1,1'-binaphthalen]-2-yl)-1*H*-imidazol-3-ium copper (II)-chloride (C1f-(*R*,*R*))

The synthesis was carried out according to **GP2** using ligand **L1f-(*R*,*R*)** (25.0 mg, 0.026 mmol, 1.0 equiv.) and Cu(acac)$_2$ (6.94 mg, 0.026 mmol, 1.0 equiv.). The pre-catalyst **C1f-(*R*,*R*)** was obtained as a pale green solid (24.7 mg, 0.024 mmol, 93%).

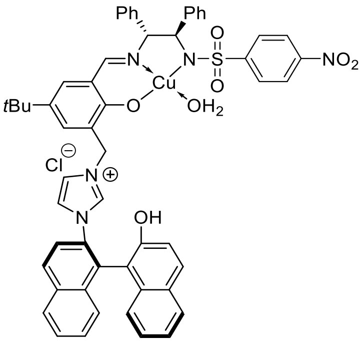

C1f-(*R*,*R*)

$C_{55}H_{48}ClCuN_5O_7S$, ***M***: 1022.06 g/mol. **m.p**. = 204 °C. **[α]$^{20}_{D}$** (c = 1.00 mg/mL, DCM): 31.2. **^{1}H NMR:** paramagnetic species. **^{13}C NMR:** paramagnetic species. **IR (solid):** $\tilde{\nu}$ = 3063, 2909, 1731, 1622, 1587, 1542, 1522, 1510, 1450, 1426, 1390, 1366, 1345, 1290, 1248, 1222, 1190, 1147, 1099, 1020, 995, 946, 911, 839, 811, 738, 691, 618, 552 cm^{-1}. **HRMS (ESI)** m/z: calculated for $[C_{55}H_{46}CuN_5O_6S]^+$: 967.2459. Found: 967.2454.

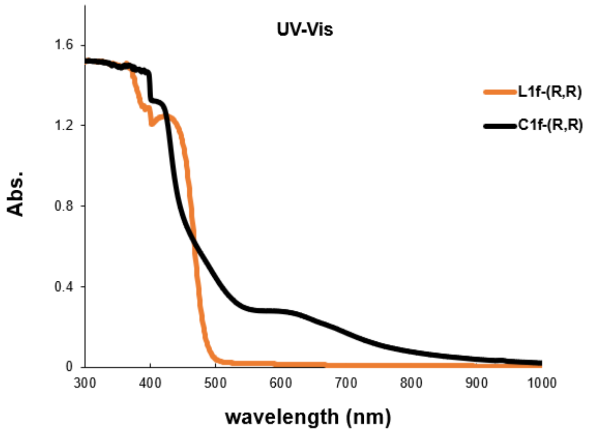

3-(5-(*tert*-Butyl)-2-hydroxy-3-((*E*)-(((1*S*,2*S*)-2-(methylsulfonamido)-1,2-diphenylethyl)imino)methyl)benzyl)-1-((*R*)-2'-hydroxy-[1,1'-binaphthalen]-2-yl)-1H-imidazol-3-ium copper (II) chloride (C1g-(*S*,*S*))

The synthesis was carried out according to **GP2** using ligand **L1g-(*S*,*S*)** (25.0 mg, 0.03 mmol, 1.0 equiv.) and Cu(acac)$_2$ (7.90 mg, 0.023 mmol, 1.0 equiv.). The pre-catalyst **C1g-(*S*,*S*)** was obtained as a pale green solid (26.3 mg, 0.029 mmol, 96%).

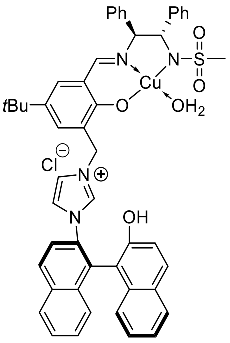

C1g-(*S*,*S*)

$C_{50}H_{47}ClCuN_4O_5S$, ***M***: 915.00 g/mol. **m.p**. = 223 °C. **[α]$^{20}_{D}$** (c = 1.00 mg/mL, DCM): –15.2. **^{1}H NMR:** paramagnetic species. **^{13}C NMR:** paramagnetic species. **IR (solid):** $\tilde{\nu}$ = 3137, 3060, 2959, 1713, 1625, 1543, 1510, 1494, 1434, 1346, 1267, 1222, 1120, 998, 937, 752, 735, 701, 653 cm^{-1}. **HRMS (ESI)** m/z: calculated for $[C_{50}H_{45}CuN_4O_4S]^+$: 860.2452. Found: 967.2454.

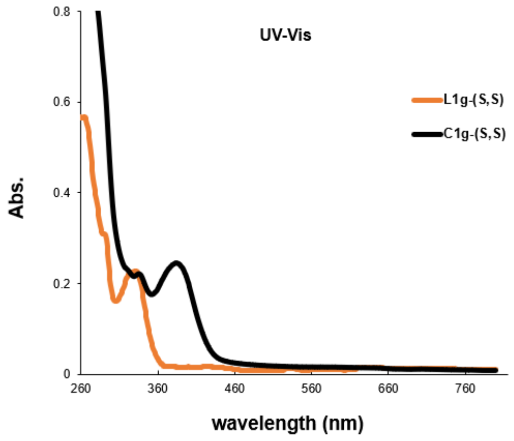

3-(5-(*tert*-Butyl)-3-((*E*)-(((1*R*,2*R*)-1,2-diphenyl-2(methylsulfonamido)ethyl)imino)methyl)-2-hydroxybenzyl)-1-(2'-hydroxy-[1,1' binaphthalen]-2-yl)-1*H*-imidazol-3-ium-copper(II)chloride (C1g-(*R*,*R*))

The synthesis was carried out according to **GP2** using ligand **L1g-(*R*,*R*)** (11.0 mg, 0.013 mmol, 1.0 equiv.) and $Cu(acac)_2$ (3.45 mg, 0.013 mmol, 1.0 equiv.). The pre-catalyst **C1g-(*R*,*R*)** was obtained as a pale green solid (10.8 mg, 0.012 mmol, 93%).

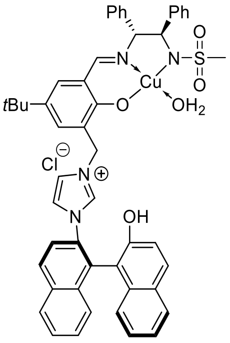

C1g-(*R*,*R*)

$C_{50}H_{47}ClCuN_4O_5S$, ***M***: 915.00 g/mol. **m.p**. = 196 °C. **[α]$^{20}_{D}$** (c = 1.00 mg/mL, DCM): 10.1. **^{1}H NMR:** paramagnetic species. **^{13}C NMR:** paramagnetic species. **IR (solid):** $\tilde{\nu}$ = 3138, 3062, 2955, 1717, 1621, 1545, 1514, 1492, 1437, 1345, 1269, 1220, 1121, 998, 935, 754, 730, 704, 651 cm^{-1}. **HRMS (ESI)** m/z: calculated for $[C_{50}H_{45}CuN_4O_4S]^+$: 860.2452. Found: 860.2456.

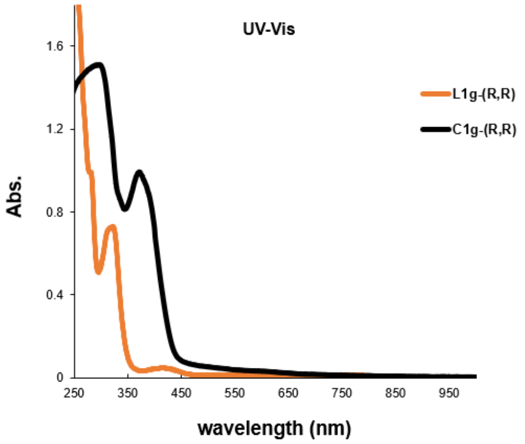

3-(5-(*tert*-Butyl)-3-((*E*)-(((1*S*,2*S*)-1,2-diphenyl-2-((4-methylbenzene)sulfonamido)ethyl)imino)methyl)-2-hydroxybenzyl)-1-(2'-hydroxy-[1,1'-binaphthalen]-2-yl)-1*H*-imidazol-3-ium copper (II)-chloride (C1h-(*S*,*S*))

The synthesis was carried out according to **GP2** using ligand **L1h-(*S*,*S*)** (15.4 mg, 0.017 mmol, 1.0 equiv.) and $Cu(acac)_2$ (4.42 mg, 0.017 mmol, 1.0 equiv.). The pre-catalyst **C1h-(*S*,*S*)** was obtained as a pale green solid (15.9 mg, 0.016 mmol, 96%).

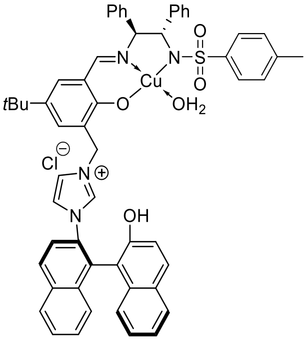

C1h-(*S*,*S*)

$C_{56}H_{51}ClCuN_4O_5S$, ***M***: 991.10 g/mol. **m.p.** = 208 °C. **$[\alpha]^{20}_D$** (c = 1.00 mg/mL, DCM): –50.6. **^{1}H NMR:** paramagnetic species. **^{13}C NMR:** paramagnetic species. **IR (solid):** $\tilde{\nu}$ = 3058, 2919, 1624, 1542, 1510, 1451, 1433, 1344, 1271, 1221, 1131, 1095, 991, 907, 815, 750, 734, 699 cm^{-1}. **HRMS (ESI)** m/z: calculated for $[C_{56}H_{49}CuN_4O_4S]^+$: 936.2765. Found: 936.2766.

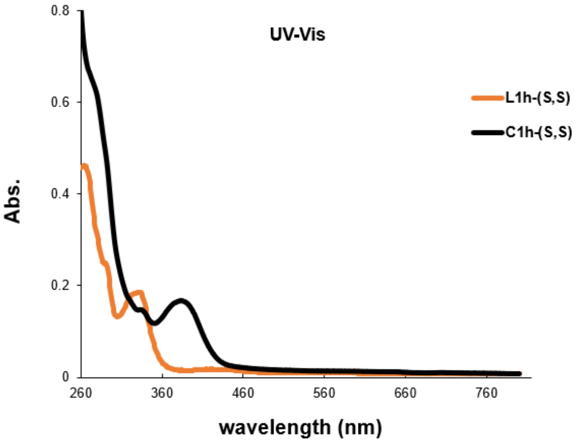

3-(5-(*tert*-Butyl)-3-((*E*)-(((1*R*,2*R*)-1,2-diphenyl-2-((4-methylbenzene)sulfonamido)ethyl)imino)methyl)-2-hydroxybenzyl)-1-(2'-hydroxy-[1,1'-binaphthalen]-2-yl)-1*H*-imidazol-3-ium copper (II)-chloride (C1h-(*R*,*R*))

The synthesis was carried out according to **GP2** using ligand **L1h-(*R*,*R*)** (15.0 mg, 0.016 mmol, 1.0 equiv.) and Cu(acac)$_2$ (4.31 mg, 0.016 mmol, 1.0 equiv.). The pre-catalyst **C1h-(*R*,*R*)** was obtained as a pale green solid (14.8 mg, 0.015 mmol, 95%).

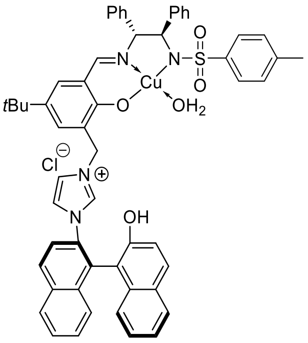

C1h-(*R*,*R*)

$C_{56}H_{51}ClCuN_4O_5S$, ***M***: 991.10 g/mol. **m.p**. = 198 °C. **[α]$^{20}_{D}$** (c = 1.00 mg/mL, DCM): 38.0. **^{1}H NMR:** paramagnetic species. **^{13}C NMR:** paramagnetic species. **IR (solid):** $\tilde{\nu}$ = 3060, 2956, 1626, 1543, 1514, 1495, 1452, 1435, 1346, 1273, 1221, 1132, 1098, 992, 815, 751, 699 cm^{-1}. **HRMS (ESI)** m/z: calculated for $[C_{56}H_{49}CuN_4O_4S]^+$: 936.2765. Found: 936.2768.

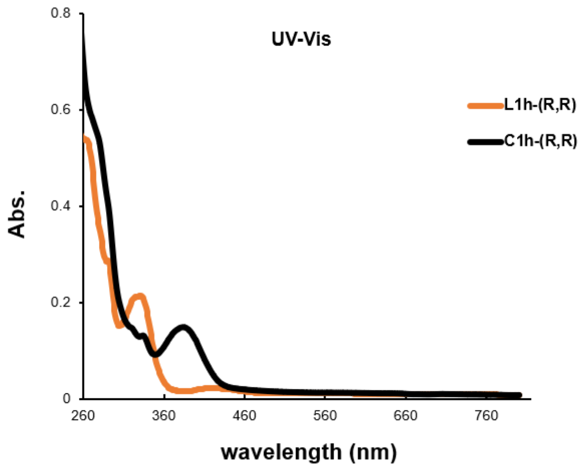

3-(5-(*tert*-Butyl)-3-((*E*)-(((1*S*,2*S*)-1,2-diphenyl-2-((perfluorophenyl)sulfonamido)ethyl)imino)methyl)-2-hydroxybenzyl)-1-(2'-hydroxy-[1,1'-binaphthalen]-2-yl)-1*H*-imidazol-3-ium copper (II)-chloride (C1i-(*S*,*S*))

The synthesis was carried out according to **GP2** using ligand **L1i-(*S*,*S*)** (15.0 mg, 0.015 mmol, 1.0 equiv.) and Cu(acac)$_2$ (3.98 mg, 0.015 mmol, 1.0 equiv.). The pre-catalyst **C1i-(*S*,*S*)** was obtained as a pale green solid (14.9 mg, 0.014 mmol, 95%).

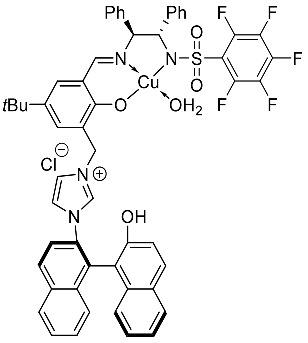

C1i-(*S*,*S*)

$C_{55}H_{44}ClCuF_5N_4O_5S$, ***M*:** 1049.00 g/mol. **m.p**. = 191 °C. **$[\alpha]^{20}_{D}$** (c = 1.00 mg/mL, DCM): –72.3. **^{1}H NMR:** paramagnetic species. **^{13}C NMR:** paramagnetic species. **IR (solid):** $\tilde{\nu}$ = 3062, 2961, 1627, 1575, 1544, 1518, 1487, 1452, 1433, 1272, 1154, 1096, 989, 938, 818, 700, 601 cm^{-1}. **HRMS (ESI)** m/z: calculated for $[C_{55}H_{42}CuN_4O_4S]^+$: 1012.2137. Found: 1012.2131.

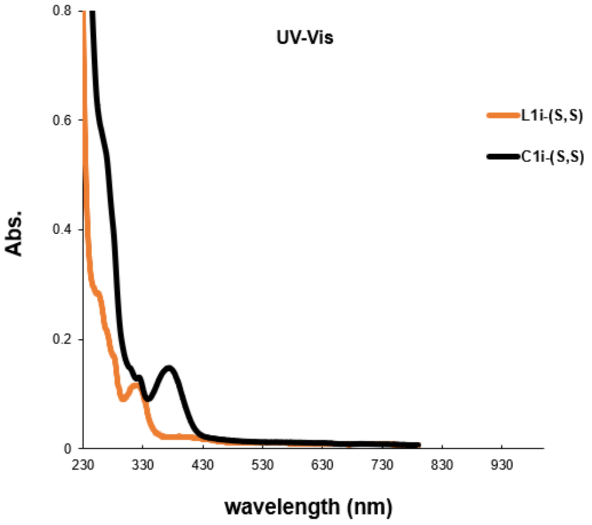

3-(5-(*tert*-Butyl)-3-((*E*)-(((1*R*,2*R*)-1,2-diphenyl-2-((perfluorophenyl)sulfonamido)ethyl)imino)methyl)-2-hydroxybenzyl)-1-(2'-hydroxy-[1,1'-binaphthalen]-2-yl)-1*H*-imidazol-3-ium copper (II)-chloride (C1i-(*R*,*R*))

The synthesis was carried out according to **GP2** using ligand **L1i-(*R*,*R*)** (15.0 mg, 0.015 mmol, 1.0 equiv.) and $Cu(acac)_2$ (3.98 mg, 0.015 mmol, 1.0 equiv.). The pre-catalyst **C1i-(*R*,*R*)** was obtained as a pale green solid (14.5 mg, 0.014 mmol, 92 %).

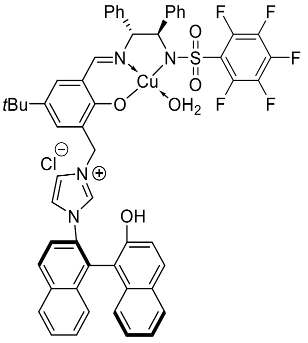

C1i-(*R*,*R*)

$C_{55}H_{44}ClCuF_5N_4O_5S$, ***M*:** 1049.00 g/mol. **m.p**. = 205 °C. **$[\alpha]^{20}_{D}$** (c = 1.00 mg/mL, DCM): 36.1. **^{1}H NMR:** paramagnetic species. **^{13}C NMR:** paramagnetic species. **IR (solid):** $\tilde{\nu}$ = 3060, 2960,

1626, 1577, 1543, 1516, 1489, 1450, 1434, 1275, 1151, 1095, 988, 938, 817, 701, 600 cm^{-1}.
HRMS (ESI) m/z: calculated for $[C_{55}H_{44}CuN_4O_4S]^+$: 1012.2137. Found: 1012.2137.

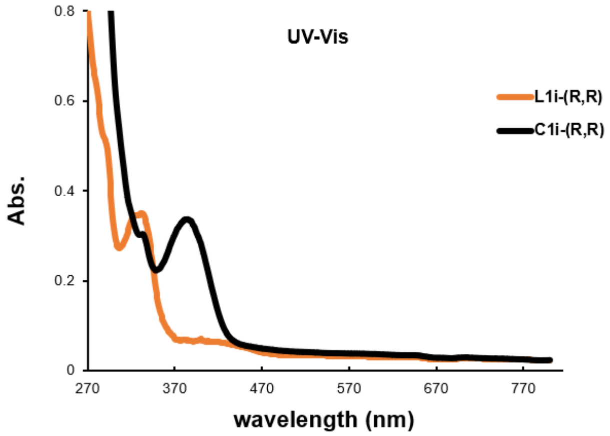

Screening of the Sulfonyl Residues of Catalyst C1.

Table S1.

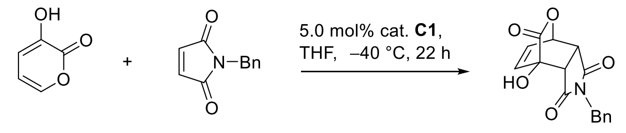

1a **2B** **3aB**

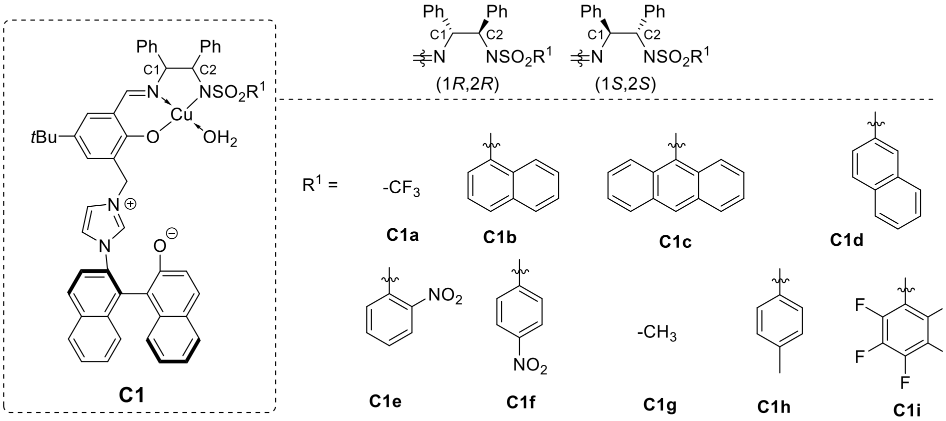

Entry	**C1** (config.)	yield[a] / [%]	dr[b]	ee[c] / [%]
1	**C1a** (1*S*,2*S*)	97	>98:2	90
2	**C1a** (1*R*,2*R*)	96	>98:2	–86
3	**C1b** (1*S*,2*S*)	98	>98:2	–96
4	**C1b** (1*R*,2*R*)	96	>98:2	–40
5	**C1c** (1*S*,2*S*)	98	>98:2	–90
6	**C1d** (1*S*,2*S*)	97	>98:2	–65
7	**C1d** (1*R*,2*R*)	98	>98:2	–34
8	**C1e** (1*S*,2*S*)	95	>98:2	36
9	**C1e** (1*R*,2*R*)	97	>98:2	–30
10	**C1f** (1*S*,2*S*)	94	>98:2	36
11	**C1f** (1*R*,2*R*)	96	>98:2	–13
12	**C1g** (1*S*,2*S*)	98	>98:2	16
13	**C1g** (1*R*,2*R*)	95	>98:2	–12
14	**C1h** (1*S*,2*S*)	97	>98:2	–51
15	**C1h** (1*R*,2*R*)	96	>98:2	50
16	**C1i** (1*S*,2*S*)	98	>98:2	32
17	**C1i** (1*R*,2*R*)	96	>98:2	–30

[a] Yield of isolated product. [b] *Endo*/*exo* ratios determined by ^1H NMR using the crude product. [c] The enantiomeric excess of the *endo*-isomer was determined by ^1H NMR using saturated $CDCl_3$ solution of (*R*)-(+)-binaphthol.[7] A minus sign indicates that the antipode of the enantiomer depicted was generated in excess.

Investigation of Maleimide Dienophiles (2) and Maleic Anhydride (6) in the Diels-Alder Reaction with 3-Hydroxypyrone (1a).

TableS2.

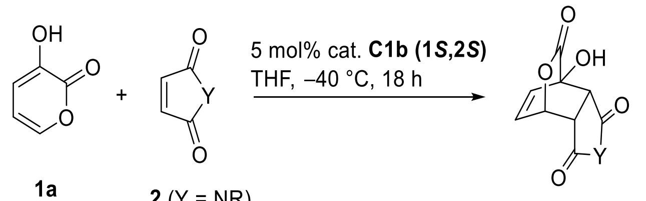

1a **2** (Y = NR) **6** (Y = O) ***ent*-3a** (Y = NR) **7** (Y = O)

#	**2** or **6**	R-N / O	(*ent*-)**3a** or **7**	yield[a]	dr[b]	ee[c]
1	**2A**	Me-N	*ent*-**3aA**	93	>98:2	93
2	**2B**	Bn-N	*ent*-**3aB**	98	>98:2	98
3	**2C**	4-O_2N-C_6H_4-N	*ent*-**3aC**	94	>98:2	94
4	**2D**	cyc-Hex-N	*ent*-**3aD**	92	>98:2	96
5[d]	**2E**	Boc-N	**3aE**	90	>98:2	–92
6[e]	**2F**	H-N	*ent*-**3aF**	89	>98:2	90
7	**2G**	Ph-N	*ent*-**3aG**	94	>98:2	91
8	**2H**	2,6-$(MeO)_2C_6H_3$-N	*ent*-**3aH**	93	>98:2	91
9	**2I**	3-Cl-C_6H_4-N	*ent*-**3aI**	93	>98:2	94
10	**2J**	4-F_3C-C_6H_4-N	*ent*-**3aJ**	90	>98:2	96
11	**2K**	3-O_2N-C_6H_4-N	*ent*-**3aK**	94	>98:2	95
12	**2L**	4-Cl-C_6H_4-N	*ent*-**3aL**	92	>98:2	90
13	**2M**	2,4,6-$Me_3C_6H_2$-N	*ent*-**3aM**	91	>98:2	97
14[d],[f]	**6**	O	**7**	92	>98:2	–84

[a] Yield of isolated product. [b] *Endo*/*exo* ratios determined by ^1H NMR using the crude product. [c] The enantiomeric excess was determined by ^1H NMR using saturated $CDCl_3$ solutions of (*R*)-(+)-binaphthol.[7] A minus sign indicates that the antipode of the enantiomer depicted was generated in excess. [d] **C1a** (1*S*, 2*S*) was used as catalyst. [e] The reaction was performed at –20 °C. [f] The reaction was performed at 0 °C.

Catalytic Diels−Alder Reactions of 3-Hydroxypyrone in Control Experiments

TableS3.

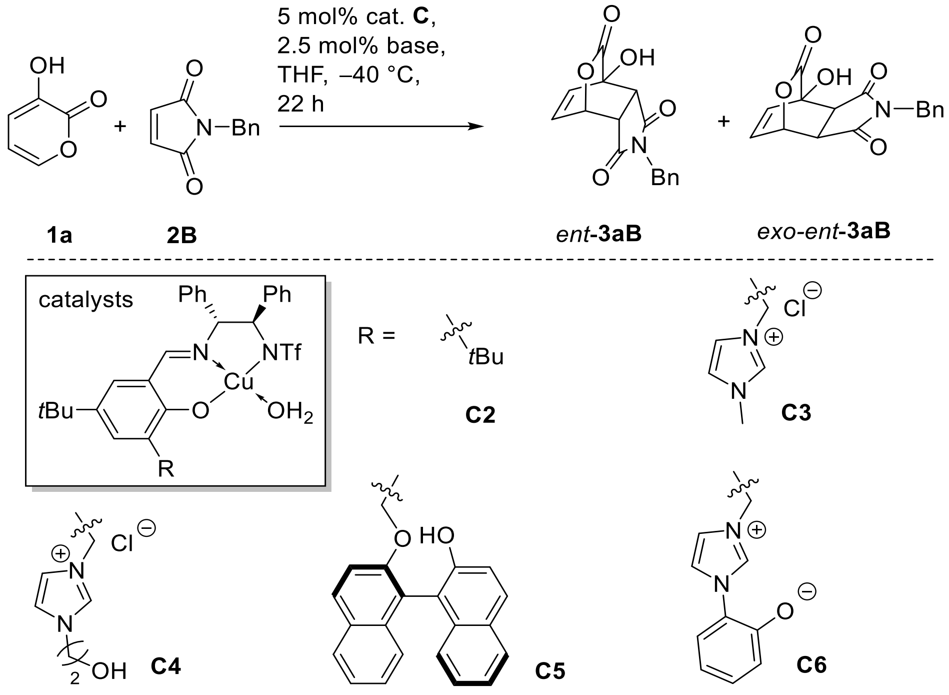

#	Base	Catalyst	yield[a] / [%]	*dr*[b]	*ee*[c](*endo*/*exo*) [%]
1	Cs_2CO_3	C2	92	84 / 16	–1 / –2
2	Et_3N	C2	81	94 / 6	–2 / 2
3	Cs_2CO_3	C3	96	82 / 18	–4 / 0
4	Et_3N	C3	65	96 / 4	26 / 0
5	Cs_2CO_3	C4	93	84 / 16	8 / 0
6	Et_3N	C4	88	94 / 6	10 / 0
7	K_2CO_3	C4	70	94 / 6	32 / 2
8	Cs_2CO_3	C5	94	90 / 10	42 / 5
9	Et_3N	C5	72	84 / 16	0 / 0
10	K_2CO_3	C5	85	92 / 8	5 / 0
11[d]	-	C6	95	> 98 / 2	86 / n.d.

[a] Yield of isolated product. [b] *Endo*/*exo* ratios determined by ^1H NMR using the crude product. [c] The enantiomeric excess was determined by ^1H NMR using saturated $CDCl_3$ solutions of (*R*)-(+)-binaphthol. A minus sign indicates that the antipode of the enantiomer depicted was generated in excess. [d] In the absence of additional base.

Catalytic Diels−Alder Reactions of 3-Hydroxypyrone in Control Experiments

TableS4.[11]

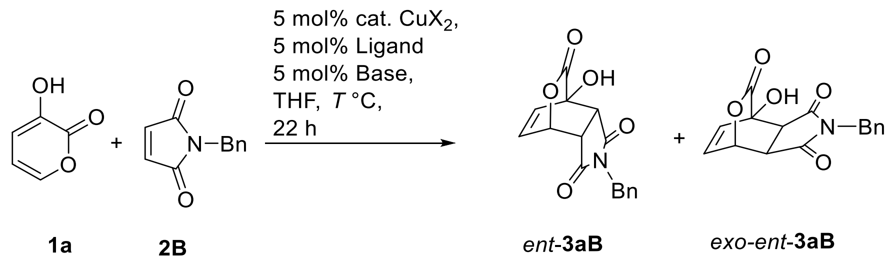

1a **2B** *ent*-**3aB** *exo*-*ent*-**3aB**

Ligands

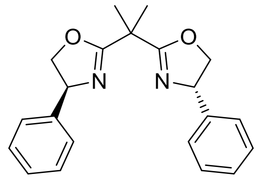

L2

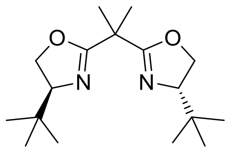

L3

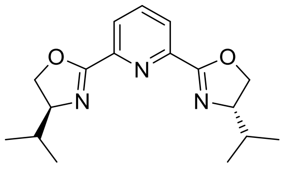

L4

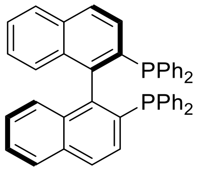

L5

#	Base	CuX_2	Ligand	T [°C]	yield[a] / [%]	*dr*[b]	*ee*[c](*endo*/*exo*) [%]
1	TEA	$Cu(OTf)_2$	L2	25	95	97 / 3	30 / n.d.
2	DIPEA	$Cu(OTf)_2$	L2	25	93	98 / 2	46 / n.d.
3	Cs_2CO_3	$Cu(OTf)_2$	L2	25	95	98 /2	0 / n.d.
4	DIPEA	$Cu(OTf)_2$	L2	–20	92	94 / 6	24 / 1
5	-[d]	$Cu(OAc)_2$	L2	–20	85	96 / 4	0 / n.d.
6	DIPEA	$Cu(OTf)_2$	L3	25	92	98 / 2	8 / n.d.
7	DIPEA	$Cu(OTf)_2$	L3	–20	42	97 / 3	5 / n.d.
8	DIPEA	$Cu(OTf)_2$	L4	25	92	90 /10	0 / 0
9	DIPEA	$Cu(OTf)_2$	L4	–20	55	92 /8	0 / 0
10	DIPEA	$Cu(OTf)_2$	L5	25	92	97 / 3	0 / 0
11	DIPEA	$Cu(OTf)_2$	L5	–20	94	98 / 2	2 / 0

[a] Yield of isolated product. [b] *Endo*/*exo* ratios determined by ^1H NMR using the crude product. [c] The enantiomeric excess was determined by ^1H NMR using saturated $CDCl_3$ solutions of (*R*)-(+)-binaphthol. [d] In the absence of additional base.

Characterization of Diels-Alder Adducts

(3a*S*,4*S*,7*S*,7a*R*)-4,7-Ethenopyranol [3,4-*c*] pyrrole-1,3,6(2*H*)-trione-3a,4,7,7a-tetrahydro-7-hydroxy-2-methyl (*ent*-3aA)

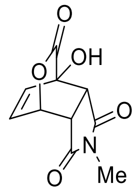

3aA

The product **3aA** was synthesized according to **GP4** using 3-hydroxypyrone **1a** (11.2 mg, 0.10 mmol, 1.0 equiv.), *N*-methylmaleimide **2A** (11.6 mg, 0.105 mmol, 1.05 equiv.) and catalyst **C1b** (4.86 mg, 0.005 mmol, 5.0 mol%). **3aA** was isolated as a white solid (20.7 mg, 0.093 mmol, 93%, *ee* = 93%). The enantiomeric excess was determined by ^{1}H NMR as described in **GP8**.

$C_{10}H_9NO_5$, ***M*:** 223.18 g/mol. **$[\alpha]^{20}_D$:** –75.4 (c = 1.0 mg/mL, acetone, sample with 93% *ee*). **^{1}H NMR ($CDCl_3$, 400 MHz, 21 °C):** δ (ppm) = 6.51 (*d*, *J* = 8.2 Hz, 1H, C*H*=CH), 6.42 (*dd*, *J* = 8.2, 2.6 Hz, 1H, C*H*=CH), 5.59 (*dt*, *J* = 4.7 Hz, 1.9 Hz, 1H, C*H*-O-C=O), 3.89 (*s*, 1H, O*H*), 3.76 (*dd*, *J* = 7.9, 2.6 Hz, 1H, O-CH-C*H*-C(=O)N), 3.12 (*d*, *J* = 8.2 Hz, 1H, C*H*-C(OH)-C=O), 2.97 (*s*, 3H, C*H*$_3$).

The NMR spectra is in agreement to the one reported in the literature.[12]

(3a*S*,4*S*,7*S*,7a*R*)-4,7-Ethenopyranol [3,4-*c*] pyrrole-1,3,6(2*H*)-trione-3a,4,7,7a-tetrahydro-7-hydroxy-2-(phenylmethyl) (*ent*-3aB)

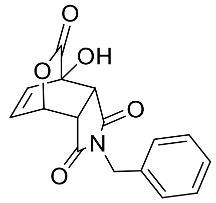

3aB

The product **3aB** was synthesized according to **GP4** using 3-hydroxypyrone **1a** (11.2 mg, 0.10 mmol, 1.0 equiv.), *N*-benzylmaleimide **2B** (19.6 mg, 0.105 mmol, 1.05 equiv.) and catalyst **C1b** (4.86 mg, 0.005 mmol, 5.0 mol%). **3aB** was isolated as a white solid (29.3 mg, 0.098 mmol, 98%, *ee* = 98%). The enantiomeric excess was determined by ^{1}H NMR as described in **GP8**.

$C_{16}H_{13}NO_5$, ***M*:** 299.28 g/mol. **$[\alpha]^{20}_D$:** –80.5 (c = 1.0 mg/mL, acetone, sample with 98% *ee*). **^{1}H NMR (CD_3CN, 500 MHz, 21 °C):** δ (ppm) = 7.37-7.24 (*m*, 3H, Ar*H*), 7.21 (*d*, *J* = 7.6 Hz, 2H, Ar*H*), 6.37-6.26 (*m*, 2H, C*H*=C*H*), 5.51 (*dt*, *J* = 4.4 Hz, 2.3 Hz, 1H, C*H*-O-C=O), 4.62 (*s*, 1H, O*H*), 4.52 (*s*, 2H, C*H*$_2$Ph), 3.82 (*dd*, *J* = 8.2, 4.8 Hz, 1H, O-CH-C*H*-C(=O)N), 3.18 (*d*, *J* = 8.2 Hz, 1H, C*H*-C(OH)-C=O).
The NMR spectra is in agreement to the one reported in the literature.[13]

(3a*S*,4*S*,7*S*,7a*R*)-4,7-Ethenopyranol [3,4-*c*] pyrrole-1,3,6(2*H*)-trione-3a,4,7,7a-tetrahydro-7-hydroxy-2-(4-nitrophenyl) (*ent*-3aC)

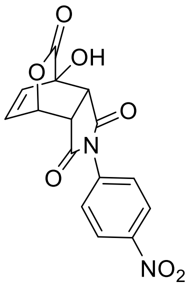

3aC

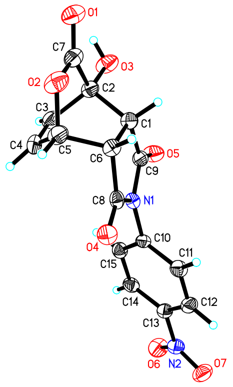

The product **3aC** was synthesized according to **GP4** using 3-hydroxypyrone **1a** (11.2 mg, 0.10 mmol, 1.0 equiv.), *N*-(4-nitrophenyl)maleimide **2C** (22.9 mg, 0.105 mmol, 1.05 equiv.) and catalyst **C1b** (4.86 mg, 0.005 mmol, 5.0 mol%). **3aC** was isolated as a white solid (31.0 mg, 0.094 mmol, 94%, *ee* = 94%). The enantiomeric excess was determined by ^{1}H NMR as described in **GP8**.

$C_{15}H_{10}N_2O_7$, ***M*:** 330.25 g/mol. **m.p.:** 185 °C. **$[\alpha]^{20}_{D}$** : –52.2 (c = 1.0 mg/mL, acetone, sample with 94% *ee*). **^{1}H NMR ($(CD_3)_2CO$, 400 MHz, 21 °C)**: δ (ppm) = 8.37 (*d*, *J* = 9.1 Hz, 2H, Ar*H*), 7.60 (*d*, *J* = 9.1 Hz, 2H, Ar*H*), 6.73-6.63 (*m*, 2H, C*H*=C*H*), 5.75 (*s*, 1H, O*H*), 5.67 (*dt*, *J* = 4.5, 1.9 Hz, 1H, C*H*-O-C=O), 4.16 (*dd*, *J* = 7.9, 4.7 Hz, 1H, O-CH-C*H*-C(=O)N), 3.52 (*d*, *J* = 8.3 Hz, 1H, C*H*-C(OH)-C=O). **^{13}C NMR ($(CD_3)_2CO$, 100 MHz, 21 °C):** δ (ppm) = 172.8, 172.3, 172.2, 147.9, 138.2, 137.5, 129.7, 128.2, 124.7, 76.2, 72.4, 47.5, 44.1. **IR (solid):** $\tilde{\nu}$ = 3463, 3082, 1768, 1718, 1596, 1525, 1497, 1379, 1348, 1309, 1249, 1183, 1141, 1054, 973, 917, 855, 747, 698 cm^{-1}. **HRMS (ESI)**: m/z calculated for $[C_{15}H_{10}N_2O_7Na]^+$: 353.0380, found: 353.0369.
CCDC 1995951 contains the supplementary crystallographic data for compound **3aC**. These data can be obtained free of charge from the Cambridge Crystallographic Data Centre via www.ccdc.cam.ac.uk/data_request/cif.

(3a*S*,4*S*,7*S*,7a*R*)-4,7-Ethenopyranol [3,4-*c*] pyrrole-1,3,6(2*H*)-trione-3a,4,7,7a-tetrahydro-7-hydroxy-2-cyclohexyl (*ent*-3aD)

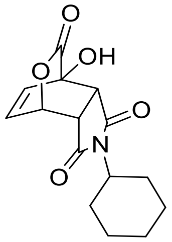

3aD

The product **3aD** was synthesized according to **GP4** using 3-hydroxypyrone **1a** (11.2 mg, 0.10 mmol, 1.0 equiv.), *N*-cyclohexylmaleimide **2D** (18.8 mg, 0.105 mmol, 1.05 equiv.) and catalyst **C1b** (4.86 mg, 0.005 mmol, 5.0 mol%). **3aD** was isolated as a white solid (29.4 mg, 0.092 mmol, 92%, *ee* = 96%). The enantiomeric excess was determined by ^{1}H NMR as described in **GP8**.

$C_{15}H_{17}NO_5$, ***M*:** 319.69 g/mol. **m.p.:** 174 °C. **$[\alpha]^{20}_D$:** –82.1 (c = 1.0 mg/mL, acetone, sample with 96% *ee*). **^{1}H NMR ($CDCl_3$, 300 MHz, 21 °C):** *δ* (ppm) = 6.49 (*d*, *J* = 8.3 Hz, 1H, C*H*=CH), 6.39 (*dd*, *J* = 8.3, 4.8 Hz, 1H, CH=C*H*), 5.55 (*dt*, *J* = 4.8, 1.9 Hz, 1H, C*H*-O), 3.59 (*br. s*, 1H, O*H*), 3.89 (*tt*, *J* = 12.5, 4.2 Hz, 1H, $NCH_{cyclohexyl}$), 3.68 (*dd*, *J* = 8.3, 4.8 Hz, 1H, O-CH-C*H*-C(=O)N), 3.04 (*d*, *J* = 8.3 Hz, 1H, C*H*-C(OH)-C=O), 2.11-1.95 (*m*, 2H, $CH_{2cyclohexyl}$), 1.80 (*d*, *J* = 12.5, 2H, $CH_{cyclohexyl}$), 1.64 (*d*, *J* = 12.3 Hz, 3H, $CH_{cyclohexyl}$), 1.50 (*d*, *J* = 12.5 Hz, 2H, $CH_{cyclohexyl}$), 1.35-1.10 (*m*, 2H, $CH_{cyclohexyl}$). **^{13}C NMR ($CDCl_3$, 100 MHz, 21°C):** *δ* (ppm) = 173.5, 173.2, 172.1, 136.9, 128.2, 75.2, 72.4, 52.6, 45.9, 42.5, 28.7, 28.6, 25.7, 25.0. **IR (solid):** $\tilde{\nu}$ = 3435, 2391, 2856, 2255, 1762, 1689, 1453, 1398, 1370, 1347, 1306, 1258, 1190, 1135, 1055, 980, 947, 908, 878, 852, 807, 792, 728, 695, 675, 641, 558, 441 cm^{-1}. **HRMS (ESI)**: m/z calculated for $[C_{15}H_{17}NO_5Na]^+$: 314.0999, found: 314.0998.

(3a*R*,4*R*,7*R*,7a*S*)-4,7-Ethenopyranol [3,4-*c*] pyrrole-1,3,6(2*H*)-trione-3a,4,7,7a-tetrahydro-7-hydroxy-2-*tert*-Butyloxycarbonyl (3aE)

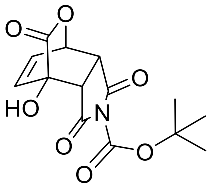

3aE

The product **3aE** was synthesized according to **GP4** using 3-hydroxypyrone **1a** (11.2 mg, 0.10 mmol, 1.0 equiv.), *N*-Boc maleimide **2E** (20.7 mg, 0.105 mmol, 1.05 equiv.) and catalyst **C1a** (4.56 mg, 0.005 mmol, 5.0 mol%). **3aE** was isolated as a white solid (27.8 mg, 0.090 mmol, 90%, *ee* = 92%). The enantiomeric excess was determined by ^{1}H NMR as described in **GP8**.

$C_{14}H_{15}NO_7$, ***M*:** 309.27 g/mol. **m.p.:** 141 °C. **$[\alpha]^{20}_D$:** 79.0 (c = 0.90 mg/mL, acetone, sample with 92% *ee*). **^{1}H NMR (CD_3CN, 400 MHz, 21 °C):** *δ* (ppm) = 6.59-6.46 (*m*, 2H, C*H*=C*H*), 5.56 (*dt*, *J* = 4.7 Hz, 2.3 Hz, 1H, -C*H*-O-C=O), 4.63 (*s*, 1H, O*H*), 3.90 (*dd*, *J* = 8.2, 4.8 Hz, 1H, O-CH-C*H*-C(=O)N), 3.25 (*d*, *J* = 8.2 Hz, 1H, C*H*-C(OH)-C=O), 1.54 (*s*, 9H, $C(CH_3)_3$). **^{13}C NMR (CD_3CN, 100 MHz, 21 °C):** *δ* (ppm) = 172.7, 170.7, 170.4, 146.8, 137.7, 130.1, 87.2, 76.1, 72.7, 47.5, 41.2, 27.8. **IR (solid):** $\tilde{\nu}$ = 3426, 3092, 2986, 1753, 1720, 1463, 1345, 1308, 1277, 1260, 1245, 1183, 1133, 1050, 1000, 980, 949, 845, 813, 790, 730, 699, 630, 597, 439 cm^{-1}. **HRMS (ESI):** m/z calculated for $[C_{14}H_{15}NO_7Na]^+$: 332.0741, found: 332.0736.

(3a*R*,4*R*,7*R*,7a*S*)-4,7-Ethenopyranol [3,4-*c*] pyrrole-1,3,6(2*H*)-trione-3a,4,7,7a-tetrahydro-7-hydroxy (3aF)

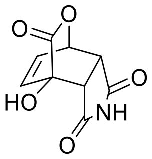

3aF

3aE (30.9 mg, 0.1 mmol, 1.0 equiv., sample with 92% of *ee*) was dissolved in DCM (3.0 mL) and TFA was added (11.5 µL, 0.15 mmol, 1.5 equiv.). The reaction mixture was stirred at room temperature until starting material was gone (3 h, monitored by TLC). The solvent was removed in vacuo and the crude product was dissolved in a small amount of DCM (0.2 mL) and *n*-pentane (5 mL) was added causing precipitation. The solid was filtered off and washed

with 5 mL of pentane to provide a white solid **3aF** (19.2 mg, 0.092 mmol, 92%, 92% of *ee*). The enantiomeric excess was determined by ^{1}H NMR as described in **GP8**.

The enantiomer (*ent*)-**3aF** was synthesized according to **GP4** using 3-hydroxypyrone **1a** (11.2 mg, 0.10 mmol, 1.0 equiv.), the parent maleimide **2F** (20.7 mg, 0.105 mmol, 1.05 equiv.) and **C1b** (4.86 mg, 0.005 mmol, 5.0 mol%). (*ent*)-**3aF** was isolated as a white solid after purification by column chromatography (PE : EE = 2:1 to pure EE) (18.6 mg, 0.089 mmol, 89%, *ee* = 90%). The enantiomeric excess was determined by ^{1}H NMR as described in **GP8**.

$C_9H_7NO_5$, ***M*:** 209.16 g/mol. **m.p.:** 135 °C. **$[\alpha]^{20}_{D}$:** 51.0 (c = 0.90 mg/mL, sample with 92% *ee*). (*ent*)-**3aF** **$[\alpha]^{20}_{D}$:** –62.1 (c = 0.95 mg/mL, sample with 90% *ee*). **^{1}H NMR ($(CD_3)_2SO$, 400 MHz, 21 °C):** δ (ppm) = 11.40 (*s*, 1H, N*H*), 6.73 (*s*, 1H, O*H*), 6.54-6.50 (*m*, 1H, C*H*=CH), 6.45 (*d*, *J* = 6.3 Hz, 1H, CH=C*H*), 5.47 (*dt*, *J* = 4.8 Hz, 1.8 Hz, 1H, C*H*-O-C=O), 3.79 (*dd*, *J* = 8.2, 4.7 Hz, 1H, O-CH-C*H*-C(=O)N), 3.12 (*d*, *J* = 8.2 Hz, 1H, C*H*-C(OH)-C=O). **^{13}C NMR ($(CD_3)_2SO$, 100 MHz, 21 °C):** δ (ppm) = 175.3, 174.6, 172.6, 136.9, 128.8, 75.2, 71.3, 47.6, 43.7. **IR (solid):** $\tilde{\nu}$ = 3257, 1763, 1712, 1359, 1309, 1188, 1142, 968, 693 cm^{-1}. **HRMS (ESI):** m/z calculated for $[C_9H_7NO_5Na]^+$: 232.0216, found: 232.0209.

(3a*S*,4*S*,7*S*,7a*R*)-4,7-Ethenopyranol [3,4-*c*] pyrrole-1,3,6(2*H*)-trione-3a,4,7,7a-tetrahydro-7-hydroxy-2-phenyl (*ent*-3aG)

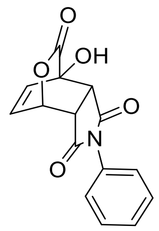

3aG

The product **3aG** was synthesized according to **GP4** using 3-hydroxypyrone **1a** (11.2 mg, 0.10 mmol, 1.0 equiv.), *N*-phenylmaleimide **2G** (18.1 mg, 0.105 mmol, 1.05 equiv.) and catalyst **C1b** (4.86 mg, 0.005 mmol, 5.0 mol%). **3aG** was isolated as a white solid (30.0 mg, 0.094 mmol, 94%, *ee* = 91%). The enantiomeric excess was determined by ^{1}H NMR as described in **GP8**.

$C_{15}H_{11}NO_5$, ***M*:** 319.69 g/mol. **m.p.:** 166 °C. **$[\alpha]^{20}_{D}$:** –28.0 (c = 0.82 mg/mL, acetone, sample with 90% *ee*). **^{1}H NMR (CD_3CN, 400 MHz, 21 °C):** δ (ppm) = 7.58-7.42 (*m*, 3H, Ar*H*), 7.24-7.14 (*m*, 2H, Ar*H*), 6.64-6.54 (*m*, 2H, Ar*H*), 5.62 (*dt*, *J* = 4.5 Hz, 1.0 Hz, 1H, C*H*-O-C=O), 4.74 (*br. s*, 1H, O*H*), 3.98 (*dd*, *J* = 8.0, 4.5 Hz, 1H, O-CH-C*H*-C(=O)N), 3.34 (*d*, *J* = 8.0 Hz, 1H, C*H*-C(OH)-C=O). **^{13}C NMR (CD_3CN, 100 MHz, 21 °C):** δ (ppm) = 173.8, 173.6, 173.0, 137.7, 132.9, 130.1, 130.0, 129.9, 127.8, 76.3, 73.0, 47.3, 44.0. **IR (solid):** $\tilde{\nu}$ = 3370, 3090, 2261, 2158, 1770, 1718, 1597, 1496, 1388, 1362, 1227, 1192, 1139, 1037, 972, 832, 695, 492 cm^{-1}. **HRMS (ESI)**: m/z calculated for $[C_{15}H_{11}NO_5Na]^+$: 308.0529, found: 308.0545.

(3a*S*,4*S*,7*S*,7a*R*)-4,7-Ethenopyranol [3,4-*c*] pyrrole-1,3,6(2*H*)-trione-3a,4,7,7a-tetrahydro-7-hydroxy-2-(2,6-dimethoxyphenyl) (*ent*-3aH)

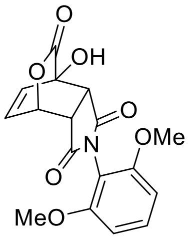

3aH

The product **3aH** was synthesized according to **GP4** using 3-hydroxypyrone **1a** (11.2 mg, 0.10 mmol, 1.0 equiv.), *N*-(2,6-dimethoxyphenyl)maleimide **2H** (24.4 mg, 0.105 mmol, 1.05 equiv.) and catalyst **C1b** (4.86 mg, 0.005 mmol, 5.0 mol%). **3aH** was isolated as a white solid (32.1 mg, 0.093 mmol, 93%, *ee* = 91%). The enantiomeric excess was determined by ^{1}H NMR as described in **GP8**.

$C_{17}H_{15}NO_7$, ***M*:** 345.30 g/mol. **m.p.:** 162 °C. **$[\alpha]^{20}_D$:** –74.9 (c = 1.0 mg/mL, acetone, sample with 91% *ee*). **^{1}H NMR (CD_3CN, 500 MHz, 21 °C):** δ (ppm) = 7.40 (*t*, *J* = 8.5 Hz, 1H, Ar*H*), 6.71 (*t*, *J* = 7.6 Hz, 2H, Ar*H*), 6.55-6.47 (*m*, 2H, C*H*=C*H*), 5.57 (*dt*, *J* = 4.7 Hz, 1.7 Hz, 1H, C*H*-O-C=O), 4.57 (*s*, 1H, O*H*), 3.96 (*dd*, *J* = 8.2, 4.7 Hz, 1H, O-CH-C*H*-C(=O)N), 3.75 (*s*, 6H, OC*H*$_3$), 3.32 (*d*, *J* = 8.2 Hz, 1H, C*H*-C(OH)-C=O). **^{13}C NMR (CD_3CN, 100 MHz, 21 °C):** δ (ppm) = 173.2, 173.1, 173.0, 157.2, 157.1, 137.5, 132.3, 129.8, 109.9, 105.4, 105.3, 76.4, 73.2, 56.8, 56.7, 47.7, 44.3. **IR (solid):** $\tilde{\nu}$ = 3453, 2947, 2844, 2259, 1765, 1710, 1599, 1503, 1482, 1446, 1385, 1363, 1306, 1262, 1196, 1139, 1111, 1054, 1030, 971, 916, 820, 791, 774, 756, 694, 481 cm^{-1}. **HRMS (ESI)**: m/z calculated for $[C_{17}H_{15}NO_7Na]^+$: 368.0741, found: 368.0745.

(3a*S*,4*S*,7*S*,7a*R*)-4,7-Ethenopyranol [3,4-*c*] pyrrole-1,3,6(2*H*)-trione-3a,4,7,7a-tetrahydro-7-hydroxy-2-(3-chlorophenyl) (*ent*-3aI)

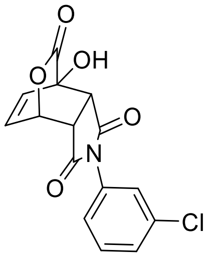

3aI

The product **3aI** was synthesized according to **GP4** using 3-hydroxypyrone **1a** (11.2 mg, 0.10 mmol, 1.0 equiv.), *N*-(3-chlorophenyl)maleimide **2I** (21.7 mg, 0.105 mmol, 1.05 equiv.) and catalyst **C1b** (4.86 mg, 0.005 mmol, 5.0 mol%). **3aI** was isolated as a white solid (29.7 mg, 0.093 mmol, 93%, *ee* = 94%). The enantiomeric excess was determined by ^{1}H NMR as described in **GP8**.

$C_{15}H_{10}NClO_5$, ***M***: 319.69 g/mol. **m.p.:** 174 °C. **$[\alpha]^{20}_D$:** –80.0 (c = 1.0 mg/mL, acetone, sample with 94% *ee*). **^{1}H NMR (CD_3CN, 400 MHz, 21 °C)**: δ (ppm) = 7.51-7.44 (*m*, 2H, Ar*H*), 7.27-7.22 (*m*, 1H, Ar*H*), 7.19-7.12 (*m*, 1H, Ar*H*), 6.60-6.52 (*m*, 2H, C*H*=C*H*), 5.60 (*dt*, *J* = 4.6, 2.4 Hz, 1H, C*H*-O), 4.73-4.56 (*br*, 1H, O*H*), 3.97 (*dd*, *J* = 8.4, 4.7 Hz, 1H, O-CH-C*H*-C(=O)N), 3.34 (*d*, *J* = 8.4 Hz, 1H, C*H*-C(OH)-C=O). **^{13}C NMR (CD_3CN, 100 MHz, 21 °C):** δ (ppm) = 173.5, 173.3, 173.0, 137.8, 134.9, 134.1, 131.6, 130.1, 130.0, 127.7, 126.4, 76.4, 73.0, 47.4, 44.1. **IR (solid):**

$\tilde{\nu}$ = 3434, 3077, 1765, 1709, 1647, 1593, 1480, 1380, 1185, 1138, 1078, 1054, 978, 931, 782, 695, 493 cm^{-1}. **HRMS (ESI)**: m/z calculated for $[C_{15}H_{10}NClO_5Na]^+$: 342.0140, found: 342.0140.

(3a*S*,4*S*,7*S*,7a*R*)-4,7-Ethenopyranol [3,4-*c*] pyrrole-1,3,6(2*H*)-trione-3a,4,7,7a-tetrahydro-7-hydroxy-2-(4-(trifluoromethyl)phenyl) (*ent*-3aJ)

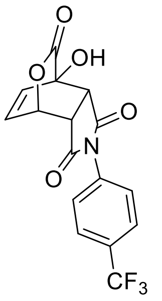

3aJ

The product **3aJ** was synthesized according to **GP4** using 3-hydroxypyrone **1a** (11.2 mg, 0.10 mmol, 1.0 equiv.), *N*-((4-trifluoromethyl)phenyl)maleimide **2J** (25.3 mg, 0.105 mmol, 1.05 equiv.) and catalyst **C1b** (4.86 mg, 0.005 mmol, 5.0 mol%). **3aJ** was isolated as a white solid (31.8 mg, 0.090 mmol, 90%, *ee* = 96%). The enantiomeric excess was determined by ^{1}H NMR as described in **GP8**.

$C_{16}H_{10}F_3NO_5$, ***M*:** 353.25 g/mol. **m.p.:** 180 °C. **$[\alpha]^{20}_D$**: –42.1 (c = 1.0 mg/mL, acetone, sample with 96% *ee*). **^{1}H NMR (CD_3CN, 400 MHz, 21 °C):** δ (ppm) = 7.82 (*d*, *J* = 8.5 Hz, 2H, Ar*H*), 7.40 (*d*, *J* = 8.3 Hz, 2H, Ar*H*), 6.61-6.54 (*m*, 2H, C*H*=C*H*), 5.61 (*dt*, *J* = 4.6 Hz, 2.6 Hz, 1H, -C*H*-O-C=O), 4.75 (*s*, 1H, O*H*), 3.99 (*dd*, *J* = 8.0, 4.4 Hz, 1H, O-CH-C*H*-C(=O)N), 3.36 (*d*, *J* = 8.1 Hz, 1H, C*H*-C(OH)-C=O). **^{19}F NMR (CD_3CN, 376 MHz, 21 °C):** δ (ppm) = –63.3. **^{13}C NMR (CD_3CN, 100 MHz, 21 °C):** δ (ppm) = 173.5, 173.3, 173.0, 137.9, 136.3, 131.3, 130.2, 128.5, 127.3, 127.2, 126.3, 123.6, 76.4, 73.1, 47.6, 44.3. **IR (solid):** $\tilde{\nu}$ = 3453, 3088, 2968, 1766, 1711, 1615, 1519, 1417, 1386, 1324, 1238, 1169, 1131, 1067, 1021, 972, 917, 873, 846, 815, 729, 696, 605, 506 cm^{-1}. **HRMS (ESI)**: m/z calculated for $[C_{16}H_{10}F_3NO_5Na]^+$: 376.0403, found: 376.0392.

(3a*S*,4*S*,7*S*,7a*R*)-4,7-Ethenopyranol [3,4-*c*] pyrrole-1,3,6(2*H*)-trione-3a,4,7,7a-tetrahydro-7-hydroxy-2-(3-nitrophenyl) (*ent*-3aK)

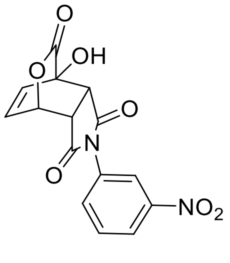

3aK

The product **3aK** was synthesized according to **GP4** using 3-hydroxypyrone **1a** (11.2 mg, 0.10 mmol, 1.0 equiv.), *N*-(3-nitrophenyl)maleimide **2K** (22.9 mg, 0.105 mmol, 1.05 equiv.) and catalyst **C1b** (4.86 mg, 0.005 mmol, 5.0 mol%). **3aK** was isolated as a white solid (31.0 mg, 0.094 mmol, 94%, *ee* = 95%). The enantiomeric excess was determined by ^{1}H NMR as described in **GP8**.

$C_{15}H_{10}N_2O_7$, ***M*:** 330.25 g/mol. **m.p.:** 179 °C. **$[\alpha]^{20}_D$**: –65.9 (c = 1.0 mg/mL, acetone, sample with 95% *ee*). **^{1}H NMR (CD_3CN, 400 MHz, 21 °C):** δ (ppm) = 8.27 (*ddd*, *J* = 8.2, 2.3, 1.1 Hz, 1H, Ar*H*), 8.13 (*t*, *J* = 2.1 Hz, 1H, Ar*H*), 7.66-7.60 (*m*, 2H, Ar*H*), 6.66-6.53 (*m*, 2H, C*H*=C*H*), 5.69 (*dt*, *J* = 4.5 Hz, 2.3 Hz, 1H, C*H*-O-C=O), 4.10 (*s*, 1H, O*H*), 4.00 (*dd*, *J* = 8.1, 4.8 Hz, 1H, O-CH-C*H*-C(=O)N), 3.37 (*d*, *J* = 8.2 Hz, 1H, C*H*-C(OH)-C=O). **^{13}C NMR (CD_3CN, 176 MHz, 21 °C):** δ (ppm) = 173.3, 173.1, 172.9, 149.5, 137.8, 133.8, 133.6, 131.3, 130.2, 124.7, 122.6, 76.3, 72.9, 47.5, 44.2. **IR (solid):** $\tilde{\nu}$ = 3465, 3092, 1767, 1716, 1532, 1484, 1384, 1352, 1309, 1238, 1187, 1141, 1092, 1055, 979, 939, 697, 483 cm^{-1}. **HRMS (ESI)**: m/z calculated for $[C_{15}H_{10}N_2O_7Na]^+$: 353.0380, found: 353.0369.

(3a*S*,4*S*,7*S*,7a*R*)-4,7-Ethenopyranol [3,4-*c*] pyrrole-1,3,6(2*H*)-trione-3a,4,7,7a-tetrahydro-7-hydroxy-2-(4-chlorophenyl) (*ent*-3aL)

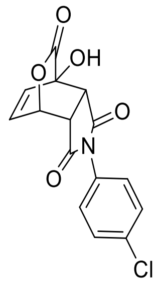

3aL

The product **3aL** was synthesized according to **GP4** using 3-hydroxypyrone **1a** (11.2 mg, 0.10 mmol, 1.0 equiv.), *N*-(4-chlorophenyl)maleimide **2L** (22.8 mg, 0.105 mmol, 1.05 equiv.) and catalyst **C1b** (4.86 mg, 0.005 mmol, 5.0 mol%).**3aL** was isolated as a white solid (29.4 mg, 0.092 mmol, 92%, *ee* = 90%). The enantiomeric excess was determined by ^{1}H NMR as described in **GP8**.

$C_{15}H_{10}NClO_5$, *M*: 319.69 g/mol. **m.p.:** 180 °C. **$[\alpha]^{20}_D$** : –69.4 (c = 1.0 mg/mL, acetone, sample with 90% *ee*). **^{1}H NMR ($(CD_3)_2SO$, 700 MHz, 21 °C)**: δ = 7.58-7.56 (*m*, 2H, Ar*H*), 7.21-7.19 (*m*, 2H, Ar*H*), 6.89 (*s*, 1H, O*H*), 6.61 (*dd*, *J* = 8.3, 4.9 Hz, 1H, C*H*=CH), 6.53 (*d*, *J* = 8.3 Hz, 1H, CH=C*H*), 5.61 (*dt*, *J* = 4.9, 1.9 Hz, 1H, O-C*H*), 4.01 (*dd*, *J* = 8.3, 4.9 Hz, 1H, O-CH-C*H*-C(=O)N), 3.34 (*dd*, *J* = 7.9, 0.88 Hz, 1H, C*H*-C(OH)-C=O). **^{13}C NMR ($(CD_3)_2SO$, 176 MHz, 21 °C)**: δ = 172.8, 172.4, 172.2, 136.9, 133.26, 130.6, 129.2, 129.0, 128.7, 75.3, 71.4, 46.4, 42.8. **IR (solid):** $\tilde{\nu}$ = 3437, 3078, 1766, 1711, 1493, 1386, 1307, 1188, 1140, 1091, 1055, 972, 811, 786, 756, 695, 508, 427 cm^{-1}. **HRMS (ESI)**: m/z calculated for $[C_{15}H_{10}NClO_5Na]^+$: 342.0140, found: 342.0135.

(3a*S*,4*S*,7*S*,7a*R*)-4,7-Ethenopyranol [3,4-*c*] pyrrole-1,3,6(2*H*)-trione-3a,4,7,7a-tetrahydro-7-hydroxy-2-(2,4,6-trimethylphenyl) (*ent*-3aM)

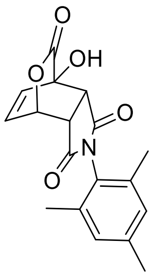

3aM

The product **3aM** was synthesized according to **GP4** using 3-hydroxypyrone **1a** (11.2 mg, 0.10 mmol, 1.0 equiv.), *N*-(2,4,6-trimethylphenyl)maleimide **2M** (22.6 mg, 0.105 mmol, 1.05 equiv.) and catalyst **C1b** (4.86 mg, 0.005 mmol, 5.0 mol%). **3aM** was isolated as a white solid (29.8 mg, 0.091 mmol, 91%, *ee* = 97%). The enantiomeric excess was determined by ^{1}H NMR as described in **GP8**.

$C_{18}H_{17}NO_5$, ***M*:** 327.34 g/mol. **$[\alpha]^{20}_{D}$:** –34.8 (c = 1.0 mg/mL, acetone, sample with 97% *ee*). **^{1}H NMR ($CDCl_3$, 400 MHz, 21 °C):** *δ* (ppm) = 6.97 (*s*, 1H, Ar*H*), 6.93 (*s*, 1H, Ar*H*), 6.65 (*s*, 1H, Ar*H*), 6.62 (*m*, 2H, C*H*=C*H*), 5.72 (*m*, 1H, C*H*-O-C=O), 4.05 (*s*, 1H, O*H*), 3.97 (*dd*, *J* = 4.4, 8.1 Hz, 1H, O-CH-C*H*-C(=O)N), 3.33 (*d*, *J* = 8.2 Hz, 1H, C*H*-C(OH)-C=O), 2.28 (*s*, 3H, CH_3), 2.03 (*s*, 3H, CH_3), 2.00 (*s*, 3H, CH_3).
The NMR spectra is in agreement to the one reported in the literature.[9]

(3a*R*,4*R*,7*S*,7a*S*)-4,7-Ethenopyranol-(8-methyl) [3,4-*c*] pyrrole-1,3,6(2*H*)-trione-3a,4,7,7a-tetrahydro-7-hydroxy-2-(phenylmethyl) (3bB)

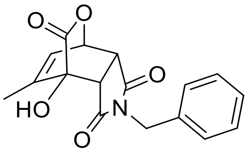

3bB

The product **3bB** was synthesized according to **GP4** using 4-methyl-3-hydroxypyrone **1b** (12.6 mg, 0.10 mmol, 1.0 equiv.), *N*-benzylmaleimide **2B** (19.6 mg, 0.105 mmol, 1.05 equiv.) and catalyst **C1a** (4.56 mg, 0.005 mmol, 5.0 mol%). **3bB** was isolated as a white solid (29.1 mg, 0.093 mmol, 93%, *ee* = 93%). The enantiomeric excess was determined by ^{1}H NMR as described in **GP8**.

$C_{17}H_{15}NO_5$, ***M*:** 313.30 g/mol. **m.p.:** 166 °C. **$[\alpha]^{20}_{D}$** : 53.9 (c = 1.0 mg/mL, acetone, sample with 93% *ee*). **^{1}H NMR (CD_3CN, 400 MHz, 21 °C):** *δ* (ppm) = 7.37-7.25 (*m*, 3H, Ar*H*), 7.24-7.18 (*m*, 2H, Ar*H*), 5.94 (*m*, 1H, C*H*=C-CH_3), 5.41 (*t*, *J* = 4.8 Hz, 1H, C*H*-O-C=O), 4.51 (*q*, *J* = 18.7, 14.5 Hz, 2H, CH_2Ph), 4.40 (*br*, 1H, O*H*), 3.77 (*dd*, *J* = 8.1, 4.8 Hz, 1H, O-CH-C*H*-C(=O)N), 3.14 (*d*, *J* = 7.9 Hz, 1H, C*H*-C(OH)-C=O), 1.52 (*d*, *J* = 1.8 Hz, 3H, CH_3). **^{13}C NMR (CD_3CN, 176 MHz, 21 °C):** *δ* (ppm) = 174.5, 174.2, 173.4, 146.6, 136.8, 129.4, 128.9, 128.6, 122.5, 77.5, 72.9, 47.7, 43.9, 43.0, 15.4. **IR (solid):** $\tilde{\nu}$ = 3449, 2957, 1758, 1693, 1431, 1396, 1342, 1312, 1291, 1237, 1156, 1133, 1086, 1029, 1004, 949, 903, 803, 772, 750, 699, 629, 484 cm^{-1}. **HRMS (ESI)**: m/z calculated for $[C_{17}H_{15}NO_5Na]^+$: 336.0842, found: 336.0838.

(3a*R*,4*S*,7*R*,7a*S*)-4,7-Ethenopyranol-(8-chloro) [3,4-*c*] pyrrole-1,3,6(2*H*)-trione-3a,4,7,7a-tetrahydro-7-hydroxy-2-(phenylmethyl) (3cB)

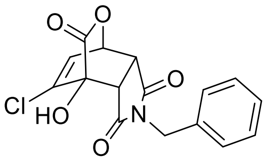

3cB

The product **3cB** was synthesized according to **GP4** using 4-chloro-3-hydroxypyrone **1c** (14.6 mg, 0.10 mmol, 1.0 equiv.), *N*-benzylmaleimide **2B** (19.6 mg, 0.105 mmol, 1.05 equiv.) and catalyst **C1a** (4.56 mg, 0.005 mmol, 5.0 mol%). **3cB** was isolated as a white solid (30.7 mg, 0.092 mmol, 92%, *ee* = 95%). The enantiomeric excess was determined by ^{1}H NMR as described in **GP8**.

$C_{16}H_{12}ClNO_5$, ***M*:** 333.72 g/mol. **m.p.:** 172 °C. **$[\alpha]^{20}_{D}$** : 80.5 (c = 0.98 mg/mL, acetone, sample with 95% *ee*). **^{1}H NMR ($CDCl_3$, 400 MHz, 21 °C):** δ (ppm) = 7.33-7.27 (*m*, 5H, Ar*H*), 6.16 (*d*, *J* = 5.4 Hz, 1H, C*H*=C-Cl), 5.53 (*t*, *J* = 5.2 Hz, 1H, C*H*-O-C=O), 4.61 (*q*, *J* = 14.2, 13.8 Hz, 2H, CH_2Ph), 4.11 (*br. s*, 1H, O*H*), 3.78 (*dd*, *J* = 7.7, 4.8 Hz, 1H, O-CH-C*H*-C(=O)N), 3.26 (*d*, *J* = 8.1 Hz, 1H, C*H*-C(OH)-C=O). **^{13}C NMR ($CDCl_3$, 176 MHz, 21 °C):** δ (ppm) = 172.2, 172.1, 169.7, 138.9, 134.8, 129.0, 128.9, 128.5, 123.1, 75.8, 71.9, 46.7, 43.3, 43.0. **IR (solid):** $\tilde{\nu}$ = 3434, 3087, 1767, 1696, 1612, 1496, 1455, 1432, 1396, 1316, 1296, 1236, 1170, 1137, 1079, 1004, 955, 907, 815, 779, 730, 701, 662, 619, 575, 526, 491, 491 cm^{-1}. **HRMS (ESI)**: m/z calculated for $[C_{16}H_{12}ClNO_5Na]^+$: 356.0296, found: 356.0292.

(3a*R*,4*S*,7*R*,7a*S*)-4,7-Ethenopyranol-(8-bromo) [3,4-*c*] pyrrole-1,3,6(2*H*)-trione-3a,4,7,7a-tetrahydro-7-hydroxy-2-(phenylmethyl) (3dB)

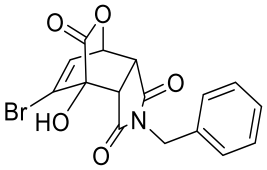

3dB

The product **3dB** was synthesized according to **GP4** using 4-bromo-3-hydroxypyrone **1d** (19.1 mg, 0.10 mmol, 1.0 equiv.), *N*-benzylmaleimide **2B** (19.6 mg, 0.105 mmol, 1.05 equiv.) and catalyst **C1a** (4.56 mg, 0.005 mmol, 5.0 mol%). **3dB** was isolated as a white solid (34.0 mg, 0.094 mmol, 94%, *ee* = 93%). The enantiomeric excess was determined by ^{1}H NMR as described in **GP8**.

$C_{16}H_{12}BrNO_5$, ***M*:** 362.18 g/mol. **m.p.:** 182 °C. **$[\alpha]^{20}_{D}$:** 82.8 (c = 1.0 mg/mL, sample with 93% *ee*). **^{1}H NMR ($(CD_3)_2SO$, 500 MHz, 21 °C):** δ (ppm) = 7.44 (*s*, 1H, O*H*), 7.35-7.22 (*m*, 3H, Ar*H*), 7.16 (*d*, *J* = 7.5 Hz, 2H, Ar*H*), 6.80 (*d*, *J* = 5.8 Hz, 1H, C*H*=C-Br), 5.59 (*t*, *J* = 5.1 Hz, 1H, C*H*-O-C=O), 4.50 (*q*, *J* = 15.1, 10.6 Hz, 2H, CH_2Ph), 3.93 (*dd*, *J* = 7.3, 4.5 Hz, 1H, O-CH-C*H*-C(=O)N), 3.44 (*d*, *J* = 7.8 Hz, 1H, C*H*-C(OH)-C=O). **^{13}C NMR ($(CD_3)_2SO$, 100 MHz, 21 °C):** δ (ppm) = 173.1, 171.8, 170.9, 135.5, 129.2, 128.5, 128.4, 127.4, 127.3, 75.9, 72.0, 46.5, 43.0, 41.8. **IR (solid):** $\tilde{\nu}$ = 3064, 2922, 1768, 1700, 1607, 1430, 1395, 1353, 1316, 1275, 1171, 1132, 1051, 1024, 1001, 954, 943, 821, 804, 732, 700, 614, 568, 486 cm^{-1}. **HRMS (ESI)**: m/z calculated for $[C_{16}H_{12}BrNO_5Na]^+$: 399.9791, found: 399.9800.

(3a*S*,4*S*,7*S*,7a*R*)-4,7-Etheno-1*H*-pyrrolo [3,4-*c*] pyridine-1,3,6(2*H,* 3a*H*)-trione-4,5,7,7a-tetrahydro-7-hydroxy-2-(methylphenyl)-5-[(2-nitrophenyl)sulfonyl] (*ent*-5aB)

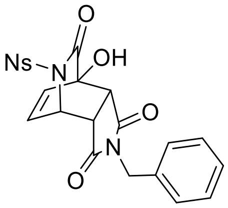

5aB

The product **5aB** was synthesized according to **GP6** using *N*-nosyl-3-hydroxypyridone **4a** (29.6 mg, 0.10 mmol, 1.0 equiv.), *N*-benzylmaleimide **2B** (19.6 mg, 0.105 mmol, 1.05 equiv.) and catalyst **C1b** (4.96 mg, 0.005 mmol, 5.0 mol%). **5aB** was isolated as a white solid (46.9 mg, 0.097 mmol, 97%, *ee* = 94%). The enantiomeric excess was determined by ^{1}H NMR as described in **GP8**.

$C_{22}H_{17}N_3O_8S$, ***M*:** 483.45 g/mol. **m.p.:** 193 °C. **$[\alpha]^{20}_{D}$:** –92.3 (c = 1.0 mg/mL, acetone, sample with 94% *ee*). **^{1}H NMR (CD_3CN, 400 MHz, 21 °C):** δ (ppm) = 8.37 (*d*, *J* = 7.6 Hz, 1H, Ar*H*), 7.98-7.86 (*m*, 3H, Ar*H*), 7.39-7.21 (*m*, 5H, Ar*H*), 6.55 (*dd*, *J* = 8.7, 5.6 Hz, 1H, C*H*=C*H*), 6.18 (*d*, *J* = 8.0 Hz, 1H, C*H*=C*H*), 5.50 (*dt*, *J* = 4.8, 1.7 Hz, 1H, C*H*-O-C=O), 4.59 (*br. s*, 1H, O*H*), 4.62-4.52 (*m*, 2H, C*H*$_2$Ph), 3.90 (*dd*, *J* = 7.9, 4.1 Hz, 1H, O-CH-C*H*-C(=O)N), 3.25 (*d*, *J* = 8.1 Hz, 1H, C*H*-C(OH)-C=O). **^{13}C NMR (CD_3CN, 100 MHz, 21 °C):** δ (ppm) = 174.3, 173.6, 170.8, 148.3, 136.9, 136.4, 136.3, 133.9, 133.4, 131.1, 131.0, 129.1, 128.5, 128.2, 125.6, 78.8, 53.2, 47.8, 44.4, 42.9. **IR (solid):** $\tilde{\nu}$ = 3094, 2261, 1742, 1711, 1546, 1376, 1351, 1183, 1156, 1038, 960, 915, 832, 780, 594, 556 cm^{-1}. **HRMS (ESI)**: m/z calculated for $[C_{22}H_{17}N_3O_8SNa]^+$: 506.0629, found: 506.0628.

(3a*S*,4*S*,7*S*,7a*R*)-4,7-Etheno-1*H*-pyrrolo [3,4-*c*] pyridine-1,3,6(2*H,* 3a*H*)-trione-4,5,7,7a-tetrahydro-7-hydroxy-2-(4-nitrophenyl)-5-[(2-nitrophenyl)sulfonyl] (*ent*-5aC)

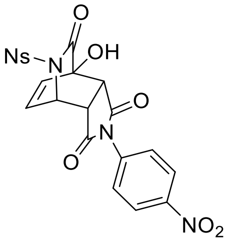

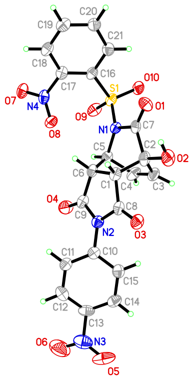

5aC

The product **5aC** was synthesized according to **GP6** using *N*-nosyl-3-hydroxypyridone **4a** (29.6 mg, 0.10 mmol, 1.0 equiv.), *N*-(4-nitrophenyl)maleimide **2C** (22.9 mg, 0.105 mmol, 1.05 equiv.) and catalyst **C1b** (4.96 mg, 0.005 mmol, 5.0 mol%). **5aC** was isolated as a white solid (48.3 mg, 0.094 mmol, 94%, *ee* = 94%). The enantiomeric excess was determined by ^{1}H NMR as described in **GP8**.

$C_{21}H_{14}N_4O_{10}S$, ***M*:** 514.42 g/mol. **m.p.:** 215 °C. **$[\alpha]^{20}_{D}$:** –110.2 (c = 1.0 mg/mL, acetone, sample with 94% *ee*). **^{1}H NMR ($(CD_3)_2SO$, 400 MHz, 21 °C):** δ (ppm) = 8.40 (*d*, *J* = 9.0 Hz, 2H, Ar*H*), 8.35 (*dd*, *J* = 7.5, 1.7 Hz, 1H, Ar*H*), 8.18 (*dd*, *J* = 7.5, 1.7 Hz, 1H, Ar*H*), 8.11-8.00 (*m*, 2H, Ar*H*),

7.56 (*d*, *J* = 9.0 Hz, 2H, Ar*H*), 7.03 (*s*, 1H, O*H*), 6.83 (*dd*, *J* = 8.3, 5.7 Hz, 1H, C*H*=C*H*), 6.47 (*d*, *J* = 8.6 Hz, 1H, C*H*=C*H*), 5.44 (*dt*, *J* = 5.1, 1.8 Hz, 1H, C*H*-O-C=O), 4.09 (*dd*, *J* = 8.4, 4.1 Hz, 1H, O-CH-C*H*-C(=O)N), 3.49 (*d*, *J* = 8.1 Hz, 1H, C*H*-C(OH)-C=O). **^{13}C NMR ($(CD_3)_2SO$, 100 MHz, 21 °C):** δ (ppm) = 172.8, 171.6, 170.3, 147.1, 146.9, 137.1, 136.7, 136.3, 133.1, 132.8, 130.2, 129.7, 127.8, 125.3, 124.4, 78.3, 52.1, 47.4, 43.8. **IR (solid):** $\tilde{\nu}$ = 3096, 1719, 1595, 1543, 1527, 1497, 1375, 1347, 1182, 1105, 849, 739, 579, 558 cm^{-1}. **HRMS (ESI)**: m/z calculated for $[C_{21}H_{14}N_4O_{10}SNa]^+$: 537.0323 , found: 537.0322.

CCDC 2003855 contains the supplementary crystallographic data for compound **5aC**. These data can be obtained free of charge from the Cambridge Crystallographic Data Centre via www.ccdc.cam.ac.uk/data_request/cif.

(3a*S*,4*S*,7*S*,7a*R*)-4,7-Etheno-1*H*-pyrrolo [3,4-*c*] pyridine-1,3,6(2*H*, 3a*H*)-trione-4,5,7,7a-tetrahydro-7-hydroxy-5-[(2-nitrophenyl)sulfonyl] (*ent*-5aF)

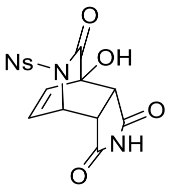

5aF

The product **5aF** was synthesized according to **GP6** using *N*-nosyl-3-hydroxypyridone **4a** (29.6 mg, 0.10 mmol, 1.0 equiv.), maleimide **2F** (10.2 mg, 0.105 mmol, 1.05 equiv.) and catalyst **C1b** (4.96 mg, 0.005 mmol, 5.0 mol%). **5aF** was isolated as a white solid (37.4 mg, 0.095 mmol, 94%, *ee* = 95%). The enantiomeric excess was determined by ^{1}H NMR as described in **GP8**.

$C_{15}H_{11}N_3O_8S$, ***M*:** 393.32 g/mol. **m.p.:** 198 °C. **$[\alpha]^{20}_D$:** –98.3 (c = 1.0 mg/mL, acetone, sample with 95% *ee*). **^{1}H NMR (CD_3CN, 400 MHz, 21 °C):** δ (ppm) = 8.37 (*d*, *J* = 6.8 Hz, 1H, Ar*H*), 8.00-7.85 (*m*, 3H, Ar*H*), 6.69 (*m*, 1H, C*H*=C*H*), 6.34 (*d*, *J* = 7.9 Hz, 1H, C*H*=C*H*), 5.44 (*dt*, *J* = 4.7, 1.6 Hz, 1H, C*H*-O-C=O), 3.84 (*dd*, *J* = 8.2, 4.3 Hz, 1H, O-CH-C*H*-C(=O)N), 3.19 (*d*, *J* = 8.1 Hz, 1H, C*H*-C(OH)-C=O). **^{13}C NMR (CD_3CN, 176 MHz, 21 °C):** δ (ppm) = 174.8, 174.0, 170.9, 136.9, 136.6, 135.7, 133.9, 133.4, 131.1, 130.9, 125.6, 78.7, 53.1, 49.0, 45.7. **IR (solid):** $\tilde{\nu}$ = 2360, 2261, 2178, 1721, 1542, 1352, 1183, 1102, 1044, 832, 779 cm^{-1}. **HRMS (ESI)**: m/z calculated for $[C_{15}H_{12}N_3O_8S]^+$: 394.0340, found: 394.0341.

(3a*S*,4*S*,7*R*,7a*R*)-4,7-Etheno-1*H*-pyrrolo[3,4-*c*] pyridine-1,3,6(2*H*, 3a*H*)-trione-4,5,7,7a-tetrahydro-7-hydroxy-8-allyl-2-(methylphenyl)-5-[(2-nitrophenyl)sulfonyl] (*ent*-5bB)

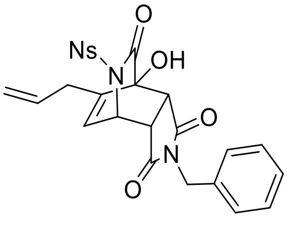

5bB

The product **5bB** was synthesized according to **GP6** using *N*-nosyl-4-allyl-3-hydroxypyridone **4b** (33.6 mg, 0.10 mmol, 1.0 equiv.), *N*-benzylmaleimide **2B** (19.6 mg, 0.105 mmol, 1.05

equiv.) and catalyst **C1b** (4.96 mg, 0.005 mmol, 5.0 mol%). **5bB** was isolated as a white solid (49.7 mg, 0.095 mmol, 95%, *ee* = 95%). The enantiomeric excess was determined by ^{1}H NMR as described in **GP8**.

$C_{25}H_{21}N_3O_8S$, ***M*:** 523.52 g/mol. **m.p.:** 181°C. **$[\alpha]^{20}_{D}$:** –96.1 (c = 1.0 mg/mL, sample with 96% *ee*). **^{1}H NMR (CD_3CN, 400 MHz, 21 °C):** δ (ppm) = 8.35 (*d*, *J* = 7.7 Hz, 1H, Ar*H*), 7.97-7.86 (*m*, 3H, Ar*H*), 7.38-7.24 (*m*, 5H, Ar*H*), 6.10 (*dt*, *J* = 6.0 Hz, 1H, C*H*=C), 5.44 (*dd*, *J* = 6.3, 3.9 Hz, C*H*-O-C=O), 5.42-5.33 (*m*, 1H, C*H*=CH_{2allyl}), 4.92 (*m*, 1H, CH=CH_{2allyl}), 4.92 (*dq*, *J* = 17.0, 3.7, 1.6 Hz,1H, CH=CH_{2allyl}), 4.54 (*m*, 2H, CH_2Ph), 4.48 (*br. s*, 1H, O*H*), 3.85 (*dd*, *J* = 8.1, 4.1 Hz, 1H, O-CH-C*H*-C(=O)N), 3.23 (*d*, *J* = 8.1 Hz, 1H, C*H*-C(OH)-C=O), 2.79-2.69 (*m*, 1H, OCH_2), 2.53-2.43 (*m*, 1H, OCH_2). **^{13}C NMR (CD_3CN, 176 MHz, 21 °C):** δ (ppm) = 174.8, 174.0, 171.4, 148.7, 147.9, 137.3, 136.9, 134.3, 134.1, 133.8, 131.5, 129.6, 129.2, 128.8, 126.0, 123.9, 180.1, 53.4, 48.3, 45.1, 43.3, 33.6. **IR (solid):** $\tilde{\nu}$ = 3466, 1701, 1542, 1432, 1396, 1312, 1266, 1177, 1123, 1100, 980, 927, 735, 701, 558 cm^{-1}. **HRMS (ESI)**: m/z calculated for $[C_{25}H_{21}N_3O_8SNa]^+$: 546.0942, found: 546.0943.

(3a*S*,4*R*,7*S*,7a*R*)-4,7-Etheno-1*H*-pyrrolo[3,4-*c*] pyridine-1,3,6(2*H,* 3a*H*)-trione-4,5,7,7a-tetrahydro-7-hydroxy-8-chloro-2-(methylphenyl)-5-[(2-nitrophenyl)sulfonyl] (*ent*-5cB)

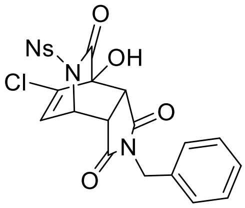

5cB

The product **5cB** was synthesized according to **GP6** using *N*-nosyl-4-chloro-3-hydroxypyridone **4c** (33.1 mg, 0.10 mmol, 1.0 equiv.), *N*-benzylmaleimide **2B** (19.6 mg, 0.105 mmol, 1.05 equiv.) and catalyst **C1b** (4.96 mg, 0.005 mmol, 5.0 mol%). **5cB** was isolated as a white solid (50.2 mg, 0.097 mmol, 97%, *ee* = 79%). The enantiomeric excess was determined by ^{1}H NMR as described in **GP8**.

$C_{22}H_{16}ClN_3O_8S$, ***M*:** 517.89 g/mol. **m.p.:** 167°C. **$[\alpha]^{20}_{D}$:** –92.1 (c = 1.0 mg/mL, acetone, sample with 79% *ee*). **^{1}H NMR (CD_3CN, 400 MHz, 21 °C):** δ (ppm) = 8.38 (*d*, *J* = 7.1 Hz, 1H Ar*H*), 8.00-7.87 (*m*, 3H, Ar*H*), 7.41-7.20 (*m*, 5H, Ar*H*), 6.60 (*d*, *J* = 6.6 Hz, 1H, C*H*=C), 5.53 (*dd*, *J* = 6.6, 4.0 Hz, C*H*-O-C=O), 4.92 (*br. s*, 1H, O*H*), 4.58 (*m*, 2H, CH_2Ph), 3.94 (*dd*, *J* = 7.6, 4.2 Hz, 1H, O-CH-C*H*-C(=O)N), 3.39 (*d*, *J* = 8.2 Hz, 1H, C*H*-C(OH)-C=O). **^{13}C NMR (CD_3CN, 75 MHz, 21 °C):** δ (ppm) = 173.8, 172.7, 168.9, 148.2, 138.1, 137.1, 136.2, 134.0, 133.5, 130.8, 129.1, 128.3, 128.2, 126.3, 125.8, 79.2, 53.1, 48.0, 44.6, 42.9. **IR (solid):** $\tilde{\nu}$ = 3467, 3093, 1779, 1737, 1702, 1591, 1541, 1434, 1395, 1352, 1289, 1177, 1102, 942, 852, 735, 583, 557, 495 cm^{-1}. **HRMS (ESI)**: m/z calculated for $[C_{22}H_{16}ClN_3O_8SNa]^+$: 540.0239, found: 540.0242.

(3a*S*,4*S*,7*S*,7a*R*)-4,7-Etheno-1*H*-pyrrolo[3,4-*c*] pyridine-1,3,6(2*H,* 3a*H*)-trione-4,5,7,7a-tetrahydro-7-hydroxy-2-(methylphenyl) (*ent*-5dB)

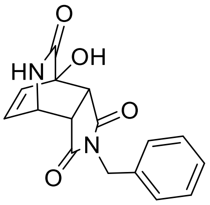

5dB

To a solution of the **5aB** (48.3 mg, 0.1 mmol, 1.0 equiv., sample with 94% of *ee*) in THF (5.0 mL) was added DBU (16.7 mg, 0.11 mmol, 1.1 equiv.) and PhSH (11.6 mg, 0.105 mmol, 1.05 equiv.). The resulting reaction mixture was stirred for 10 min at room temperature, diluted with water (5 mL) and extracted with AcOEt (3 x 5 mL). The combined organic phases were dried over Na_2SO_4 and solvent was evaporated under reduced pressure. The crude product was dissolved in a small amount of DCM (0.2 mL) and *n*-pentane was added causing precipitation. The solid was filtered off and the precipitation was repeated to afford the pure product **5dB** as white solid (28.0 mg, 0.094 mmol, 94%, 94% *ee*). The enantiomeric excess was determined by ^{1}H NMR as described in **GP8**.

$C_{16}H_{14}N_2O_4$, ***M*:** 298.29 g/mol. **m.p.:** 158 °C. **$[\alpha]^{20}_D$** : –72.0 (c = 1.0 mg/mL , sample with 94% *ee*). **^{1}H NMR (($(CD_3)_2CO$, 400 MHz, 21 °C):** *δ* (ppm) = 7.97 (*br. s*, 1H, N*H*), 7.37-7.21 (*m*, 5H, Ar*H*), 6.31 (*dd*, *J* = 8.2, 2.3 Hz, 1H, C*H*=CH), 6.18 (*d*, *J* = 8.2 Hz, 1H, CH=C*H*), 4.72 (*m*, 1H, C*H*-O-C=O), 4.67 (*s*, 1H, O*H*), 4.55 (*s*, 2H, C*H*$_2$Ph), 3.71 (*dd*, *J* = 8.1, 4.3 Hz, 1H, O-CH-C*H*-C(=O)N), 3.08 (*d*, *J* = 8.2 Hz, 1H, C*H*-C(OH)-C=O). **^{13}C NMR ($(CD_3)_2CO$, 100 MHz, 21 °C):** *δ* (ppm) = 174.6, 174.2, 173.7, 136.3, 136.2, 130.3, 128.3, 128.1, 127.4, 77.1, 49.1, 48.2, 45.8, 41.7. **IR (solid):** $\tilde{\nu}$ = 3317, 1771, 1431, 1397, 1343, 1297, 1204, 1171, 1068, 923, 739, 699, 485 cm^{-1}. **HRMS (ESI)**: m/z calculated for $[C_{16}H_{14}N_2O_4Na]^+$: 321.0846, found: 321.0841.

(3a*R*,4*R*,7*R*,7a*S*)-4,7-Ethenopyranol [3,4-*c*] pyrran-1,3,6(2*H*)-trione-3a,4,7,7a-tetrahydro-7-hydroxy (7)

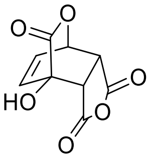

7

The product **7** was synthesized according to **GP4** using 3-hydroxypyrone **1a** (11.2 mg, 0.10 mmol, 1.0 equiv.), maleic anhydride **6** (10.2 mg, 0.105 mmol, 1.05 equiv.) and catalyst **C1a** (4.56 mg, 0.005 mmol, 5.0 mol%) at 0 °C. **7** was isolated as a white solid (19.3 mg, 0.092 mmol, 92%, *ee* = 84%). The enantiomeric excess was determined by ^{1}H NMR as described in **GP8**.

$C_9H_6O_6$, ***M*:** 210.14 g/mol. **m.p.:** 110 °C. **$[\alpha]^{20}_D$:** 29.1 (c = 0.90 mg/mL, acetonitrile, sample with 84% *ee*). **^{1}H NMR (CD_3CN, 400 MHz, 21 °C):** *δ* (ppm) = 6.67-6.56 (*m*, 2H, C*H*=C*H*), 5.62 (*dt*, *J* = 4.8 Hz, 2.2 Hz, 1H, C*H*-O-C=O), 4.86 (*br. s*, 1H, O*H*), 4.20 (*dd*, *J* = 8.2, 4.8 Hz, 1H, O-CH-C*H*-C(=O)N), 3.58 (*d*, *J* = 8.5 Hz, 1H, C*H*-C(OH)-C=O). **^{13}C NMR (CD_3CN, 176 MHz, 21 °C):** *δ* (ppm) = 171.6, 168.5, 168.0, 138.1, 130.5, 75.2, 71.7, 48.1, 45.2. **IR (solid):** $\tilde{\nu}$ = 3424, 1769, 1364, 1234, 1149, 1063, 1013, 965, 926, 697 cm^{-1}. **MS (ESI) *m/z*:** 211.0 (M$^+$), 166.0 (M–CO_2), 122.0 (M–CO_2–CO_2), 94.0 (M–CO_2–CO_2–CO).

Ethyl (3a*R*,4*S*,7*R*,7a*R*)-4,7-Methano-1*H*-isoindole-(2*H*,7*H*)-1,3,5-trione, tetrahydro- 2-(phenylmethyl)-4-carboxylate (9)

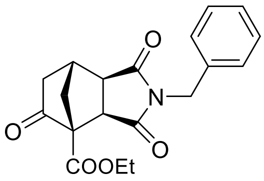

9

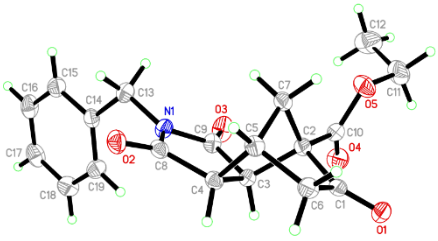

The product **9** was synthesized according to **GP7** using enone **8** (18.4 mg, 0.12 mmol, 1.2 equiv.), *N*-benzylmaleimide **2B** (18.7 mg, 0.10 mmol, 1.0 equiv.) and catalyst **C1a** (0.45 mg, 0.0005 mmol, 0.50 mol%) at –20 °C. **9** was isolated as a white solid (31.7 mg, 0.093 mmol, 93%, *ee* = 98%). The enantiomeric excess was determined by ^{1}H NMR as described in **GP8**.

$C_{19}H_{19}NO_5$, ***M*:** 341.36 g/mol. **$[\alpha]^{20}_{D}$:** 63.7 (c = 1.0 mg/mL, acetone, sample with 98% *ee*). **^{1}H NMR ($CDCl_3$, 400 MHz, 21 °C):** δ (ppm) = 7.33-7.20 (*m*, 5H, Ar*H*), 4.59 (*q*, *J* = 27.6 Hz, 14.0 Hz, 2H, CH_2Ph), 4.27 (*m*, 2H, OCH_2CH$_3$), 3.17 (*d*, *J* = 7.4 Hz, 1H, O=C-C*H*CH-C=O), 2.97 (*m*, 1H, C*H*(CH$_2$)), 2.86 (*d*, *J* = 7.4 Hz, 1H, O=C-CHC*H*-C=O), 2.35 (*dd*, *J* = 18.7, 4.7 Hz, 1H, C*H*HC=O), 2.10 (*dd*, *J* = 18.0, 4.0 Hz, 1H, CH*H*C=O), 1.93 (*dd*, *J* = 12.1, 1.2 Hz, 1H, CH_2), 1.60 (*m*, 1H CH_2), 1.29 (*t*, *J* = 7.4 Hz, 3H, OCH$_2$CH_3). **^{13}C NMR ($CDCl_3$ 176 MHz, 21 °C):** δ (ppm) = 204.9, 175.8, 173.5, 166.3, 135.4, 128.9, 128.8, 128.3, 65.2, 61.8, 48.0, 44.9, 43.8, 43.0, 36.6, 34.3, 14.2. **IR (solid):** $\tilde{\nu}$ = 2956, 2917, 1762, 1704, 1543, 1462, 1394, 1319, 1260, 1174, 1097, 1018, 800, 733, 701 cm^{-1}. **HRMS (ESI):** m/z calculated for $[C_{19}H_{19}NO_5Na]^+$: 364.1155, found: 364.1162.

CCDC 2002396 contains the supplementary crystallographic data for compound **9**. These data can be obtained free of charge from the Cambridge Crystallographic Data Centre via www.ccdc.cam.ac.uk/data_request/cif.

Ethyl (1*S*,2*S*,3*S*,4*R*)-3-Nitro-6-oxo-2-phenylbicyclo[2.2.1]heptane-1-carboxylate (11)

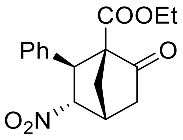

11

The product **11** was synthesized according to **GP7** using enone **8** (18.4 mg, 0.12 mmol, 1.2 equiv.), *β*-nitrostyrene **10** (14.9 mg, 0.10 mmol, 1.0 equiv.) and catalyst **C1a** (0.023 mg, 0.000025 mmol, 0.025 mol%). **11** was isolated as a white oily solid (27.9 mg, 0.092 mmol, 92%, *ee* = 98%). The enantiomeric excess was determined by HPLC with a Chiralcel OD-H column (*n*-heptane/*i*-propanol = 90/10, 0.6 mL/min, detection at 220 nm, *t* (minor) = 48.0 min, *t* (major) = 66.5 min.

$C_{16}H_{17}NO_5$, ***M*:** 303.31 g/mol. **$[\alpha]^{20}_D$:** 56.9 (c = 1.0 mg/mL, acetone, sample with 98% *ee*). **^1H NMR ($CDCl_3$, 400 MHz, 21 °C):** δ (ppm) = 7.28-7.22 (*m*, 3H, Ar*H*), 7.19-7.16 (*m*, 2H, Ar*H*), 5.08 (*dt*, *J* = 5.1 Hz, 1.4 Hz, 1H, C*H*NO_2), 4.00 (*dd*, *J* = 5.2, 1.9 Hz, 1H, C*H*Ph), 3.89 (*q*, *J* = 7.5 Hz, 2H, OC$H_2$$CH_3$), 3.36 (*m*, 1H, ($CH_2$)C*H*CH$NO_2$), 2.68 (*m*, 1H, COC*H*H), 2.45-2.26 (*m*, 3H, COCH*H*, (CH_2)), 0.82 (*t*, *J* = 7.4 Hz, 3H, OCH_2CH_3). **^{13}C NMR (CD_3CN 176 MHz, 21 °C):** δ (ppm) = 204.1, 166.9, 137.8, 129.1, 128.3, 127.9, 93.1, 68.4, 61.3, 47.3, 38.8, 38.4, 37.8, 13.6. **IR (solid):** $\tilde{\nu}$ = 2982, 1761, 1725, 1547, 1467, 1370, 1315, 1260, 1098, 1030, 1012, 984, 756, 701 cm^{-1}. **HRMS (ESI):** m/z calculated for $[C_{16}H_{17}NO_5Na]^+$: 326.0999, found: 326.0994.

Confirming the Configurational Outcome with 2D-NMR Experiments of Compound 11.

To confirm the relative configuration of compound **11**, several 2D-NMR-Experiments were performed (COSY, HSQC, HMBC and NOESY).

COSY-Experiment

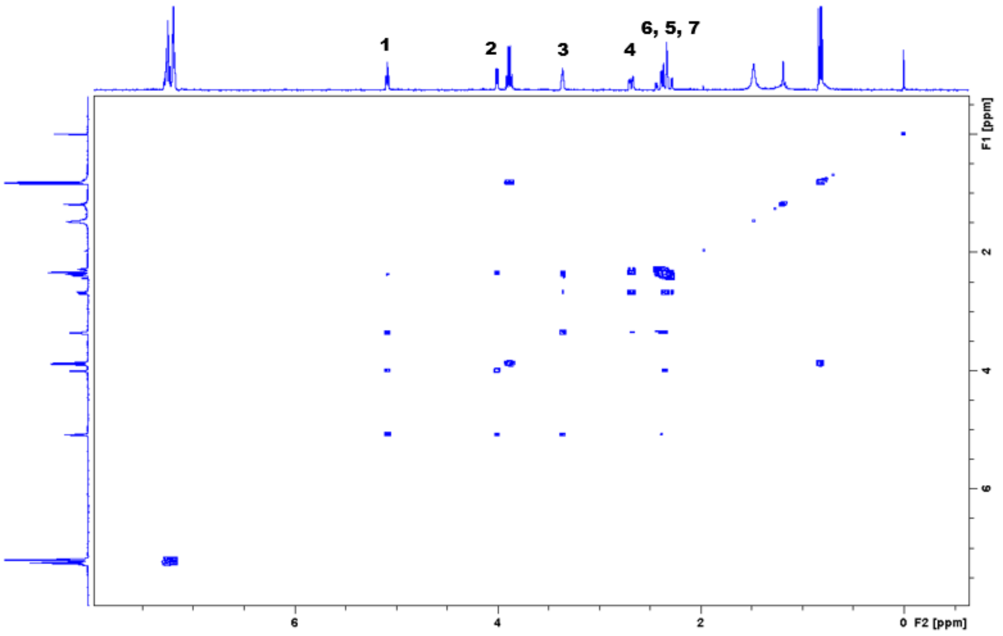

HSQC-Experiment

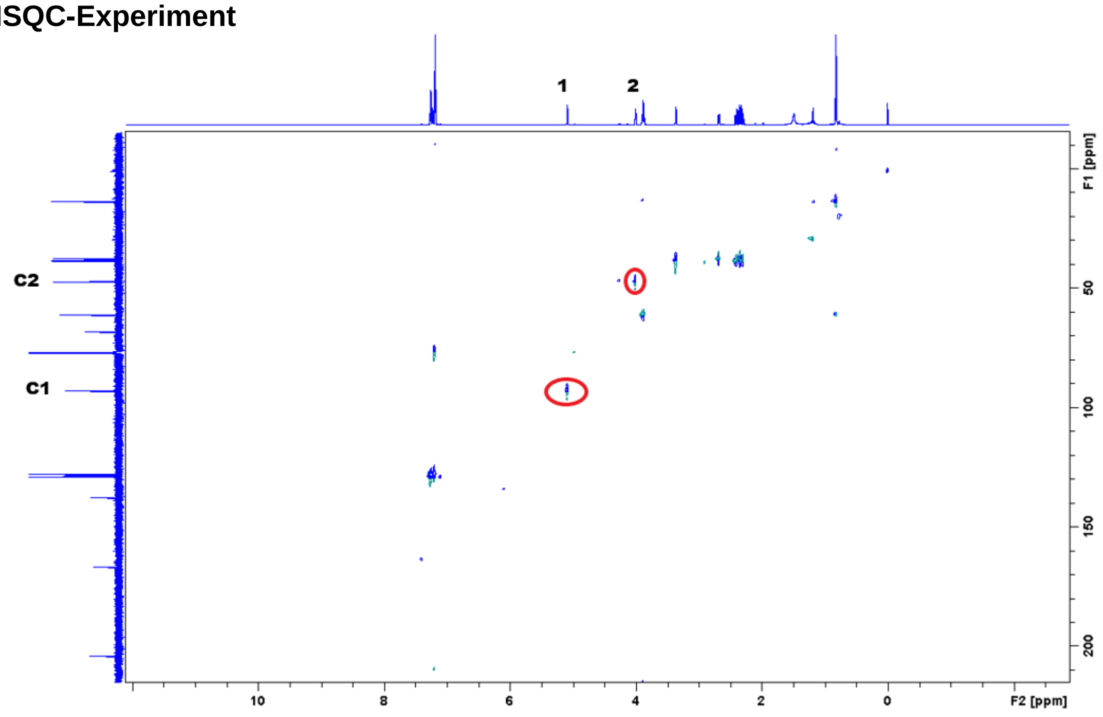

HMBC-Experiment

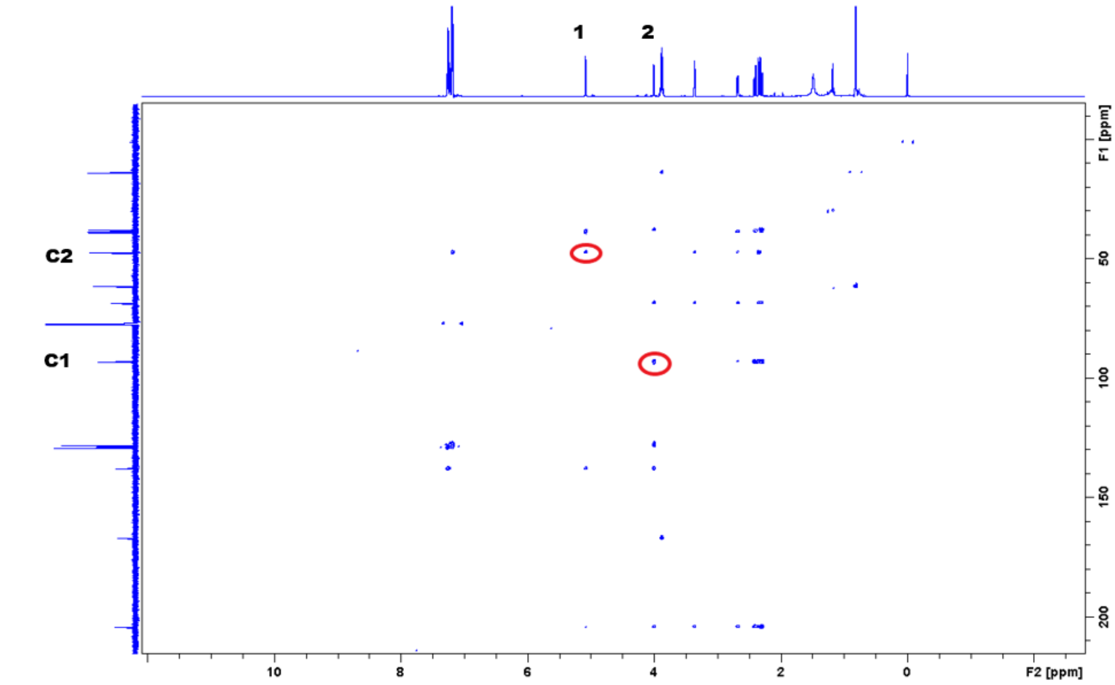

NOESY-Experiment

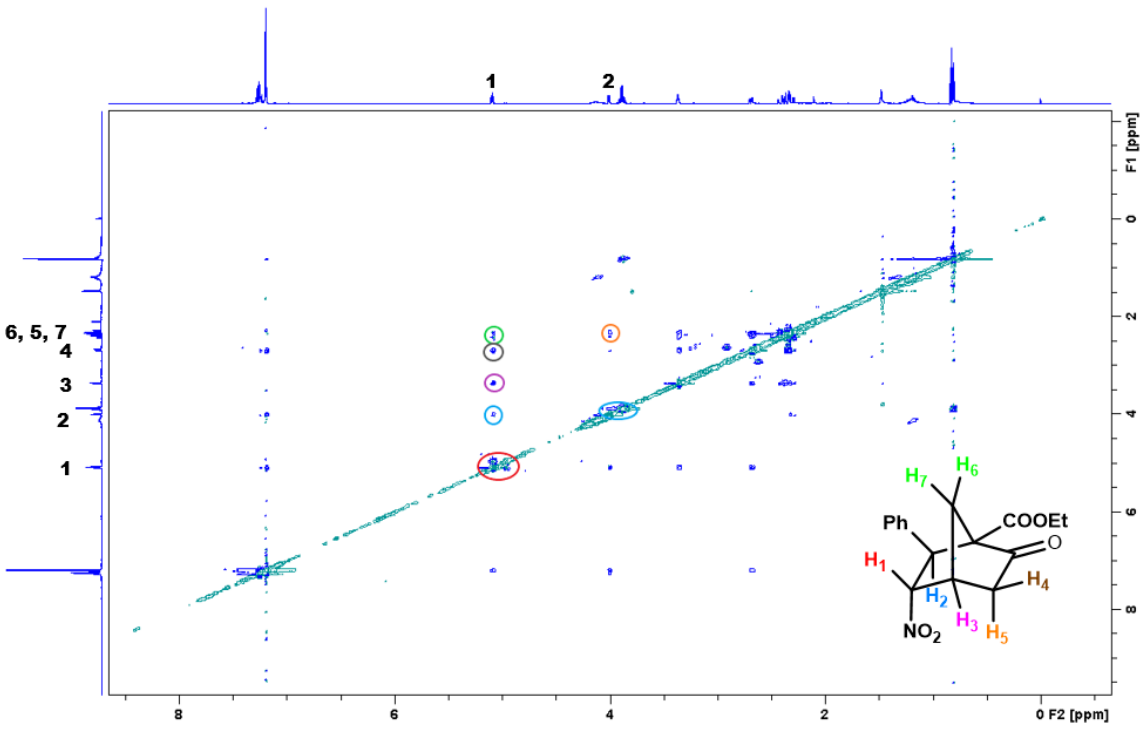

Derivatisation of the Catalytic Product 11.

Ethyl (1*S*,2*S*,3*S*,4*R*,6*R*)-3-Nitro-6-hydroxy-2-phenylbicyclo[2.2.1]heptane-1-carboxylate (11a)

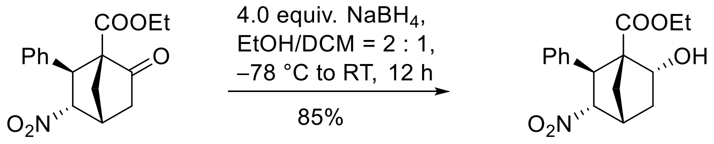

11 **11a**

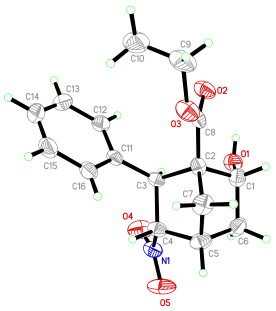

11 (30.3 mg, 0.10 mmol, 1.0 equiv., *ee* = 98%) was dissolved in ethanol/dichloromethane (2.0 mL / 1 mL) and cooled to –78 °C under nitrogen atmosphere. $NaBH_4$ (15.1 mg, 0.40 mmol, 4.0 equiv.) was added in portions over the five minutes at –78 °C. Reaction mixture was allowed to slowly warm to room temperature and stirred for 12 h. Saturated aqueous ammonium chloride (5 mL) was added and reaction mixture was extracted with EE (3 x 10 mL). The combined organic phases were dried over Na_2SO_4 and the solvent was removed under reduced pressure. The crude product was dissolved in a small amount of DCM (0.5 mL) and added to *n*-pentane solution (5 mL) causing the precipitation. The solid was filtered, dried under high pressure to yield the pure **11a** (25.9 mg, 0.085 mmol, 85%).

$C_{16}H_{19}NO_5$, ***M*:** 305.33 g/mol. **m.p.:** 140-141 °C **$[\alpha]^{20}_D$:** 36.1. (c = 1.0 mg/mL, acetone). **^{1}H NMR ($CDCl_3$, 400 MHz, 21 °C):** δ (ppm) = 7.34-7.26 (*m*, 5H, Ar*H*), 5.05 (*dt*, *J* = 5.3 Hz, 1.8 Hz, 1H, C*H*NO_2), 4.63 (*dd*, *J* = 6.4, 2.3 Hz, 1H, C*H*Ph), 4.40 (*dd*, *J* = 10.5, 4.2 Hz, 1H, C*H*OH), 3.78 (*m*, 2H, OC*H*$_2$CH_3), 3.00 (*m*, 1H, (CH_2)C*H*CHNO_2), 2.80 (*br. s*, 1H, O*H*), 2.36 (*dd*, *J* = 11.5, 3.4 Hz, 1H, (C*H*$_2$)COOEt), 2.19 (*m*, 1H, HO-CC*H*H), 1.93 (*dt*, *J* = 11.8, 2.0 Hz, 1H, (C*H*$_2$)COOEt), 1.35 (*dt*, *J* = 14.3, 4.1 Hz, 1H, HO-CC*H*H), 0.82 (*t*, *J* = 7.3 Hz, 3H, OCH_2C*H*$_3$). **^{13}C NMR ($CDCl_3$, 176 MHz, 21 °C):** δ (ppm) = 172.1, 139.0, 128.7, 128.5, 127.7, 93.3, 73.0, 61.8, 60.8, 43.0, 40.3, 36.9, 30.8, 13.6. **IR (solid):** $\tilde{\nu}$ = 3564, 2982, 1708, 1542, 1455, 1372, 1340, 1316, 1259, 1100, 1081, 1057, 756, 700, 605 cm^{-1}. **HRMS (ESI):** m/z calculated for $[C_{16}H_{19}NO_5Na]^+$: 328.1155 found: 328.1151.

CCDC 1998669 contains the supplementary crystallographic data for compound **11a**. These data can be obtained free of charge from the Cambridge Crystallographic Data Centre via www.ccdc.cam.ac.uk/data_request/cif.

Catalyst Recycling

The catalytic reaction was done applying **GP6**. The reaction mixture was filtered over silica gel with PE / EE = 1:1 as eluent. The catalyst **C1b** turned green and was sticking on the top. After elution with 20 mL of the solvent mixture, residual solvent was then largely pressed through the silica pad (to dryness). By using a solvent mixture of DCM/THF/NEt_3 (66/33/1) the catalyst turned brown again and the activated catalyst was flashed down from the silica and collected using approximately 10 mL of the solvent mixture. The solvent was removed under reduced pressure, the resulting brown solid was dissolved in a small amount of DCM and precipitated by addition of *n*-pentane (app. 5 mL). The precipitate was filtered off, and the activated catalyst was used in the next catalytic reaction without further purification as described in **GP6**.

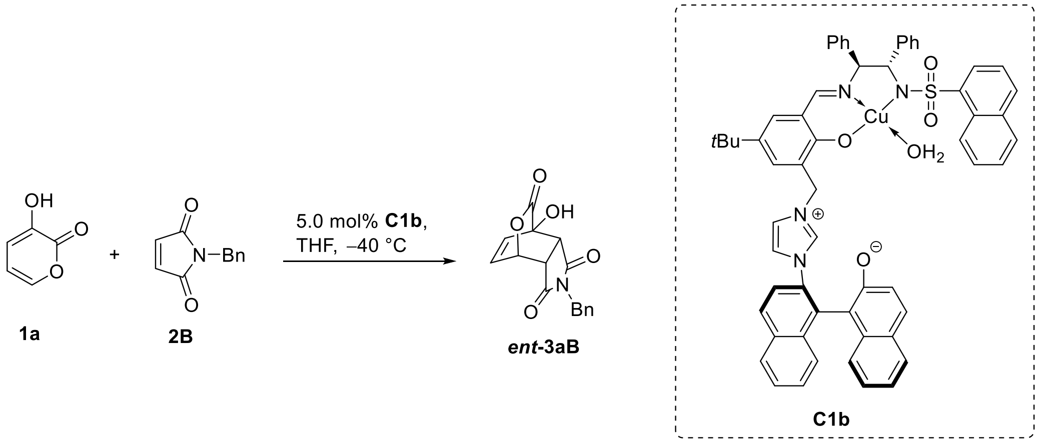

Run[a]	yield (%)[b]	dr[c]	ee[d] / [%]
**1**	94	>98:2	98
**2**	92	>98:2	98
**3**	92	>98:2	96
**4**	91	>98:2	93
**5**	90	>98:2	91

[a] Reactions were performed on 0.1 mmol scale. [b] Yield of isolated product. [c] *Endo*/*exo* ratios determined by ^1H NMR using the crude product. [d] The enantiomeric excess was determined by ^1H NMR using saturated $CDCl_3$ solution of (*R*)-(+)-binaphthol.[7]

Mechanistic Study

Mass Spectrometric Experiments

The activated catalyst **C1b** (0.51 mg, 0.00052 mmol, 1.0 equiv.) and hydroxypyrone **1a** (0.11 mg, 0.001 mmol, 2.0 equiv.) were dissolved in THF (0.5 mL). The reaction sample was measured by ESI-MS after 15 min. A Cu-dienolate species was detected as shown by the following spectra.

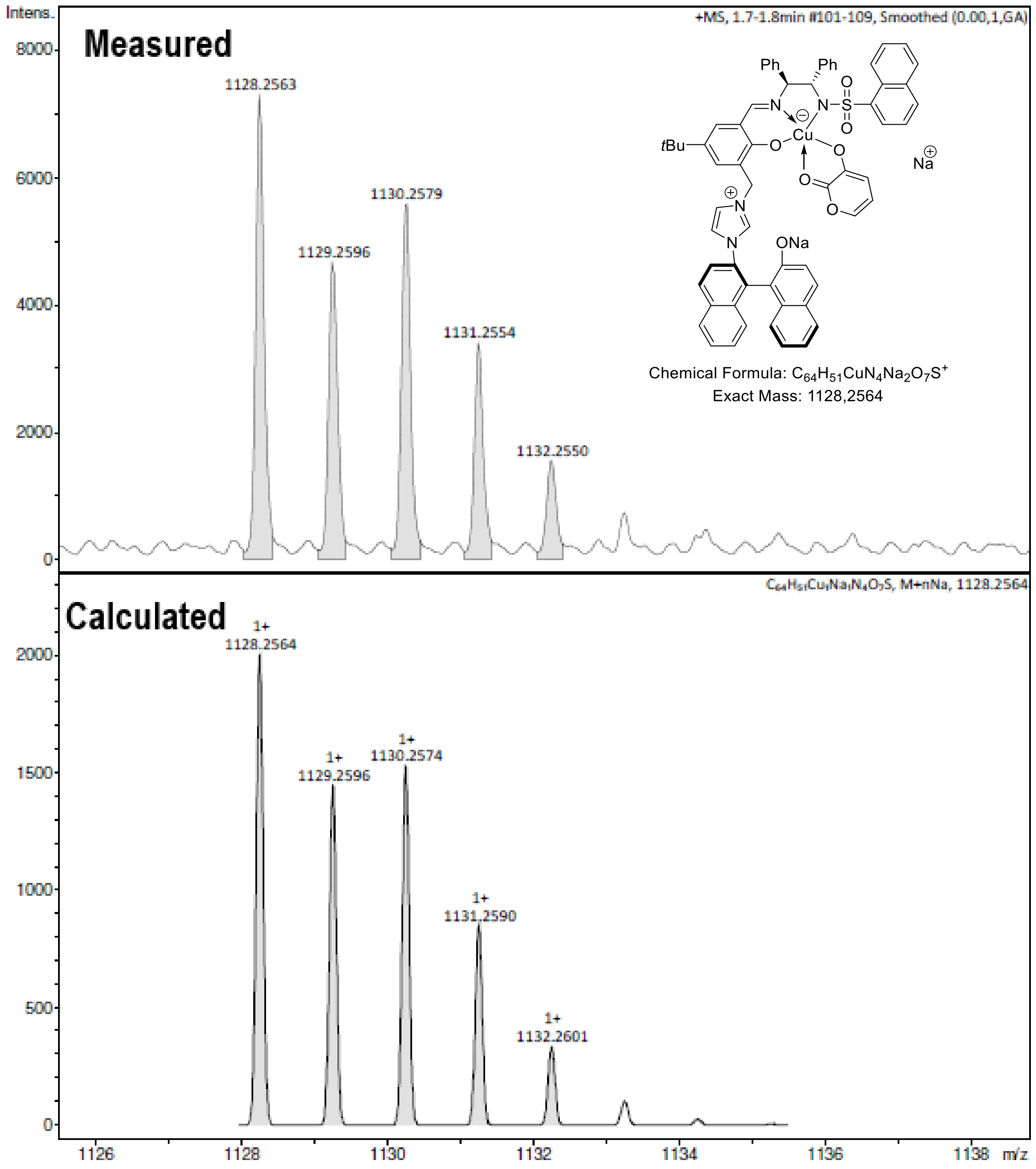

^{1}H NMR Titration Experiments

Catalyst **C1b** (0.51 mg, 0.00052 mmol, 1.0 equiv.) was dissolved in THF-d_8 (0.2 mL) and filled in the NMR-tube. The hydroxypyrone **1a** (0, 1.0, 2.0 and 10.0 equiv.) was added directly to the tube and after shaking for one minute at –20 ºC the ^{1}H NMR spectra was recorded (**Figure S1**).

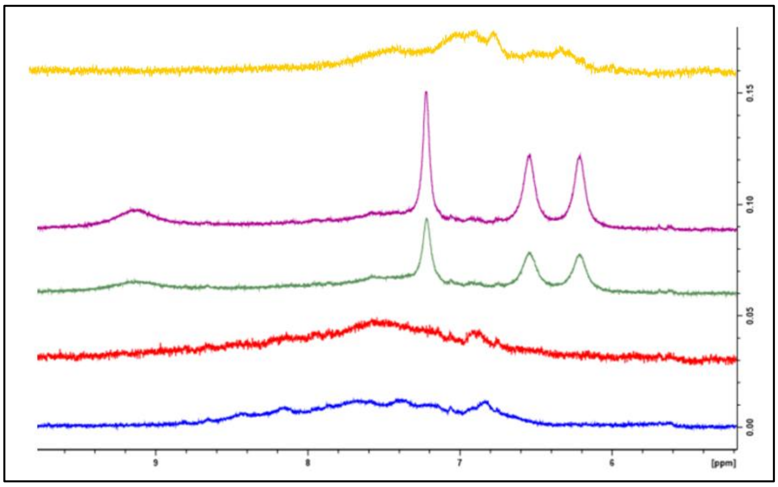

Figure S1. ^{1}H-NMR titration experiments. The blue curve shows the spectrum of **C1b** in THF-d_8 at –20 °C. For the red curve, 1.0 equiv. of **1a** were added. For the green and purple curve, 2.0, and 10.0 equiv. of **1a** were added, respectively. The yellow curve shows the spectra of pre-catalyst of **C1b**.

UV-Vis Titration Experiments

UV-Vis titration experiments were performed in which the betaine catalyst **C1b** was treated with 3-hydroxy-2-pyrone **1a**. Catalyst **C1b** (0.25 mg, 0.00025mmol, 1.0 equiv.) was dissolved in THF (1.0 mL) and filled in a cuvette (d = 10mm, Quartz SUPRASIL®). **1a** (0.2-10.0 equiv.) was added directly to the cuvette and after shaking, the UV-Vis spectra were measured at room temperature (**Figure S2**).

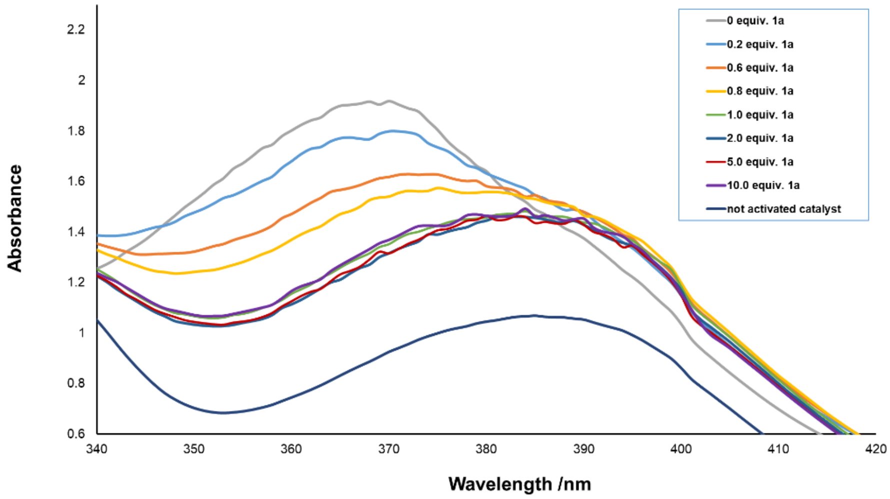

Figure S2. UV-Vis spectra of the titration experiment using **1a.**

Kinetic Experiments

Probing Catalyst Robustness and Product Influence

Blackmond's reaction progress kinetic analysis (RPKA) was performed using ^{1}H-NMR spectroscopy for monitoring the complete course of the catalytic reaction. By the so-called "same excess" protocol, the catalyst robustness and a possible product inhibition under the reaction conditions was assessed.[14] The model reaction of **1a** and **2B** was examined at –20 °C in THF-d_8 using 3.0 mol% of **C1b**. The experiments were performed starting from three different points. The different initial concentrations for the reactants **1a** and **2B** and the catalyst for the kinetic experiments are summarized in **Table S1**.

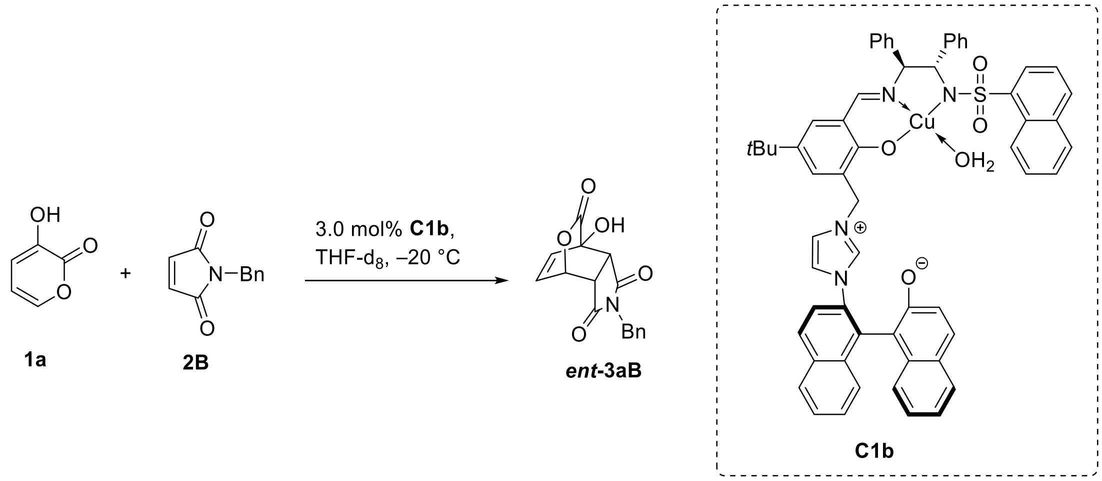

Table S1: Different initial concentrations of hydroxypyrone **1a**, maleimide **2B** and product **3aB** in "same-excess"-experiments and "product addition" for investigation of possible product inhibition and catalyst stability.

Experiment	Description	[**1a**] / mol/L	Equiv. of **1a**	[**2B**] / mol/L	Equiv. of **2B**	[**3aB**] / mol/L	Equiv. of **3aB**	[**C1b**] / mol/L	Equiv. of **C1b**
**B1**	reference	0.04	1.00	0.042	1.05	0.00	0.00	0.0012	0.03
**B2**	same excess	0.02	0.50	0.022	0.55	0.00	0.00	0.0012	0.03
**B3**	product addition	0.02	0.50	0.022	0.55	0.02	0.50	0.0012	0.03

The reactions were performed by adding a solution of the catalyst **C1b**, 1,2-diphenylethane (internal standard, 0.02 mmol), hydroxypyrone **1a**, maleimide **2B** in tetrahydrofuran-d_8 (0.5 mL) to an NMR sample tube. The reaction mixture was analysed by ^{1}H NMR spectroscopy at −20 °C to monitor the conversion of **2B** in dependence of time. First, the progress of a reference reaction was monitored with the indicated initial substrate concentrations (**Table 1**, **Experiment B1**). The second experiment was done with the initial substrate concentrations which were equal to those of the reference reaction when 50% conversion was reached (**Experiment B2**). The third measurement was done with the same initial conditions like in the second reaction, but with the addition of product **3aB** (**Experiment B3**). These experiments could provide information of either catalyst deactivation or product inhibition. The difference between the reactions **B2** and **B3** compared to **B1** is that when the latter reaction reached 50% of conversion, the catalyst was not fresh anymore because it has undergone a number of turnovers already, whereas in **B2** and **B3** the catalyst is fresh at the starting point. **Figure S3** presents a comparison of kinetic profiles of these three reactions. Time adjustment was done by simple shifting the data of reactions **B2** and **B3** to the point where the concentrations

are the same as for reference reaction **B1**. Since the reaction progress from this point onward is almost identical as the time shift of the **B2** curve shows, it appears that no significant catalyst decomposition occurs during the reaction. Additionally, an overlay of the reaction profiles **B3** with **B1** was found demonstrating that the product **3aB** does not inhibit the catalyst.

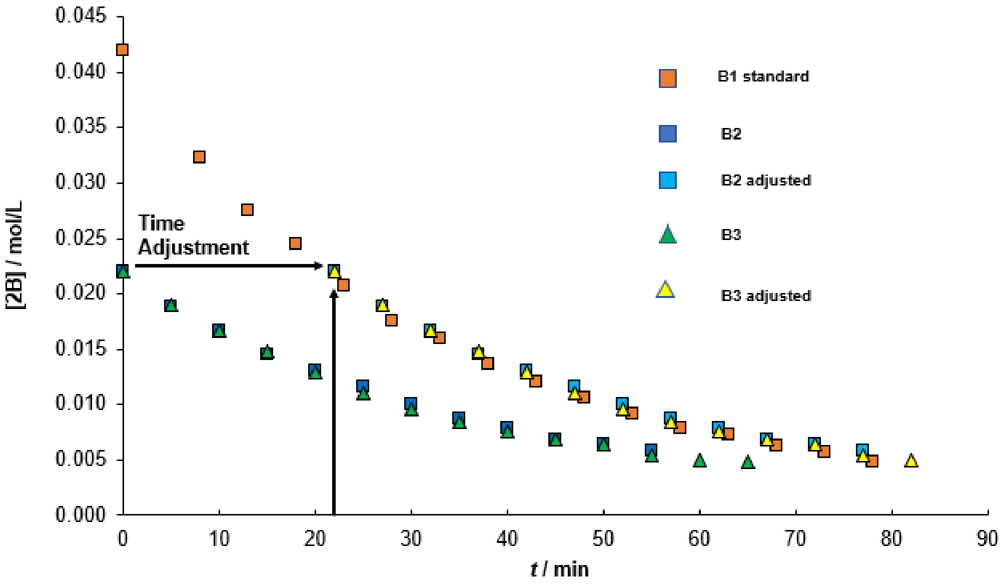

Figure S3. Probing catalyst stability and product influence on the catalytic reaction.

Raw Data and Calculated Concentrations for the "Same-Excess"-and "Product Addition"-Experiments

Table S2. Raw data of the "same-excess"-**Experiments B1-B3**, calculated concentration of maleimide **2B** and time adjustment

**B1-reference**		**B2-same excess**			**B3-Product addition**		
**Time (min)**	**[2B] mol/L**	**Time /min**	**Time adjustment /min**	**[2B] mol/L**	**Time /min**	**Time adjustment /min**	**[2B] mol/L**
0	0.0420	0	22	0.0220	0	22	0.0220
10	0.0323	10	27	0.0189	10	27	0.0190
15	0.0276	15	32	0.0167	15	32	0.0166
20	0.0245	20	37	0.0145	20	37	0.0147
25	0.0207	25	42	0.0131	25	42	0.0129
30	0.0175	30	47	0.0116	30	47	0.0110
35	0.0160	35	52	0.0101	35	52	0.0096
40	0.0136	40	57	0.0088	40	57	0.0085
45	0.0121	45	62	0.0078	45	62	0.0076
50	0.0106	50	67	0.0069	50	67	0.0068
55	0.0091	55	72	0.0064	55	72	0.0065
60	0.0078	60	77	0.0058	60	77	0.0054
65	0.0074	65	82	0.0056	65	82	0.0049
70	0.0062	70	87	0.0052	70	87	0.0048
75	0.0057	75	97	0.0047	75		
80	0.0048	80	102	0.0043	80		

Kinetic Experiments–Determination of Reaction Orders Using Variable Time Normalization Graphical Analysis (VTNA)

The orders of all reaction components were determined using the variable time normalization graphical analysis method (VTNA) described by Burés.[15,16] Four reactions **E1-E4** with different initial concentrations of each component, catalyst **C1b**, hydroxypyrone **1a** and maleimide **2B**, were performed and monitored via ^{1}H NMR. The different initial concentrations for the components used in the kinetic experiments are summarized in the **Table S3**.

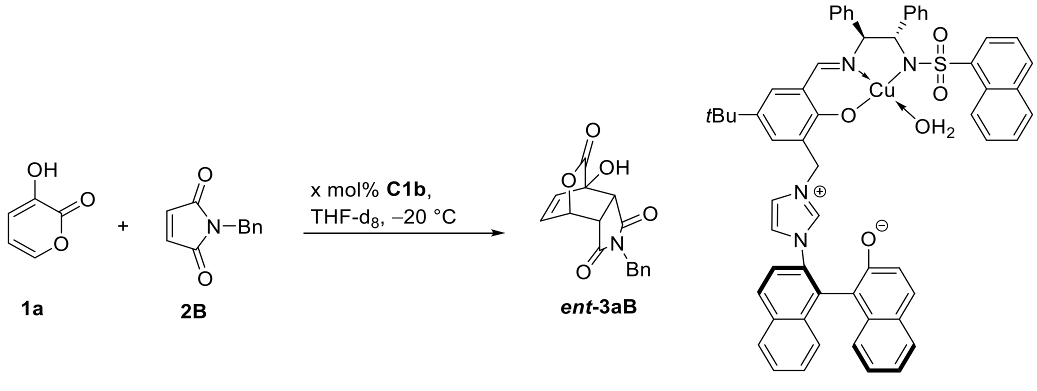

Table S3: Variation of the initial concentrations of catalyst **C1b**, hydroxypyrone **1a** and maleimide **2B.**

Experiment	Description	[**1a**] / mol/L	[**2B**] / mol/L	[**C1b**] / mol/L
**E1**	reference	0.04	0.042	0.0012
**E2**	diff. [**C1b**]	0.04	0.042	0.0016
**E3**	diff. [**1a**]	0.08	0.042	0.0012
**E4**	diff. [**2B**]	0.04	0.024	0.0012

The corresponding experiments **E1-E4** were performed by adding a solution of the catalyst **C1b**, 1,2-diphenylethane (internal standard, 0.02 mmol), hydroxypyrone **1a**, maleimide **2B** in THF-d$_8$ (0.5 mL) to an NMR sample tube at –20 °C as shown in **Table S3**. The reaction mixture was analysed by ^{1}H NMR spectroscopy at –20 °C to monitor the conversion of **1a** and **2B** and the yield of **3aB** in dependence of time (**Figure S4**).

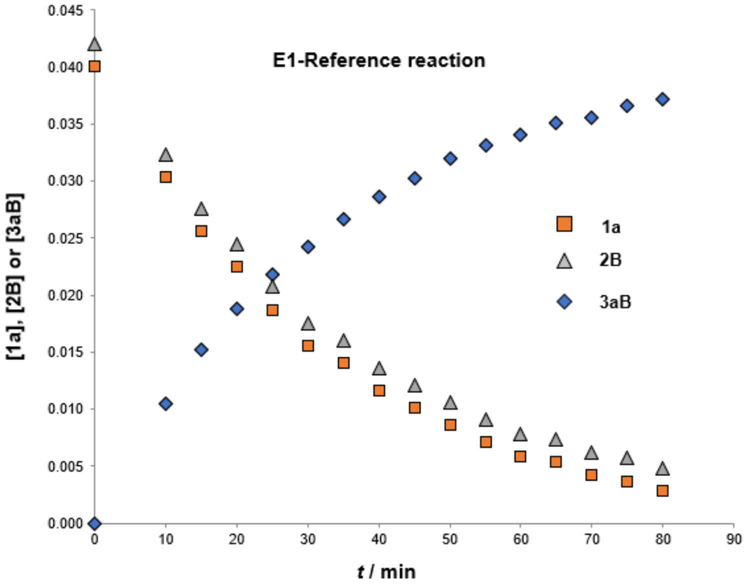

Figure S4. Conversion of **1a** and **2B** and the yield of **3aB** in dependence of time.

The reaction progress profiles of all four experiments **E1-E4** are plotted in **Figure S5** and were investigated using VTNA. The order in each component can be determined by systematically changing each exponent of the normalized time axis, with the intention to obtain a linear overlay of all reaction profiles in the plot.

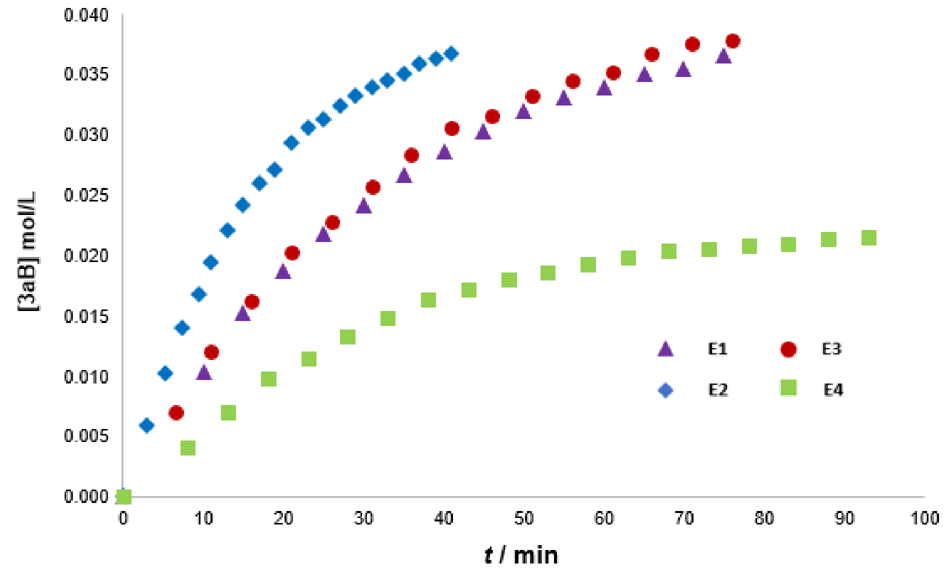

Figure S5. Original reaction progress profile of the formation of product **3aB** for the four reactions.

The best fit for the normalization of the time scale axis was achieved for partial orders of 0.07 for 3-hydroxy-2-pyrone **1a**, 1.00 for *N*-Benzylmaleimide **2B** and 1.95 for catalyst **C1b**. The empirical rate law under the mentioned reaction conditions is thus:

$$r = k *[\mathbf{C1b}]^{1.95} * [\mathbf{1a}]^{0.07} * [\mathbf{2B}]^{1.00}$$

When the normalization is applied to all the components, the result is a plot with a straight line with a slope equal to k_{obs}. The slope, k_{obs} of the reaction and was found to be k_{obs}= $1.8 \cdot 10^4 L^{2.02} mol^{-2.02} s^{-1}$. The plot of this time normalized reaction profiles is shown in **Figure S6**.

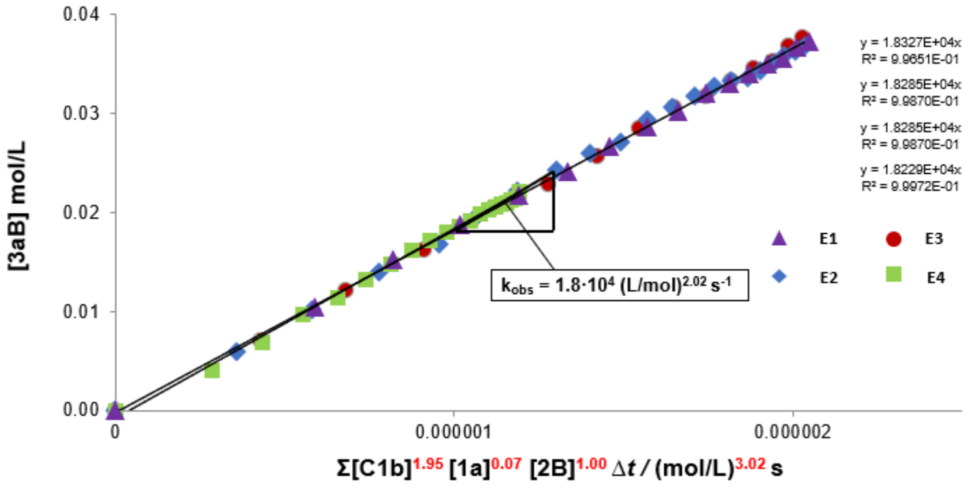

Figure S6. Best overlay of all four reaction progress profiles with orders **1.95** in **C1b**, **0.07** in **1a** and **1.00** in **2B**. Unit of x-axis in mol/(L·s) **(k_{obs}= $1.8 \cdot 10^4 L^{2.02} mol^{-2.02} s^{-1}$).**

Raw Data, Calculated Concentrations and Processed Data for the VTNA.

All processed data are given in α = 1.95, β = 0.07, γ = 1.00.

E1, reference reaction

Time (min)	[C1b] mol/L	[1a] mol/L	[2B] mol/L	[3aB] mol/L	$\sum[C1b]^{\alpha}[1a]^{\beta}[2B]^{\gamma}\Delta t$
0	0.0012	0.0400	0.0420	0.0000	0.0000
10	0.0012	0.0303	0.0323	0.0104	5.8643E-07
15	0.0012	0.0256	0.0276	0.0152	8.1902E-07
20	0.0012	0.0227	0.0245	0.0188	1.0189E-06
25	0.0012	0.0187	0.0207	0.0218	1.1906E-06
30	0.0012	0.0155	0.0175	0.0242	1.3341E-06
35	0.0012	0.0140	0.0160	0.0267	1.4585E-06
40	0.0012	0.0116	0.0136	0.0287	1.5673E-06
45	0.0012	0.0101	0.0121	0.0303	1.6608E-06
50	0.0012	0.0085	0.0106	0.0320	1.7423E-06
55	0.0012	0.0071	0.0091	0.0331	1.8121E-06
60	0.0012	0.0058	0.0078	0.0340	1.8714E-06
65	0.0012	0.0054	0.0074	0.0350	1.9241E-06
70	0.0012	0.0042	0.0062	0.0356	1.9706E-06
75	0.0012	0.0037	0.0057	0.0366	2.0109E-06
80	0.0012	0.0028	0.0048	0.0372	2.0460E-06

[**1a**] was calculated using the Blackmond's "excess" method. ([**1a**] = [**2B**]–[e]). [e] is defined as the difference of concentration of the two reactants and remains constant during the reaction.

E2, Difference in [C1b]

Time (min)	[C1b] mol/L	[1a] mol/L	[2B] mol/L	[3aB] mol/L	$\sum[C1b]^{\alpha}[1a]^{\beta}[2B]^{\gamma}\Delta t$
0	0.0016	0.0400	0.0420	0.0000	0.00000
3	0.0016	0.0345	0.0359	0.0060	3.5734E-07
5	0.0016	0.0305	0.0319	0.0102	5.7983E-07
7	0.0016	0.0277	0.0290	0.0141	7.7805E-07
9	0.0016	0.0242	0.0255	0.0168	9.5412E-07
11	0.0016	0.0215	0.0227	0.0195	1.0610E-06
13	0.0016	0.0193	0.0206	0.0221	1.1879E-06
15	0.0016	0.0166	0.0178	0.0243	1.2994E-06
17	0.0016	0.0154	0.0167	0.0260	1.3988E-06
19	0.0016	0.0137	0.0149	0.0271	1.4892E-06
21	0.0016	0.0122	0.0134	0.0294	1.5695E-06
23	0.0016	0.0111	0.0123	0.0307	1.6417E-06
25	0.0016	0.0099	0.0111	0.0314	1.7072E-06
27	0.0016	0.0086	0.0097	0.0325	1.7651E-06
29	0.0016	0.0078	0.0090	0.0333	1.8166E-06
31	0.0016	0.0070	0.0081	0.0340	1.8633E-06
33	0.0016	0.0059	0.0071	0.0345	1.9045E-06
35	0.0016	0.0054	0.0065	0.0351	1.9409E-06
37	0.0016	0.0050	0.0061	0.0359	1.9744E-06
39	0.0016	0.0045	0.0056	0.0363	2.0055E-06
41	0.0016	0.0041	0.0052	0.0368	2.0338E-06

[**1a**] was calculated using the Blackmond's "excess" method. ([**1a**] = [**2B**]–[e]). [e] is defined as the difference of concentration of the two reactants and remains constant during the reaction.

E3, Difference in [1a]

Time (min)	[C1b] mol/L	[1a] mol/L	[2B] mol/L	[3aB] mol/L	$\sum[C1b]^{\alpha}[1a]^{\beta}[2B]^{\gamma}\Delta t$
0	0.0012	0.0800	0.0420	0.0000	0.00000
6	0.0012	0.0734	0.0354	0.0071	4.2526E-07
11	0.0012	0.0679	0.0299	0.0121	6.7043E-07
16	0.0012	0.0639	0.0259	0.0162	9.0055E-07
21	0.0012	0.0604	0.0224	0.0203	1.0976E-06
26	0.0012	0.0573	0.0193	0.0229	1.2658E-06
31	0.0012	0.0547	0.0167	0.0257	1.4094E-06
36	0.0012	0.0525	0.0145	0.0285	1.5325E-06
41	0.0012	0.0507	0.0127	0.0306	1.6387E-06
46	0.0012	0.0491	0.0111	0.0317	1.7304E-06
51	0.0012	0.0472	0.0092	0.0333	1.8074E-06
56	0.0012	0.0461	0.0081	0.0345	1.8721E-06
61	0.0012	0.0451	0.0071	0.0353	1.9283E-06
66	0.0012	0.0441	0.0061	0.0368	1.9762E-06
71	0.0012	0.0433	0.0053	0.0376	2.0170E-06
76	0.0012	0.0425	0.0046	0.0379	2.0517E-06

[**1a**] was calculated using the Blackmond's "excess" method. ([**1a**] = [**2B**]–[e]). [e] is defined as the difference of concentration of the two reactants and remains constant during the reaction.

E3, Difference in [2B]

Time (min)	[C1b] mol/L	[1a] mol/L	[2B] mol/L	[3aB] mol/L	$\sum[C1b]^{\alpha}[1a]^{\beta}[2B]^{\gamma}\Delta t$
0	0.0012	0.0400	0.0240	0.0000	0.000000
8	0.0012	0.0357	0.0197	0.0042	2.7933E-07
13	0.0012	0.0326	0.0166	0.0070	4.2351E-07
18	0.0012	0.0302	0.0142	0.0098	5.4506E-07
23	0.0012	0.0277	0.0117	0.0115	6.4694E-07
28	0.0012	0.0260	0.0100	0.0133	7.3212E-07
33	0.0012	0.0244	0.0084	0.0149	8.0393E-07
38	0.0012	0.02377	0.0077	0.0163	8.6636E-07
43	0.0012	0.0225	0.0065	0.0173	9.2115E-07
48	0.0012	0.0215	0.0055	0.0181	9.6747E-07
53	0.0012	0.0206	0.0046	0.0186	1.0063E-06
58	0.0012	0.0201	0.0042	0.0193	1.0398E-06
63	0.0012	0.0195	0.0035	0.0199	1.0690E-06
68	0.0012	0.0188	0.0029	0.0204	1.0933E-06
73	0.0012	0.0184	0.0024	0.0206	1.1136E-06
78	0.0012	0.0181	0.0021	0.0208	1.1309E-06
83	0.0012	0.0178	0.0018	0.0211	1.1457E-06
88	0.0012	0.0177	0.0017	0.0213	1.1590E-06
93	0.0012	0.0176	0.0016	0.0215	1.1717E-06
98	0.0012	0.0174	0.0014	0.0218	1.1832E-06

[**1a**] was calculated using the Blackmond's "excess" method. ([**1a**] = [**2B**]–[e]). [e] is defined as the difference of concentration of the two reactants and remains constant during the reaction.

Investigation of a Possible Non-Linear-Effect

Examination of linear or non-linear effects was done under the conditions described in **GP4** using 5.0 mol% of catalyst **C1b** with six different enantiomeric excesses (**Table S4**), which were prepared by mixing the corresponding amounts of pure enantiomers of the catalysts **C1b**. The plot of *ee*(**3aB**) as a function of *ee*(**C1b**) showed a positive non-linear effect which might be an indication of the relevance of catalyst dimers (**Figure S7**).

Table S4. Enantiomeric excess of the catalyst **C1b** and the product **3aB**.

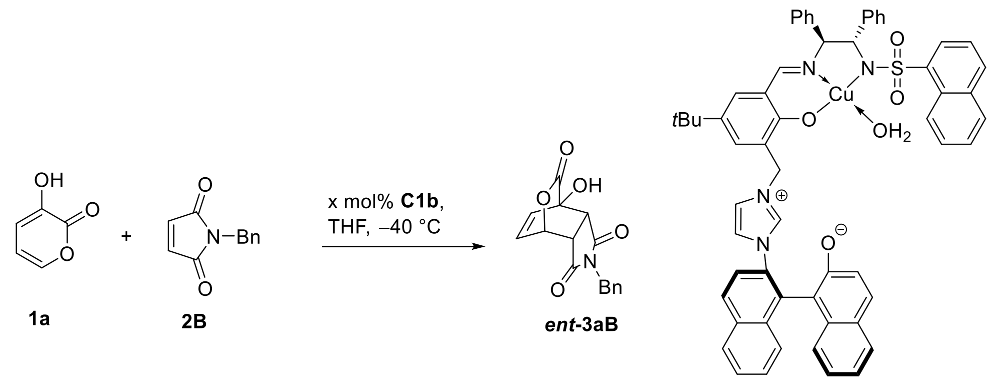

1a **2B** ***ent*-3aB** **C1b**

#	*ee* (C1b)/%	*ee* (3aB)/%
1	100	98
2	80	86
3	60	77
4	40	54
5	20	34
6	0	0

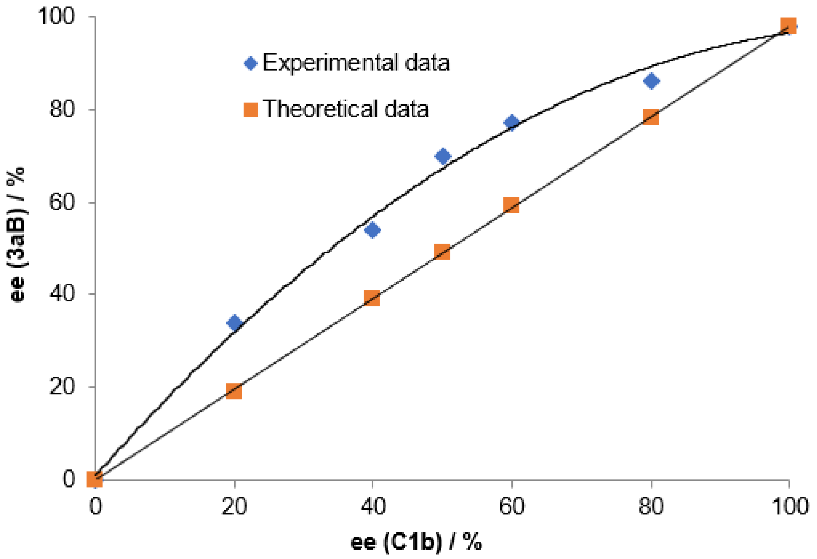

Figure S7: Non-linear effect plot of the catalytic reaction with different *ee*-values of the catalyst **C1b**.

Crystallographic Data

Catalyst C6

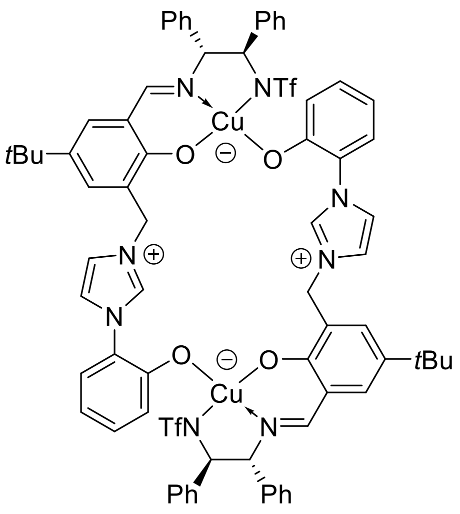

C6

CCDC 1995950 contains the supplementary crystallographic data for compound **C6**. These data can be obtained free of charge from the Cambridge Crystallographic Data Centre via www.ccdc.cam.ac.uk/data_request/cif.

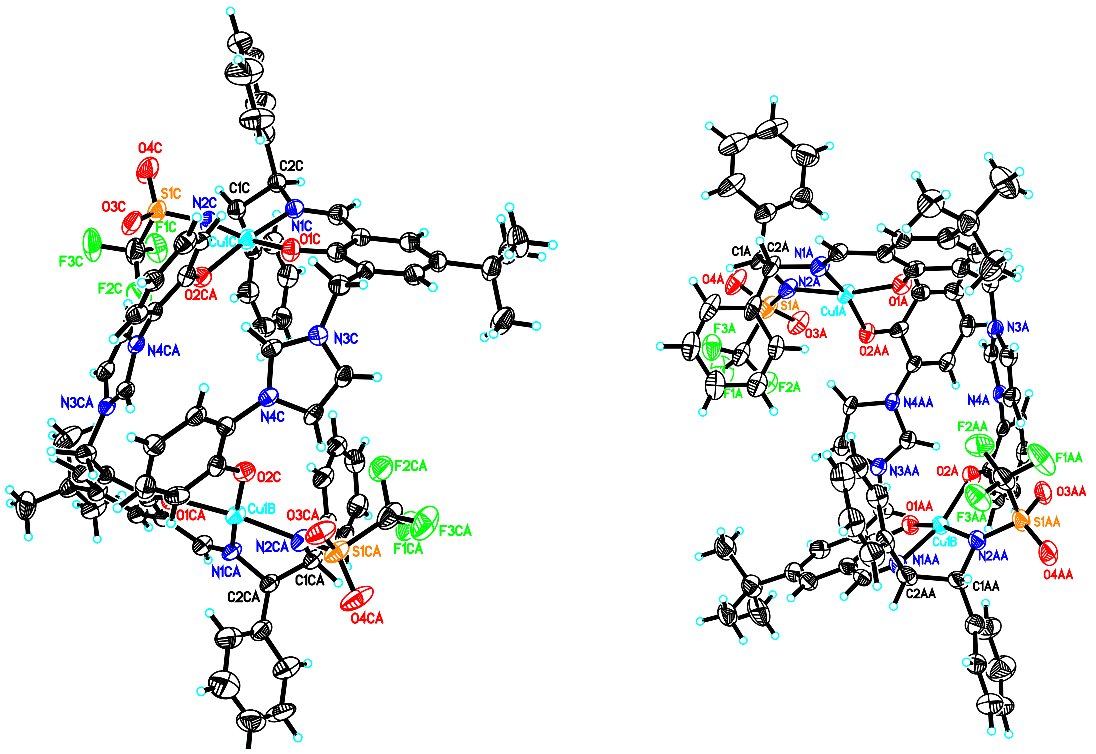

NMR Data

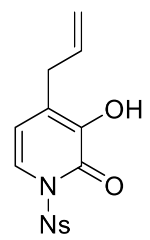

4b

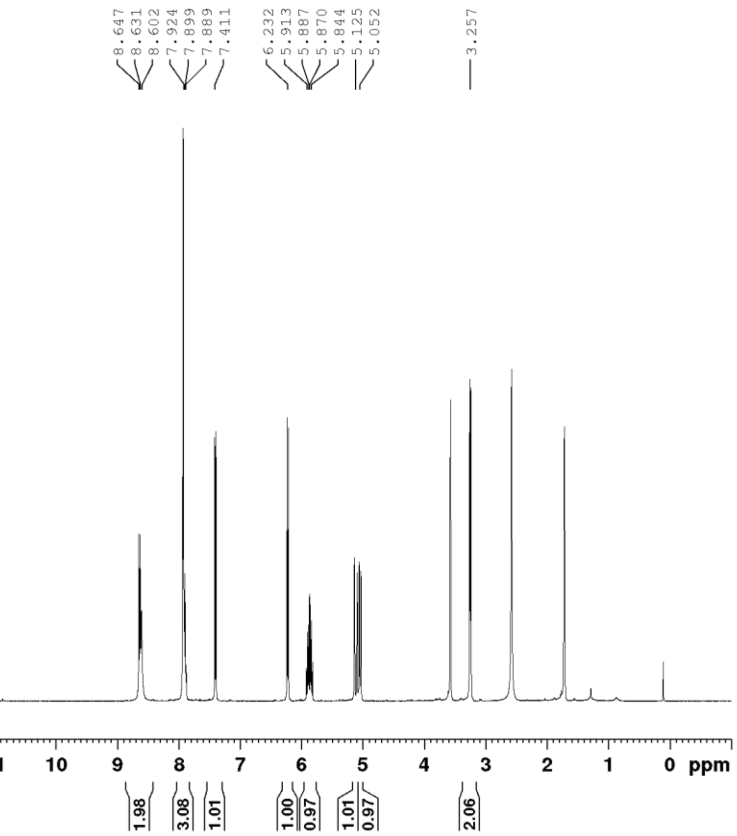


```
Current Data Parameters
NAME         56-2P_NS_ch
EXPNO                200
PROCNO                 1

F2 - Acquisition Parameters
Date_          20190618
Time              11.54
INSTRUM           spect
PROBHD   5 mm PABBO BB/
PULPROG            zg30
TD                65536
SOLVENT             THF
NS                   16
DS                    2
SWH            8012.820 Hz
FIDRES         0.122266 Hz
AQ            4.0894465 sec
RG               205.35
DW               62.400 usec
DE                 6.50 usec
TE                298.0 K
D1           1.00000000 sec
TD0                   1

======== CHANNEL f1 ========
SFO1        400.1024708 MHz
NUC1                 1H
P1                13.70 usec
PLW1        12.00000000 W

F2 - Processing parameters
SI                65536
SF          400.1000158 MHz
WDW                  EM
SSB      0
LB                 0.30 Hz
GB       0
PC                 1.00
```

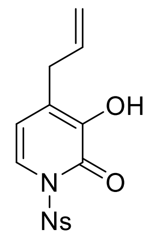

4b

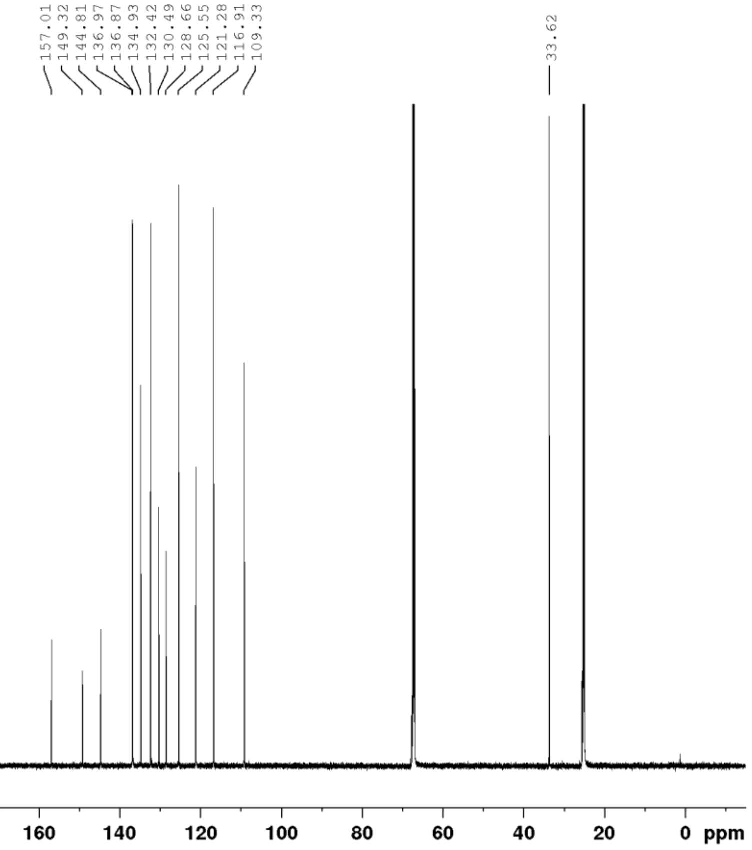


```
Current Data Parameters
NAME         56-2P-allyl
EXPNO                 91
PROCNO                 1

F2 - Acquisition Parameters
Date_          20190619
Time               3.56
INSTRUM           spect
PROBHD   5 mm CPQCI 1H-
PULPROG          zgpg30
TD                65356
SOLVENT             THF
NS                 1024
DS                    4
SWH           40760.871 Hz
FIDRES         0.623675 Hz
AQ            0.8017003 sec
RG               182.53
DW               12.267 usec
DE                18.00 usec
TE                298.0 K
D1           2.00000000 sec
D11          0.03000000 sec
TD0                   1

======== CHANNEL f1 ========
SFO1        176.1232717 MHz
NUC1                13C
P1                12.00 usec
PLW1       105.00000000 W

======== CHANNEL f2 ========
SFO2        700.3628014 MHz
NUC2                 1H
CPDPRG[2        waltz16
PCPD2             65.00 usec
PLW2         9.89999962 W
PLW12        0.15564001 W
PLW13        0.07837200 W

F2 - Processing parameters
SI               131072
SF          176.1055281 MHz
WDW                  EM
SSB      0
LB                 1.00 Hz
GB       0
PC                 1.40
```

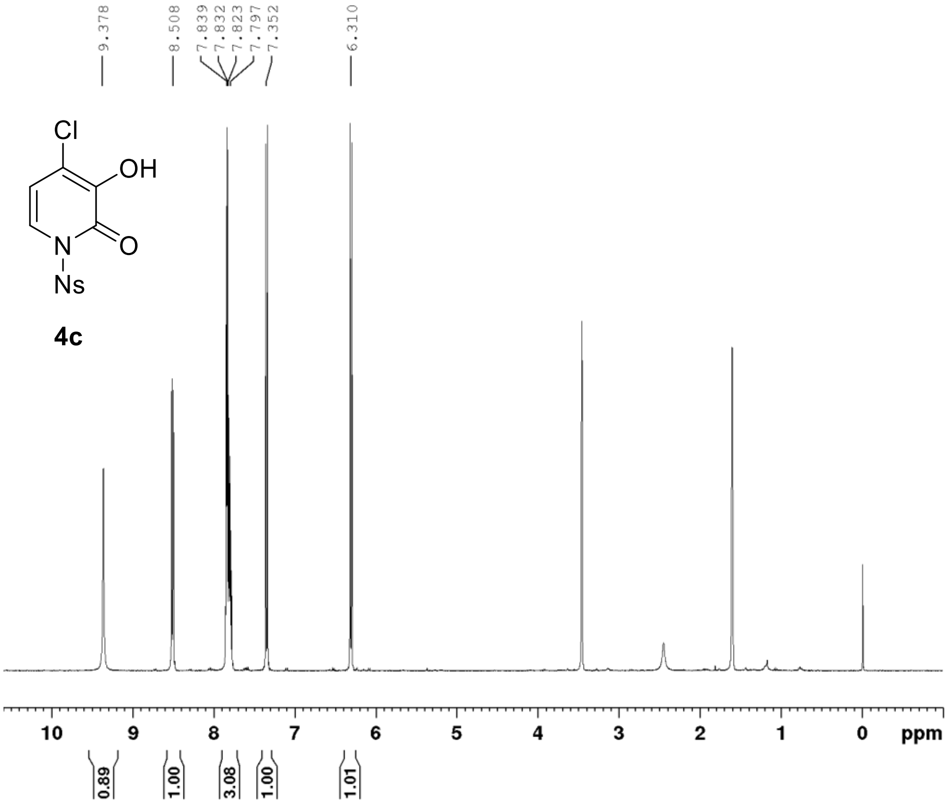


```
Current Data Parameters
NAME          56-1.2_2PC1
EXPNO                 490
PROCNO                  1

F2 - Acquisition Parameters
Date_            20200122
Time                16.49
INSTRUM             spect
PROBHD   5 mm PABBO BB/
PULPROG              zg30
TD                  65536
SOLVENT               THF
NS                     16
DS                      2
SWH           8012.820 Hz
FIDRES        0.122266 Hz
AQ           4.0894465 sec
RG                 205.35
DW              62.400 usec
DE                6.50 usec
TE               298.0 K
D1          1.00000000 sec
TD0                     1

======== CHANNEL f1 ========
SFO1        400.1024708 MHz
NUC1                   1H
P1                13.70 usec
PLW1        12.00000000 W

F2 - Processing parameters
SI                  65536
SF          400.1000649 MHz
WDW                    EM
SSB      0
LB                0.30 Hz
GB       0
PC                   1.00
```

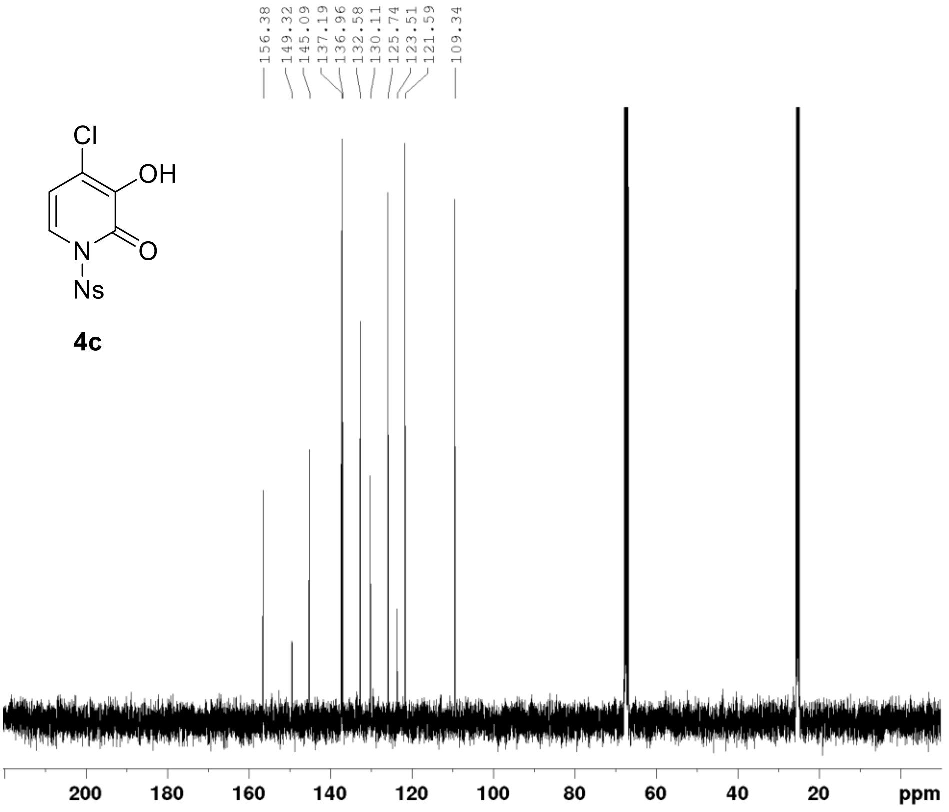


```
Current Data Parameters
NAME                 4-Cl
EXPNO                 291
PROCNO                  1

F2 - Acquisition Parameters
Date_            20190613
Time                 1.53
INSTRUM             spect
PROBHD   5 mm PABBO BB/
PULPROG           zgpg30
TD                  65536
SOLVENT               THF
NS                   1024
DS                      4
SWH          24038.461 Hz
FIDRES        0.366798 Hz
AQ           1.3631488 sec
RG                 205.35
DW              20.800 usec
DE                6.50 usec
TE               298.0 K
D1          2.00000000 sec
D11         0.03000000 sec
TD0                     1

======== CHANNEL f1 ========
SFO1        100.6152851 MHz
NUC1                  13C
P1                10.00 usec
PLW1        48.00000000 W

======== CHANNEL f2 ========
SFO2        400.1016004 MHz
NUC2                   1H
CPDPRG[2          waltz16
PCPD2             90.00 usec
PLW2        12.00000000 W
PLW12        0.27805999 W
PLW13        0.22522999 W

F2 - Processing parameters
SI                  32768
SF          100.6051472 MHz
WDW                    EM
SSB      0
LB                1.00 Hz
GB       0
PC                   1.40
```

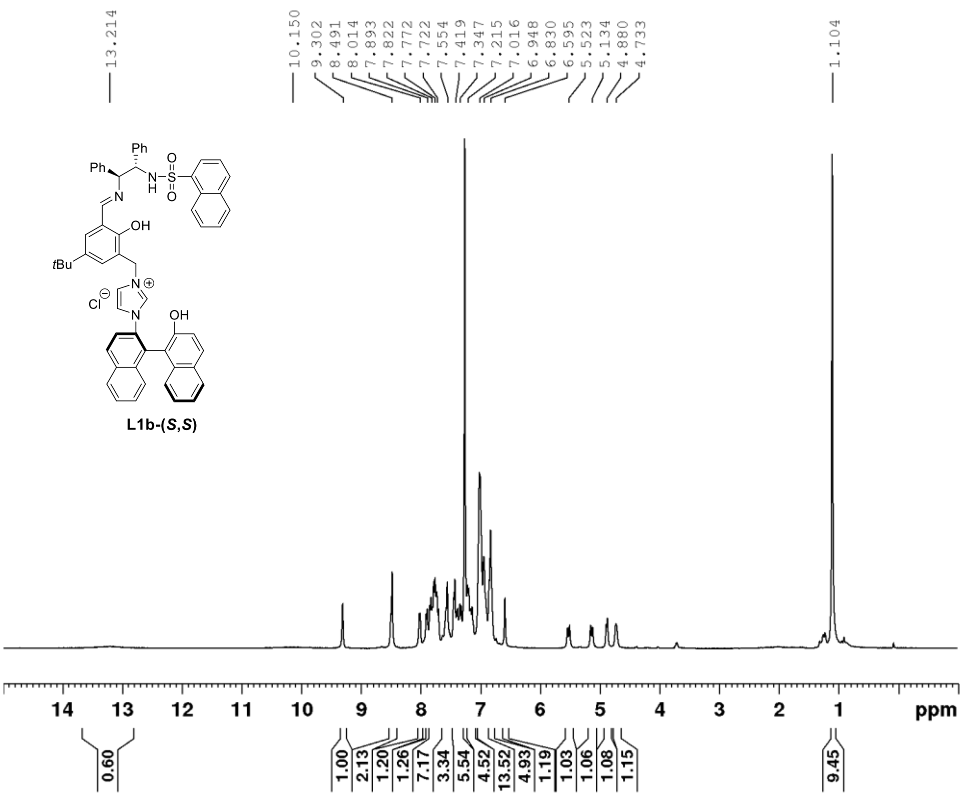

Current Data Parameters
NAME 62-MA-LS018
EXPNO 30
PROCNO 1

F2 - Acquisition Parameters
Date_ 20180807
Time 8.30
INSTRUM spect
PROBHD 5 mm PABBO BB/
PULPROG zg30
TD 65536
SOLVENT CDCl3
NS 16
DS 2
SWH 8012.820 Hz
FIDRES 0.122266 Hz
AQ 4.0894465 sec
RG 100.21
DW 62.400 usec
DE 6.50 usec
TE 298.0 K
D1 1.00000000 sec
TD0 1

======== CHANNEL f1 ========
SFO1 400.1024708 MHz
NUC1 1H
P1 13.70 usec
PLW1 12.00000000 W

F2 - Processing parameters
SI 65536
SF 400.1000107 MHz
WDW EM
SSB 0
LB 0.30 Hz
GB 0
PC 1.00

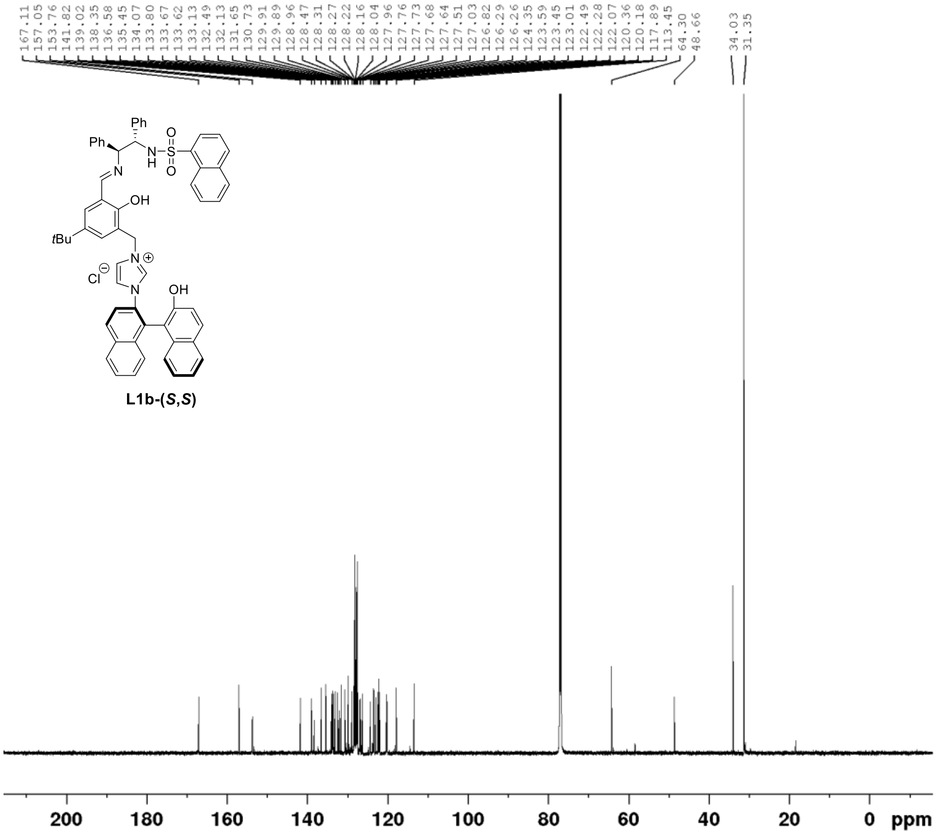

Current Data Parameters
NAME LS018
EXPNO 101
PROCNO 1

F2 - Acquisition Parameters
Date_ 20180720
Time 7.24
INSTRUM spect
PROBHD 5 mm CPQCI 1H-
PULPROG zgpg30
TD 65356
SOLVENT CDCl3
NS 2048
DS 4
SWH 40760.871 Hz
FIDRES 0.623675 Hz
AQ 0.8017003 sec
RG 182.53
DW 12.267 usec
DE 18.00 usec
TE 298.0 K
D1 2.00000000 sec
D11 0.03000000 sec
TD0 1

======== CHANNEL f1 ========
SFO1 176.1232717 MHz
NUC1 13C
P1 12.00 usec
PLW1 105.00000000 W

======== CHANNEL f2 ========
SFO2 700.3628014 MHz
NUC2 1H
CPDPRG[2 waltz16
PCPD2 65.00 usec
PLW2 9.89999962 W
PLW12 0.15564001 W
PLW13 0.07837200 W

F2 - Processing parameters
SI 131072
SF 176.1056620 MHz
WDW EM
SSB 0
LB 1.00 Hz
GB 0
PC 1.40

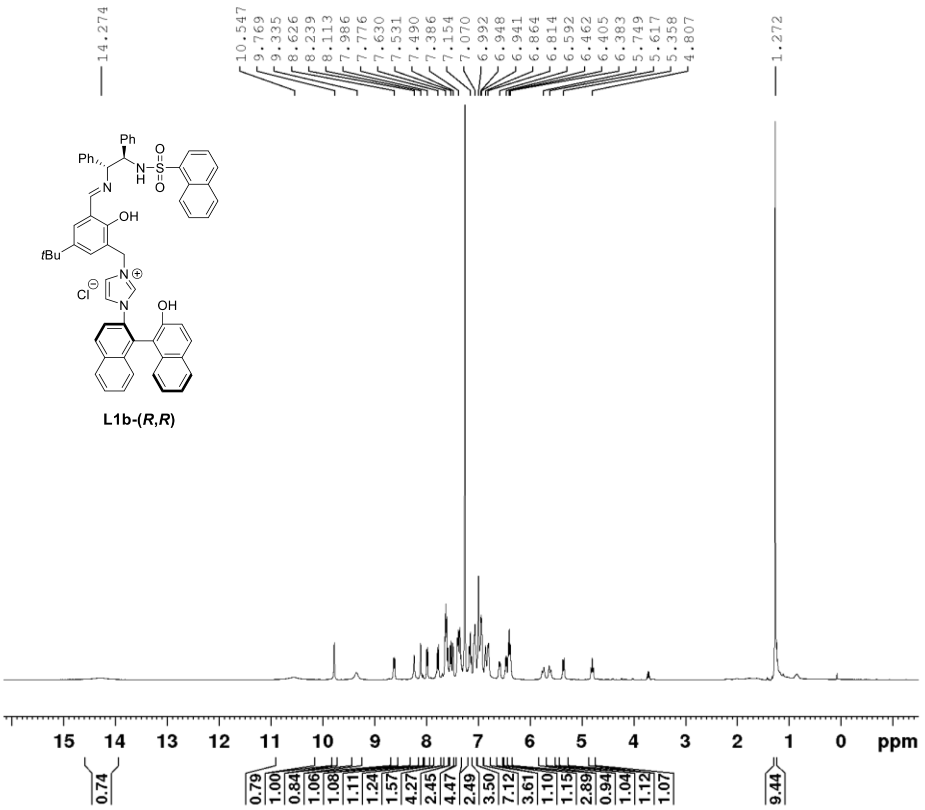


```
Current Data Parameters
NAME          62-MA-LS017
EXPNO                  20
PROCNO                  1

F2 - Acquisition Parameters
Date_            20180807
Time                 8.24
INSTRUM             spect
PROBHD   5 mm PABBO BB/
PULPROG              zg30
TD                  65536
SOLVENT             CDCl3
NS                     16
DS                      2
SWH              8012.820 Hz
FIDRES           0.122266 Hz
AQ              4.0894465 sec
RG                 124.07
DW                 62.400 usec
DE                   6.50 usec
TE                  298.0 K
D1             1.00000000 sec
TD0                     1

======== CHANNEL f1 ========
SFO1          400.1024708 MHz
NUC1                   1H
P1                  13.70 usec
PLW1          12.00000000 W

F2 - Processing parameters
SI                  65536
SF            400.1000100 MHz
WDW                    EM
SSB      0
LB                   0.30 Hz
GB       0
PC                   1.00
```

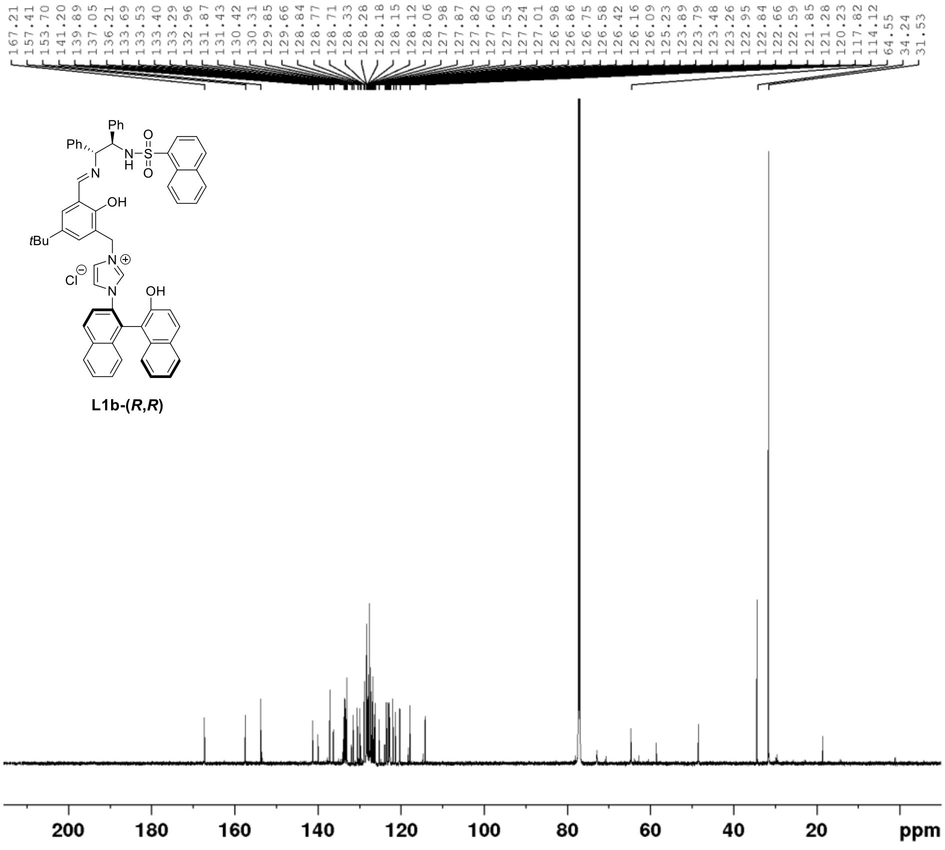


```
Current Data Parameters
NAME             6SRORK~M
EXPNO                  20
PROCNO                  1

F2 - Acquisition Parameters
Date_            20180807
Time                10.27
INSTRUM             spect
PROBHD   5 mm CPQCI 1H-
PULPROG            zgpg30
TD                  65356
SOLVENT             CDCl3
NS                   2048
DS                      4
SWH             40760.871 Hz
FIDRES           0.623675 Hz
AQ              0.8017003 sec
RG                 182.53
DW                 12.267 usec
DE                  18.00 usec
TE                  298.0 K
D1             2.00000000 sec
D11            0.03000000 sec
TD0                     1

======== CHANNEL f1 ========
SFO1          176.1232717 MHz
NUC1                  13C
P1                  12.00 usec
PLW1         105.00000000 W

======== CHANNEL f2 ========
SFO2          700.3628014 MHz
NUC2                   1H
CPDPRG[2          waltz16
PCPD2               65.00 usec
PLW2           9.89999962 W
PLW12          0.15564001 W
PLW13          0.07837200 W

F2 - Processing parameters
SI                 131072
SF            176.1056620 MHz
WDW                    EM
SSB      0
LB                   1.00 Hz
GB       0
PC                   1.40
```

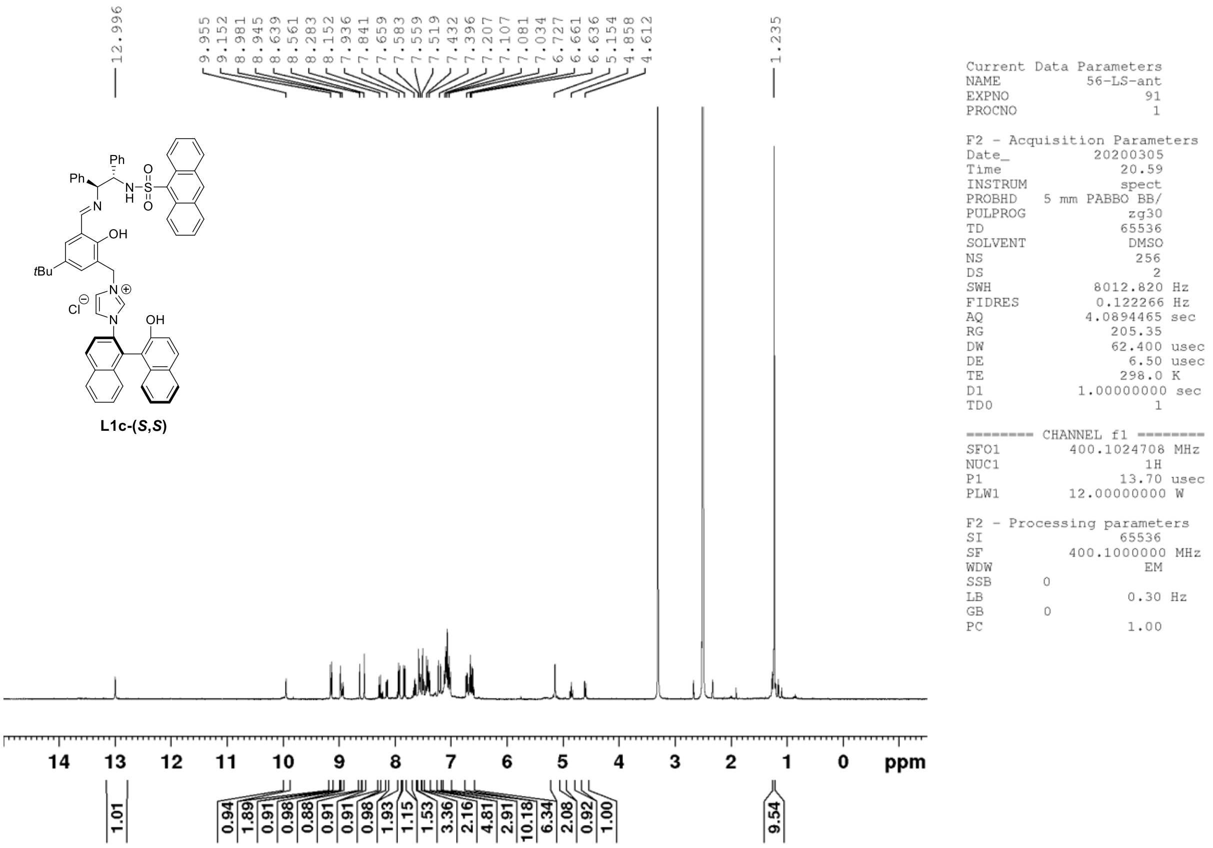
12.996
9.955 9.152 8.981 8.945 8.639 8.561 8.283 8.152 7.936 7.841 7.659 7.583 7.559 7.519 7.432 7.396 7.207 7.107 7.081 7.034 6.727 6.661 6.636 5.154 4.858 4.612
1.235
Ph
Ph
N
N
H
S
O
O
OH
tBu
N
N
Cl
OH
L1c-(S,S)
14 13 12 11 10 9 8 7 6 5 4 3 2 1 0 ppm
1.01
0.94 1.89 0.91 0.98 0.88 0.91 0.91 0.98 1.93 1.15 1.53 3.36 2.16 4.81 2.91 10.18 6.34 2.08 0.92 1.00
9.54
Current Data Parameters
NAME 56-LS-ant
EXPNO 91
PROCNO 1
F2 - Acquisition Parameters
Date_ 20200305
Time 20.59
INSTRUM spect
PROBHD 5 mm PABBO BB/
PULPROG zg30
TD 65536
SOLVENT DMSO
NS 256
DS 2
SWH 8012.820 Hz
FIDRES 0.122266 Hz
AQ 4.0894465 sec
RG 205.35
DW 62.400 usec
DE 6.50 usec
TE 298.0 K
D1 1.00000000 sec
TD0 1
======== CHANNEL f1 ========
SFO1 400.1024708 MHz
NUC1 1H
P1 13.70 usec
PLW1 12.00000000 W
F2 - Processing parameters
SI 65536
SF 400.1000000 MHz
WDW EM
SSB 0
LB 0.30 Hz
GB 0
PC 1.00

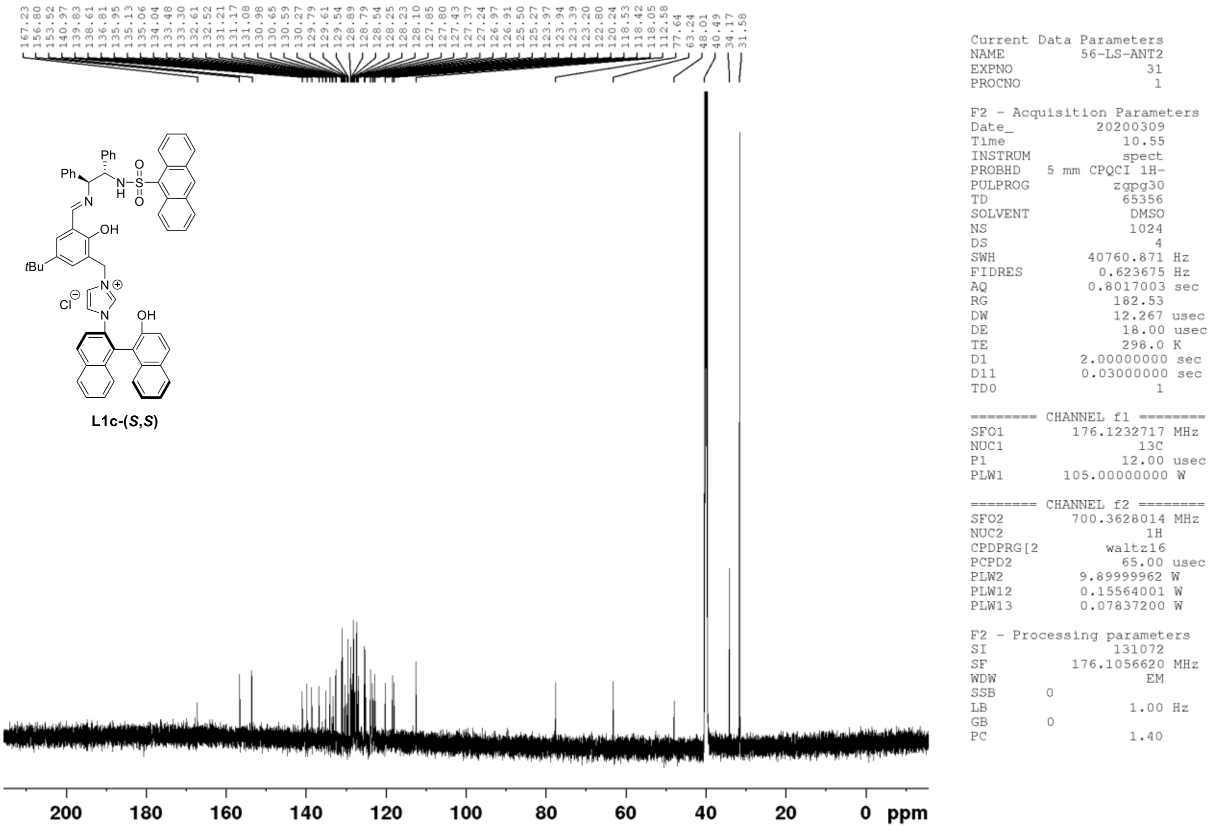
167.23 156.80 153.52 140.97 139.83 138.61 136.81 135.95 135.13 135.06 134.04 133.48 133.30 132.61 132.52 131.21 131.17 131.08 130.98 130.65 130.59 130.27 129.79 129.61 129.54 128.89 128.79 128.54 128.25 128.23 128.10 127.85 127.80 127.43 127.37 127.24 126.97 126.91 125.50 125.27 123.97 123.94 123.39 123.20 122.80 120.24 118.53 118.42 118.05 112.58 77.64 63.24 48.01 40.49 34.17 31.58
Ph
Ph
N
N
H
S
O
O
OH
tBu
N
N
Cl
OH
L1c-(S,S)
200 180 160 140 120 100 80 60 40 20 0 ppm
Current Data Parameters
NAME 56-LS-ANT2
EXPNO 31
PROCNO 1
F2 - Acquisition Parameters
Date_ 20200309
Time 10.55
INSTRUM spect
PROBHD 5 mm CPQCI 1H-
PULPROG zgpg30
TD 65356
SOLVENT DMSO
NS 1024
DS 4
SWH 40760.871 Hz
FIDRES 0.623675 Hz
AQ 0.8017003 sec
RG 182.53
DW 12.267 usec
DE 18.00 usec
TE 298.0 K
D1 2.00000000 sec
D11 0.03000000 sec
TD0 1
======== CHANNEL f1 ========
SFO1 176.1232717 MHz
NUC1 13C
P1 12.00 usec
PLW1 105.00000000 W
======== CHANNEL f2 ========
SFO2 700.3628014 MHz
NUC2 1H
CPDPRG[2 waltz16
PCPD2 65.00 usec
PLW2 9.89999962 W
PLW12 0.15564001 W
PLW13 0.07837200 W
F2 - Processing parameters
SI 131072
SF 176.1056620 MHz
WDW EM
SSB 0
LB 1.00 Hz
GB 0
PC 1.40

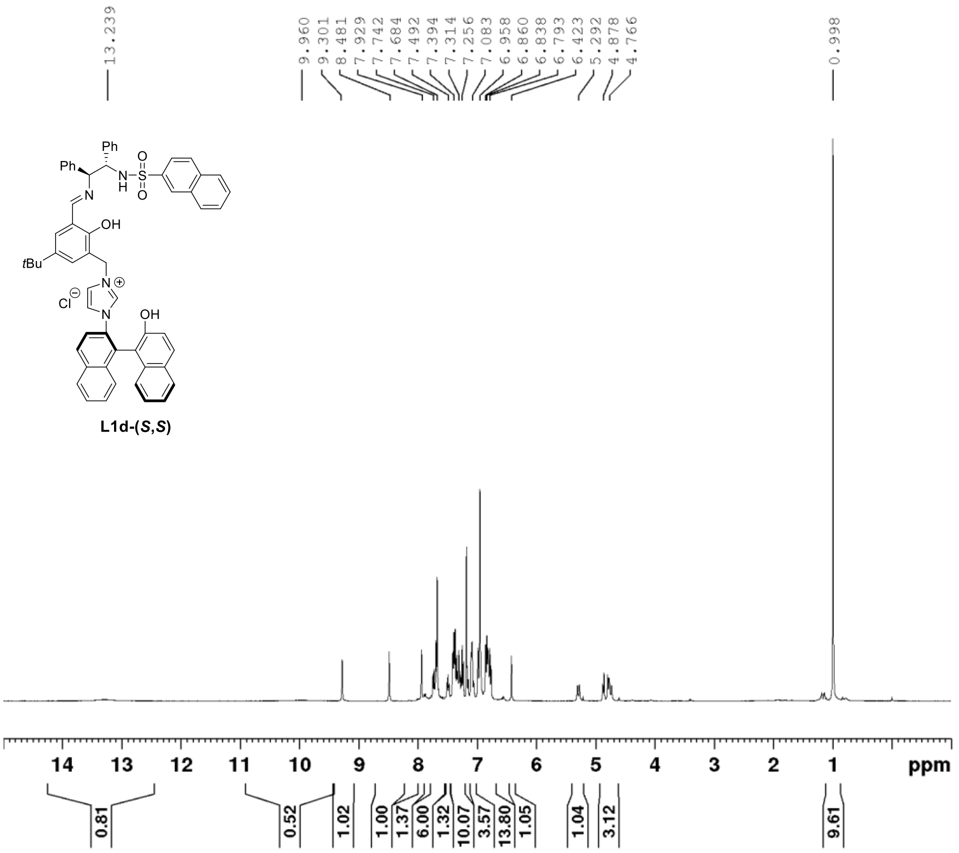

Current Data Parameters
NAME 56-LS-2-naph1.2
EXPNO 410
PROCNO 1

F2 - Acquisition Parameters
Date_ 20180720
Time 13.17
INSTRUM spect
PROBHD 5 mm PABBO BB/
PULPROG zg30
TD 65536
SOLVENT CDCl3
NS 16
DS 2
SWH 8012.820 Hz
FIDRES 0.122266 Hz
AQ 4.0894465 sec
RG 124.07
DW 62.400 usec
DE 6.50 usec
TE 298.0 K
D1 1.00000000 sec
TD0 1

======== CHANNEL f1 ========
SFO1 400.1024708 MHz
NUC1 1H
P1 13.70 usec
PLW1 12.00000000 W

F2 - Processing parameters
SI 65536
SF 400.1000400 MHz
WDW EM
SSB 0
LB 0.30 Hz
GB 0
PC 1.00

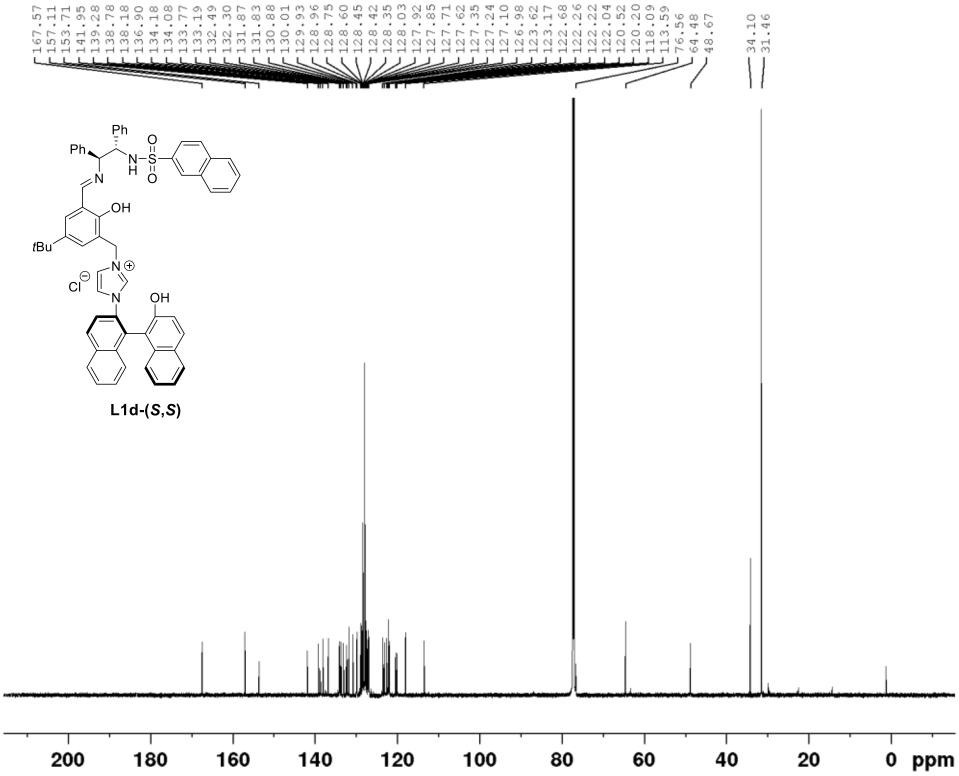

Current Data Parameters
NAME 56-LS-S,S-2-naph
EXPNO 31
PROCNO 1

F2 - Acquisition Parameters
Date_ 20180726
Time 12.17
INSTRUM spect
PROBHD 5 mm CPQCI 1H-
PULPROG zgpg30
TD 65356
SOLVENT CDCl3
NS 2048
DS 4
SWH 40760.871 Hz
FIDRES 0.623675 Hz
AQ 0.8017003 sec
RG 182.53
DW 12.267 usec
DE 18.00 usec
TE 298.0 K
D1 2.00000000 sec
D11 0.03000000 sec
TD0 1

======== CHANNEL f1 ========
SFO1 176.1232717 MHz
NUC1 13C
P1 12.00 usec
PLW1 105.00000000 W

======== CHANNEL f2 ========
SFO2 700.3628014 MHz
NUC2 1H
CPDPRG[2 waltz16
PCPD2 65.00 usec
PLW2 9.89999962 W
PLW12 0.15564001 W
PLW13 0.07837200 W

F2 - Processing parameters
SI 131072
SF 176.1056416 MHz
WDW EM
SSB 0
LB 1.00 Hz
GB 0
PC 1.40

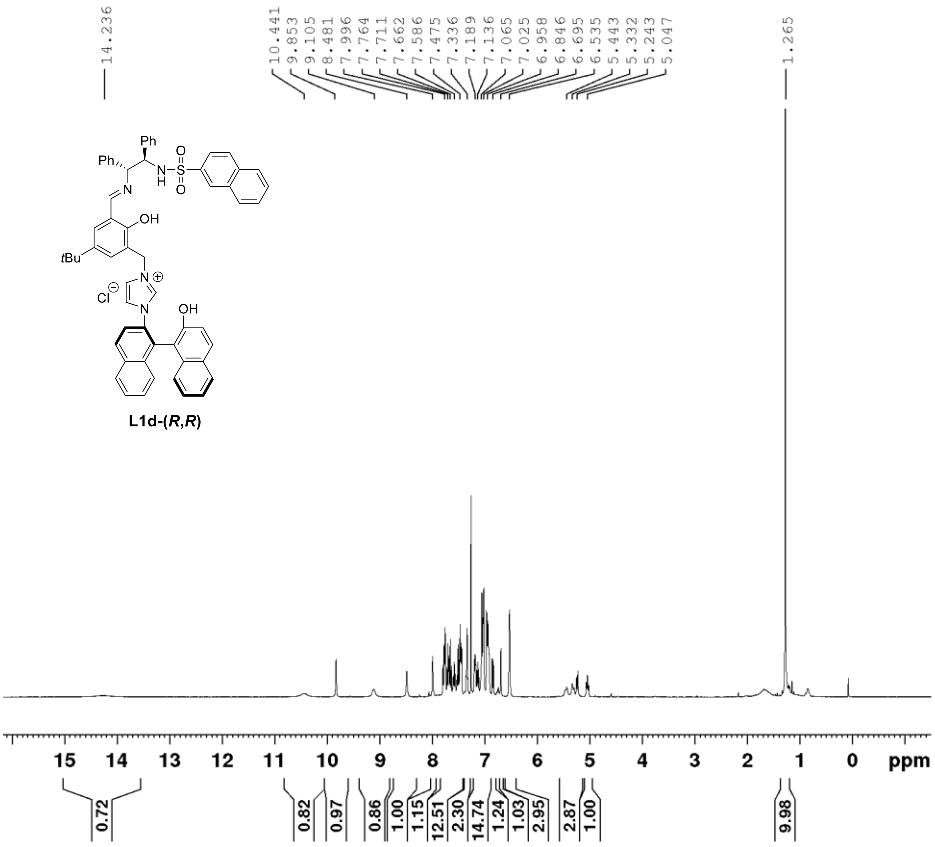


```
Current Data Parameters
NAME      56-1.4LS-2-naph
EXPNO                  40
PROCNO                  1

F2 - Acquisition Parameters
Date_            20180730
Time                 9.39
INSTRUM             spect
PROBHD   5 mm PABBO BB/
PULPROG              zg30
TD                  65536
SOLVENT             CDCl3
NS                     16
DS                      2
SWH              8012.820 Hz
FIDRES           0.122266 Hz
AQ              4.0894465 sec
RG                 182.64
DW                 62.400 usec
DE                   6.50 usec
TE                  298.0 K
D1             1.00000000 sec
TD0                     1

======== CHANNEL f1 ========
SFO1         400.1024708 MHz
NUC1                   1H
P1                  13.70 usec
PLW1          12.00000000 W

F2 - Processing parameters
SI                  65536
SF            400.1000081 MHz
WDW                    EM
SSB      0
LB                   0.30 Hz
GB       0
PC                   1.00
```

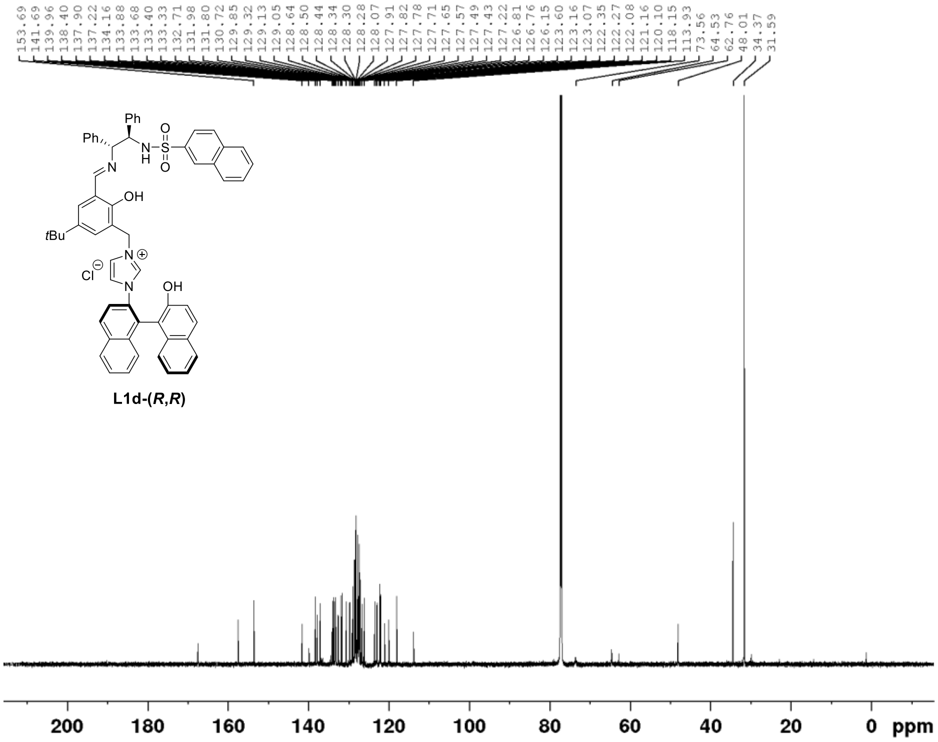


```
Current Data Parameters
NAME      56-LS-RR-2-HAPH
EXPNO                  21
PROCNO                  1

F2 - Acquisition Parameters
Date_            20180730
Time                11.52
INSTRUM             spect
PROBHD   5 mm CPQCI 1H-
PULPROG            zgpg30
TD                  65356
SOLVENT             CDCl3
NS                   2048
DS                      4
SWH             40760.871 Hz
FIDRES           0.623675 Hz
AQ              0.8017003 sec
RG                 182.53
DW                 12.267 usec
DE                  18.00 usec
TE                  298.0 K
D1             2.00000000 sec
D11            0.03000000 sec
TD0                     1

======== CHANNEL f1 ========
SFO1         176.1232717 MHz
NUC1                  13C
P1                  12.00 usec
PLW1         105.00000000 W

======== CHANNEL f2 ========
SFO2         700.3628014 MHz
NUC2                   1H
CPDPRG[2          waltz16
PCPD2               65.00 usec
PLW2           9.89999962 W
PLW12          0.15564001 W
PLW13          0.07837200 W

F2 - Processing parameters
SI                 131072
SF            176.1056425 MHz
WDW                    EM
SSB      0
LB                   1.00 Hz
GB       0
PC                   1.40
```

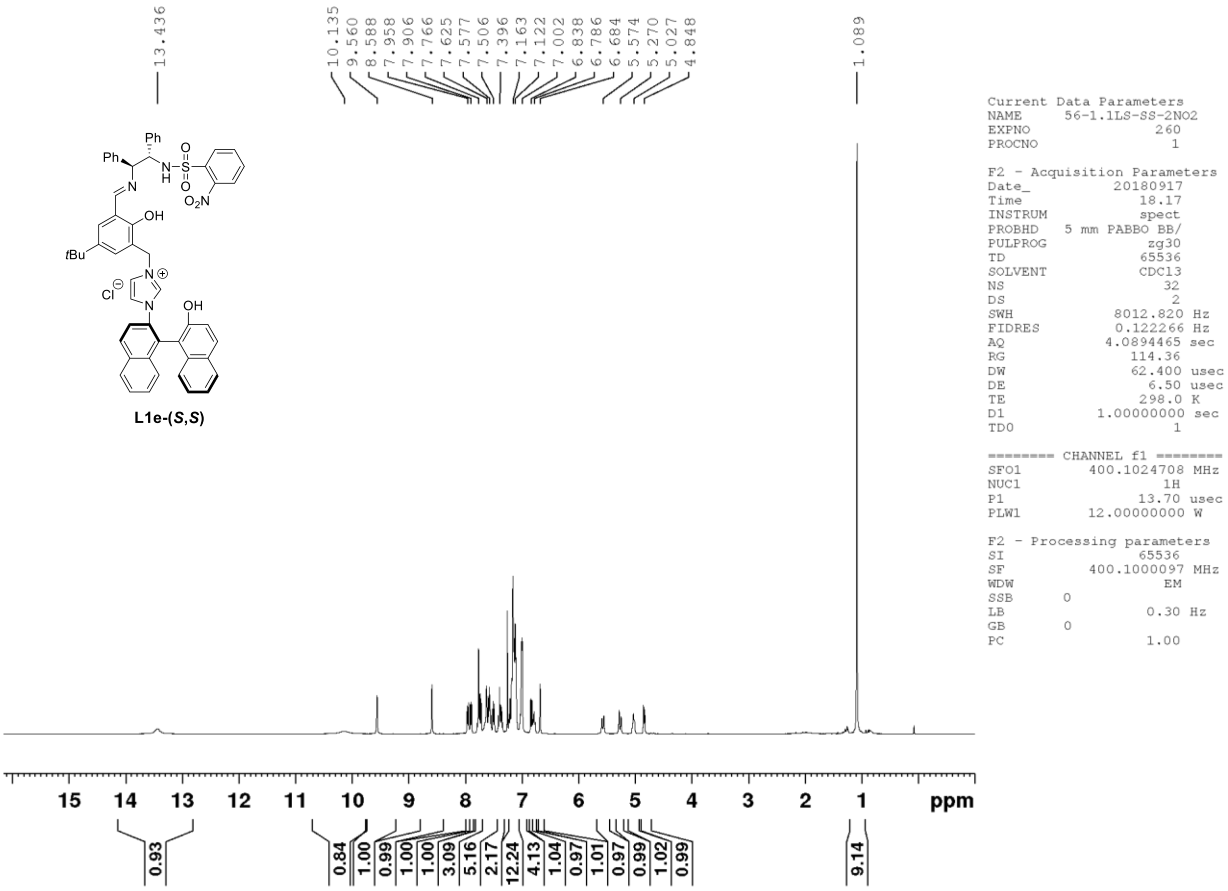
13.436
10.135
9.560
8.588
7.958
7.906
7.766
7.625
7.577
7.506
7.396
7.163
7.122
7.002
6.838
6.786
6.684
5.574
5.270
5.027
4.848
1.089
L1e-(S,S)
Current Data Parameters
NAME 56-1.1LS-SS-2NO2
EXPNO 260
PROCNO 1
F2 - Acquisition Parameters
Date_ 20180917
Time 18.17
INSTRUM spect
PROBHD 5 mm PABBO BB/
PULPROG zg30
TD 65536
SOLVENT CDCl3
NS 32
DS 2
SWH 8012.820 Hz
FIDRES 0.122266 Hz
AQ 4.0894465 sec
RG 114.36
DW 62.400 usec
DE 6.50 usec
TE 298.0 K
D1 1.00000000 sec
TD0 1
======== CHANNEL f1 ========
SFO1 400.1024708 MHz
NUC1 1H
P1 13.70 usec
PLW1 12.00000000 W
F2 - Processing parameters
SI 65536
SF 400.1000097 MHz
WDW EM
SSB 0
LB 0.30 Hz
GB 0
PC 1.00
15 14 13 12 11 10 9 8 7 6 5 4 3 2 1 ppm
0.93
0.84
1.00
0.99
1.00
1.00
3.09
5.16
2.17
12.24
4.13
1.04
0.97
1.01
0.97
0.99
1.02
0.99
9.14

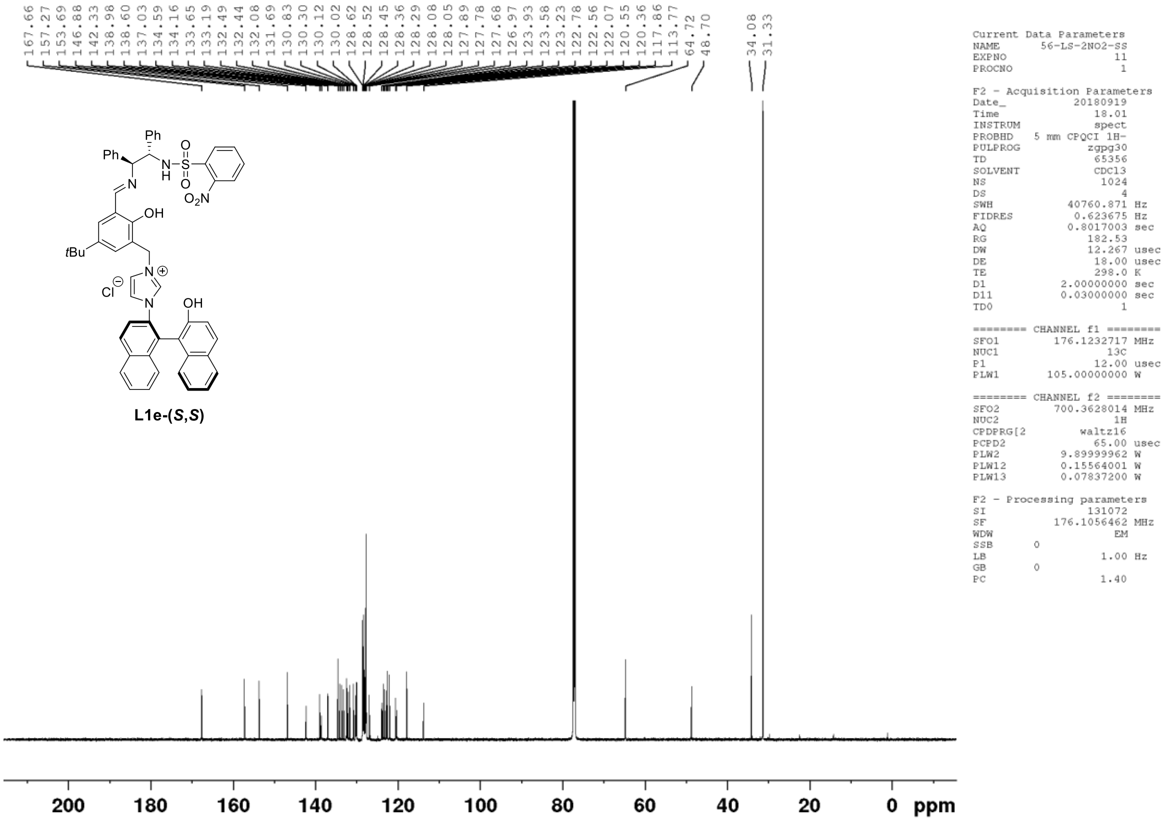
167.66
157.27
153.69
146.88
142.33
138.98
138.60
137.03
134.59
134.16
133.65
133.19
132.49
132.44
132.08
131.69
130.83
130.30
130.12
130.02
128.62
128.52
128.45
128.36
128.29
128.08
128.05
127.89
127.78
127.68
126.97
123.93
123.58
123.23
122.78
122.56
122.07
120.55
120.36
117.86
113.77
64.72
48.70
34.08
31.33
L1e-(S,S)
Current Data Parameters
NAME 56-LS-2NO2-SS
EXPNO 11
PROCNO 1
F2 - Acquisition Parameters
Date_ 20180919
Time 18.01
INSTRUM spect
PROBHD 5 mm CPQCI 1H-
PULPROG zgpg30
TD 65356
SOLVENT CDCl3
NS 1024
DS 4
SWH 40760.871 Hz
FIDRES 0.623675 Hz
AQ 0.8017003 sec
RG 182.53
DW 12.267 usec
DE 18.00 usec
TE 298.0 K
D1 2.00000000 sec
D11 0.03000000 sec
TD0 1
======= CHANNEL f1 =======
SFO1 176.1232717 MHz
NUC1 13C
P1 12.00 usec
PLW1 105.00000000 W
======= CHANNEL f2 =======
SFO2 700.3628014 MHz
NUC2 1H
CPDPRG[2 waltz16
PCPD2 65.00 usec
PLW2 9.89999962 W
PLW12 0.15564001 W
PLW13 0.07837200 W
F2 - Processing parameters
SI 131072
SF 176.1056462 MHz
WDW EM
SSB 0
LB 1.00 Hz
GB 0
PC 1.40
200 180 160 140 120 100 80 60 40 20 0 ppm

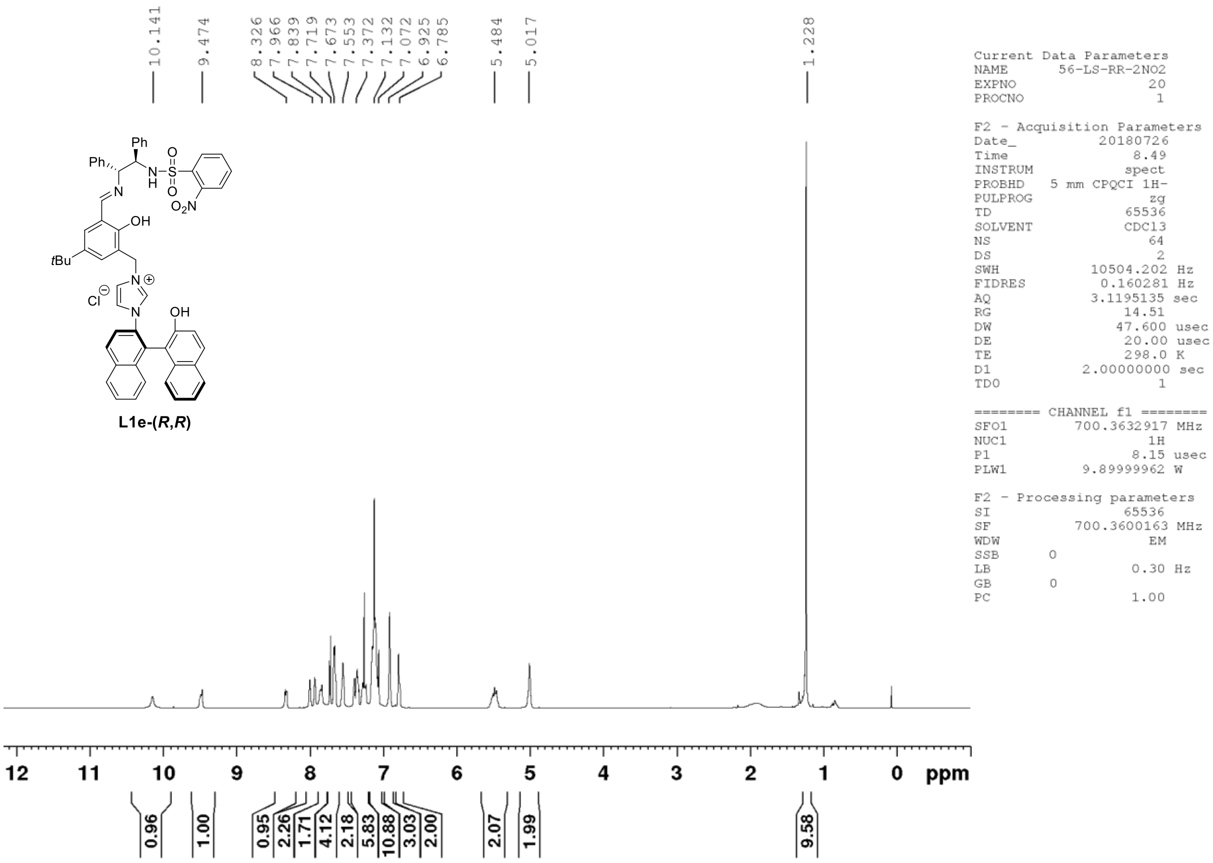
10.141
9.474
8.326
7.966
7.839
7.719
7.673
7.553
7.372
7.132
7.072
6.925
6.785
5.484
5.017
1.228
Ph
Ph
N
NH
S
O
O
O2N
OH
tBu
Cl
N
N
OH
L1e-(R,R)
0.96
1.00
0.95
2.26
1.71
4.12
2.18
5.83
10.88
3.03
2.00
2.07
1.99
9.58
12 11 10 9 8 7 6 5 4 3 2 1 0 ppm
Current Data Parameters
NAME 56-LS-RR-2NO2
EXPNO 20
PROCNO 1
F2 - Acquisition Parameters
Date_ 20180726
Time 8.49
INSTRUM spect
PROBHD 5 mm CPQCI 1H-
PULPROG zg
TD 65536
SOLVENT CDCl3
NS 64
DS 2
SWH 10504.202 Hz
FIDRES 0.160281 Hz
AQ 3.1195135 sec
RG 14.51
DW 47.600 usec
DE 20.00 usec
TE 298.0 K
D1 2.00000000 sec
TD0 1
======== CHANNEL f1 ========
SFO1 700.3632917 MHz
NUC1 1H
P1 8.15 usec
PLW1 9.89999962 W
F2 - Processing parameters
SI 65536
SF 700.3600163 MHz
WDW EM
SSB 0
LB 0.30 Hz
GB 0
PC 1.00

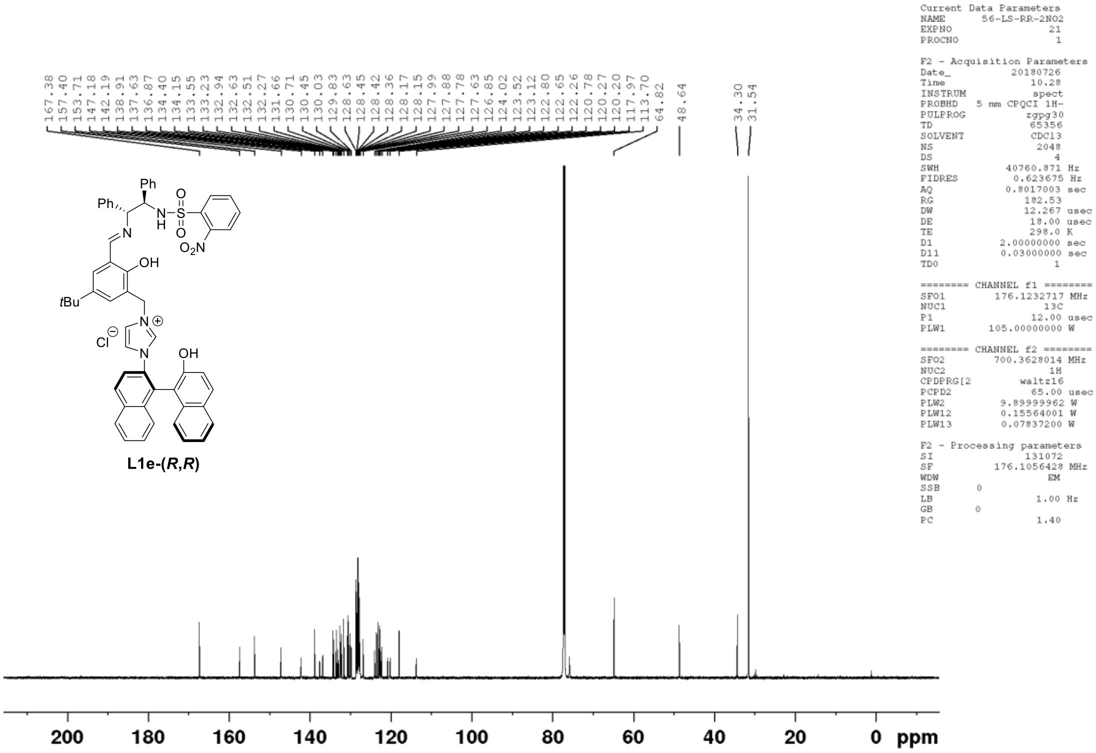
167.38
157.40
153.71
147.18
142.19
138.91
137.63
136.87
134.40
134.15
133.55
133.23
132.94
132.63
132.51
132.27
131.66
130.71
130.45
130.03
129.83
128.63
128.45
128.42
128.36
128.17
128.15
127.99
127.88
127.78
127.63
126.85
124.02
123.52
123.12
122.80
122.65
122.26
120.78
120.27
120.20
117.97
113.70
64.82
48.64
34.30
31.54
L1e-(R,R)
200 180 160 140 120 100 80 60 40 20 0 ppm
Current Data Parameters
NAME 56-LS-RR-2NO2
EXPNO 21
PROCNO 1
F2 - Acquisition Parameters
Date_ 20180726
Time 10.28
INSTRUM spect
PROBHD 5 mm CPQCI 1H-
PULPROG zgpg30
TD 65356
SOLVENT CDCl3
NS 2048
DS 4
SWH 40760.871 Hz
FIDRES 0.623675 Hz
AQ 0.8017003 sec
RG 182.53
DW 12.267 usec
DE 18.00 usec
TE 298.0 K
D1 2.00000000 sec
D11 0.03000000 sec
TD0 1
======== CHANNEL f1 ========
SFO1 176.1232717 MHz
NUC1 13C
P1 12.00 usec
PLW1 105.00000000 W
======== CHANNEL f2 ========
SFO2 700.3628014 MHz
NUC2 1H
CPDPRG[2 waltz16
PCPD2 65.00 usec
PLW2 9.89999962 W
PLW12 0.15564001 W
PLW13 0.07837200 W
F2 - Processing parameters
SI 131072
SF 176.1056428 MHz
WDW EM
SSB 0
LB 1.00 Hz
GB 0
PC 1.40

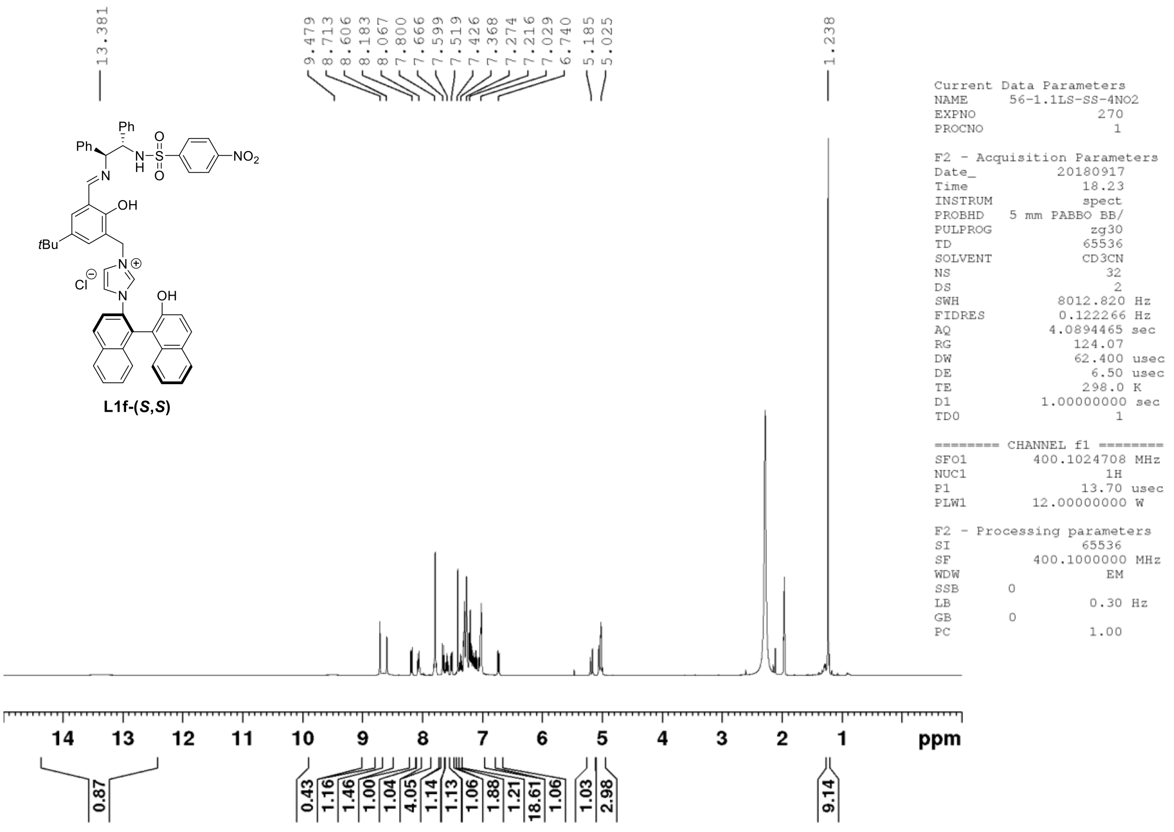

13.381
9.479
8.713
8.606
8.183
8.067
7.800
7.666
7.599
7.519
7.426
7.368
7.274
7.216
7.029
6.740
5.185
5.025
1.238
Ph
Ph
N
NH
NO2
OH
tBu
N
Cl
N
OH
L1f-(S,S)
Current Data Parameters
NAME 56-1.1LS-SS-4NO2
EXPNO 270
PROCNO 1
F2 - Acquisition Parameters
Date_ 20180917
Time 18.23
INSTRUM spect
PROBHD 5 mm PABBO BB/
PULPROG zg30
TD 65536
SOLVENT CD3CN
NS 32
DS 2
SWH 8012.820 Hz
FIDRES 0.122266 Hz
AQ 4.0894465 sec
RG 124.07
DW 62.400 usec
DE 6.50 usec
TE 298.0 K
D1 1.00000000 sec
TD0 1
======== CHANNEL f1 ========
SFO1 400.1024708 MHz
NUC1 1H
P1 13.70 usec
PLW1 12.00000000 W
F2 - Processing parameters
SI 65536
SF 400.1000000 MHz
WDW EM
SSB 0
LB 0.30 Hz
GB 0
PC 1.00
14 13 12 11 10 9 8 7 6 5 4 3 2 1 ppm
0.87
0.43
1.16
1.46
1.00
1.04
4.05
1.14
1.13
1.06
1.88
1.21
18.61
1.06
1.03
2.98
9.14

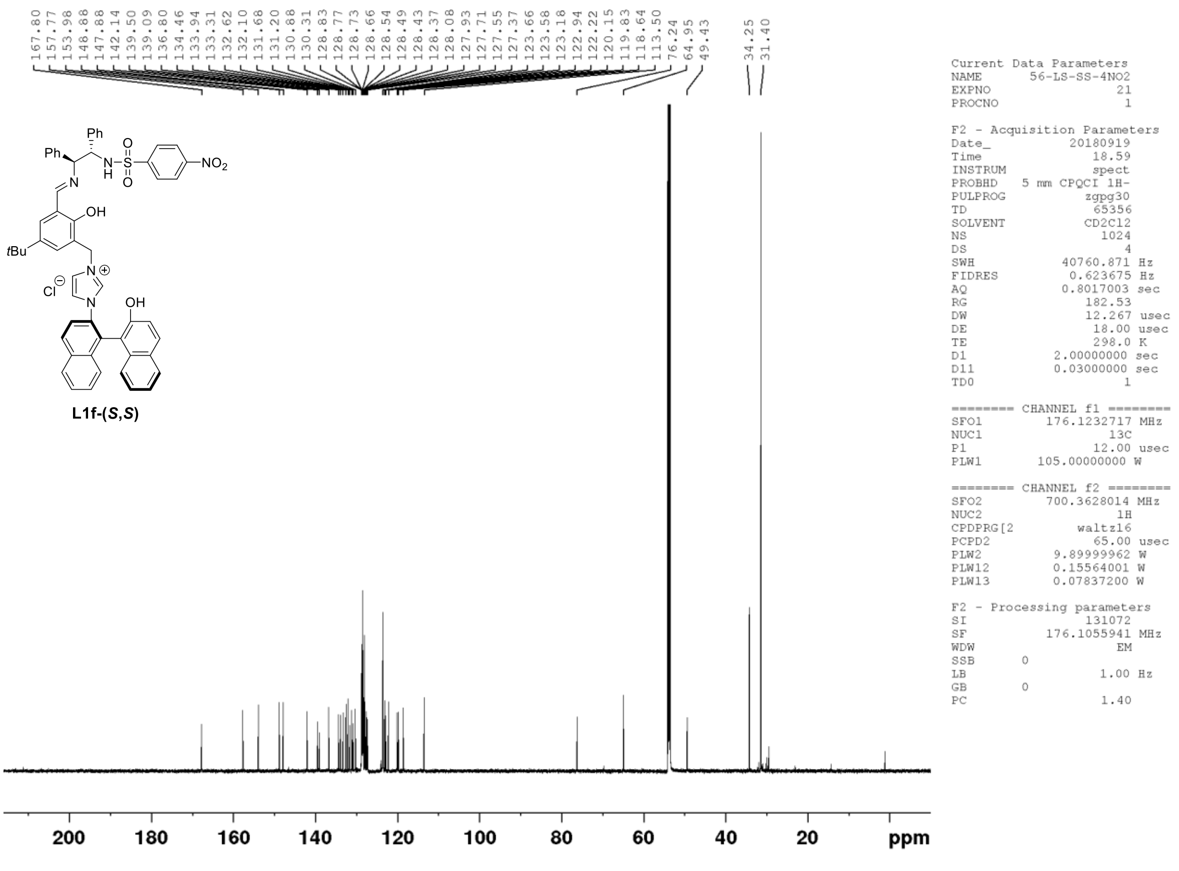

167.80
157.77
153.98
148.88
147.88
142.14
139.50
139.09
136.80
134.46
133.94
133.31
132.62
132.10
131.68
131.20
130.88
130.31
128.83
128.77
128.73
128.66
128.54
128.49
128.43
128.37
128.08
127.93
127.71
127.55
127.37
123.66
123.58
123.18
122.94
122.22
120.15
119.83
118.64
113.50
76.24
64.95
49.43
34.25
31.40
Ph
Ph
N
NH
NO2
OH
tBu
N
Cl
N
OH
L1f-(S,S)
Current Data Parameters
NAME 56-LS-SS-4NO2
EXPNO 21
PROCNO 1
F2 - Acquisition Parameters
Date_ 20180919
Time 18.59
INSTRUM spect
PROBHD 5 mm CPQCI 1H-
PULPROG zgpg30
TD 65356
SOLVENT CD2Cl2
NS 1024
DS 4
SWH 40760.871 Hz
FIDRES 0.623675 Hz
AQ 0.8017003 sec
RG 182.53
DW 12.267 usec
DE 18.00 usec
TE 298.0 K
D1 2.00000000 sec
D11 0.03000000 sec
TD0 1
======== CHANNEL f1 ========
SFO1 176.1232717 MHz
NUC1 13C
P1 12.00 usec
PLW1 105.00000000 W
======== CHANNEL f2 ========
SFO2 700.3628014 MHz
NUC2 1H
CPDPRG[2 waltz16
PCPD2 65.00 usec
PLW2 9.89999962 W
PLW12 0.15564001 W
PLW13 0.07837200 W
F2 - Processing parameters
SI 131072
SF 176.1055941 MHz
WDW EM
SSB 0
LB 1.00 Hz
GB 0
PC 1.40
200 180 160 140 120 100 80 60 40 20 ppm

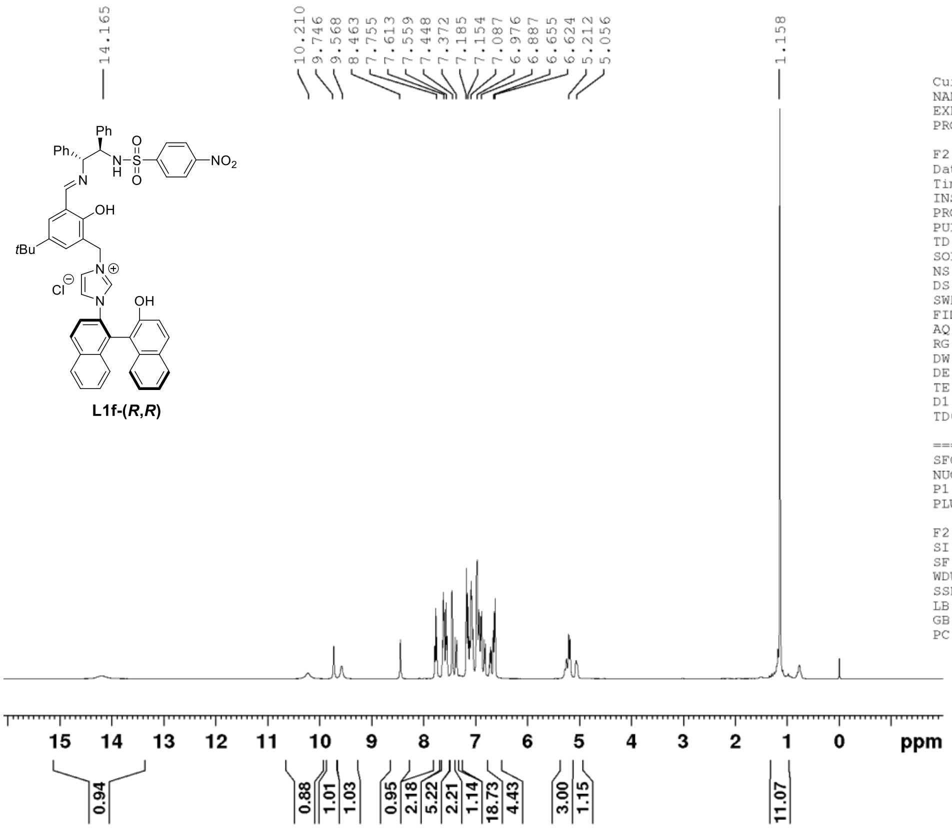


```
Current Data Parameters
NAME      56-1.4LS-RR-4-NO2
EXPNO                   290
PROCNO                    1

F2 - Acquisition Parameters
Date_              20180928
Time                  17.31
INSTRUM               spect
PROBHD   5 mm PABBO BB/
PULPROG                zg30
TD                    65536
SOLVENT               CDCl3
NS                       16
DS                        2
SWH             8012.820 Hz
FIDRES          0.122266 Hz
AQ             4.0894465 sec
RG                   114.36
DW               62.400 usec
DE                 6.50 usec
TE                298.0 K
D1           1.00000000 sec
TD0                       1

======== CHANNEL f1 ========
SFO1         400.1024708 MHz
NUC1                     1H
P1                 13.70 usec
PLW1         12.00000000 W

F2 - Processing parameters
SI                    65536
SF           400.1000412 MHz
WDW                      EM
SSB      0
LB                  0.30 Hz
GB       0
PC                     1.00
```

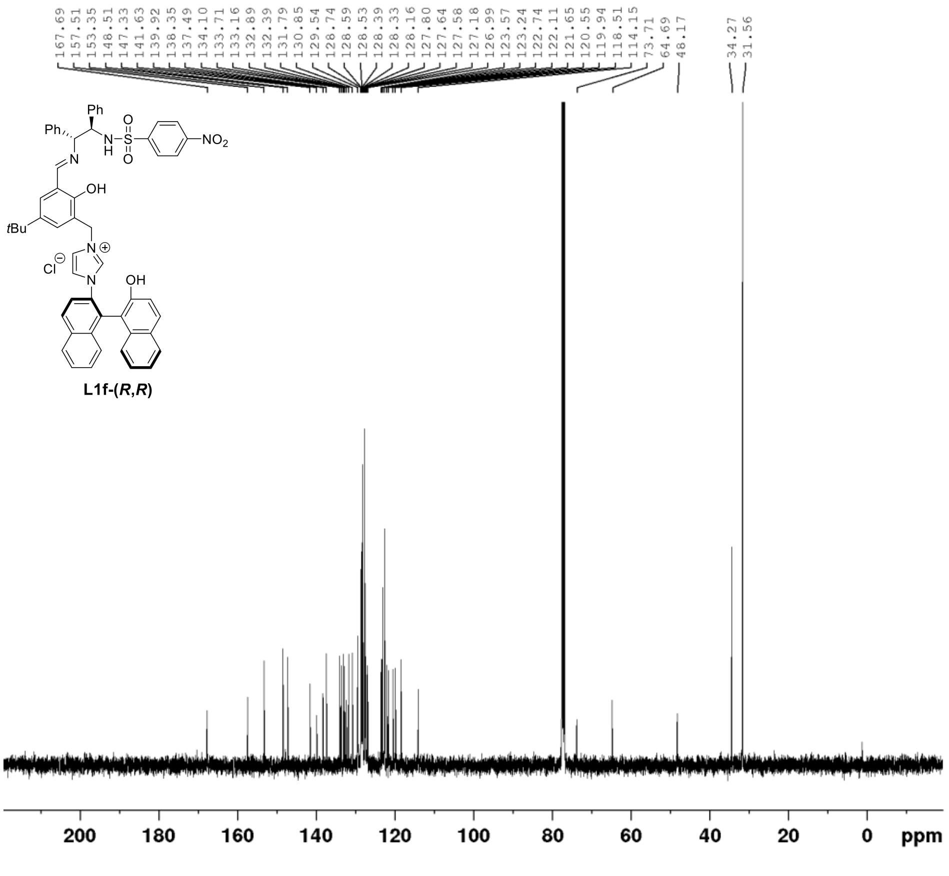


```
Current Data Parameters
NAME      56-1.4LS-RR-4-NO2
EXPNO                   291
PROCNO                    1

F2 - Acquisition Parameters
Date_              20180929
Time                   0.47
INSTRUM               spect
PROBHD   5 mm PABBO BB/
PULPROG             zgpg30
TD                    65536
SOLVENT               CDCl3
NS                     1024
DS                        4
SWH            24038.461 Hz
FIDRES          0.366798 Hz
AQ             1.3631488 sec
RG                   205.35
DW               20.800 usec
DE                 6.50 usec
TE                298.0 K
D1           2.00000000 sec
D11          0.03000000 sec
TD0                       1

======== CHANNEL f1 ========
SFO1         100.6152851 MHz
NUC1                    13C
P1                 10.00 usec
PLW1         48.00000000 W

======== CHANNEL f2 ========
SFO2         400.1016004 MHz
NUC2                     1H
CPDPRG[2            waltz16
PCPD2              90.00 usec
PLW2         12.00000000 W
PLW12         0.27805999 W
PLW13         0.22522999 W

F2 - Processing parameters
SI                    32768
SF           100.6052132 MHz
WDW                      EM
SSB      0
LB                  1.00 Hz
GB       0
PC                     1.40
```

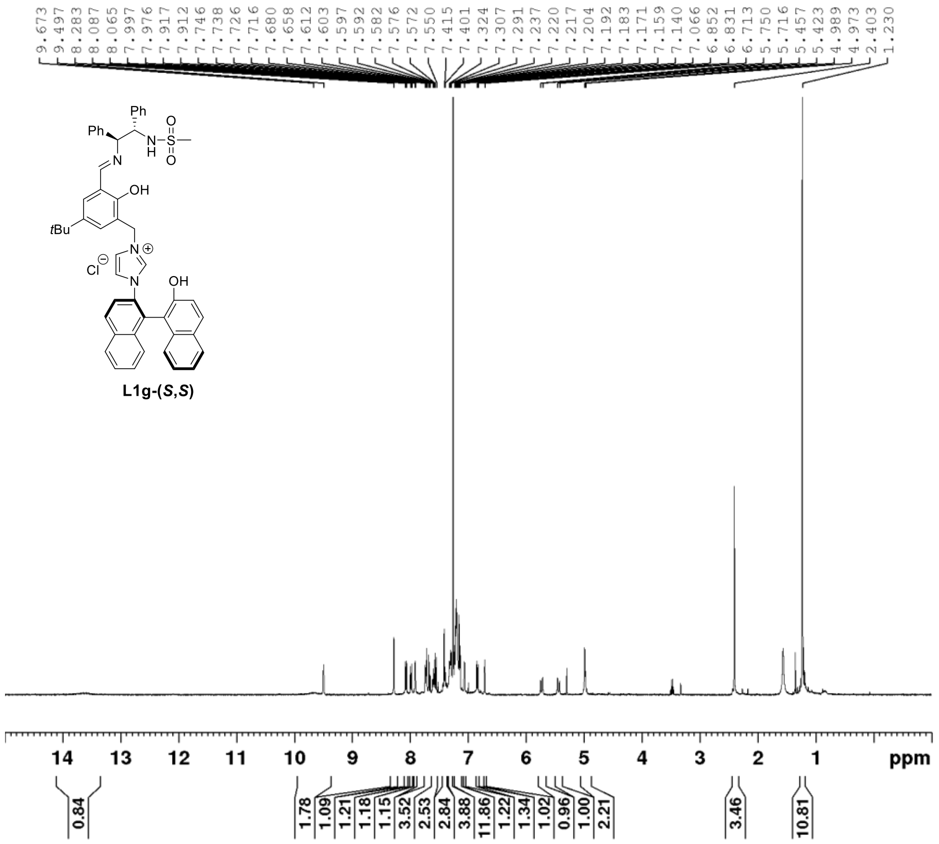


```
Current Data Parameters
NAME            38-LS-032
EXPNO                 490
PROCNO                  1

F2 - Acquisition Parameters
Date_            20160422
Time                17.44
INSTRUM             spect
PROBHD   5 mm PABBO BB/
PULPROG              zg30
TD                  65536
SOLVENT             CDCl3
NS                     64
DS                      2
SWH             8012.820 Hz
FIDRES          0.122266 Hz
AQ             4.0894465 sec
RG                 205.35
DW                 62.400 usec
DE                   6.50 usec
TE                  296.0 K
D1           1.00000000 sec
TD0                     1

======== CHANNEL f1 ========
SFO1         400.1024708 MHz
NUC1                   1H
P1                  13.70 usec
PLW1         12.00000000 W

F2 - Processing parameters
SI                  65536
SF           400.1000101 MHz
WDW                    EM
SSB      0
LB                   0.30 Hz
GB       0
PC                   1.00
```

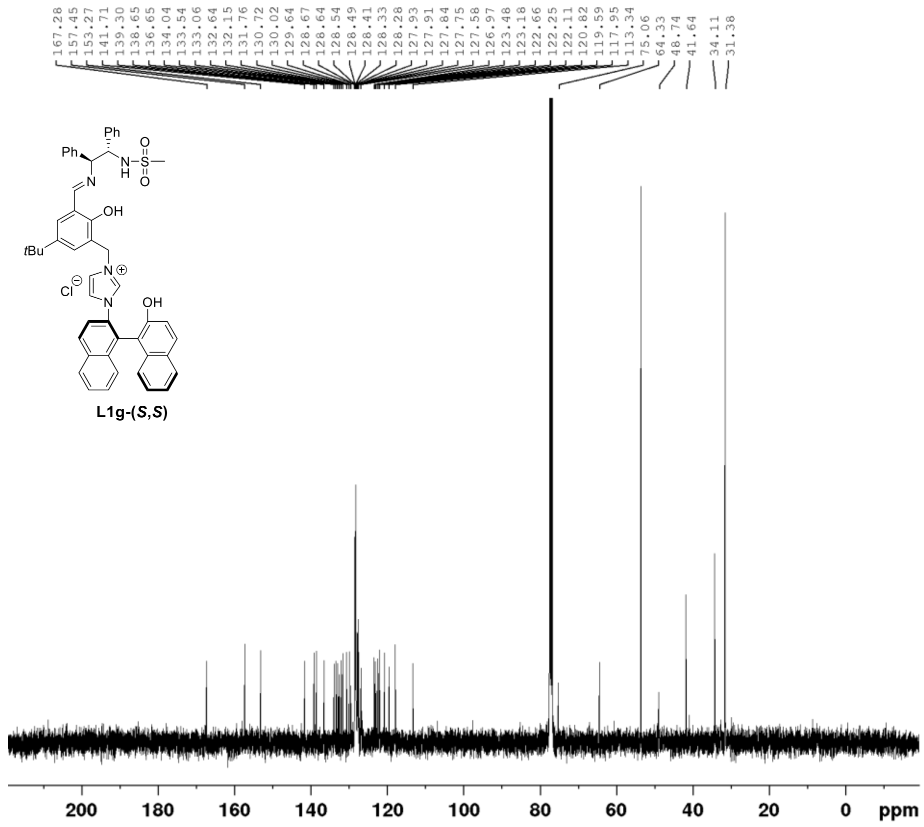


```
Current Data Parameters
NAME            38-LS-032
EXPNO                 611
PROCNO                  1

F2 - Acquisition Parameters
Date_            20160422
Time                 4.38
INSTRUM             spect
PROBHD   5 mm PABBO BB/
PULPROG            zgpg30
TD                  65536
SOLVENT             CDCl3
NS                   1024
DS                      4
SWH            24038.461 Hz
FIDRES          0.366798 Hz
AQ             1.3631488 sec
RG                 205.35
DW                 20.800 usec
DE                   6.50 usec
TE                  296.0 K
D1           2.00000000 sec
D11          0.03000000 sec
TD0                     1

======== CHANNEL f1 ========
SFO1         100.6152851 MHz
NUC1                  13C
P1                  10.00 usec
PLW1         48.00000000 W

======== CHANNEL f2 ========
SFO2         400.1016004 MHz
NUC2                   1H
CPDPRG[2          waltz16
PCPD2               90.00 usec
PLW2         12.00000000 W
PLW12         0.27805999 W
PLW13         0.22522999 W

F2 - Processing parameters
SI                  32768
SF           100.6052250 MHz
WDW                    EM
SSB      0
LB                   1.00 Hz
GB       0
PC                   1.40
```

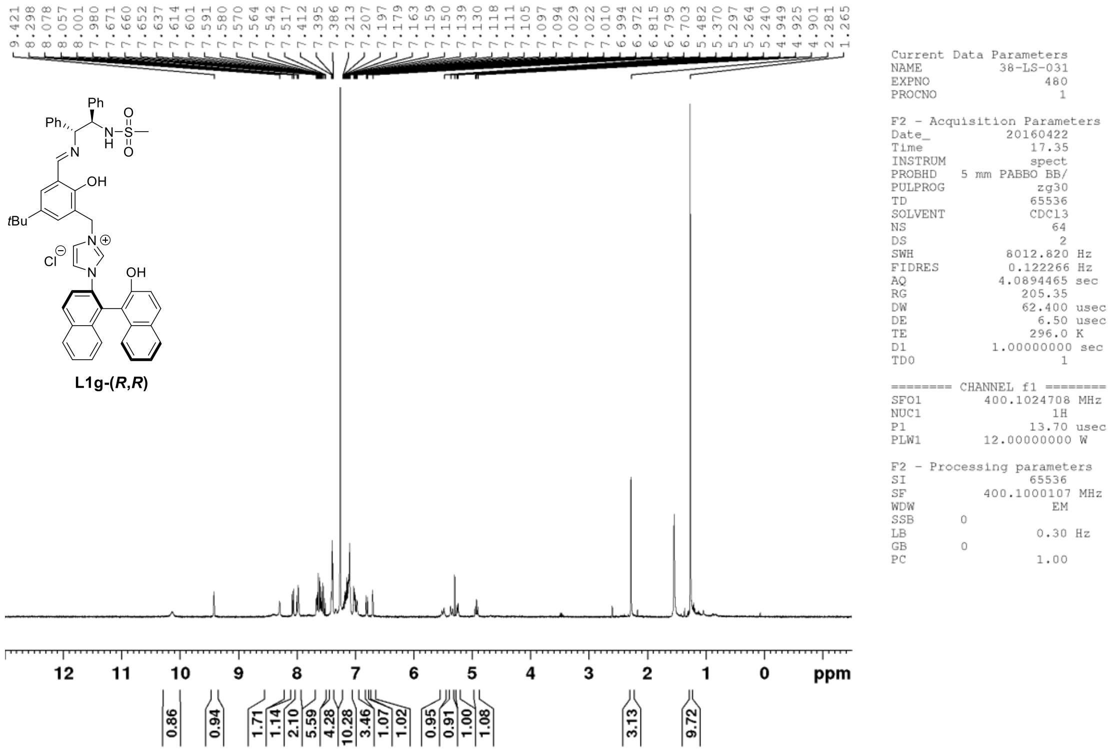
9.421 8.298 8.078 8.057 8.001 7.980 7.671 7.660 7.652 7.637 7.614 7.601 7.591 7.580 7.570 7.564 7.542 7.517 7.412 7.395 7.386 7.213 7.207 7.197 7.179 7.163 7.159 7.150 7.139 7.130 7.118 7.111 7.105 7.097 7.094 7.029 7.022 7.010 6.994 6.972 6.815 6.795 6.703 5.482 5.370 5.297 5.264 5.240 4.949 4.925 4.901 2.281 1.265
L1g-(R,R)
Current Data Parameters
NAME 38-LS-031
EXPNO 480
PROCNO 1
F2 - Acquisition Parameters
Date_ 20160422
Time 17.35
INSTRUM spect
PROBHD 5 mm PABBO BB/
PULPROG zg30
TD 65536
SOLVENT CDCl3
NS 64
DS 2
SWH 8012.820 Hz
FIDRES 0.122266 Hz
AQ 4.0894465 sec
RG 205.35
DW 62.400 usec
DE 6.50 usec
TE 296.0 K
D1 1.00000000 sec
TD0 1
======== CHANNEL f1 ========
SFO1 400.1024708 MHz
NUC1 1H
P1 13.70 usec
PLW1 12.00000000 W
F2 - Processing parameters
SI 65536
SF 400.1000107 MHz
WDW EM
SSB 0
LB 0.30 Hz
GB 0
PC 1.00
12 11 10 9 8 7 6 5 4 3 2 1 0 ppm
0.86 0.94 1.71 1.14 2.10 5.59 4.28 10.28 3.46 1.07 1.02 0.95 0.91 1.00 1.08 3.13 9.72

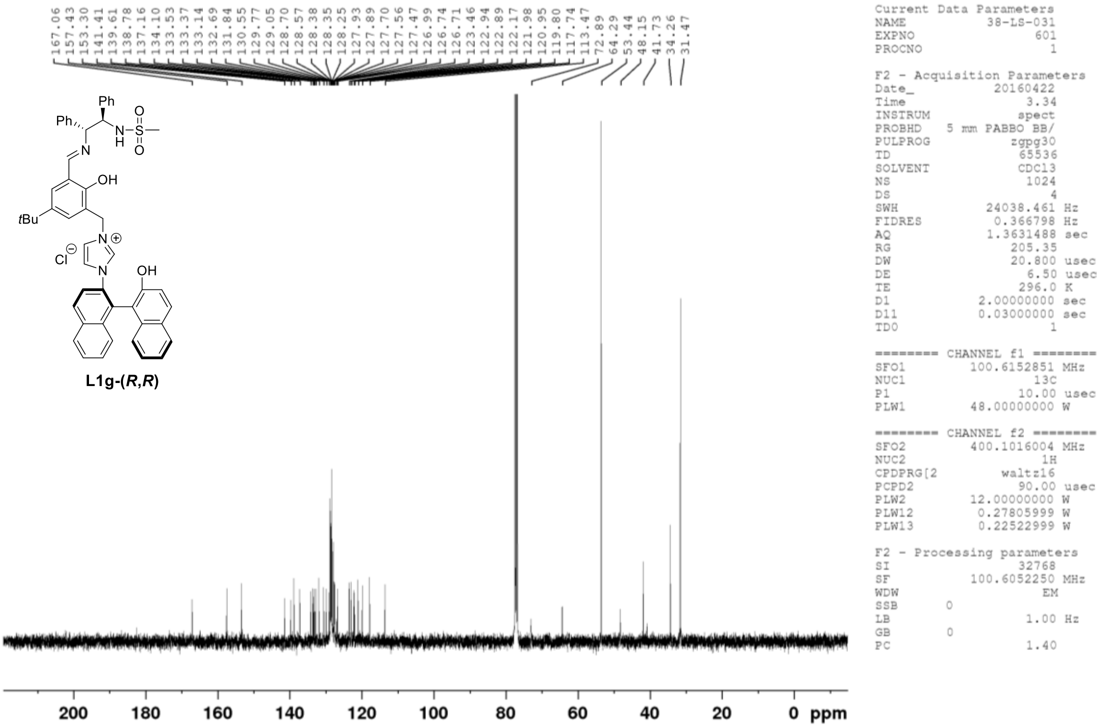
167.06 157.43 153.30 141.41 139.61 138.78 137.16 134.10 133.53 133.37 133.14 132.69 131.84 130.55 129.77 129.05 128.70 128.57 128.38 128.35 128.25 127.93 127.89 127.70 127.56 127.47 126.99 126.74 126.71 123.46 122.94 122.89 122.17 121.98 120.95 119.80 117.74 113.47 72.89 64.29 53.44 48.15 41.73 34.26 31.47
L1g-(R,R)
Current Data Parameters
NAME 38-LS-031
EXPNO 601
PROCNO 1
F2 - Acquisition Parameters
Date_ 20160422
Time 3.34
INSTRUM spect
PROBHD 5 mm PABBO BB/
PULPROG zgpg30
TD 65536
SOLVENT CDCl3
NS 1024
DS 4
SWH 24038.461 Hz
FIDRES 0.366798 Hz
AQ 1.3631488 sec
RG 205.35
DW 20.800 usec
DE 6.50 usec
TE 296.0 K
D1 2.00000000 sec
D11 0.03000000 sec
TD0 1
======== CHANNEL f1 ========
SFO1 100.6152851 MHz
NUC1 13C
P1 10.00 usec
PLW1 48.00000000 W
======== CHANNEL f2 ========
SFO2 400.1016004 MHz
NUC2 1H
CPDPRG[2 waltz16
PCPD2 90.00 usec
PLW2 12.00000000 W
PLW12 0.27805999 W
PLW13 0.22522999 W
F2 - Processing parameters
SI 32768
SF 100.6052250 MHz
WDW EM
SSB 0
LB 1.00 Hz
GB 0
PC 1.40
200 180 160 140 120 100 80 60 40 20 0 ppm

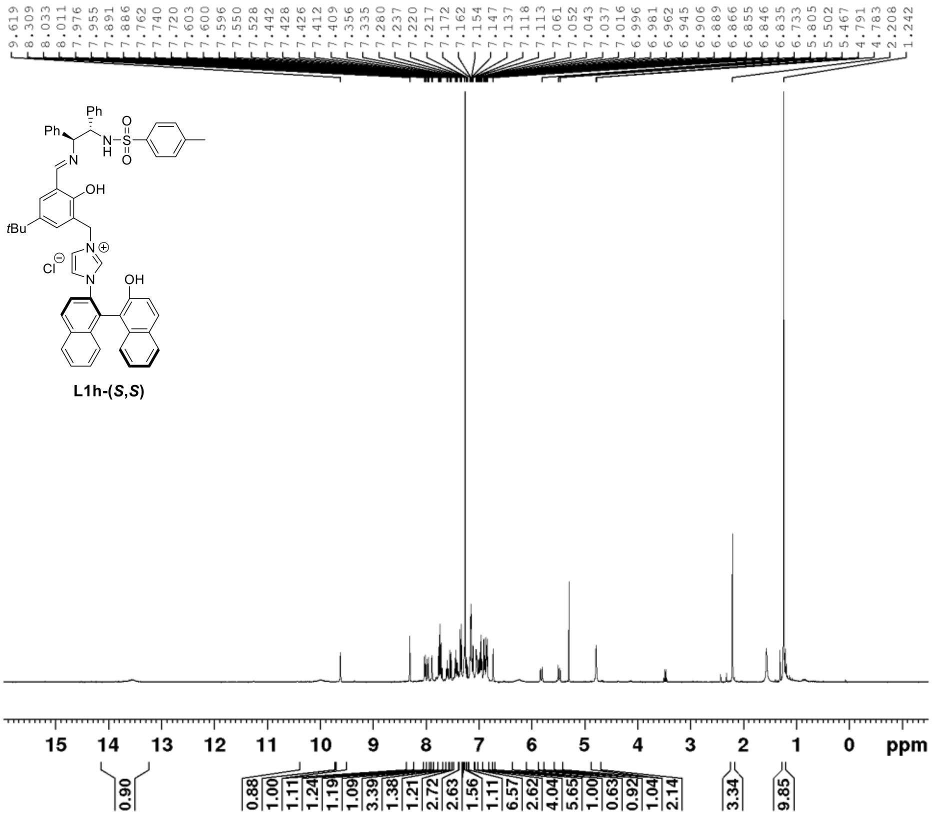

Current Data Parameters
NAME 38-LS-030.1
EXPNO 470
PROCNO 1

F2 - Acquisition Parameters
Date_ 20160422
Time 17.25
INSTRUM spect
PROBHD 5 mm PABBO BB/
PULPROG zg30
TD 65536
SOLVENT CDCl3
NS 64
DS 2
SWH 8012.820 Hz
FIDRES 0.122266 Hz
AQ 4.0894465 sec
RG 205.35
DW 62.400 usec
DE 6.50 usec
TE 296.0 K
D1 1.00000000 sec
TD0 1

======== CHANNEL f1 ========
SFO1 400.1024708 MHz
NUC1 1H
P1 13.70 usec
PLW1 12.00000000 W

F2 - Processing parameters
SI 65536
SF 400.1000100 MHz
WDW EM
SSB 0
LB 0.30 Hz
GB 0
PC 1.00

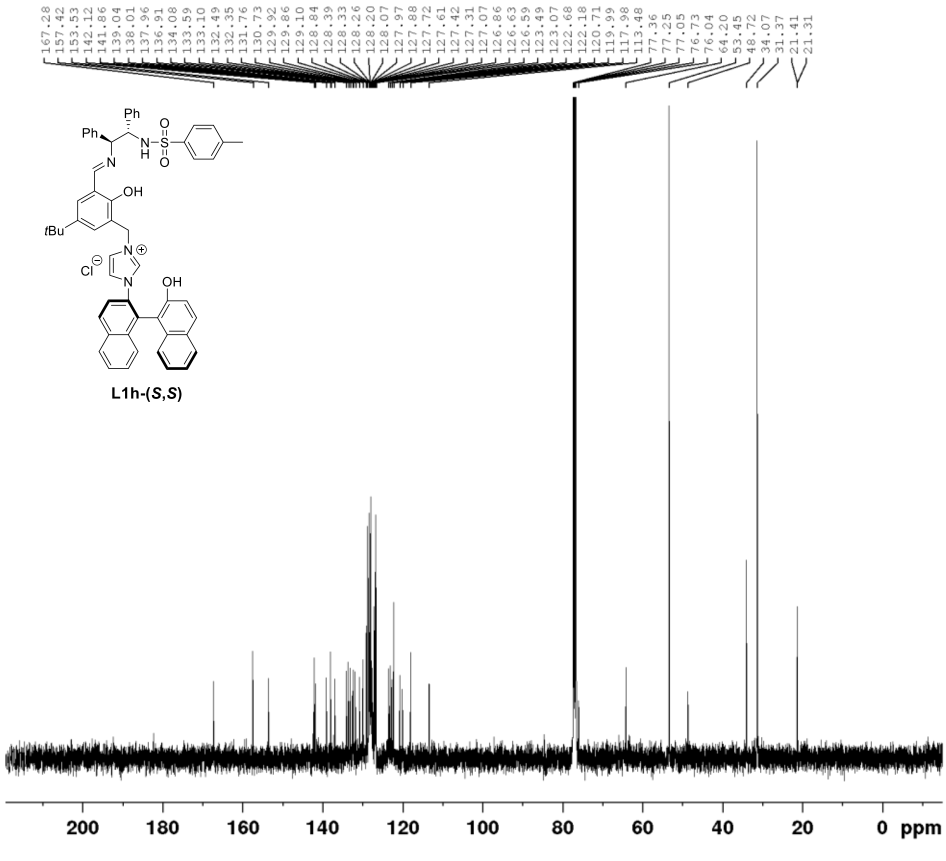

Current Data Parameters
NAME 38-LS-030.1
EXPNO 591
PROCNO 1

F2 - Acquisition Parameters
Date_ 20160422
Time 2.29
INSTRUM spect
PROBHD 5 mm PABBO BB/
PULPROG zgpg30
TD 65536
SOLVENT CDCl3
NS 1024
DS 4
SWH 24038.461 Hz
FIDRES 0.366798 Hz
AQ 1.3631488 sec
RG 205.35
DW 20.800 usec
DE 6.50 usec
TE 296.0 K
D1 2.00000000 sec
D11 0.03000000 sec
TD0 1

======== CHANNEL f1 ========
SFO1 100.6152851 MHz
NUC1 13C
P1 10.00 usec
PLW1 48.00000000 W

======== CHANNEL f2 ========
SFO2 400.1016004 MHz
NUC2 1H
CPDPRG[2 waltz16
PCPD2 90.00 usec
PLW2 12.00000000 W
PLW12 0.27805999 W
PLW13 0.22522999 W

F2 - Processing parameters
SI 32768
SF 100.6052250 MHz
WDW EM
SSB 0
LB 1.00 Hz
GB 0
PC 1.40

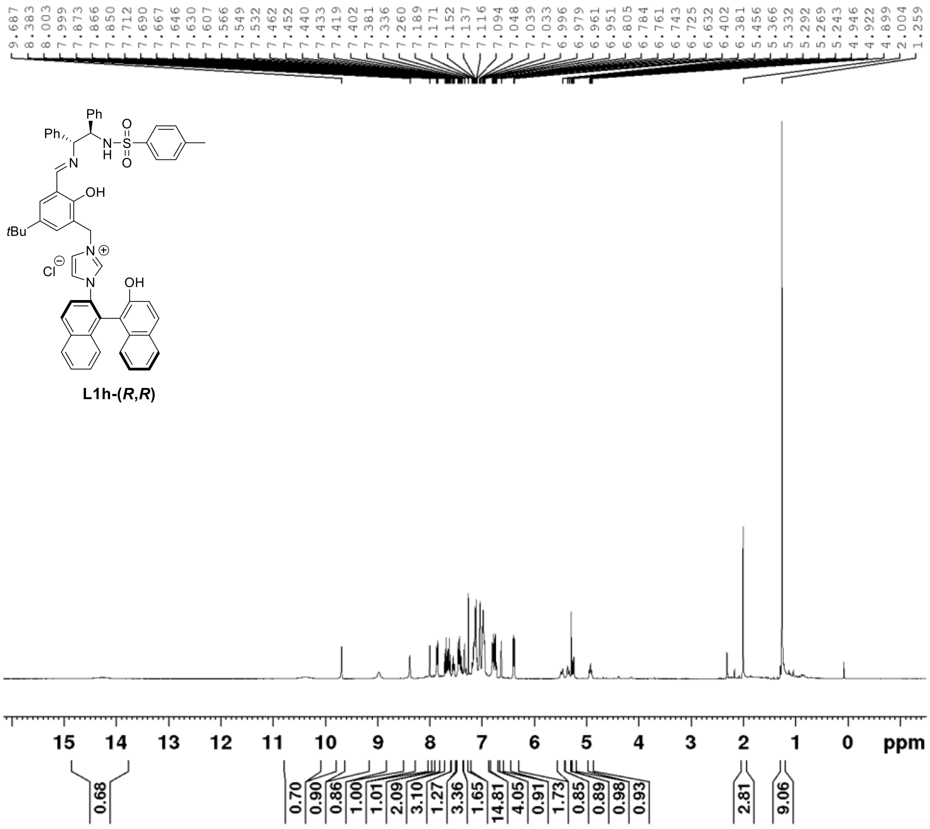

L1h-(*R*,*R*)

```
Current Data Parameters
NAME          38-LS-029
EXPNO               290
PROCNO                1

F2 - Acquisition Parameters
Date_          20160331
Time              15.20
INSTRUM           spect
PROBHD   5 mm PABBO BB/
PULPROG            zg30
TD                65536
SOLVENT           CDCl3
NS                   16
DS                    2
SWH            8012.820 Hz
FIDRES         0.122266 Hz
AQ            4.0894465 sec
RG               139.45
DW               62.400 usec
DE                 6.50 usec
TE                296.0 K
D1           1.00000000 sec
TD0                   1

======== CHANNEL f1 ========
SFO1        400.1024708 MHz
NUC1                 1H
P1                13.70 usec
PLW1        12.00000000 W

F2 - Processing parameters
SI                65536
SF          400.1000096 MHz
WDW                  EM
SSB      0
LB                 0.30 Hz
GB       0
PC                 1.00
```

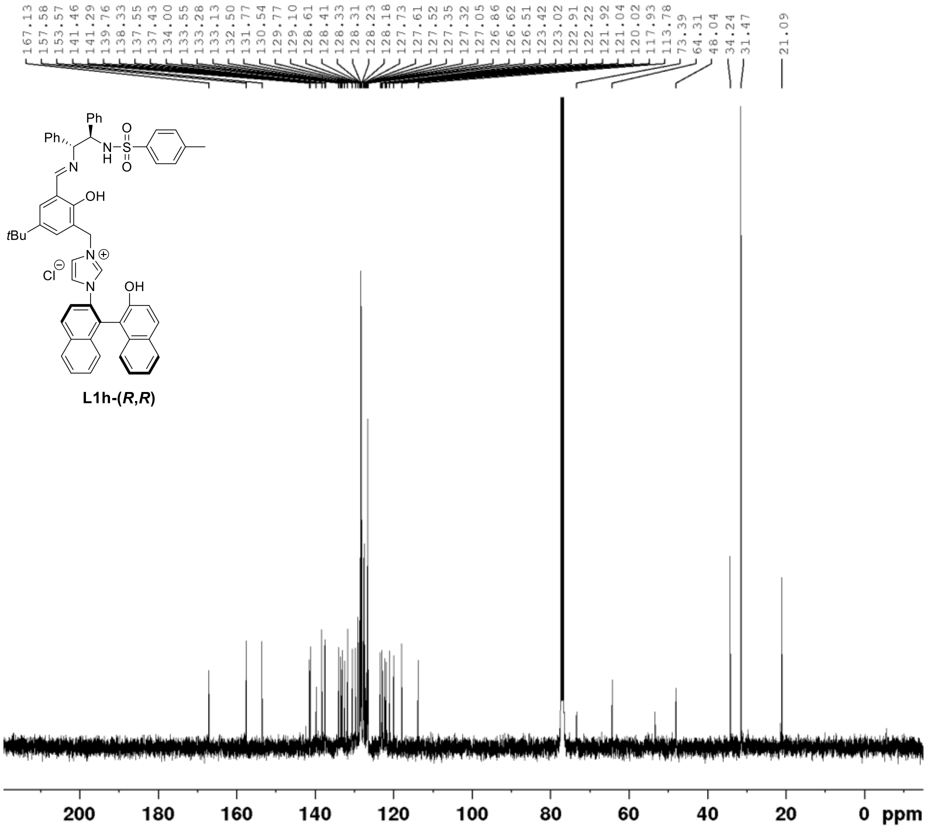

L1h-(*R*,*R*)

```
Current Data Parameters
NAME          38-LS-029
EXPNO               291
PROCNO                1

F2 - Acquisition Parameters
Date_          20160331
Time              23.27
INSTRUM           spect
PROBHD   5 mm PABBO BB/
PULPROG          zgpg30
TD                65536
SOLVENT           CDCl3
NS                 2000
DS                    4
SWH           24038.461 Hz
FIDRES         0.366798 Hz
AQ            1.3631488 sec
RG               205.35
DW               20.800 usec
DE                 6.50 usec
TE                296.0 K
D1           2.00000000 sec
D11          0.03000000 sec
TD0                   1

======== CHANNEL f1 ========
SFO1        100.6152851 MHz
NUC1                13C
P1                10.00 usec
PLW1        48.00000000 W

======== CHANNEL f2 ========
SFO2        400.1016004 MHz
NUC2                 1H
CPDPRG[2        waltz16
PCPD2             90.00 usec
PLW2        12.00000000 W
PLW12        0.27805999 W
PLW13        0.22522999 W

F2 - Processing parameters
SI                32768
SF          100.6052250 MHz
WDW                  EM
SSB      0
LB                 1.00 Hz
GB       0
PC                 1.40
```

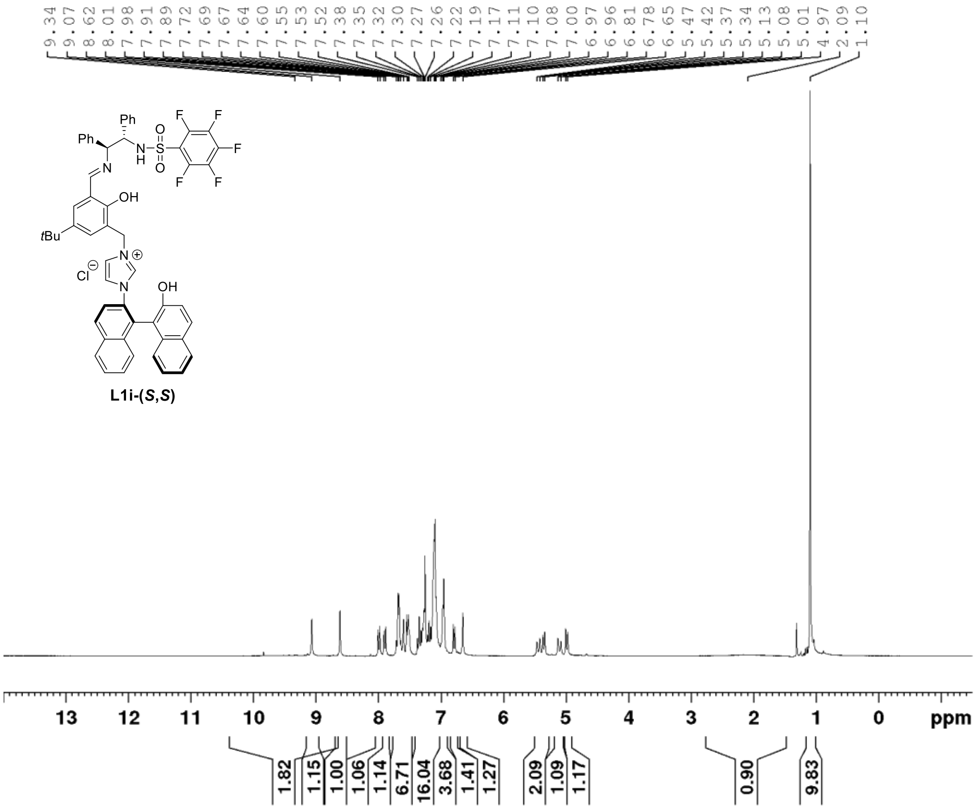


```
Current Data Parameters
NAME     38-LS-034_2019
EXPNO               200
PROCNO                1

F2 - Acquisition Parameters
Date_          20190724
Time              12.58
INSTRUM           spect
PROBHD   5 mm PABBO BB-
PULPROG            zg30
TD                32768
SOLVENT           CDCl3
NS                   16
DS                    2
SWH            6188.119 Hz
FIDRES         0.188846 Hz
AQ            2.6476543 sec
RG                  256
DW               80.800 usec
DE                 8.00 usec
TE                298.0 K
D1           1.00000000 sec
TD0                   1

======== CHANNEL f1 ========
NUC1                 1H
P1                11.05 usec
PL1               -2.00 dB
PL1W        37.02396774 W
SFO1        300.1318534 MHz

F2 - Processing parameters
SI                16384
SF          300.1300066 MHz
WDW                  EM
SSB      0
LB                 0.30 Hz
GB       0
PC                 1.00
```

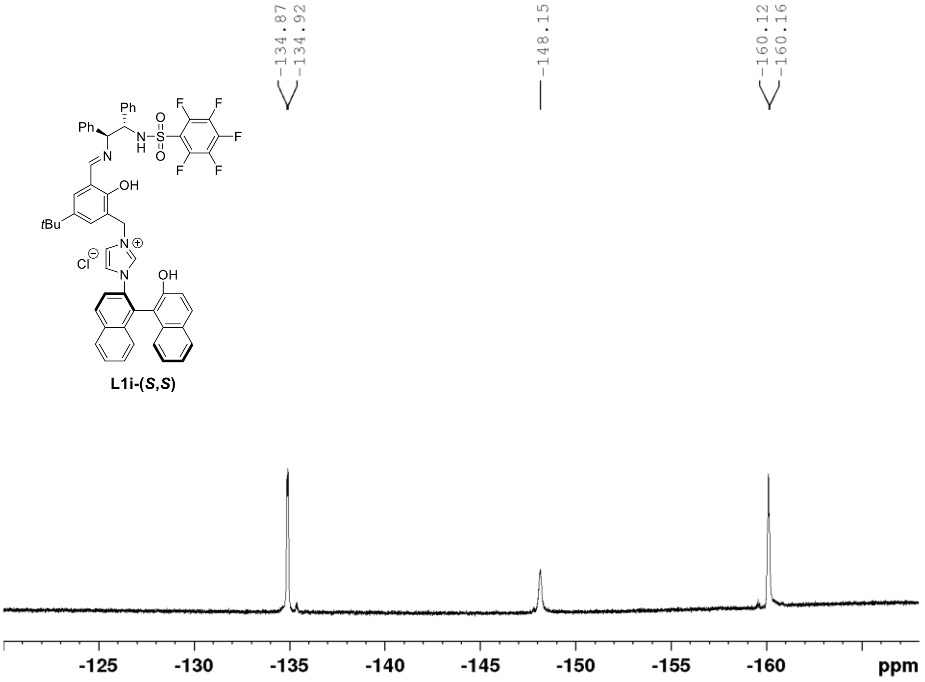


```
Current Data Parameters
NAME        38-LS-034.1
EXPNO               371
PROCNO                1

F2 - Acquisition Parameters
Date_          20160601
Time              14.56
INSTRUM           spect
PROBHD   5 mm PABBO BB/
PULPROG          zgflqn
TD               131072
SOLVENT           CDCl3
NS                   16
DS                    4
SWH           89285.711 Hz
FIDRES         0.681196 Hz
AQ            0.7340032 sec
RG               205.35
DW                5.600 usec
DE                 6.50 usec
TE                296.0 K
D1           1.00000000 sec
TD0                   1

======== CHANNEL f1 ========
SFO1        376.4324910 MHz
NUC1                19F
P1                14.65 usec
PLW1        20.00000000 W

F2 - Processing parameters
SI                65536
SF          376.4701380 MHz
WDW                  EM
SSB      0
LB                 0.30 Hz
GB       0
PC                 1.00
```

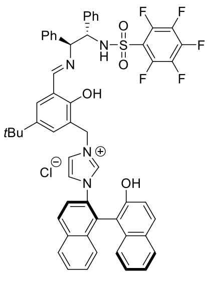

L1i-(*S*,*S*)

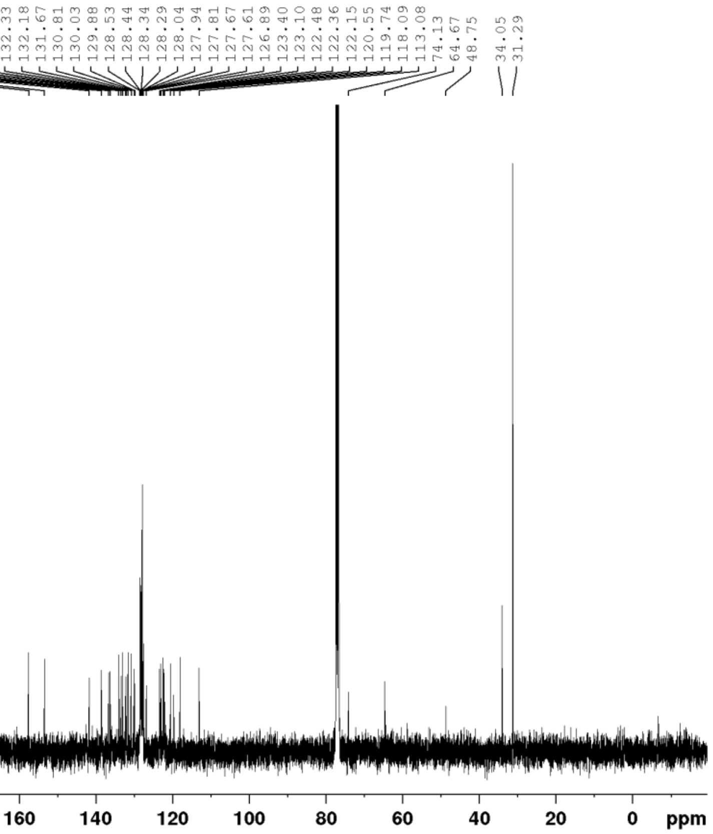

Current Data Parameters
NAME 38-LS-034_2019
EXPNO 453
PROCNO 1

F2 - Acquisition Parameters
Date_ 20190720
Time 5.24
INSTRUM spect
PROBHD 5 mm PABBO BB/
PULPROG zgpg30
TD 65536
SOLVENT CDCl3
NS 2048
DS 4
SWH 24038.461 Hz
FIDRES 0.366798 Hz
AQ 1.3631488 sec
RG 205.35
DW 20.800 usec
DE 6.50 usec
TE 298.0 K
D1 2.00000000 sec
D11 0.03000000 sec
TD0 1

======== CHANNEL f1 ========
SFO1 100.6152851 MHz
NUC1 13C
P1 10.00 usec
PLW1 48.00000000 W

======== CHANNEL f2 ========
SFO2 400.1016004 MHz
NUC2 1H
CPDPRG[2 waltz16
PCPD2 90.00 usec
PLW2 12.00000000 W
PLW12 0.27805999 W
PLW13 0.22522999 W

F2 - Processing parameters
SI 32768
SF 100.6052250 MHz
WDW EM
SSB 0
LB 1.00 Hz
GB 0
PC 1.40

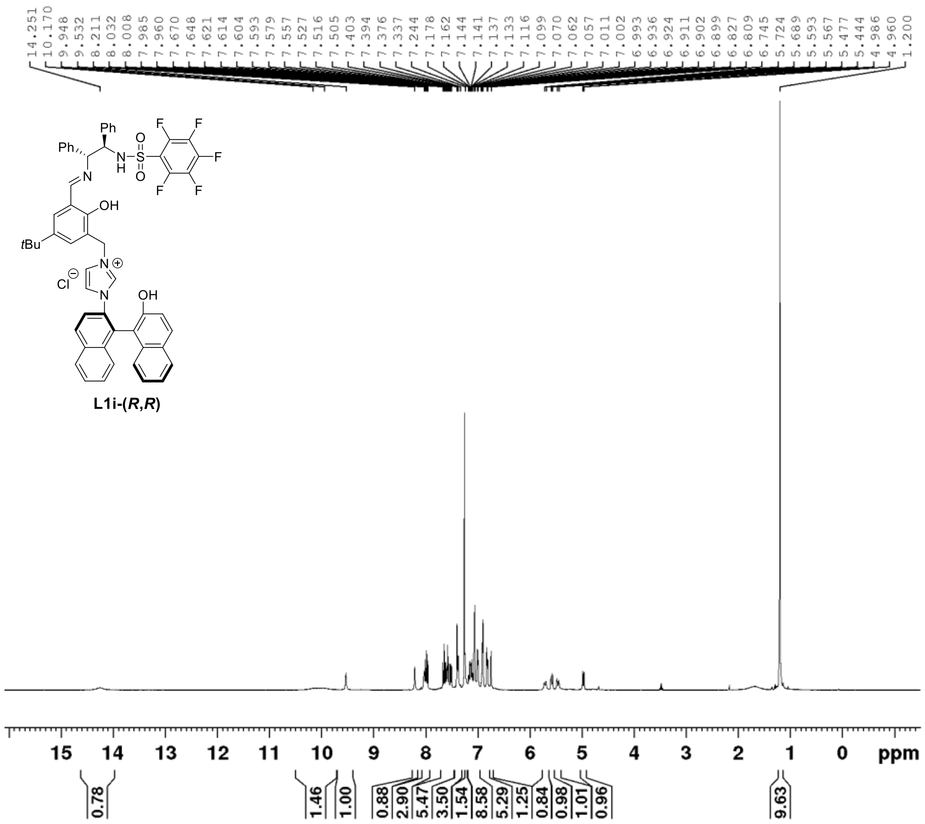


```
Current Data Parameters
NAME          38-LS-033
EXPNO               570
PROCNO                1

F2 - Acquisition Parameters
Date_          20160524
Time              17.14
INSTRUM           spect
PROBHD   5 mm PABBO BB/
PULPROG            zg30
TD                65536
SOLVENT           CDCl3
NS                   16
DS                    2
SWH            8012.820 Hz
FIDRES         0.122266 Hz
AQ            4.0894465 sec
RG               205.35
DW               62.400 usec
DE                 6.50 usec
TE                296.0 K
D1           1.00000000 sec
TD0                   1

======== CHANNEL f1 ========
SFO1        400.1024708 MHz
NUC1                 1H
P1                13.70 usec
PLW1        12.00000000 W

F2 - Processing parameters
SI                65536
SF          400.1000110 MHz
WDW                  EM
SSB      0
LB                 0.30 Hz
GB       0
PC                 1.00
```

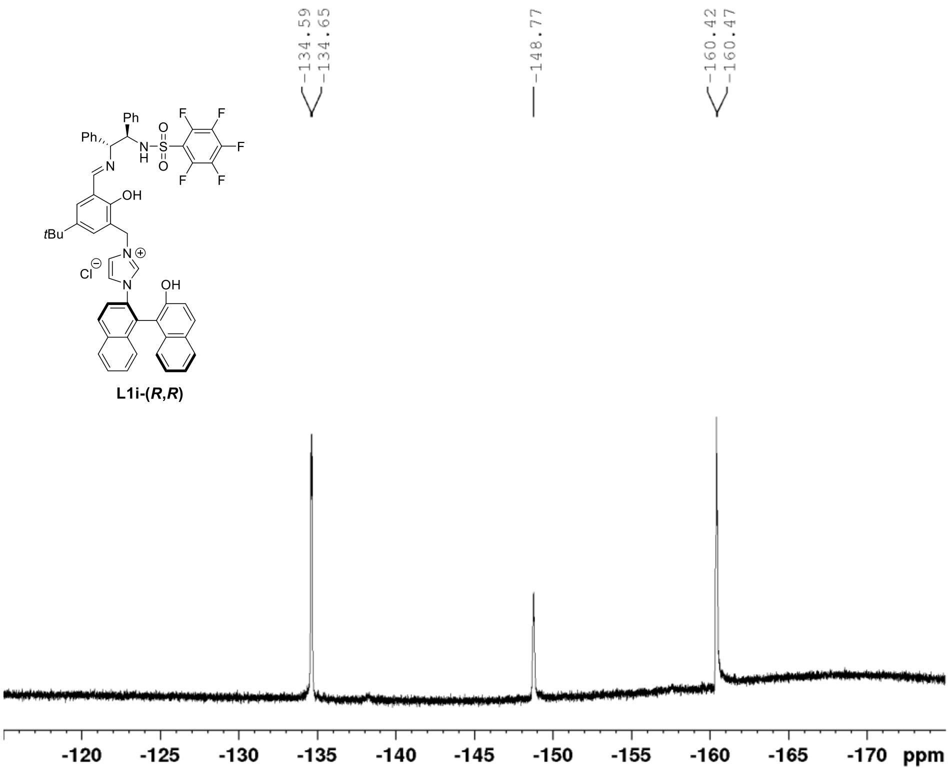


```
Current Data Parameters
NAME          38-LS-033
EXPNO               571
PROCNO                1

F2 - Acquisition Parameters
Date_          20160524
Time              17.16
INSTRUM           spect
PROBHD   5 mm PABBO BB/
PULPROG          zgflqn
TD               131072
SOLVENT           CDCl3
NS                   16
DS                    4
SWH           89285.711 Hz
FIDRES         0.681196 Hz
AQ            0.7340032 sec
RG               205.35
DW                5.600 usec
DE                 6.50 usec
TE                296.0 K
D1           1.00000000 sec
TD0                   1

======== CHANNEL f1 ========
SFO1        376.4324910 MHz
NUC1                19F
P1                14.65 usec
PLW1        20.00000000 W

F2 - Processing parameters
SI                65536
SF          376.4701380 MHz
WDW                  EM
SSB      0
LB                 0.30 Hz
GB       0
PC                 1.00
```

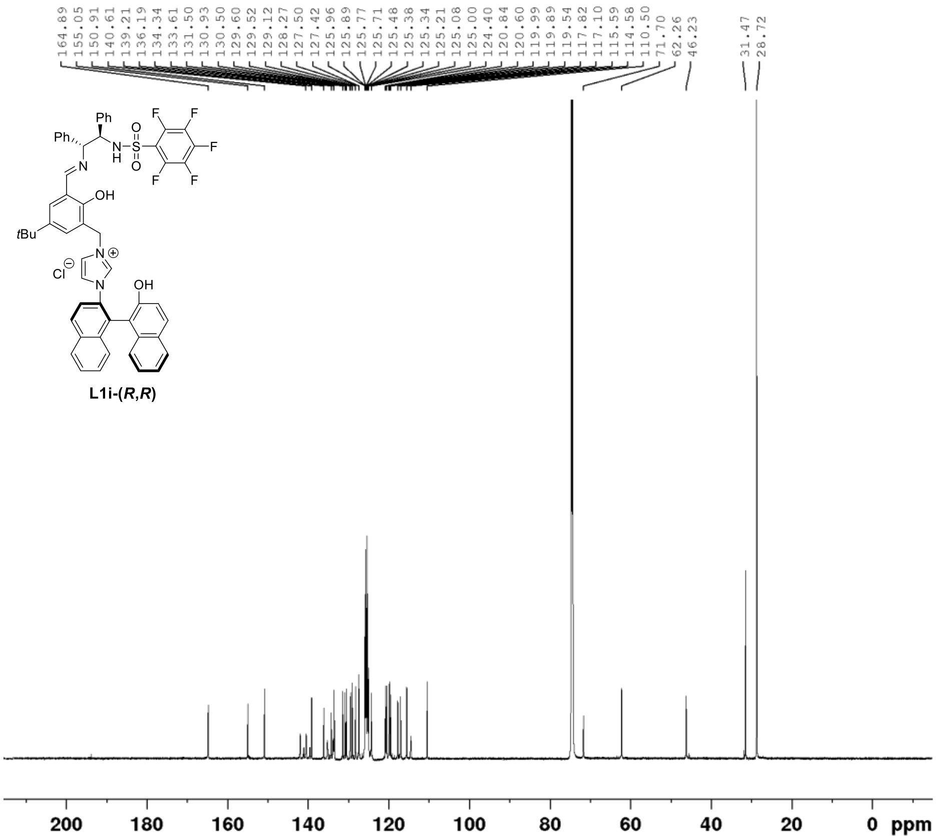

L1i-(*R*,*R*)

```
Current Data Parameters
NAME            38-LS-33
EXPNO                 41
PROCNO                 1

F2 - Acquisition Parameters
Date_           20190804
Time                5.40
INSTRUM            spect
PROBHD   5 mm CPQCI 1H-
PULPROG          zgpg30
TD                 65356
SOLVENT      CH3CN+D2O
NS                 20480
DS                     4
SWH           40760.871 Hz
FIDRES         0.623675 Hz
AQ            0.8017003 sec
RG                182.53
DW                12.267 usec
DE                 18.00 usec
TE                 298.0 K
D1            5.00000000 sec
D11           0.03000000 sec
TD0                    1

======== CHANNEL f1 ========
SFO1        176.1232717 MHz
NUC1                 13C
P1                 12.00 usec
PLW1        105.00000000 W

======== CHANNEL f2 ========
SFO2        700.3628014 MHz
NUC2                  1H
CPDPRG[2         waltz16
PCPD2              65.00 usec
PLW2          9.89999962 W
PLW12         0.15564001 W
PLW13         0.07837200 W

F2 - Processing parameters
SI                131072
SF          176.1056620 MHz
WDW                   EM
SSB      0
LB                  1.00 Hz
GB       0
PC                  1.40
```

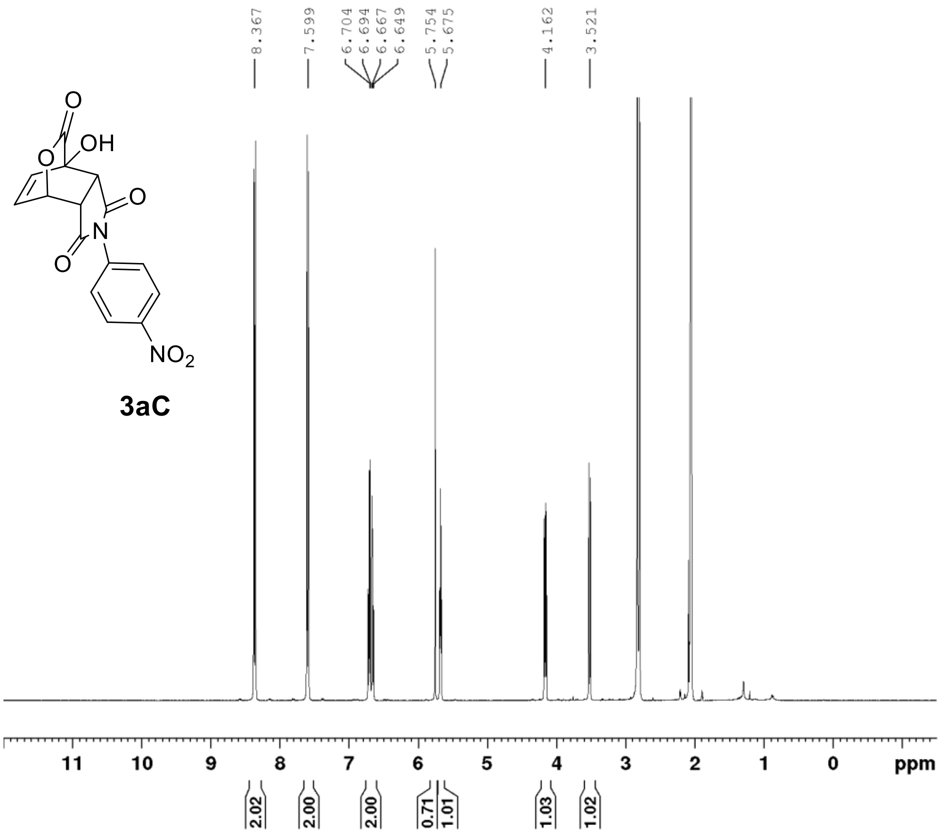


```
Current Data Parameters
NAME        56-HP4-3NO2
EXPNO               122
PROCNO                1

F2 - Acquisition Parameters
Date_          20200126
Time              20.09
INSTRUM           spect
PROBHD   5 mm PABBO BB/
PULPROG            zg30
TD                65536
SOLVENT         Acetone
NS                   64
DS                    2
SWH            8012.820 Hz
FIDRES         0.122266 Hz
AQ            4.0894465 sec
RG               205.35
DW               62.400 usec
DE                 6.50 usec
TE                298.0 K
D1           1.00000000 sec
TD0                   1

======== CHANNEL f1 ========
SFO1        400.1024708 MHz
NUC1                 1H
P1                13.70 usec
PLW1        12.00000000 W

F2 - Processing parameters
SI                65536
SF          400.1000063 MHz
WDW                  EM
SSB      0
LB                 0.30 Hz
GB       0
PC                 1.00
```

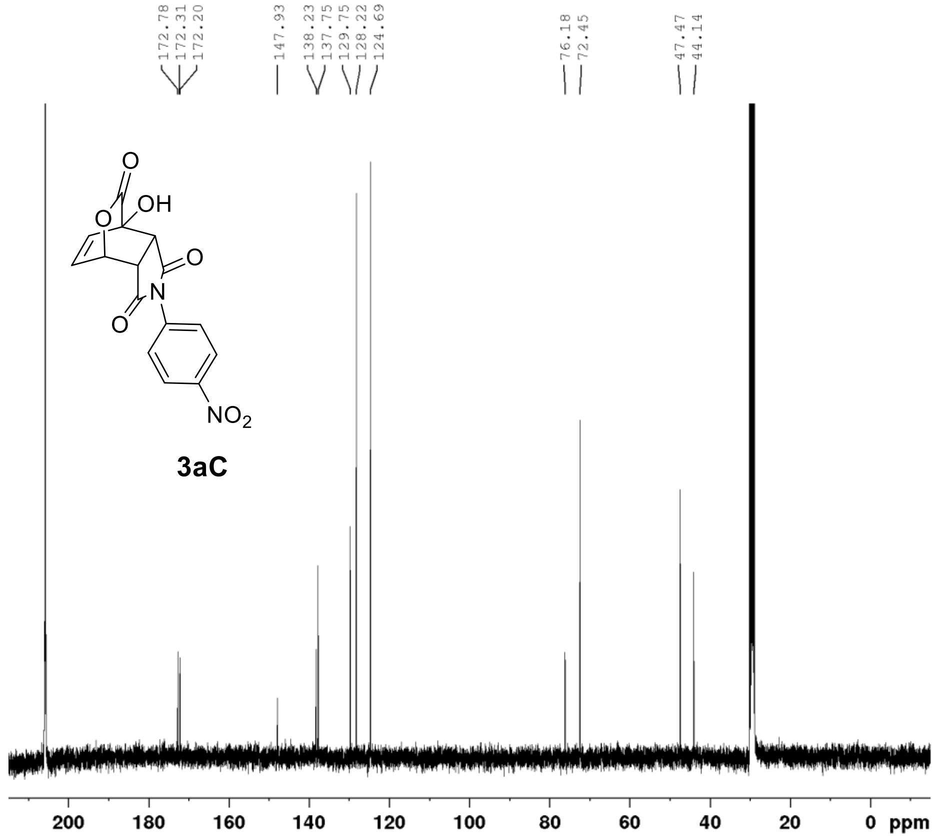


```
Current Data Parameters
NAME        56-HP4-3NO2
EXPNO               123
PROCNO                1

F2 - Acquisition Parameters
Date_          20200126
Time              21.09
INSTRUM           spect
PROBHD   5 mm PABBO BB/
PULPROG          zgpg30
TD                65536
SOLVENT         Acetone
NS                 1024
DS                    4
SWH           24038.461 Hz
FIDRES         0.366798 Hz
AQ            1.3631488 sec
RG               205.35
DW               20.800 usec
DE                 6.50 usec
TE                298.0 K
D1           2.00000000 sec
D11          0.03000000 sec
TD0                   1

======== CHANNEL f1 ========
SFO1        100.6152851 MHz
NUC1                13C
P1                10.00 usec
PLW1        48.00000000 W

======== CHANNEL f2 ========
SFO2        400.1016004 MHz
NUC2                 1H
CPDPRG[2        waltz16
PCPD2             90.00 usec
PLW2        12.00000000 W
PLW12        0.27805999 W
PLW13        0.22522999 W

F2 - Processing parameters
SI                32768
SF          100.6051675 MHz
WDW                  EM
SSB      0
LB                 1.00 Hz
GB       0
PC                 1.40
```

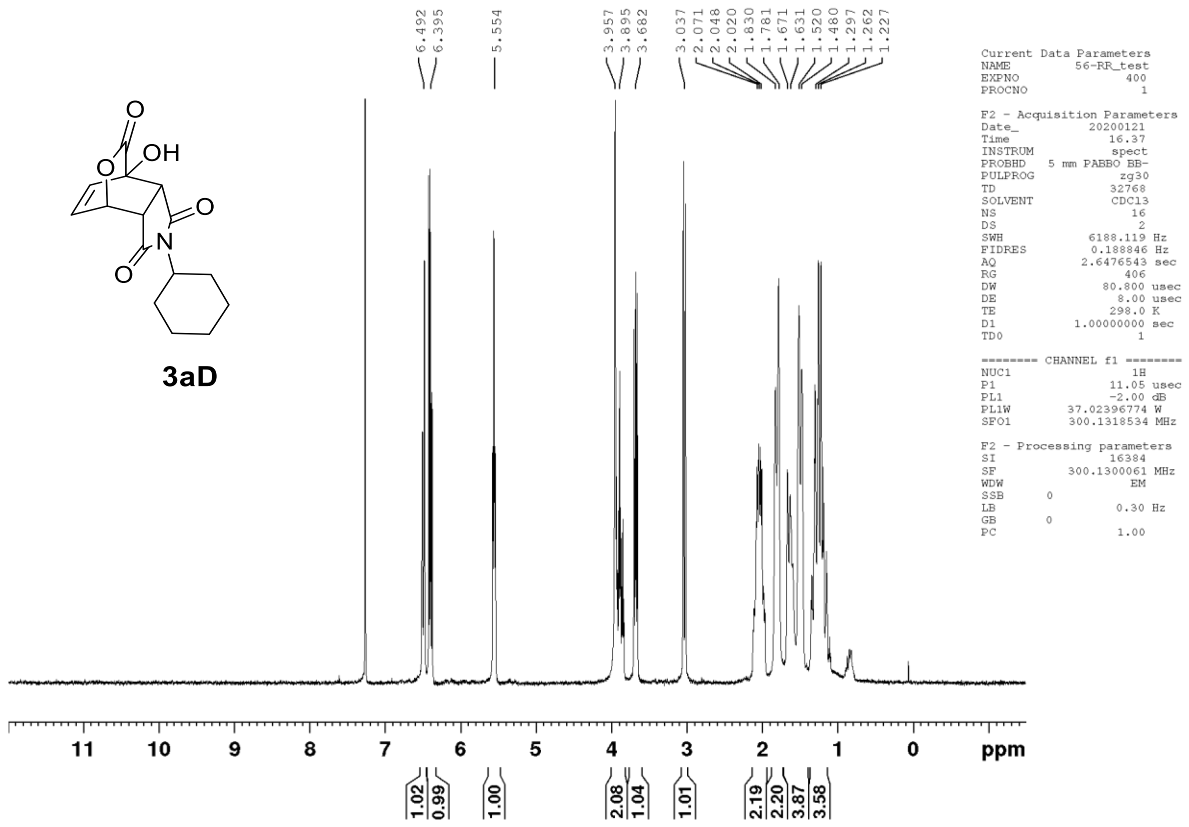
6.492
6.395
5.554
3.957
3.895
3.682
3.037
2.071
2.048
2.020
1.830
1.781
1.671
1.631
1.520
1.480
1.297
1.262
1.227
3aD
11 10 9 8 7 6 5 4 3 2 1 0 ppm
1.02
0.99
1.00
2.08
1.04
1.01
2.19
2.20
3.87
3.58
Current Data Parameters
NAME 56-RR_test
EXPNO 400
PROCNO 1
F2 - Acquisition Parameters
Date_ 20200121
Time 16.37
INSTRUM spect
PROBHD 5 mm PABBO BB-
PULPROG zg30
TD 32768
SOLVENT CDCl3
NS 16
DS 2
SWH 6188.119 Hz
FIDRES 0.188846 Hz
AQ 2.6476543 sec
RG 406
DW 80.800 usec
DE 8.00 usec
TE 298.0 K
D1 1.00000000 sec
TD0 1
======== CHANNEL f1 ========
NUC1 1H
P1 11.05 usec
PL1 -2.00 dB
PL1W 37.02396774 W
SFO1 300.1318534 MHz
F2 - Processing parameters
SI 16384
SF 300.1300061 MHz
WDW EM
SSB 0
LB 0.30 Hz
GB 0
PC 1.00

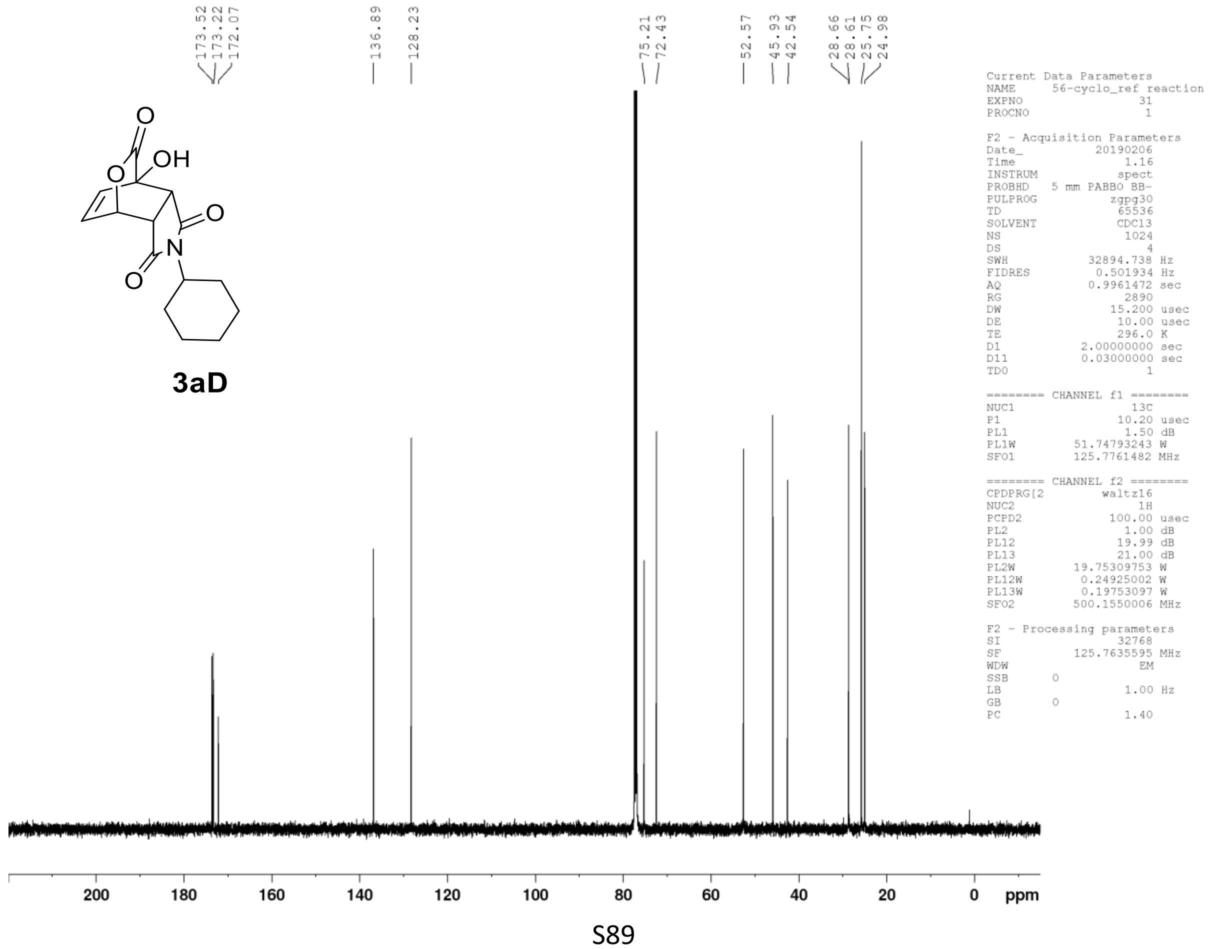
173.52
173.22
172.07
136.89
128.23
75.21
72.43
52.57
45.93
42.54
28.66
28.61
25.75
24.98
3aD
200 180 160 140 120 100 80 60 40 20 0 ppm
Current Data Parameters
NAME 56-cyclo_ref reaction
EXPNO 31
PROCNO 1
F2 - Acquisition Parameters
Date_ 20190206
Time 1.16
INSTRUM spect
PROBHD 5 mm PABBO BB-
PULPROG zgpg30
TD 65536
SOLVENT CDCl3
NS 1024
DS 4
SWH 32894.738 Hz
FIDRES 0.501934 Hz
AQ 0.9961472 sec
RG 2890
DW 15.200 usec
DE 10.00 usec
TE 296.0 K
D1 2.00000000 sec
D11 0.03000000 sec
TD0 1
======== CHANNEL f1 ========
NUC1 13C
P1 10.20 usec
PL1 1.50 dB
PL1W 51.74793243 W
SFO1 125.7761482 MHz
======== CHANNEL f2 ========
CPDPRG[2 waltz16
NUC2 1H
PCPD2 100.00 usec
PL2 1.00 dB
PL12 19.99 dB
PL13 21.00 dB
PL2W 19.75309753 W
PL12W 0.24925002 W
PL13W 0.19753097 W
SFO2 500.1550006 MHz
F2 - Processing parameters
SI 32768
SF 125.7635595 MHz
WDW EM
SSB 0
LB 1.00 Hz
GB 0
PC 1.40

3aE

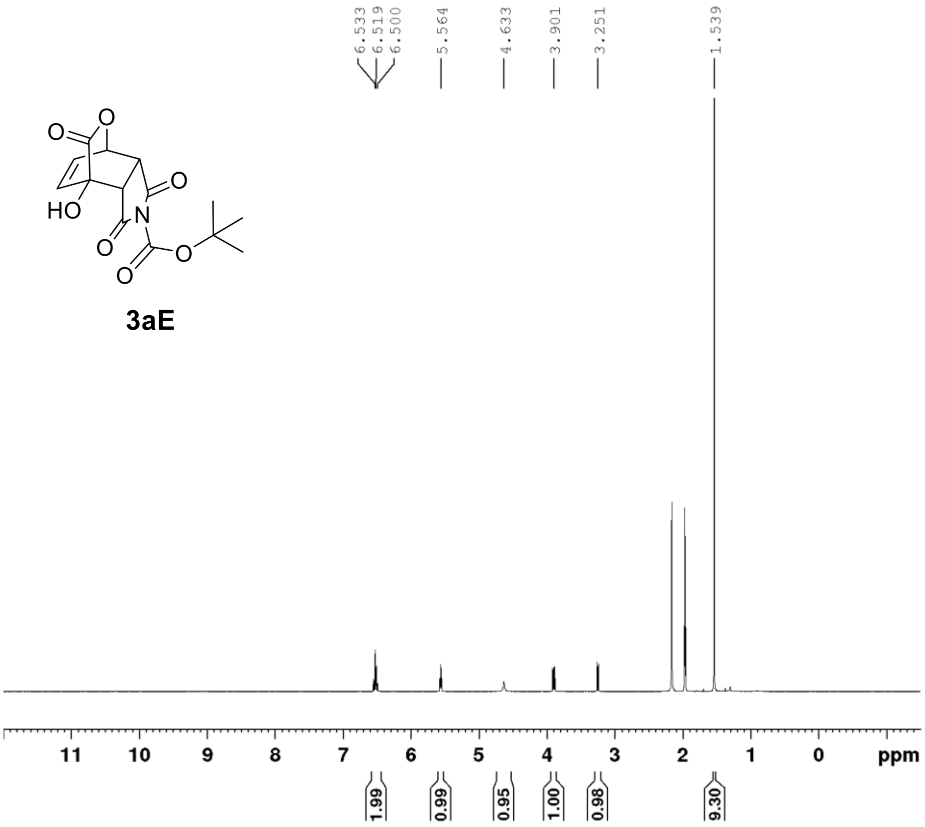


```
Current Data Parameters
NAME      56-Boc_ref-reac_pure
EXPNO              540
PROCNO               1

F2 - Acquisition Parameters
Date_          20200123
Time              19.38
INSTRUM           spect
PROBHD   5 mm PABBO BB/
PULPROG            zg30
TD                65536
SOLVENT            CD3CN
NS                   16
DS                    2
SWH            8012.820 Hz
FIDRES         0.122266 Hz
AQ            4.0894465 sec
RG               205.35
DW               62.400 usec
DE                 6.50 usec
TE                298.0 K
D1           1.00000000 sec
TD0                   1

======== CHANNEL f1 ========
SFO1        400.1024708 MHz
NUC1                 1H
P1                13.70 usec
PLW1        12.00000000 W

F2 - Processing parameters
SI                65536
SF          400.1000000 MHz
WDW                  EM
SSB      0
LB                 0.30 Hz
GB       0
PC                 1.00
```

3aE

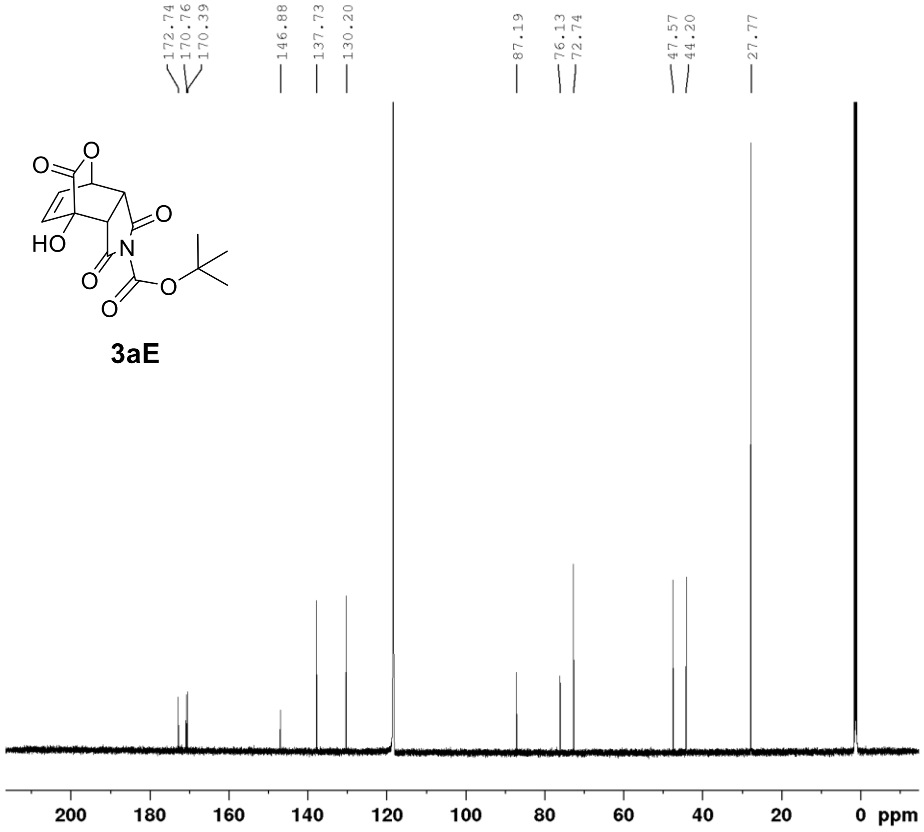


```
Current Data Parameters
NAME           56_Boc_13C
EXPNO                30
PROCNO                1

F2 - Acquisition Parameters
Date_          20200124
Time              13.43
INSTRUM           spect
PROBHD   5 mm CPQCI 1H-
PULPROG          zgpg30
TD                65356
SOLVENT           CD3CN
NS                 1024
DS                    4
SWH           40760.871 Hz
FIDRES         0.623675 Hz
AQ            0.8017003 sec
RG               182.53
DW               12.267 usec
DE                18.00 usec
TE                298.0 K
D1           2.00000000 sec
D11          0.03000000 sec
TD0                   1

======== CHANNEL f1 ========
SFO1        176.1232717 MHz
NUC1                13C
P1                12.00 usec
PLW1       105.00000000 W

======== CHANNEL f2 ========
SFO2        700.3628014 MHz
NUC2                 1H
CPDPRG[2         waltz16
PCPD2             65.00 usec
PLW2         9.89999962 W
PLW12        0.15564001 W
PLW13        0.07837200 W

F2 - Processing parameters
SI               131072
SF          176.1054948 MHz
WDW                  EM
SSB      0
LB                 1.00 Hz
GB       0
PC                 1.40
```

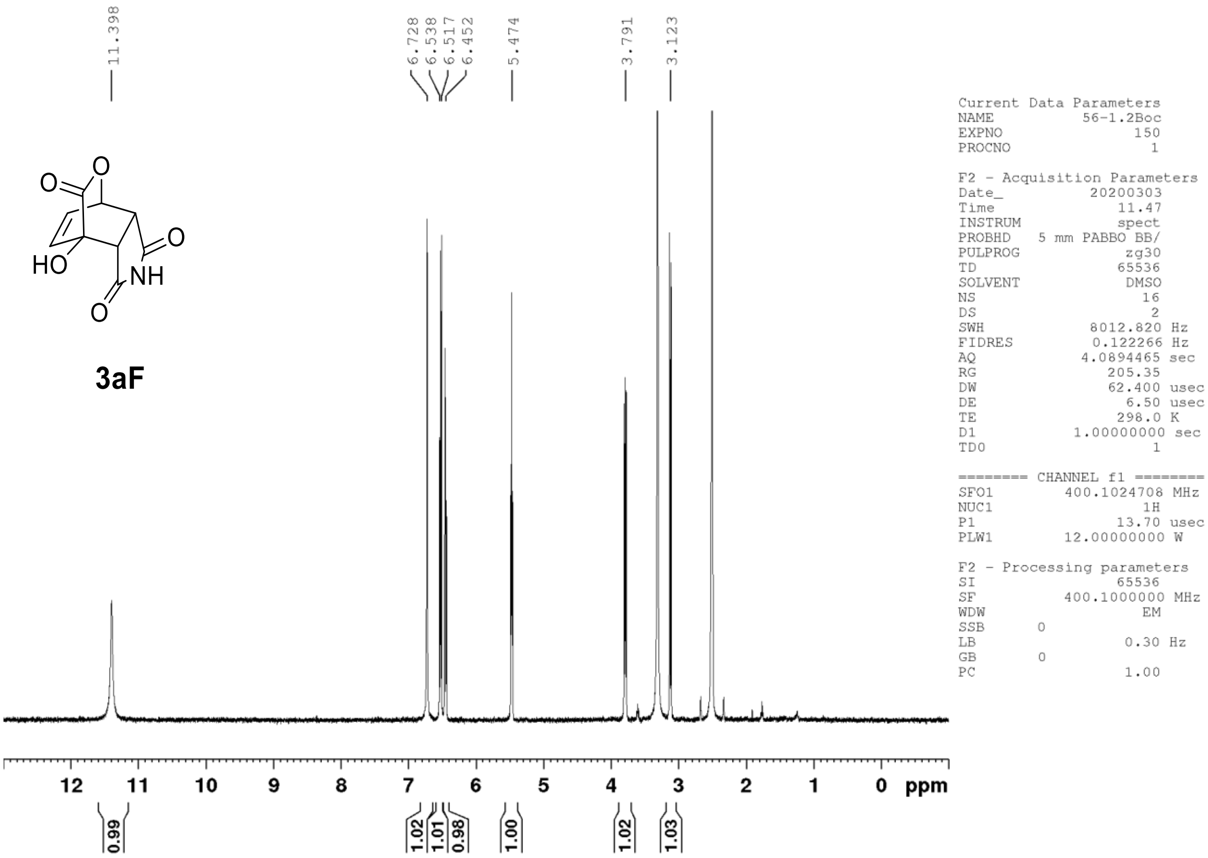
11.398
6.728
6.538
6.517
6.452
5.474
3.791
3.123
3aF
HO
NH
12 11 10 9 8 7 6 5 4 3 2 1 0 ppm
0.99
1.02
1.01
0.98
1.00
1.02
1.03
Current Data Parameters
NAME 56-1.2Boc
EXPNO 150
PROCNO 1
F2 - Acquisition Parameters
Date_ 20200303
Time 11.47
INSTRUM spect
PROBHD 5 mm PABBO BB/
PULPROG zg30
TD 65536
SOLVENT DMSO
NS 16
DS 2
SWH 8012.820 Hz
FIDRES 0.122266 Hz
AQ 4.0894465 sec
RG 205.35
DW 62.400 usec
DE 6.50 usec
TE 298.0 K
D1 1.00000000 sec
TD0 1
======== CHANNEL f1 ========
SFO1 400.1024708 MHz
NUC1 1H
P1 13.70 usec
PLW1 12.00000000 W
F2 - Processing parameters
SI 65536
SF 400.1000000 MHz
WDW EM
SSB 0
LB 0.30 Hz
GB 0
PC 1.00

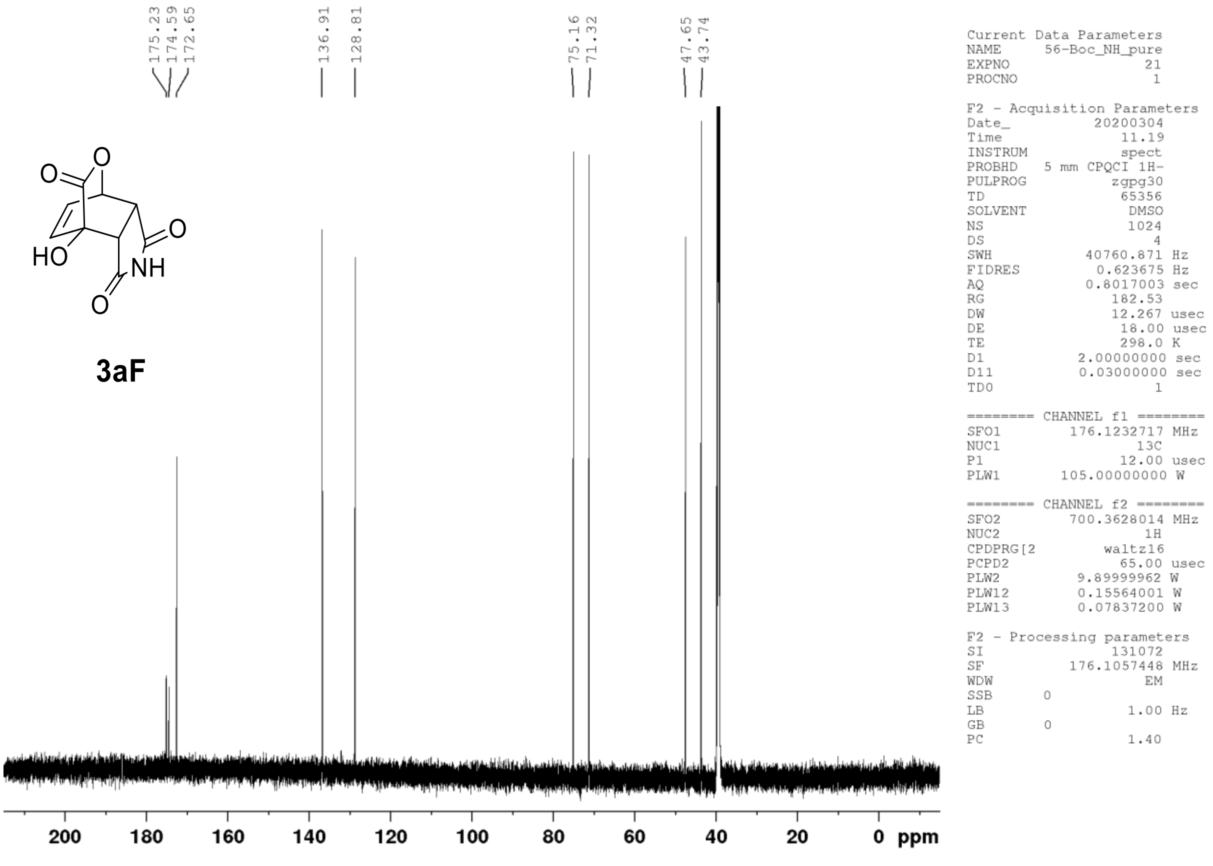
175.23
174.59
172.65
136.91
128.81
75.16
71.32
47.65
43.74
3aF
HO
NH
200 180 160 140 120 100 80 60 40 20 0 ppm
Current Data Parameters
NAME 56-Boc_NH_pure
EXPNO 21
PROCNO 1
F2 - Acquisition Parameters
Date_ 20200304
Time 11.19
INSTRUM spect
PROBHD 5 mm CPQCI 1H-
PULPROG zgpg30
TD 65356
SOLVENT DMSO
NS 1024
DS 4
SWH 40760.871 Hz
FIDRES 0.623675 Hz
AQ 0.8017003 sec
RG 182.53
DW 12.267 usec
DE 18.00 usec
TE 298.0 K
D1 2.00000000 sec
D11 0.03000000 sec
TD0 1
======== CHANNEL f1 ========
SFO1 176.1232717 MHz
NUC1 13C
P1 12.00 usec
PLW1 105.00000000 W
======== CHANNEL f2 ========
SFO2 700.3628014 MHz
NUC2 1H
CPDPRG[2 waltz16
PCPD2 65.00 usec
PLW2 9.89999962 W
PLW12 0.15564001 W
PLW13 0.07837200 W
F2 - Processing parameters
SI 131072
SF 176.1057448 MHz
WDW EM
SSB 0
LB 1.00 Hz
GB 0
PC 1.40

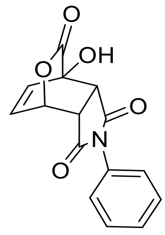
O
OH
O
O
N
O

3aG

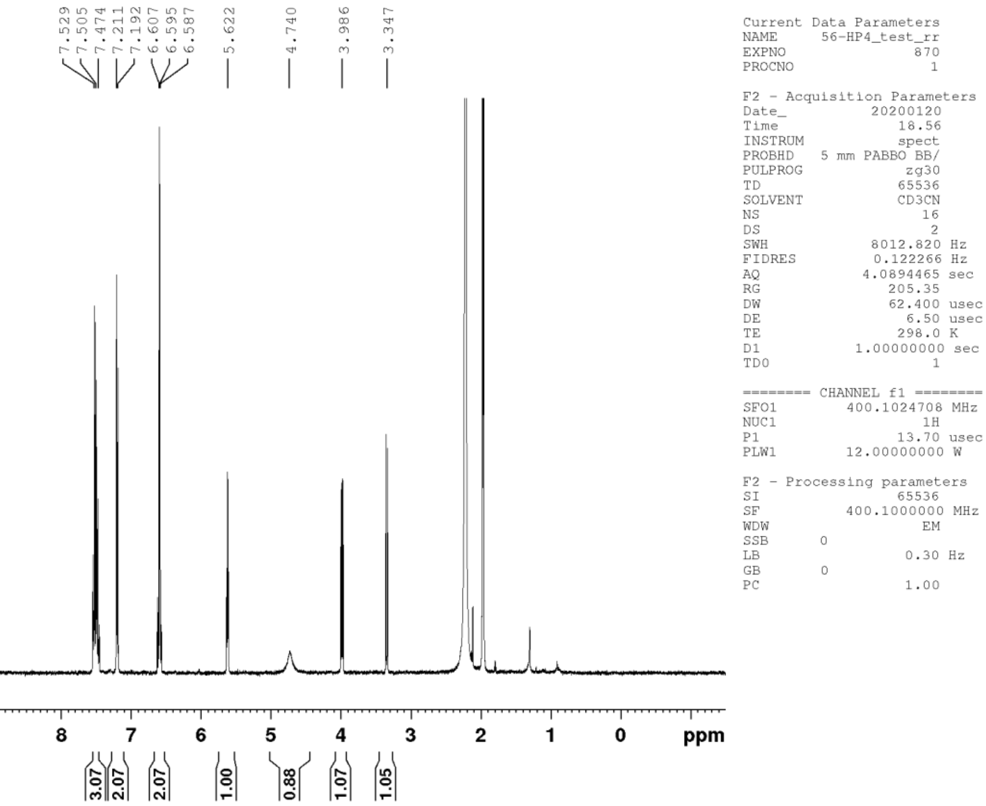
7.529
7.505
7.474
7.211
7.192
6.607
6.595
6.587
5.622
4.740
3.986
3.347
11 10 9 8 7 6 5 4 3 2 1 0 ppm
3.07
2.07
2.07
1.00
0.88
1.07
1.05
Current Data Parameters
NAME 56-HP4_test_rr
EXPNO 870
PROCNO 1
F2 - Acquisition Parameters
Date_ 20200120
Time 18.56
INSTRUM spect
PROBHD 5 mm PABBO BB/
PULPROG zg30
TD 65536
SOLVENT CD3CN
NS 16
DS 2
SWH 8012.820 Hz
FIDRES 0.122266 Hz
AQ 4.0894465 sec
RG 205.35
DW 62.400 usec
DE 6.50 usec
TE 298.0 K
D1 1.00000000 sec
TD0 1
======== CHANNEL f1 ========
SFO1 400.1024708 MHz
NUC1 1H
P1 13.70 usec
PLW1 12.00000000 W
F2 - Processing parameters
SI 65536
SF 400.1000000 MHz
WDW EM
SSB 0
LB 0.30 Hz
GB 0
PC 1.00

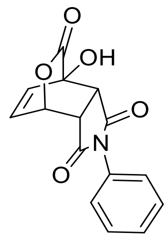
O
OH
O
O
N
O

3aG

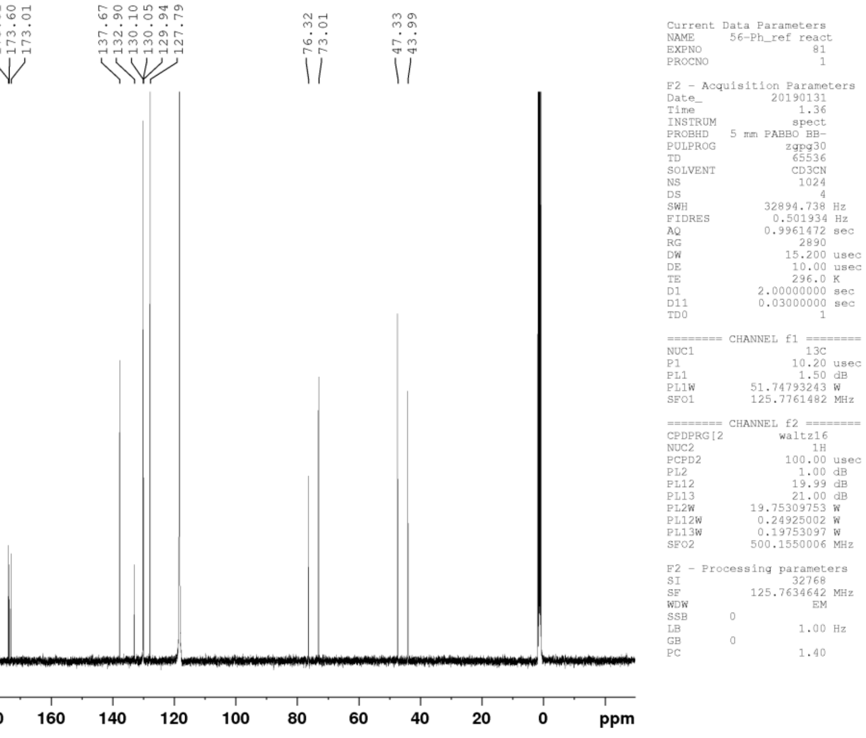
173.81
173.60
173.01
137.67
132.90
130.10
130.05
129.94
127.79
76.32
73.01
47.33
43.99
220 200 180 160 140 120 100 80 60 40 20 0 ppm
Current Data Parameters
NAME 56-Ph_ref react
EXPNO 81
PROCNO 1
F2 - Acquisition Parameters
Date_ 20190131
Time 1.36
INSTRUM spect
PROBHD 5 mm PABBO BB-
PULPROG zgpg30
TD 65536
SOLVENT CD3CN
NS 1024
DS 4
SWH 32894.738 Hz
FIDRES 0.501934 Hz
AQ 0.9961472 sec
RG 2890
DW 15.200 usec
DE 10.00 usec
TE 296.0 K
D1 2.00000000 sec
D11 0.03000000 sec
TD0 1
======== CHANNEL f1 ========
NUC1 13C
P1 10.20 usec
PL1 1.50 dB
PL1W 51.74793243 W
SFO1 125.7761482 MHz
======== CHANNEL f2 ========
CPDPRG[2 waltz16
NUC2 1H
PCPD2 100.00 usec
PL2 1.00 dB
PL12 19.99 dB
PL13 21.00 dB
PL2W 19.75309753 W
PL12W 0.24925002 W
PL13W 0.19753097 W
SFO2 500.1550006 MHz
F2 - Processing parameters
SI 32768
SF 125.7634642 MHz
WDW EM
SSB 0
LB 1.00 Hz
GB 0
PC 1.40

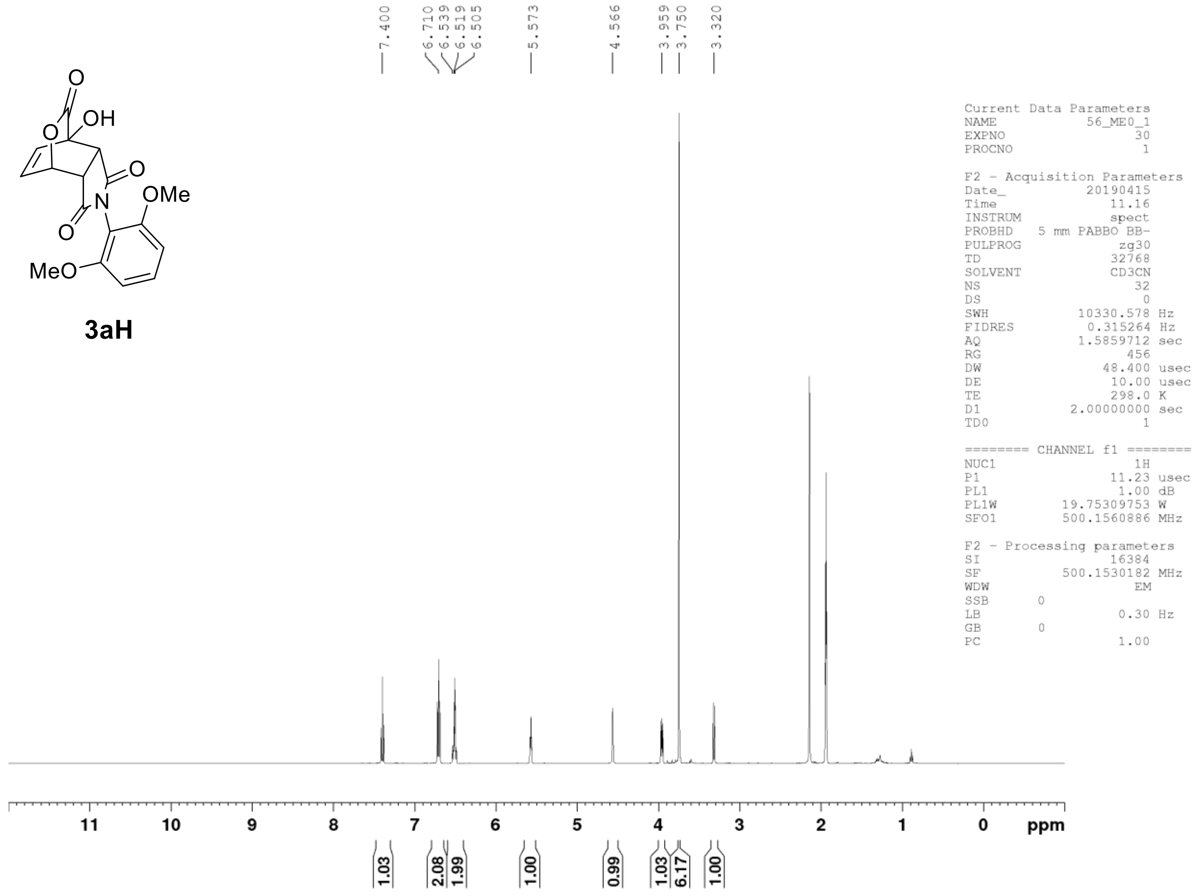
7.400
6.710
6.539
6.519
6.505
5.573
4.566
3.959
3.750
3.320
OH
O
N
OMe
MeO
3aH
Current Data Parameters
NAME 56_ME0_1
EXPNO 30
PROCNO 1
F2 - Acquisition Parameters
Date_ 20190415
Time 11.16
INSTRUM spect
PROBHD 5 mm PABBO BB-
PULPROG zg30
TD 32768
SOLVENT CD3CN
NS 32
DS 0
SWH 10330.578 Hz
FIDRES 0.315264 Hz
AQ 1.5859712 sec
RG 456
DW 48.400 usec
DE 10.00 usec
TE 298.0 K
D1 2.00000000 sec
TD0 1
======== CHANNEL f1 ========
NUC1 1H
P1 11.23 usec
PL1 1.00 dB
PL1W 19.75309753 W
SFO1 500.1560886 MHz
F2 - Processing parameters
SI 16384
SF 500.1530182 MHz
WDW EM
SSB 0
LB 0.30 Hz
GB 0
PC 1.00
11 10 9 8 7 6 5 4 3 2 1 0 ppm
1.03
2.08
1.99
1.00
0.99
1.03
6.17
1.00

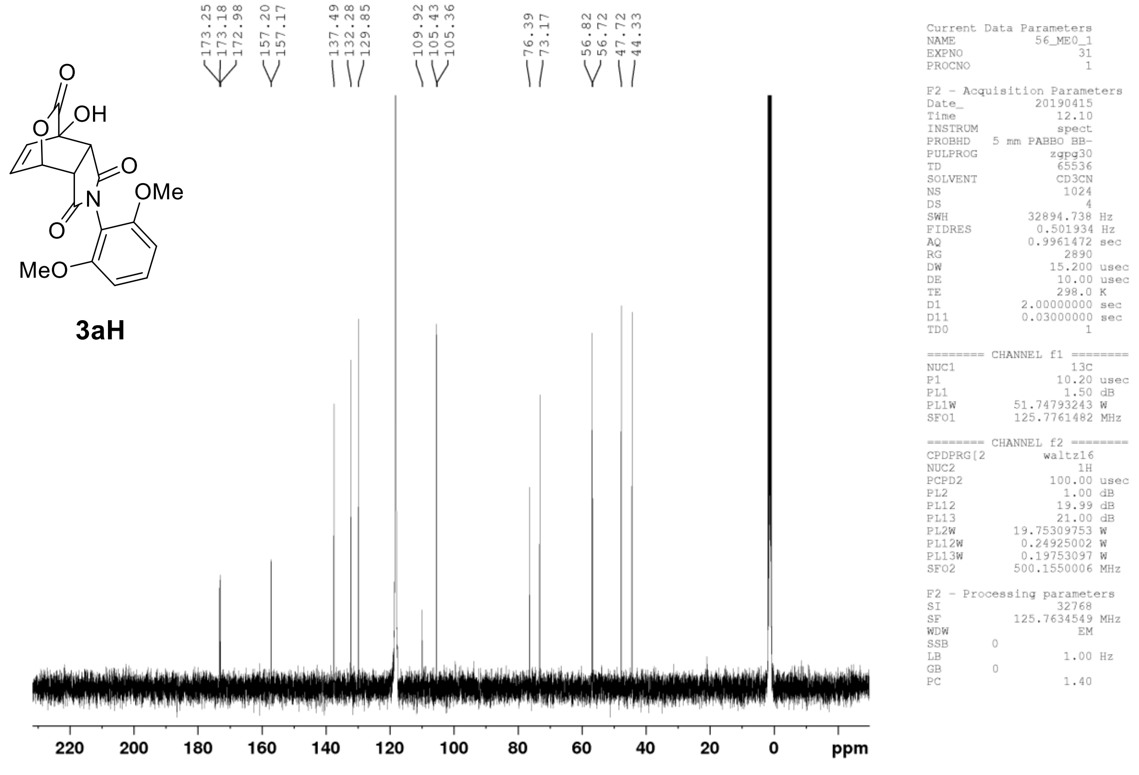
173.25
173.18
172.98
157.20
157.17
137.49
132.28
129.85
109.92
105.43
105.36
76.39
73.17
56.82
56.72
47.72
44.33
OH
O
N
OMe
MeO
3aH
Current Data Parameters
NAME 56_ME0_1
EXPNO 31
PROCNO 1
F2 - Acquisition Parameters
Date_ 20190415
Time 12.10
INSTRUM spect
PROBHD 5 mm PABBO BB-
PULPROG zgpg30
TD 65536
SOLVENT CD3CN
NS 1024
DS 4
SWH 32894.738 Hz
FIDRES 0.501934 Hz
AQ 0.9961472 sec
RG 2890
DW 15.200 usec
DE 10.00 usec
TE 298.0 K
D1 2.00000000 sec
D11 0.03000000 sec
TD0 1
======== CHANNEL f1 ========
NUC1 13C
P1 10.20 usec
PL1 1.50 dB
PL1W 51.74793243 W
SFO1 125.7761482 MHz
======== CHANNEL f2 ========
CPDPRG[2 waltz16
NUC2 1H
PCPD2 100.00 usec
PL2 1.00 dB
PL12 19.99 dB
PL13 21.00 dB
PL2W 19.75309753 W
PL12W 0.24925002 W
PL13W 0.19753097 W
SFO2 500.1550006 MHz
F2 - Processing parameters
SI 32768
SF 125.7634549 MHz
WDW EM
SSB 0
LB 1.00 Hz
GB 0
PC 1.40
220 200 180 160 140 120 100 80 60 40 20 0 ppm

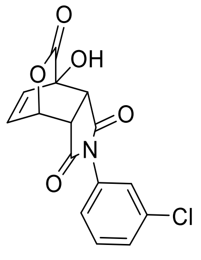

3al

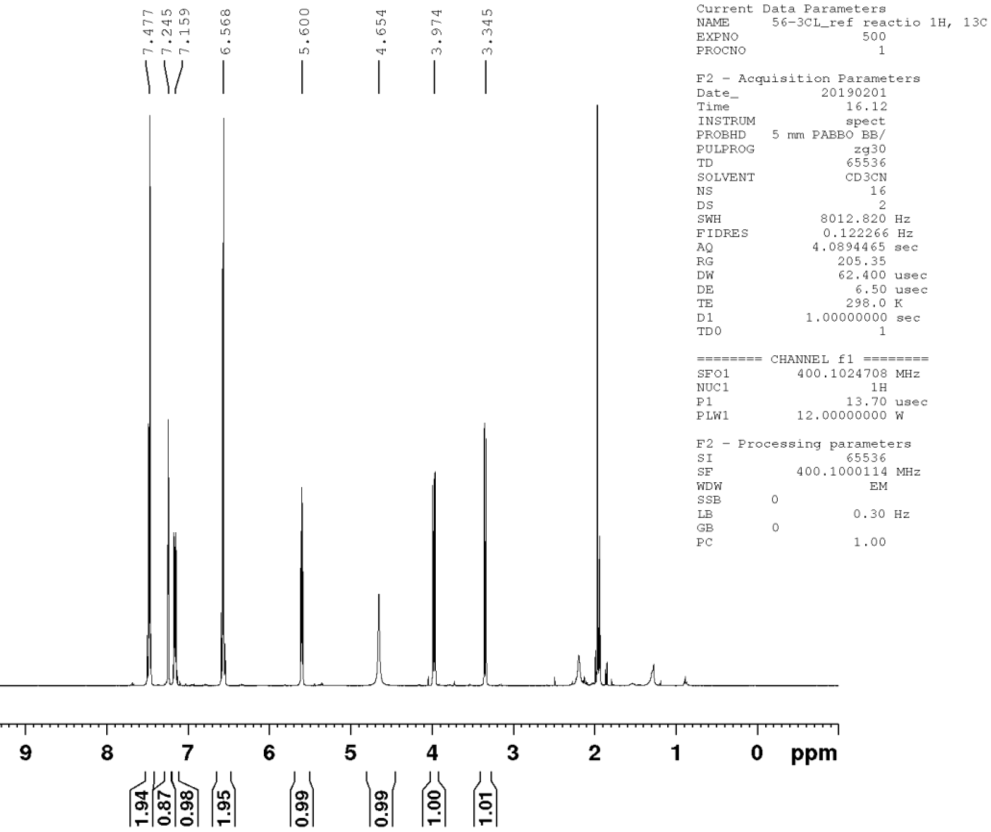
7.477
7.245
7.159
6.568
5.600
4.654
3.974
3.345
11 10 9 8 7 6 5 4 3 2 1 0 ppm
1.94
0.87
0.98
1.95
0.99
0.99
1.00
1.01
Current Data Parameters
NAME 56-3CL_ref reactio 1H, 13C
EXPNO 500
PROCNO 1
F2 - Acquisition Parameters
Date_ 20190201
Time 16.12
INSTRUM spect
PROBHD 5 mm PABBO BB/
PULPROG zg30
TD 65536
SOLVENT CD3CN
NS 16
DS 2
SWH 8012.820 Hz
FIDRES 0.122266 Hz
AQ 4.0894465 sec
RG 205.35
DW 62.400 usec
DE 6.50 usec
TE 298.0 K
D1 1.00000000 sec
TD0 1
======== CHANNEL f1 ========
SFO1 400.1024708 MHz
NUC1 1H
P1 13.70 usec
PLW1 12.00000000 W
F2 - Processing parameters
SI 65536
SF 400.1000114 MHz
WDW EM
SSB 0
LB 0.30 Hz
GB 0
PC 1.00

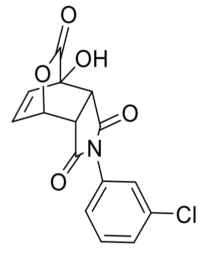

3al

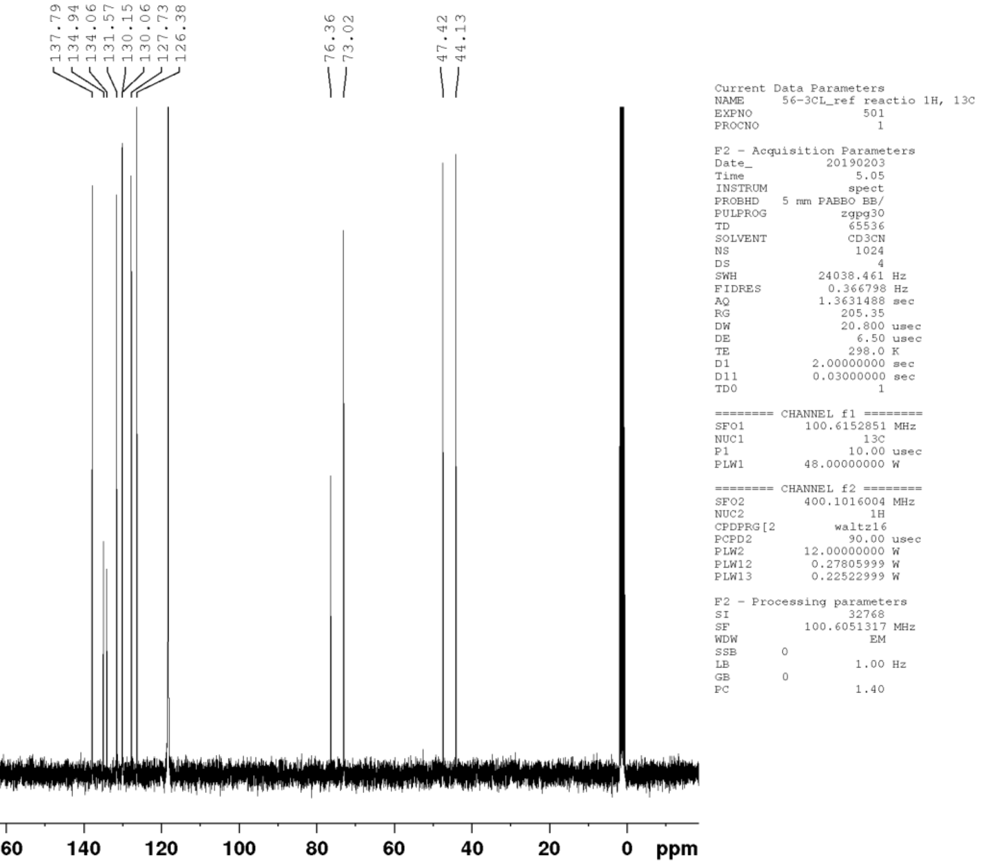
173.47
173.27
173.00
137.79
134.94
134.06
131.57
130.15
130.06
127.73
126.38
76.36
73.02
47.42
44.13
200 180 160 140 120 100 80 60 40 20 0 ppm
Current Data Parameters
NAME 56-3CL_ref reactio 1H, 13C
EXPNO 501
PROCNO 1
F2 - Acquisition Parameters
Date_ 20190203
Time 5.05
INSTRUM spect
PROBHD 5 mm PABBO BB/
PULPROG zgpg30
TD 65536
SOLVENT CD3CN
NS 1024
DS 4
SWH 24038.461 Hz
FIDRES 0.366798 Hz
AQ 1.3631488 sec
RG 205.35
DW 20.800 usec
DE 6.50 usec
TE 298.0 K
D1 2.00000000 sec
D11 0.03000000 sec
TD0 1
======== CHANNEL f1 ========
SFO1 100.6152851 MHz
NUC1 13C
P1 10.00 usec
PLW1 48.00000000 W
======== CHANNEL f2 ========
SFO2 400.1016004 MHz
NUC2 1H
CPDPRG[2 waltz16
PCPD2 90.00 usec
PLW2 12.00000000 W
PLW12 0.27805999 W
PLW13 0.22522999 W
F2 - Processing parameters
SI 32768
SF 100.6051317 MHz
WDW EM
SSB 0
LB 1.00 Hz
GB 0
PC 1.40

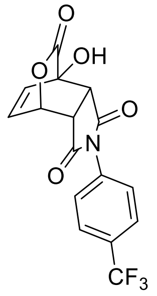

3aJ

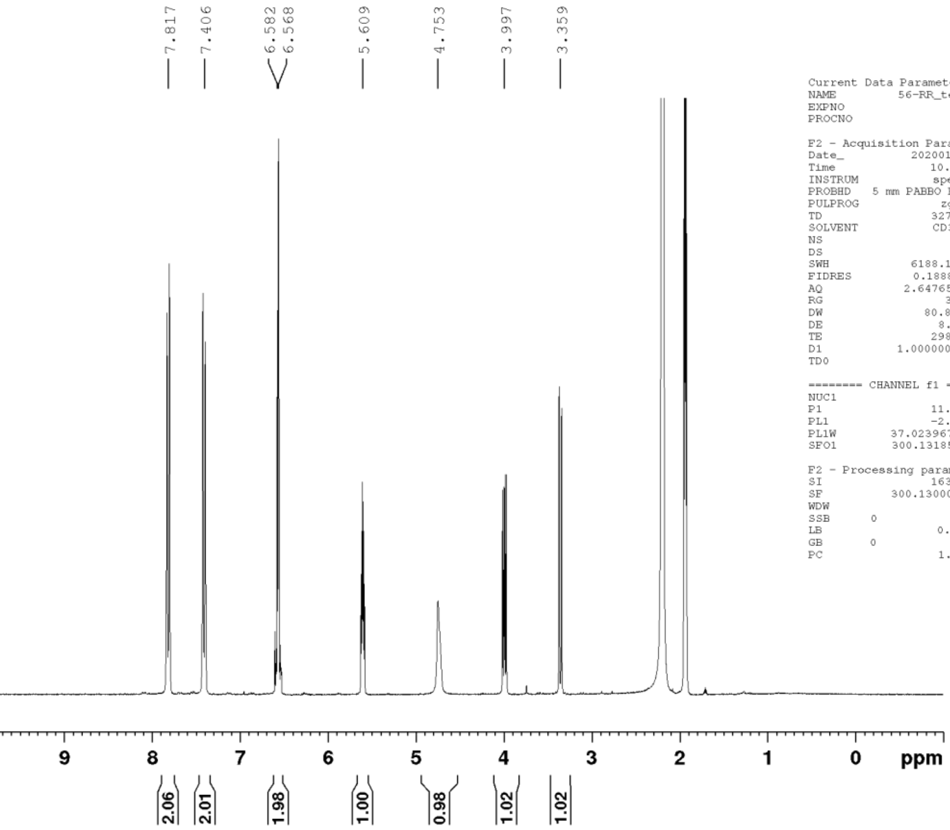


```
Current Data Parameters
NAME          56-RR_test
EXPNO                 81
PROCNO                 1

F2 - Acquisition Parameters
Date_           20200121
Time               10.10
INSTRUM            spect
PROBHD   5 mm PABBO BB-
PULPROG             zg30
TD                 32768
SOLVENT            CD3CN
NS                    64
DS                     2
SWH             6188.119 Hz
FIDRES          0.188846 Hz
AQ             2.6476543 sec
RG                   362
DW                80.800 usec
DE                  8.00 usec
TE                 298.0 K
D1            1.00000000 sec
TD0                    1

======== CHANNEL f1 ========
NUC1                  1H
P1                 11.05 usec
PL1                -2.00 dB
PL1W         37.02396774 W
SFO1         300.1318534 MHz

F2 - Processing parameters
SI                 16384
SF           300.1300075 MHz
WDW                   EM
SSB      0
LB                  0.30 Hz
GB       0
PC                  1.00
```

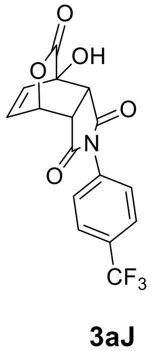

3aJ

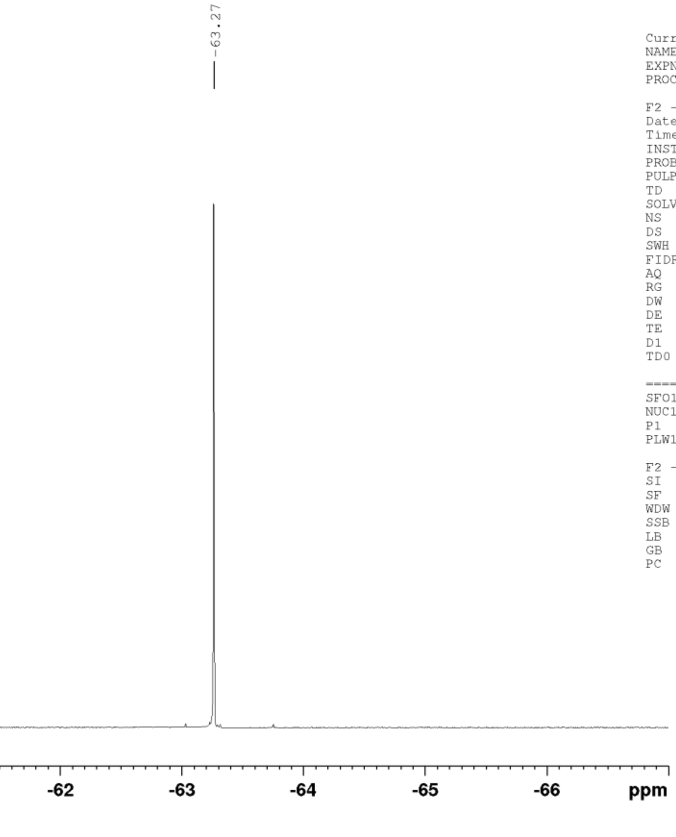


```
Current Data Parameters
NAME            56-4-CF3
EXPNO                 21
PROCNO                 1

F2 - Acquisition Parameters
Date_           20190214
Time                8.25
INSTRUM            spect
PROBHD   5 mm PABBO BB/
PULPROG           zgflqn
TD                131072
SOLVENT            CD3CN
NS                    32
DS                     4
SWH            89285.711 Hz
FIDRES          0.681196 Hz
AQ             0.7340032 sec
RG                205.35
DW                 5.600 usec
DE                  6.50 usec
TE                 298.0 K
D1            1.00000000 sec
TD0                    1

======== CHANNEL f1 ========
SFO1         376.4324910 MHz
NUC1                 19F
P1                 14.65 usec
PLW1         20.00000000 W

F2 - Processing parameters
SI                 65536
SF           376.4701380 MHz
WDW                   EM
SSB      0
LB                  0.30 Hz
GB       0
PC                  1.00
```

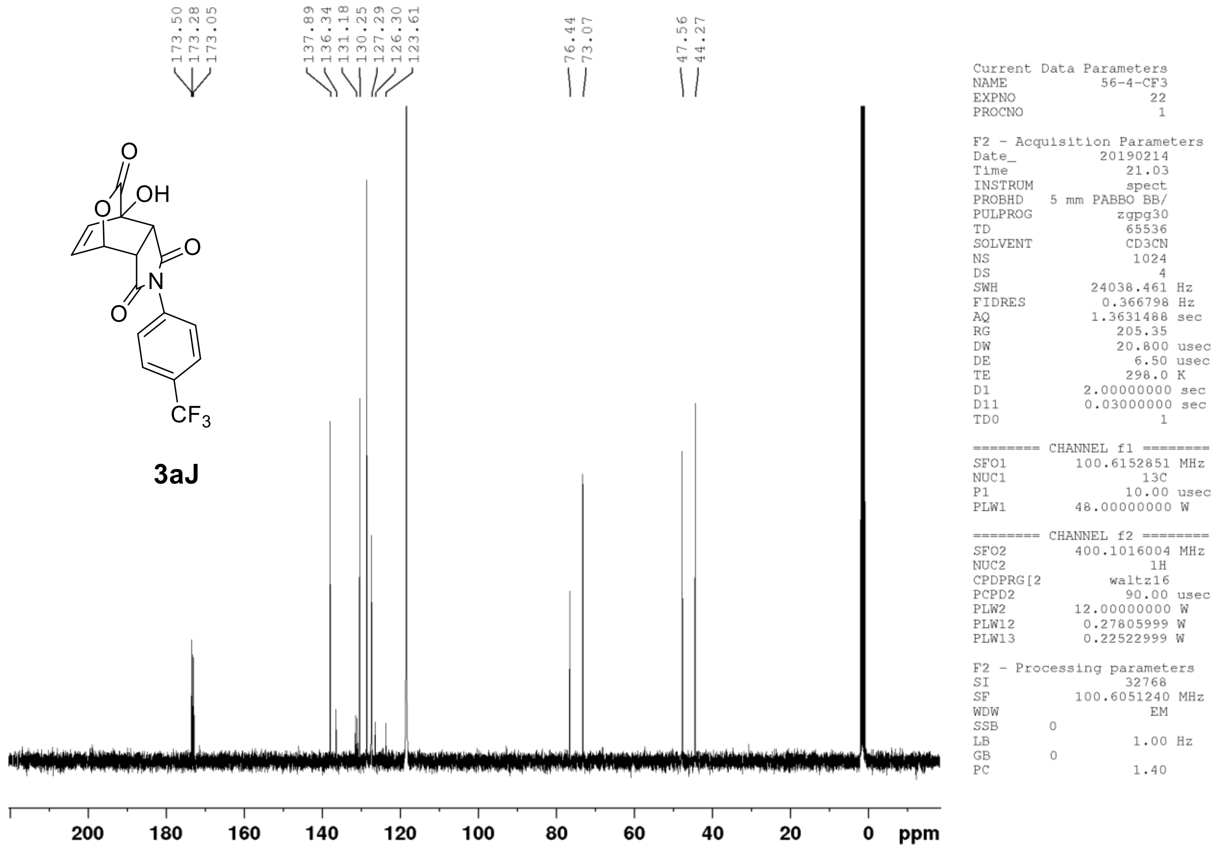
173.50
173.28
173.05
137.89
136.34
131.18
130.25
127.29
126.30
123.61
76.44
73.07
47.56
44.27
O
OH
O
O
N
O
CF3
3aJ
200
180
160
140
120
100
80
60
40
20
0
ppm
Current Data Parameters
NAME 56-4-CF3
EXPNO 22
PROCNO 1
F2 - Acquisition Parameters
Date_ 20190214
Time 21.03
INSTRUM spect
PROBHD 5 mm PABBO BB/
PULPROG zgpg30
TD 65536
SOLVENT CD3CN
NS 1024
DS 4
SWH 24038.461 Hz
FIDRES 0.366798 Hz
AQ 1.3631488 sec
RG 205.35
DW 20.800 usec
DE 6.50 usec
TE 298.0 K
D1 2.00000000 sec
D11 0.03000000 sec
TD0 1
======== CHANNEL f1 ========
SFO1 100.6152851 MHz
NUC1 13C
P1 10.00 usec
PLW1 48.00000000 W
======== CHANNEL f2 ========
SFO2 400.1016004 MHz
NUC2 1H
CPDPRG[2 waltz16
PCPD2 90.00 usec
PLW2 12.00000000 W
PLW12 0.27805999 W
PLW13 0.22522999 W
F2 - Processing parameters
SI 32768
SF 100.6051240 MHz
WDW EM
SSB 0
LB 1.00 Hz
GB 0
PC 1.40

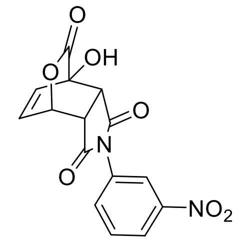

3aK

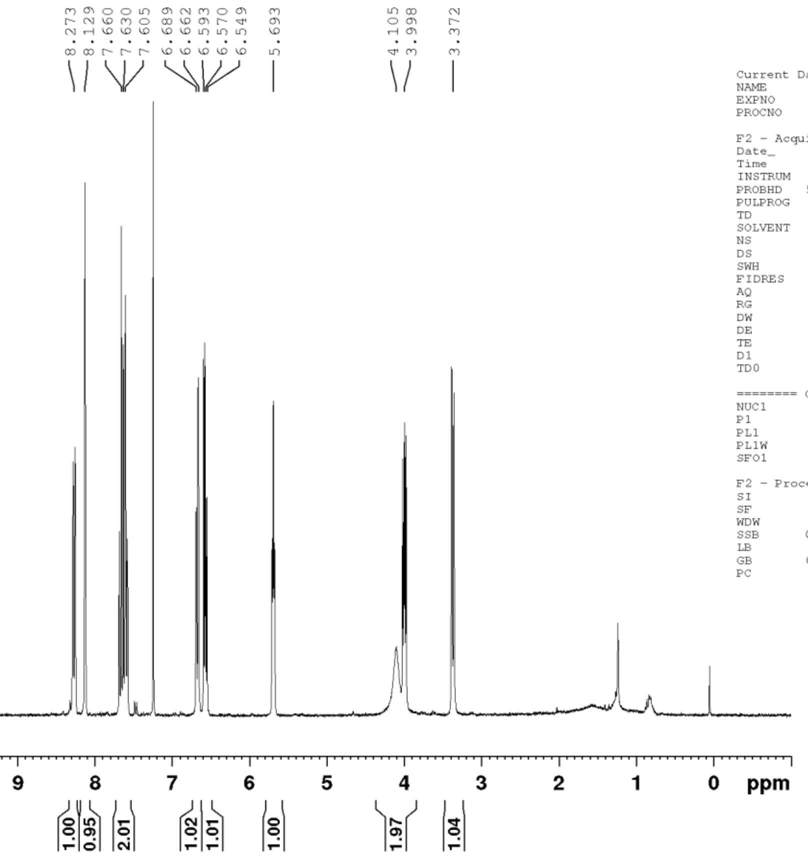


```
Current Data Parameters
NAME             56-RR_3
EXPNO                270
PROCNO                 1

F2 - Acquisition Parameters
Date_           20200124
Time               16.13
INSTRUM            spect
PROBHD   5 mm PABBO BB-
PULPROG             zg30
TD                 32768
SOLVENT            CDCl3
NS                    16
DS                     2
SWH             6188.119 Hz
FIDRES          0.188846 Hz
AQ             2.6476543 sec
RG                   456
DW                80.800 usec
DE                  8.00 usec
TE                 298.0 K
D1            1.00000000 sec
TD0                    1

======== CHANNEL f1 ========
NUC1                  1H
P1                 11.05 usec
PL1                -2.00 dB
PL1W         37.02396774 W
SFO1         300.1318534 MHz

F2 - Processing parameters
SI                 16384
SF           300.1300104 MHz
WDW                   EM
SSB      0
LB                  0.30 Hz
GB       0
PC                  1.00
```

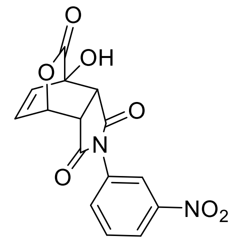

3aK

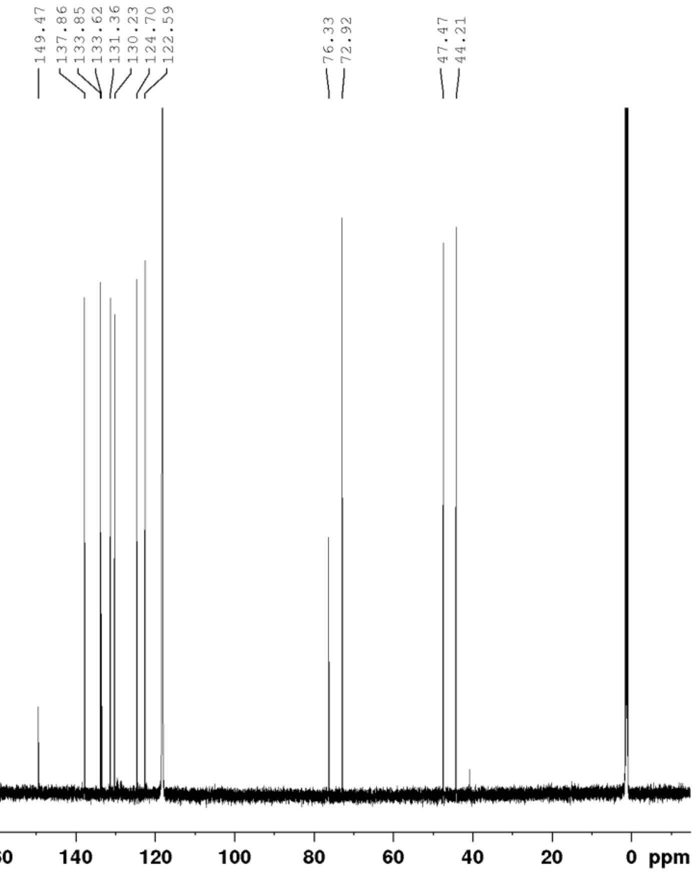


```
Current Data Parameters
NAME        56-3NO2_ref reaction
EXPNO                 41
PROCNO                 1

F2 - Acquisition Parameters
Date_           20190207
Time               12.32
INSTRUM            spect
PROBHD   5 mm CPQCI 1H-
PULPROG          zgpg30
TD                 65356
SOLVENT            CD3CN
NS                  1024
DS                     4
SWH            40760.871 Hz
FIDRES          0.623675 Hz
AQ             0.8017003 sec
RG                182.53
DW                12.267 usec
DE                 18.00 usec
TE                 298.0 K
D1            2.00000000 sec
D11           0.03000000 sec
TD0                    1

======== CHANNEL f1 ========
SFO1         176.1232717 MHz
NUC1                 13C
P1                 12.00 usec
PLW1        105.00000000 W

======== CHANNEL f2 ========
SFO2         700.3628014 MHz
NUC2                  1H
CPDPRG[2         waltz16
PCPD2              65.00 usec
PLW2          9.89999962 W
PLW12         0.15564001 W
PLW13         0.07837200 W

F2 - Processing parameters
SI                131072
SF           176.1055005 MHz
WDW                   EM
SSB      0
LB                  1.00 Hz
GB       0
PC                  1.40
```

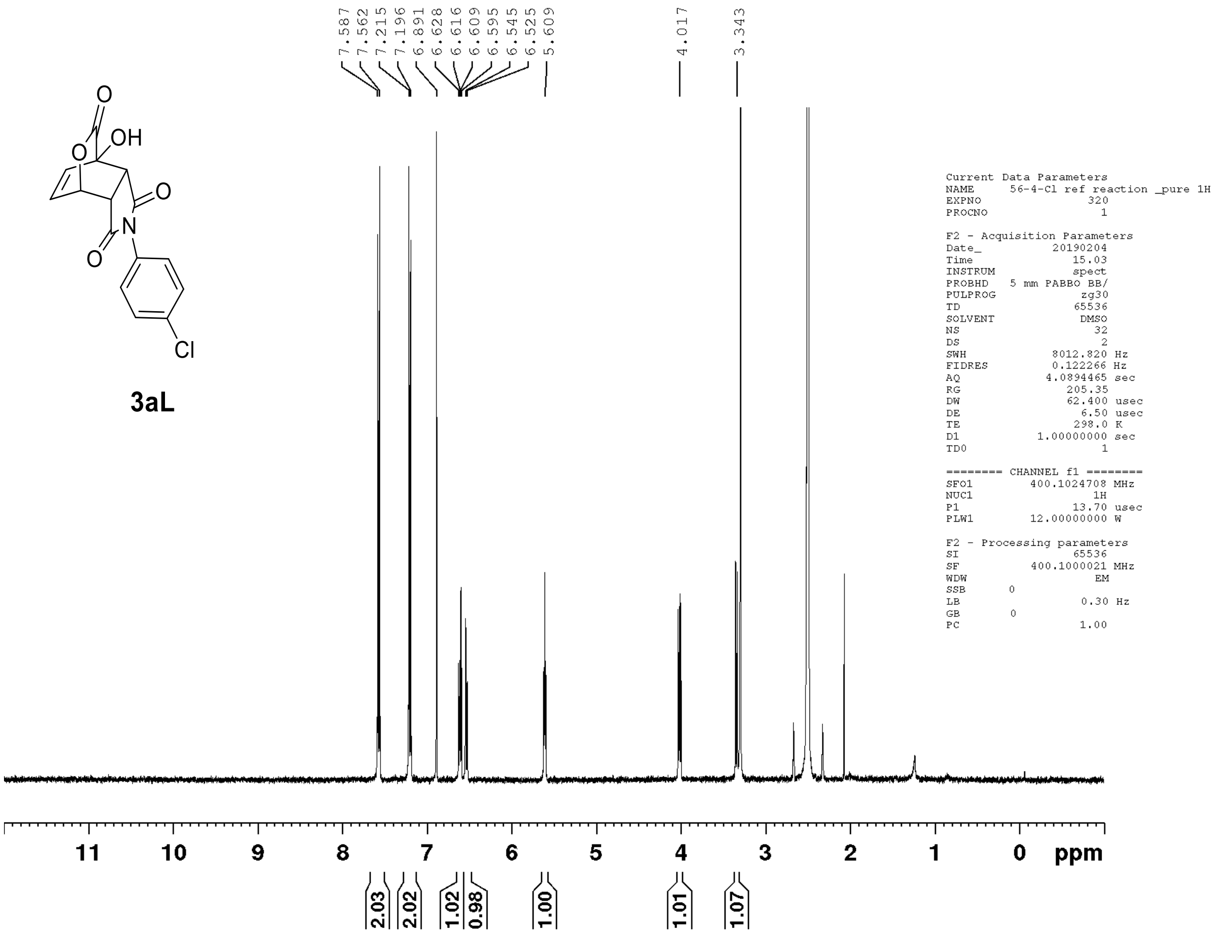


```
Current Data Parameters
NAME      56-4-Cl ref reaction _pure 1H
EXPNO                 320
PROCNO                  1

F2 - Acquisition Parameters
Date_            20190204
Time                15.03
INSTRUM             spect
PROBHD   5 mm PABBO BB/
PULPROG              zg30
TD                  65536
SOLVENT              DMSO
NS                     32
DS                      2
SWH             8012.820 Hz
FIDRES          0.122266 Hz
AQ             4.0894465 sec
RG                 205.35
DW                 62.400 usec
DE                   6.50 usec
TE                  298.0 K
D1            1.00000000 sec
TD0                     1

======== CHANNEL f1 ========
SFO1         400.1024708 MHz
NUC1                  1H
P1                  13.70 usec
PLW1         12.00000000 W

F2 - Processing parameters
SI                  65536
SF            400.1000021 MHz
WDW                    EM
SSB      0
LB                   0.30 Hz
GB       0
PC                   1.00
```

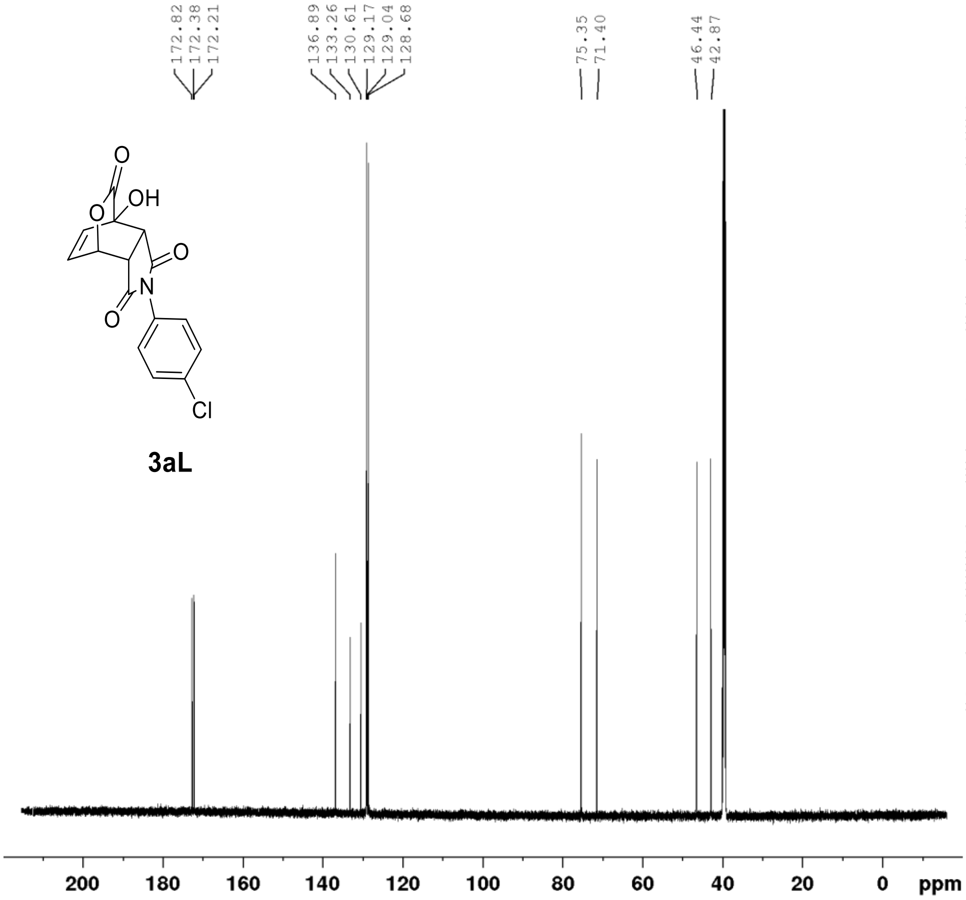


```
Current Data Parameters
NAME       56-4-Cl_ref reaction
EXPNO                   51
PROCNO                   1

F2 - Acquisition Parameters
Date_             20190207
Time                 13.32
INSTRUM              spect
PROBHD   5 mm CPQCI 1H-
PULPROG             zgpg30
TD                   65356
SOLVENT               DMSO
NS                    1024
DS                       4
SWH             40760.871 Hz
FIDRES           0.623675 Hz
AQ              0.8017003 sec
RG                 182.53
DW                 12.267 usec
DE                  18.00 usec
TE                  298.0 K
D1             2.00000000 sec
D11            0.03000000 sec
TD0                      1

======== CHANNEL f1 ========
SFO1          176.1232717 MHz
NUC1                  13C
P1                  12.00 usec
PLW1         105.00000000 W

======== CHANNEL f2 ========
SFO2          700.3628014 MHz
NUC2                   1H
CPDPRG[2           waltz16
PCPD2               65.00 usec
PLW2           9.89999962 W
PLW12          0.15564001 W
PLW13          0.07837200 W

F2 - Processing parameters
SI                  131072
SF            176.1057444 MHz
WDW                    EM
SSB      0
LB                   1.00 Hz
GB       0
PC                   1.40
```

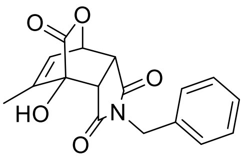

3bB

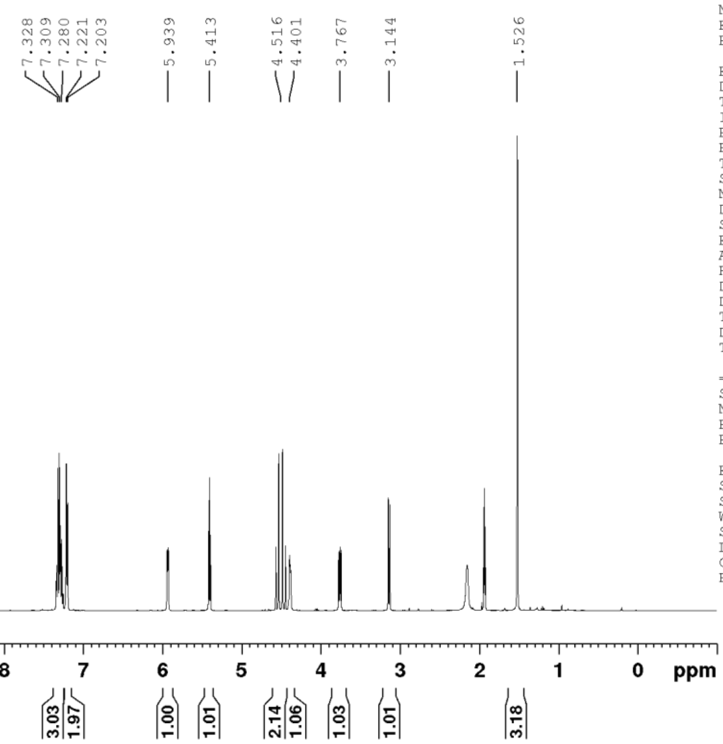


```
Current Data Parameters
NAME              56-R1
EXPNO               330
PROCNO                1

F2 - Acquisition Parameters
Date_          20190410
Time              14.50
INSTRUM           spect
PROBHD   5 mm PABBO BB/
PULPROG            zg30
TD                65536
SOLVENT           CD3CN
NS                   16
DS                    2
SWH           8012.820 Hz
FIDRES        0.122266 Hz
AQ           4.0894465 sec
RG               124.07
DW               62.400 usec
DE                 6.50 usec
TE                298.0 K
D1           1.00000000 sec
TD0                   1

======== CHANNEL f1 ========
SFO1        400.1024708 MHz
NUC1                 1H
P1                13.70 usec
PLW1        12.00000000 W

F2 - Processing parameters
SI                65536
SF          400.1000112 MHz
WDW                  EM
SSB      0
LB                 0.30 Hz
GB       0
PC                 1.00
```

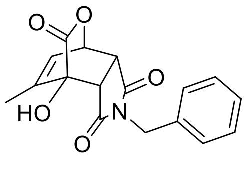

3bB

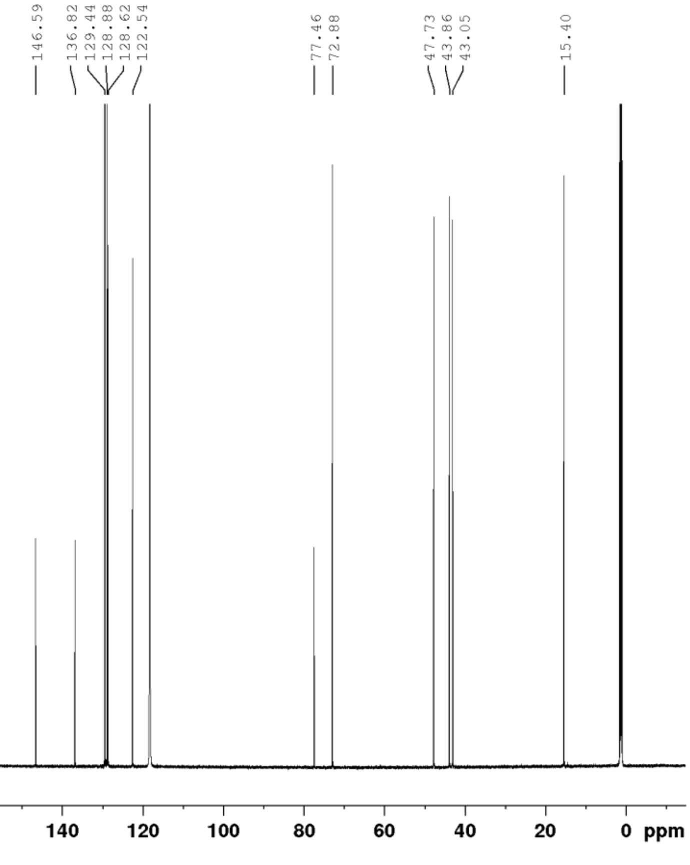


```
Current Data Parameters
NAME           56-Me-Hp
EXPNO                81
PROCNO                1

F2 - Acquisition Parameters
Date_          20190410
Time              17.13
INSTRUM           spect
PROBHD   5 mm CPQCI 1H-
PULPROG          zgpg30
TD                65356
SOLVENT           CD3CN
NS                  512
DS                    4
SWH          40760.871 Hz
FIDRES        0.623675 Hz
AQ           0.8017003 sec
RG               182.53
DW               12.267 usec
DE                18.00 usec
TE                298.0 K
D1           2.00000000 sec
D11          0.03000000 sec
TD0                   1

======== CHANNEL f1 ========
SFO1        176.1232717 MHz
NUC1                13C
P1                12.00 usec
PLW1       105.00000000 W

======== CHANNEL f2 ========
SFO2        700.3628014 MHz
NUC2                 1H
CPDPRG[2        waltz16
PCPD2             65.00 usec
PLW2         9.89999962 W
PLW12        0.15564001 W
PLW13        0.07837200 W

F2 - Processing parameters
SI               131072
SF          176.1055029 MHz
WDW                  EM
SSB      0
LB                 1.00 Hz
GB       0
PC                 1.40
```

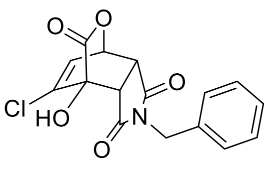

3cB

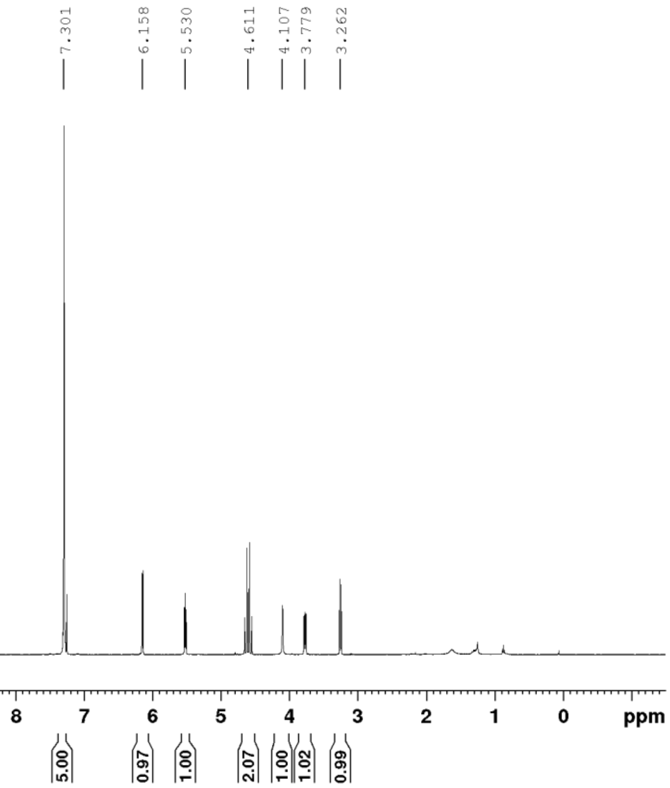


```
Current Data Parameters
NAME          56-HP4C1
EXPNO              100
PROCNO               1

F2 - Acquisition Parameters
Date_          20190409
Time              11.06
INSTRUM           spect
PROBHD   5 mm PABBO BB/
PULPROG            zg30
TD                65536
SOLVENT           CDCl3
NS                   16
DS                    2
SWH            8012.820 Hz
FIDRES         0.122266 Hz
AQ            4.0894465 sec
RG               205.35
DW               62.400 usec
DE                 6.50 usec
TE                298.0 K
D1           1.00000000 sec
TD0                   1

======== CHANNEL f1 ========
SFO1        400.1024708 MHz
NUC1                 1H
P1                13.70 usec
PLW1        12.00000000 W

F2 - Processing parameters
SI                65536
SF          400.1000101 MHz
WDW                  EM
SSB      0
LB                 0.30 Hz
GB       0
PC                 1.00
```

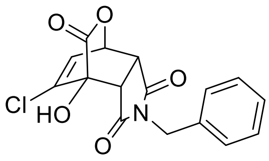

3cB

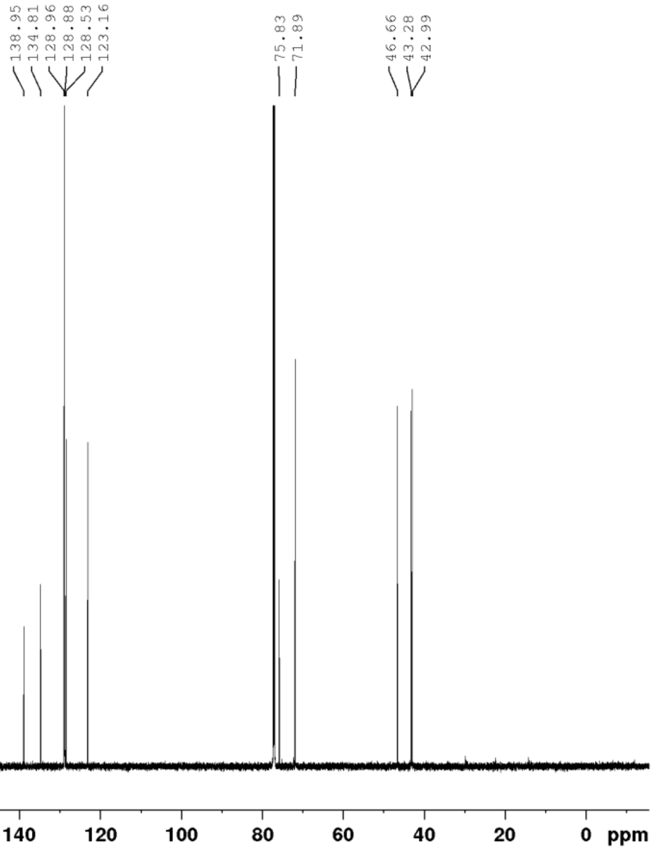


```
Current Data Parameters
NAME       56-HP4-Hp-Cl
EXPNO               21
PROCNO               1

F2 - Acquisition Parameters
Date_          20190409
Time              12.44
INSTRUM           spect
PROBHD   5 mm CPQCI 1H-
PULPROG         zgpg30
TD                65356
SOLVENT           CDCl3
NS                 1024
DS                    4
SWH           40760.871 Hz
FIDRES         0.623675 Hz
AQ            0.8017003 sec
RG               182.53
DW               12.267 usec
DE                18.00 usec
TE                298.0 K
D1           2.00000000 sec
D11          0.03000000 sec
TD0                   1

======== CHANNEL f1 ========
SFO1        176.1232717 MHz
NUC1                13C
P1                12.00 usec
PLW1       105.00000000 W

======== CHANNEL f2 ========
SFO2        700.3628014 MHz
NUC2                 1H
CPDPRG[2        waltz16
PCPD2             65.00 usec
PLW2         9.89999962 W
PLW12        0.15564001 W
PLW13        0.07837200 W

F2 - Processing parameters
SI               131072
SF          176.1056433 MHz
WDW                  EM
SSB      0
LB                 1.00 Hz
GB       0
PC                 1.40
```

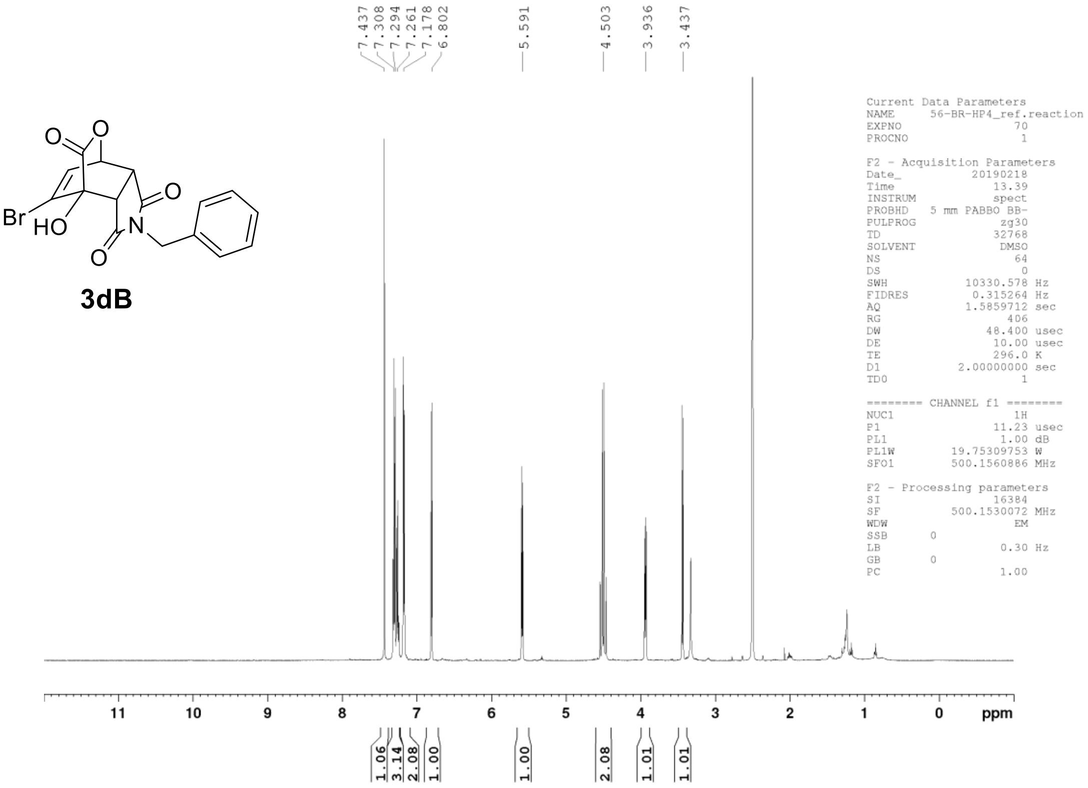
7.437
7.308
7.294
7.261
7.178
6.802
5.591
4.503
3.936
3.437
Br
HO
N
O
3dB
Current Data Parameters
NAME 56-BR-HP4_ref.reaction
EXPNO 70
PROCNO 1
F2 - Acquisition Parameters
Date_ 20190218
Time 13.39
INSTRUM spect
PROBHD 5 mm PABBO BB-
PULPROG zg30
TD 32768
SOLVENT DMSO
NS 64
DS 0
SWH 10330.578 Hz
FIDRES 0.315264 Hz
AQ 1.5859712 sec
RG 406
DW 48.400 usec
DE 10.00 usec
TE 296.0 K
D1 2.00000000 sec
TD0 1
======== CHANNEL f1 ========
NUC1 1H
P1 11.23 usec
PL1 1.00 dB
PL1W 19.75309753 W
SFO1 500.1560886 MHz
F2 - Processing parameters
SI 16384
SF 500.1530072 MHz
WDW EM
SSB 0
LB 0.30 Hz
GB 0
PC 1.00
11 10 9 8 7 6 5 4 3 2 1 0 ppm
1.06
3.14
2.08
1.00
1.00
2.08
1.01
1.01

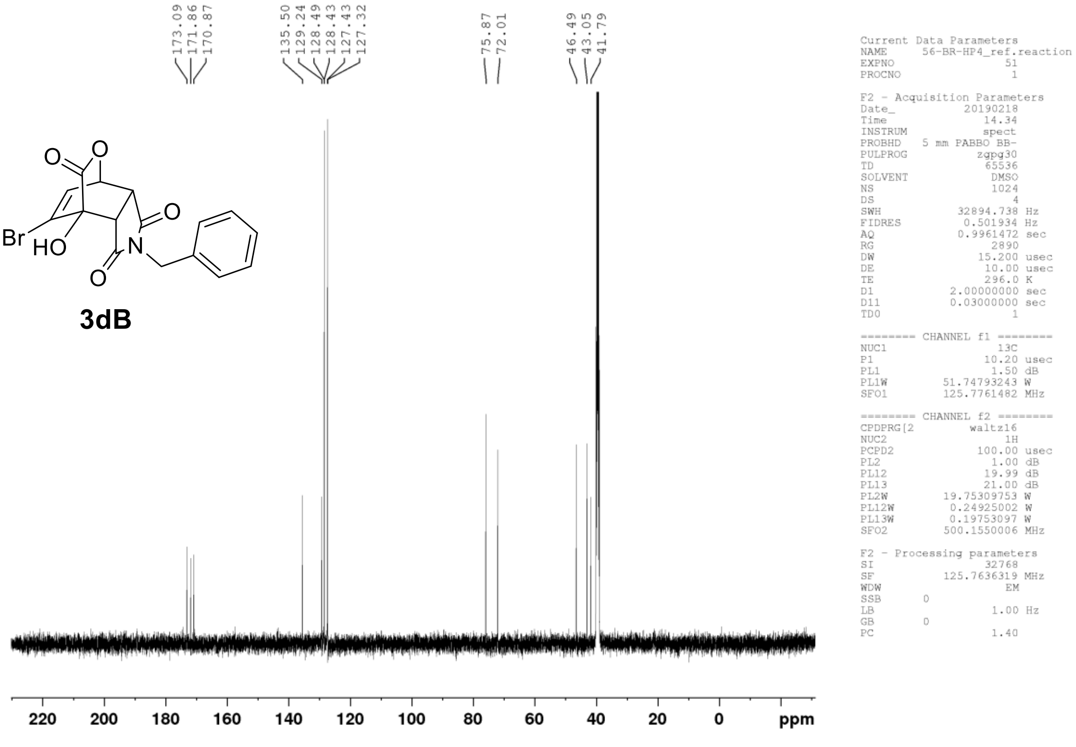
173.09
171.86
170.87
135.50
129.24
128.49
128.43
127.43
127.32
75.87
72.01
46.49
43.05
41.79
Br
HO
N
O
3dB
Current Data Parameters
NAME 56-BR-HP4_ref.reaction
EXPNO 51
PROCNO 1
F2 - Acquisition Parameters
Date_ 20190218
Time 14.34
INSTRUM spect
PROBHD 5 mm PABBO BB-
PULPROG zgpg30
TD 65536
SOLVENT DMSO
NS 1024
DS 4
SWH 32894.738 Hz
FIDRES 0.501934 Hz
AQ 0.9961472 sec
RG 2890
DW 15.200 usec
DE 10.00 usec
TE 296.0 K
D1 2.00000000 sec
D11 0.03000000 sec
TD0 1
======== CHANNEL f1 ========
NUC1 13C
P1 10.20 usec
PL1 1.50 dB
PL1W 51.74793243 W
SFO1 125.7761482 MHz
======== CHANNEL f2 ========
CPDPRG[2 waltz16
NUC2 1H
PCPD2 100.00 usec
PL2 1.00 dB
PL12 19.99 dB
PL13 21.00 dB
PL2W 19.75309753 W
PL12W 0.24925002 W
PL13W 0.19753097 W
SFO2 500.1550006 MHz
F2 - Processing parameters
SI 32768
SF 125.7636319 MHz
WDW EM
SSB 0
LB 1.00 Hz
GB 0
PC 1.40
220 200 180 160 140 120 100 80 60 40 20 0 ppm

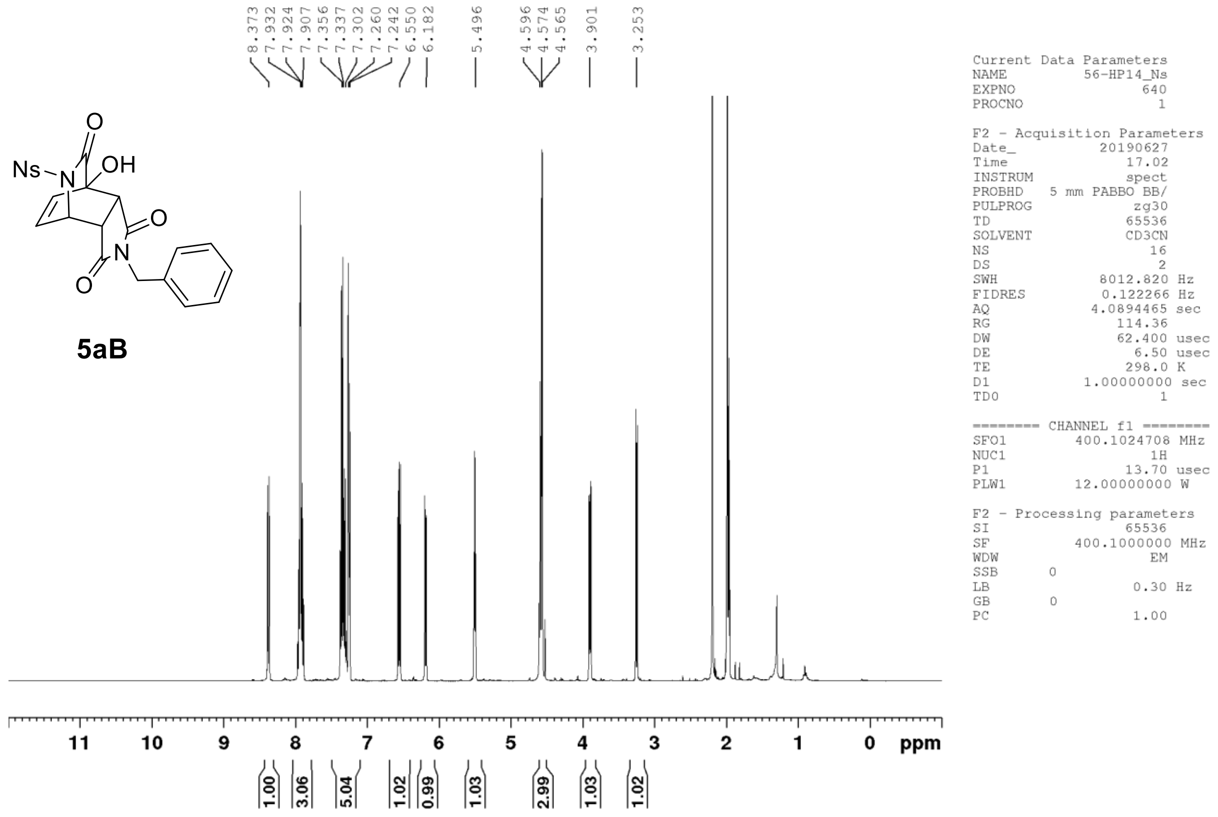

8.373
7.932
7.924
7.907
7.356
7.337
7.302
7.260
7.242
6.550
6.182
5.496
4.596
4.574
4.565
3.901
3.253
Ns
N
O
OH
O
N
O
5aB
11 10 9 8 7 6 5 4 3 2 1 0 ppm
1.00
3.06
5.04
1.02
0.99
1.03
2.99
1.03
1.02
Current Data Parameters
NAME 56-HP14_Ns
EXPNO 640
PROCNO 1
F2 - Acquisition Parameters
Date_ 20190627
Time 17.02
INSTRUM spect
PROBHD 5 mm PABBO BB/
PULPROG zg30
TD 65536
SOLVENT CD3CN
NS 16
DS 2
SWH 8012.820 Hz
FIDRES 0.122266 Hz
AQ 4.0894465 sec
RG 114.36
DW 62.400 usec
DE 6.50 usec
TE 298.0 K
D1 1.00000000 sec
TD0 1
======== CHANNEL f1 ========
SFO1 400.1024708 MHz
NUC1 1H
P1 13.70 usec
PLW1 12.00000000 W
F2 - Processing parameters
SI 65536
SF 400.1000000 MHz
WDW EM
SSB 0
LB 0.30 Hz
GB 0
PC 1.00

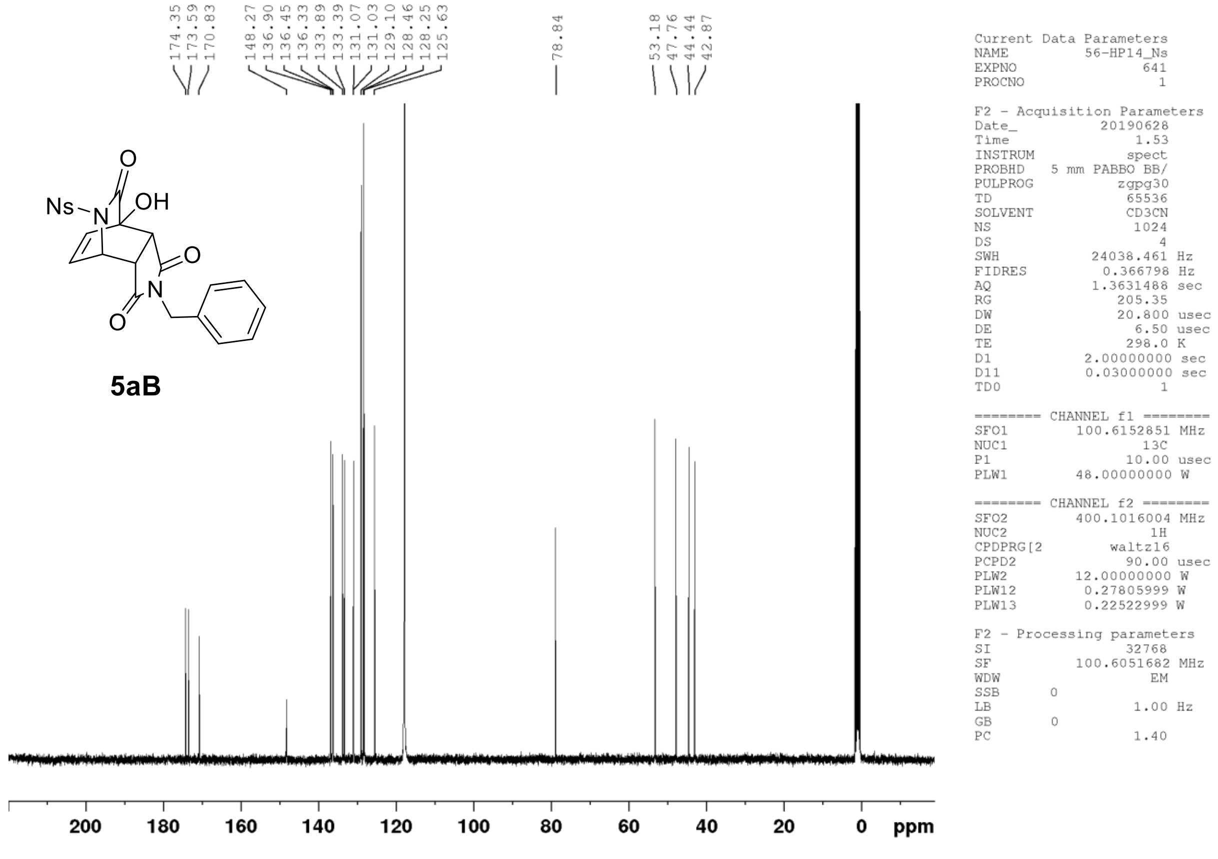

174.35
173.59
170.83
148.27
136.90
136.45
136.33
133.89
133.39
131.07
131.03
129.10
128.46
128.25
125.63
78.84
53.18
47.76
44.44
42.87
Ns
N
O
OH
O
N
O
5aB
200 180 160 140 120 100 80 60 40 20 0 ppm
Current Data Parameters
NAME 56-HP14_Ns
EXPNO 641
PROCNO 1
F2 - Acquisition Parameters
Date_ 20190628
Time 1.53
INSTRUM spect
PROBHD 5 mm PABBO BB/
PULPROG zgpg30
TD 65536
SOLVENT CD3CN
NS 1024
DS 4
SWH 24038.461 Hz
FIDRES 0.366798 Hz
AQ 1.3631488 sec
RG 205.35
DW 20.800 usec
DE 6.50 usec
TE 298.0 K
D1 2.00000000 sec
D11 0.03000000 sec
TD0 1
======== CHANNEL f1 ========
SFO1 100.6152851 MHz
NUC1 13C
P1 10.00 usec
PLW1 48.00000000 W
======== CHANNEL f2 ========
SFO2 400.1016004 MHz
NUC2 1H
CPDPRG[2 waltz16
PCPD2 90.00 usec
PLW2 12.00000000 W
PLW12 0.27805999 W
PLW13 0.22522999 W
F2 - Processing parameters
SI 32768
SF 100.6051682 MHz
WDW EM
SSB 0
LB 1.00 Hz
GB 0
PC 1.40

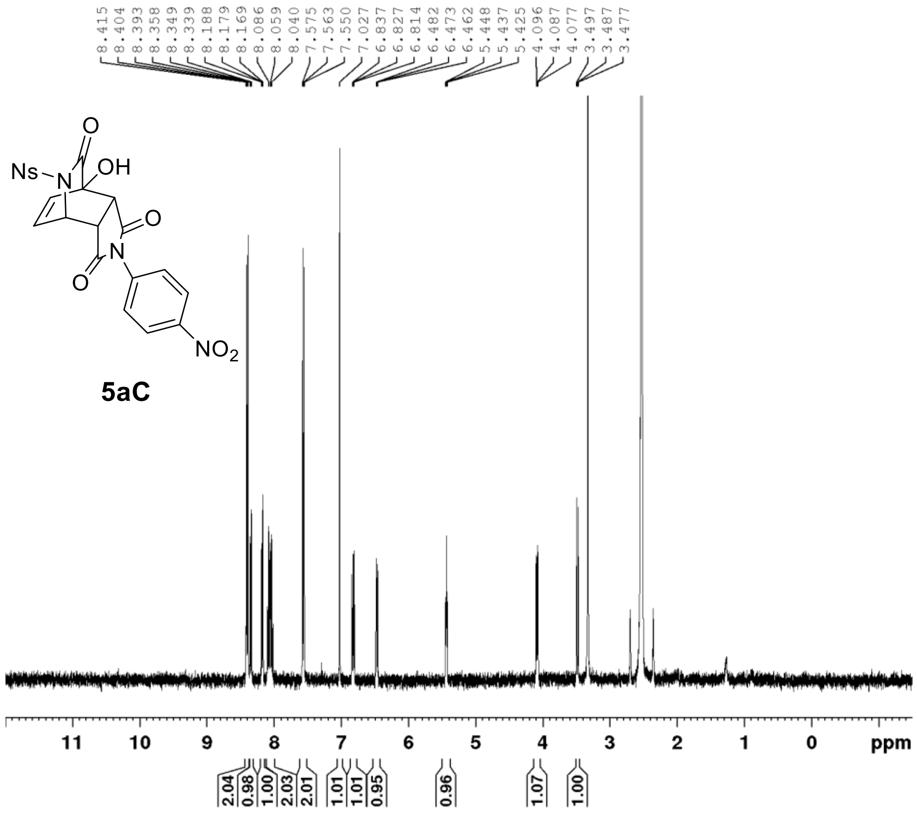

Current Data Parameters
NAME 56-HP14-NO2
EXPNO 400
PROCNO 1

F2 - Acquisition Parameters
Date_ 20200423
Time 15.15
INSTRUM spect
PROBHD 5 mm PABBO BB/
PULPROG zg30
TD 65536
SOLVENT DMSO
NS 16
DS 2
SWH 8012.820 Hz
FIDRES 0.122266 Hz
AQ 4.0894465 sec
RG 205.35
DW 62.400 usec
DE 6.50 usec
TE 298.0 K
D1 1.00000000 sec
TD0 1

======== CHANNEL f1 ========
SFO1 400.1024708 MHz
NUC1 1H
P1 13.70 usec
PLW1 12.00000000 W

F2 - Processing parameters
SI 65536
SF 400.0999906 MHz
WDW EM
SSB 0
LB 0.30 Hz
GB 0
PC 1.00

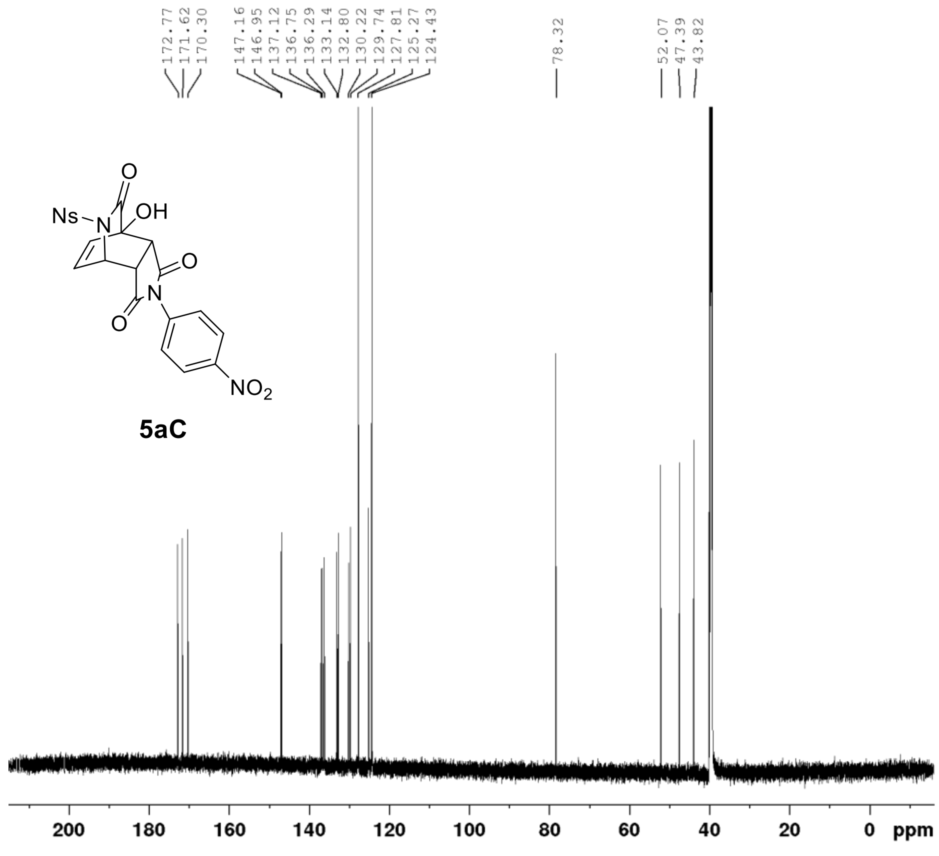

Current Data Parameters
NAME 56-HP14-4NO2
EXPNO 50
PROCNO 1

F2 - Acquisition Parameters
Date_ 20200423
Time 17.47
INSTRUM spect
PROBHD 5 mm CPQCI 1H-
PULPROG zgpg30
TD 65356
SOLVENT DMSO
NS 2048
DS 4
SWH 40760.871 Hz
FIDRES 0.623675 Hz
AQ 0.8017003 sec
RG 182.53
DW 12.267 usec
DE 18.00 usec
TE 298.0 K
D1 2.00000000 sec
D11 0.03000000 sec
TD0 1

======== CHANNEL f1 ========
SFO1 176.1232717 MHz
NUC1 13C
P1 12.00 usec
PLW1 105.00000000 W

======== CHANNEL f2 ========
SFO2 700.3628014 MHz
NUC2 1H
CPDPRG[2 waltz16
PCPD2 65.00 usec
PLW2 9.89999962 W
PLW12 0.15564001 W
PLW13 0.07837200 W

F2 - Processing parameters
SI 131072
SF 176.1057470 MHz
WDW EM
SSB 0
LB 1.00 Hz
GB 0
PC 1.40

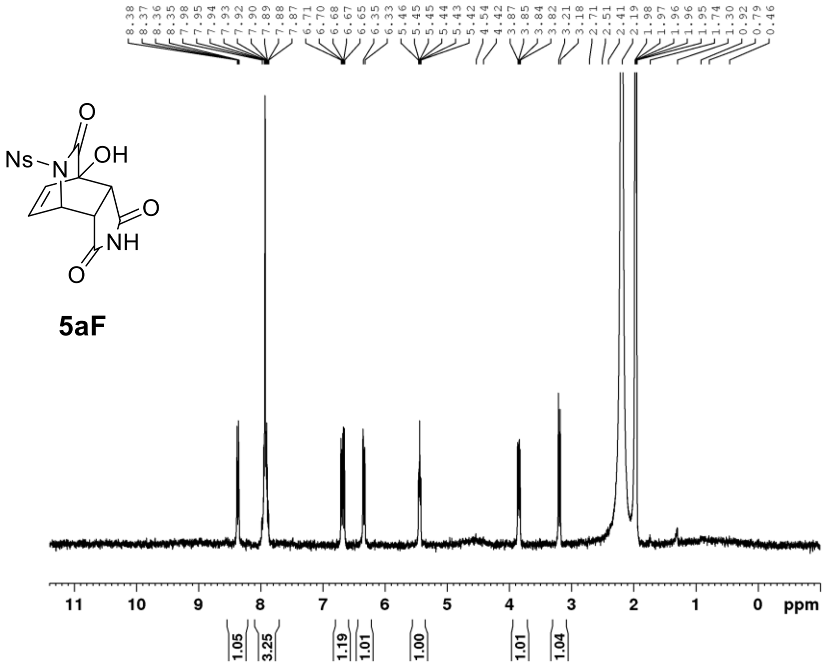


```
NAME          Jul11-2020
EXPNO                160
PROCNO                 1
Date_           20200711
Time               14.32
INSTRUM            spect
PROBHD   5 mm PABBO BB-
PULPROG             zg30
TD                 32768
SOLVENT            CD3CN
NS                    16
DS                     2
SWH          6188.119 Hz
FIDRES       0.188846 Hz
AQ         2.6477044 sec
RG                   812
DW            80.800 usec
DE              8.00 usec
TE               298.0 K
D1        1.00000000 sec
TD0                    1

======== CHANNEL f1 ========
NUC1                  1H
P1             11.05 usec
PL1             -2.00 dB
PL1W      37.02396774 W
SFO1      300.1318534 MHz
SI                 16384
SF        300.1300000 MHz
WDW                   EM
SSB                    0
LB               0.30 Hz
GB                     0
PC                  1.00
```

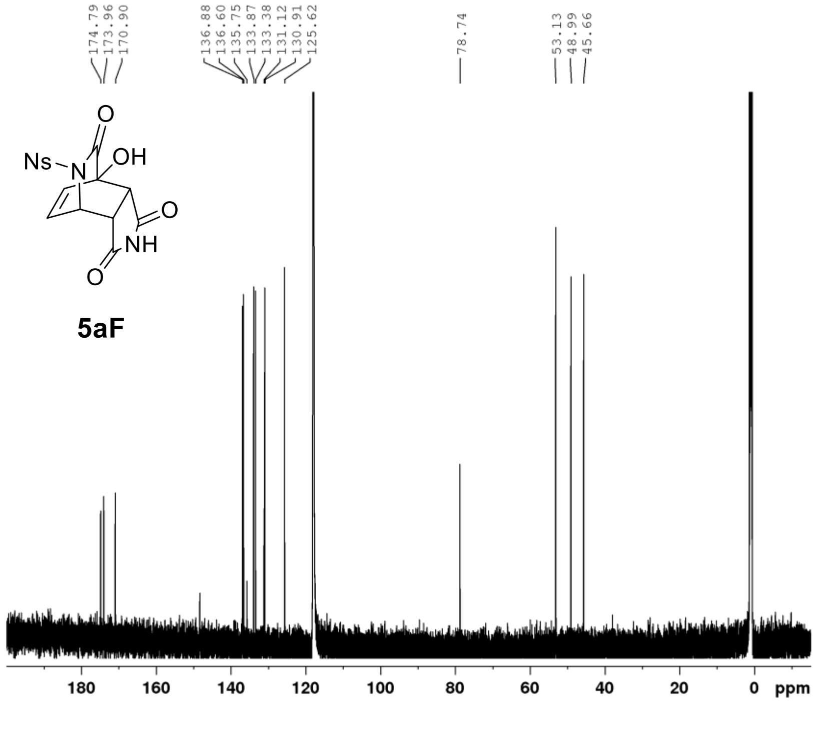


```
Current Data Parameters
NAME          Jul13-2020
EXPNO                 80
PROCNO                 1

F2 - Acquisition Parameters
Date_           20200713
Time               16.49
INSTRUM            spect
PROBHD   5 mm CPQCI 1H-
PULPROG          zgpg30
TD                 65356
SOLVENT            CD3CN
NS                  1024
DS                     4
SWH         40760.871 Hz
FIDRES       0.623675 Hz
AQ         0.8017003 sec
RG                182.53
DW            12.267 usec
DE             18.00 usec
TE               298.0 K
D1        2.00000000 sec
D11       0.03000000 sec
TD0                    1

======== CHANNEL f1 ========
SFO1      176.1232717 MHz
NUC1                 13C
P1             12.00 usec
PLW1     105.00000000 W

======== CHANNEL f2 ========
SFO2      700.3628014 MHz
NUC2                  1H
CPDPRG[2         waltz16
PCPD2          65.00 usec
PLW2        9.89999962 W
PLW12       0.15564001 W
PLW13       0.07837200 W

F2 - Processing parameters
SI                131072
SF        176.1055627 MHz
WDW                   EM
SSB      0
LB               1.00 Hz
GB       0
PC                  1.40
```

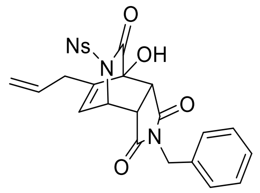
Ns
N
O
OH
O
N
O

5bB

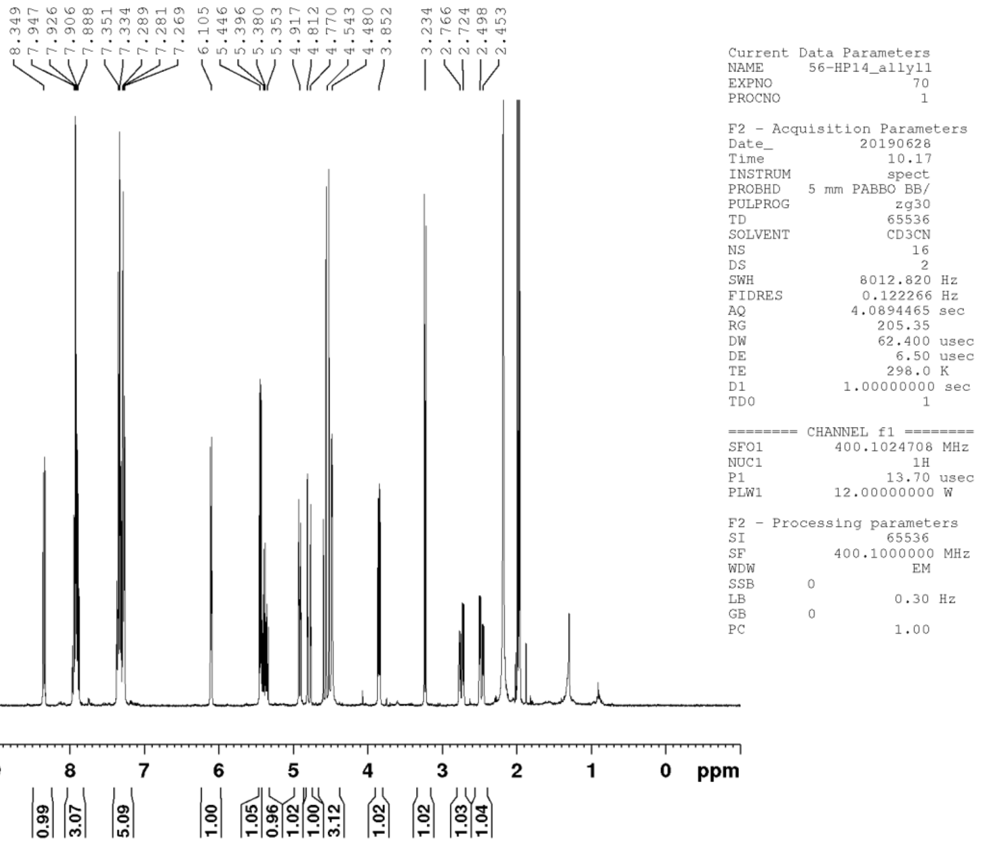
8.349
7.947
7.926
7.906
7.888
7.351
7.334
7.289
7.281
7.269
6.105
5.446
5.396
5.380
5.353
4.917
4.812
4.770
4.543
4.480
3.852
3.234
2.766
2.724
2.498
2.453
11 10 9 8 7 6 5 4 3 2 1 0 ppm
0.99
3.07
5.09
1.00
1.05
0.96
1.02
1.00
3.12
1.02
1.02
1.03
1.04
Current Data Parameters
NAME 56-HP14_allyl1
EXPNO 70
PROCNO 1
F2 - Acquisition Parameters
Date_ 20190628
Time 10.17
INSTRUM spect
PROBHD 5 mm PABBO BB/
PULPROG zg30
TD 65536
SOLVENT CD3CN
NS 16
DS 2
SWH 8012.820 Hz
FIDRES 0.122266 Hz
AQ 4.0894465 sec
RG 205.35
DW 62.400 usec
DE 6.50 usec
TE 298.0 K
D1 1.00000000 sec
TD0 1
======== CHANNEL f1 ========
SFO1 400.1024708 MHz
NUC1 1H
P1 13.70 usec
PLW1 12.00000000 W
F2 - Processing parameters
SI 65536
SF 400.1000000 MHz
WDW EM
SSB 0
LB 0.30 Hz
GB 0
PC 1.00

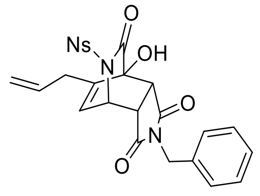
Ns
N
O
OH
O
N
O

5bB

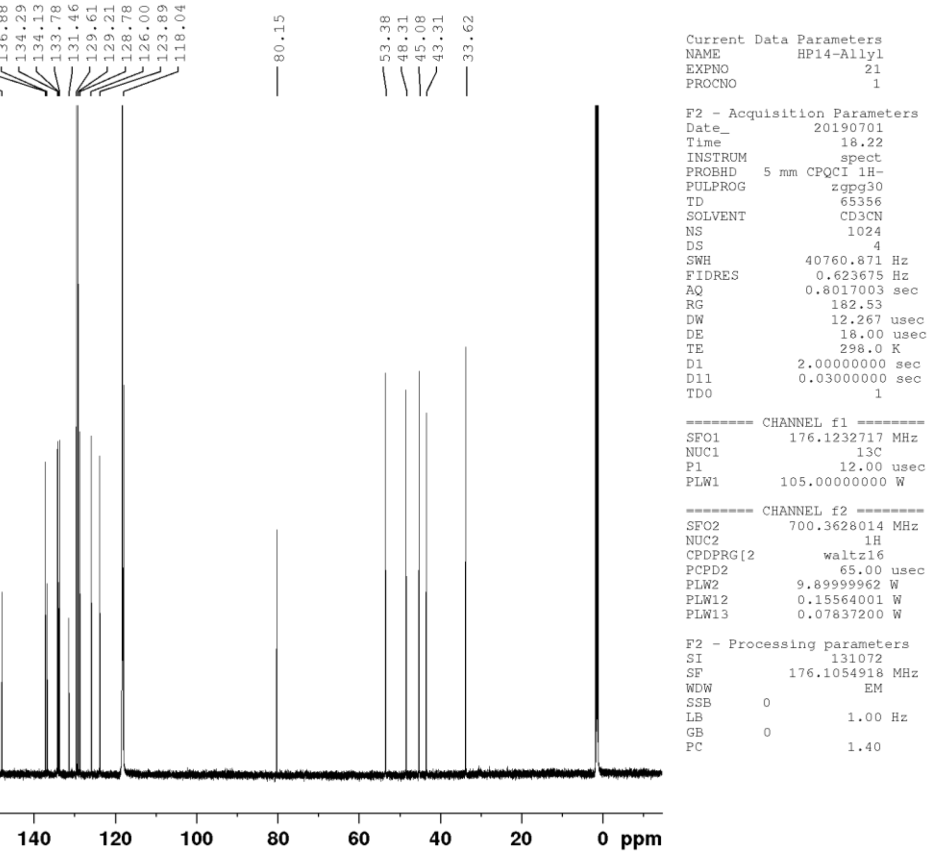
174.86
174.08
171.45
148.68
147.93
137.29
136.88
134.29
134.13
133.78
131.46
129.61
129.21
128.78
126.00
123.89
118.04
80.15
53.38
48.31
45.08
43.31
33.62
200 180 160 140 120 100 80 60 40 20 0 ppm
Current Data Parameters
NAME HP14-Allyl
EXPNO 21
PROCNO 1
F2 - Acquisition Parameters
Date_ 20190701
Time 18.22
INSTRUM spect
PROBHD 5 mm CPQCI 1H-
PULPROG zgpg30
TD 65356
SOLVENT CD3CN
NS 1024
DS 4
SWH 40760.871 Hz
FIDRES 0.623675 Hz
AQ 0.8017003 sec
RG 182.53
DW 12.267 usec
DE 18.00 usec
TE 298.0 K
D1 2.00000000 sec
D11 0.03000000 sec
TD0 1
======== CHANNEL f1 ========
SFO1 176.1232717 MHz
NUC1 13C
P1 12.00 usec
PLW1 105.00000000 W
======== CHANNEL f2 ========
SFO2 700.3628014 MHz
NUC2 1H
CPDPRG[2 waltz16
PCPD2 65.00 usec
PLW2 9.89999962 W
PLW12 0.15564001 W
PLW13 0.07837200 W
F2 - Processing parameters
SI 131072
SF 176.1054918 MHz
WDW EM
SSB 0
LB 1.00 Hz
GB 0
PC 1.40

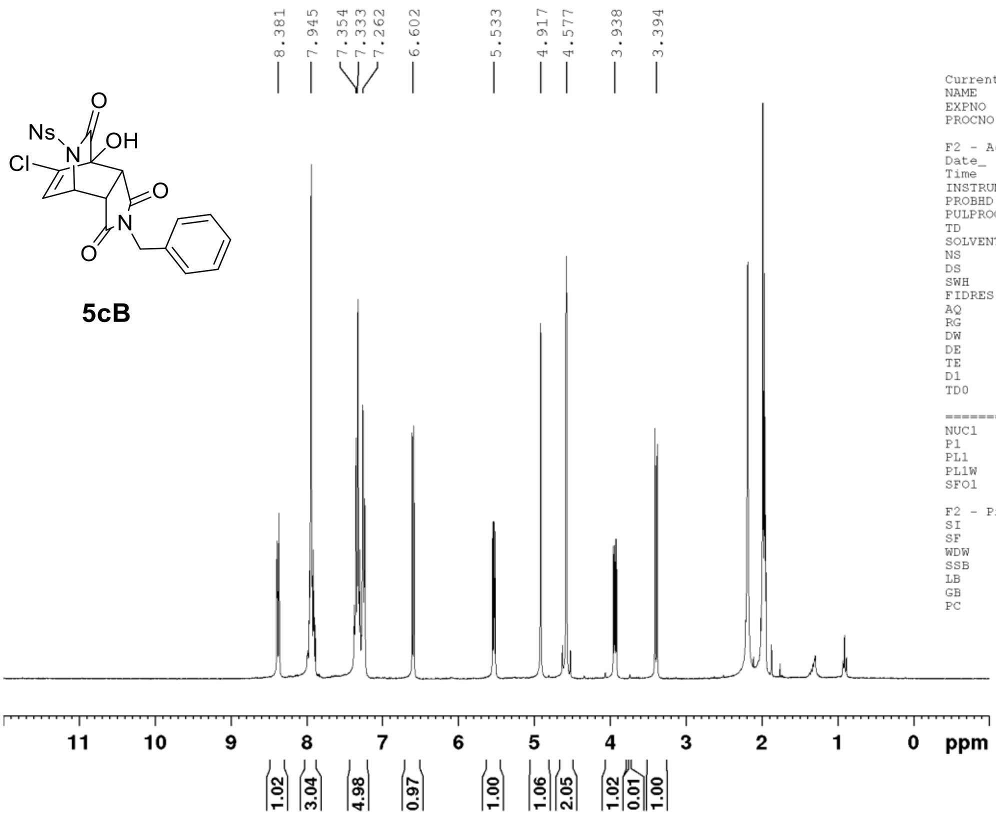

Current Data Parameters
NAME 56-HP14_C1
EXPNO 150
PROCNO 1

F2 - Acquisition Parameters
Date_ 20190626
Time 10.32
INSTRUM spect
PROBHD 5 mm PABBO BB-
PULPROG zg30
TD 32768
SOLVENT CD3CN
NS 16
DS 2
SWH 6188.119 Hz
FIDRES 0.188846 Hz
AQ 2.6476543 sec
RG 322
DW 80.800 usec
DE 8.00 usec
TE 298.0 K
D1 1.00000000 sec
TD0 1

======== CHANNEL f1 ========
NUC1 1H
P1 11.05 usec
PL1 -2.00 dB
PL1W 37.02396774 W
SFO1 300.1318534 MHz

F2 - Processing parameters
SI 16384
SF 300.1300000 MHz
WDW EM
SSB 0
LB 0.30 Hz
GB 0
PC 1.00

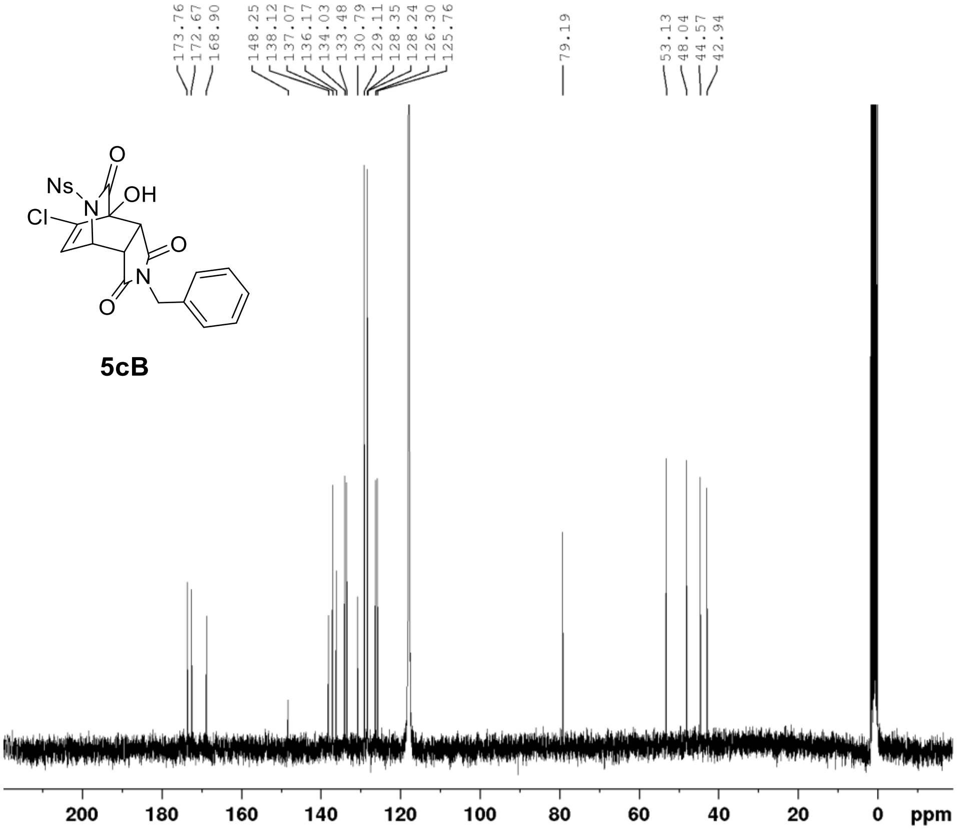

Current Data Parameters
NAME 56-HP14_C1
EXPNO 151
PROCNO 1

F2 - Acquisition Parameters
Date_ 20190626
Time 22.13
INSTRUM spect
PROBHD 5 mm PABBO BB-
PULPROG zgpg30
TD 65536
SOLVENT CD3CN
NS 1024
DS 4
SWH 18028.846 Hz
FIDRES 0.275098 Hz
AQ 1.8175317 sec
RG 2300
DW 27.733 usec
DE 8.00 usec
TE 298.5 K
D1 2.00000000 sec
D11 0.03000000 sec
TD0 1

======== CHANNEL f1 ========
NUC1 13C
P1 11.75 usec
PL1 0 dB
PL1W 31.14739227 W
SFO1 75.4752953 MHz

======== CHANNEL f2 ========
CPDPRG[2 waltz16
NUC2 1H
PCPD2 100.00 usec
PL2 -2.00 dB
PL12 17.05 dB
PL13 19.00 dB
PL2W 37.02396774 W
PL12W 0.46076876 W
PL13W 0.29409182 W
SFO2 300.1312005 MHz

F2 - Processing parameters
SI 32768
SF 75.4677086 MHz
WDW EM
SSB 0
LB 1.00 Hz
GB 0
PC 1.40

5dB

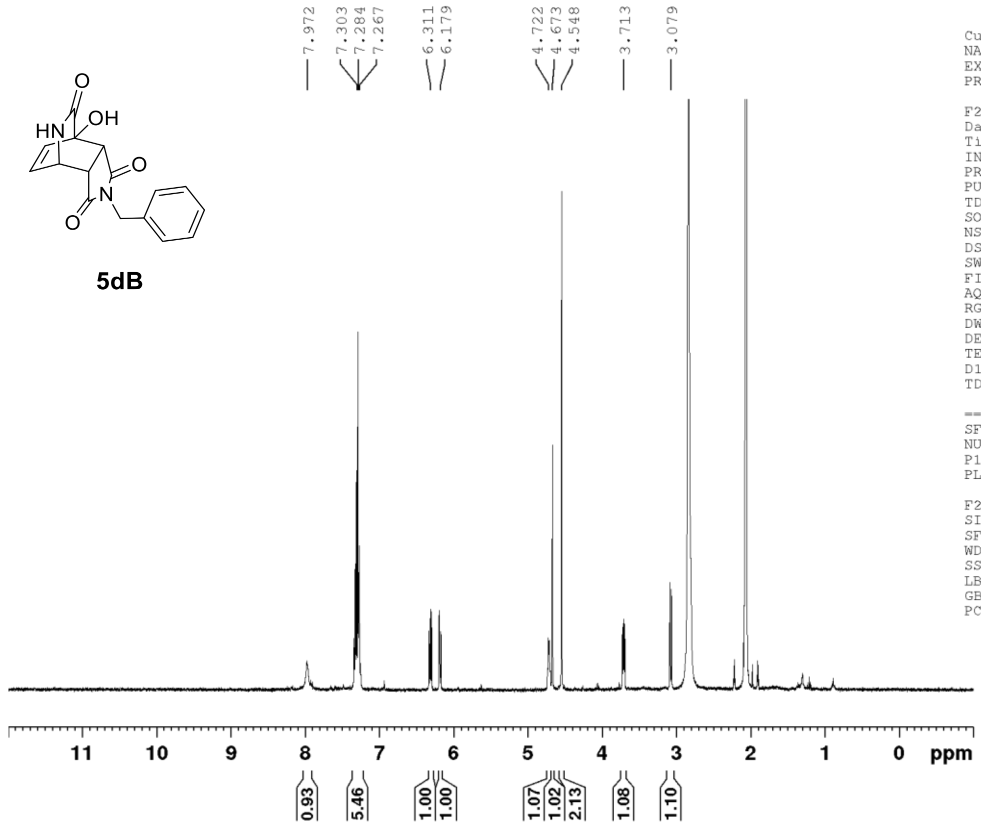


```
Current Data Parameters
NAME     56-1.4HP14-denosyl
EXPNO                 152
PROCNO                  1

F2 - Acquisition Parameters
Date_           20200402
Time               12.18
INSTRUM            spect
PROBHD   5 mm PABBO BB/
PULPROG             zg30
TD                 65536
SOLVENT          Acetone
NS                    64
DS                     2
SWH           8012.820 Hz
FIDRES        0.122266 Hz
AQ           4.0894465 sec
RG                205.35
DW              62.400 usec
DE                6.50 usec
TE               298.0 K
D1          1.00000000 sec
TD0                    1

======== CHANNEL f1 ========
SFO1       400.1024708 MHz
NUC1                  1H
P1                 13.70 usec
PLW1       12.00000000 W

F2 - Processing parameters
SI                 65536
SF          400.1000000 MHz
WDW                   EM
SSB      0
LB                 0.30 Hz
GB       0
PC                  1.00
```

5dB

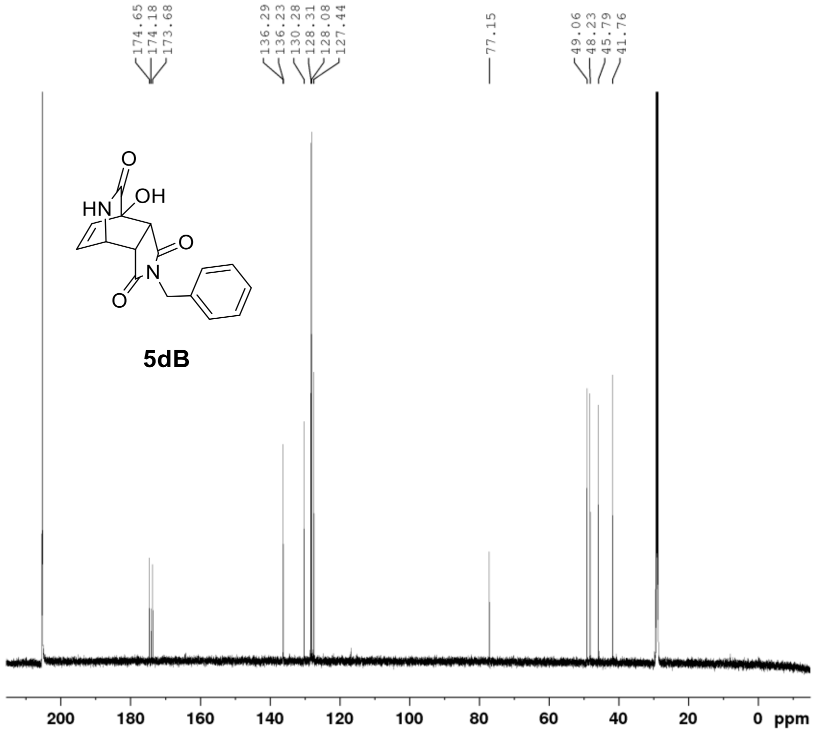


```
Current Data Parameters
NAME          56_1.6dNS
EXPNO                30
PROCNO                1

F2 - Acquisition Parameters
Date_          20200403
Time              14.19
INSTRUM           spect
PROBHD   5 mm CPQCI 1H-
PULPROG          zgpg30
TD                65356
SOLVENT         Acetone
NS                 2048
DS                    4
SWH          40760.871 Hz
FIDRES        0.623675 Hz
AQ           0.8017003 sec
RG              182.53
DW              12.267 usec
DE               18.00 usec
TE               298.0 K
D1          2.00000000 sec
D11         0.03000000 sec
TD0                  1

======== CHANNEL f1 ========
SFO1       176.1232717 MHz
NUC1                13C
P1               12.00 usec
PLW1      105.00000000 W

======== CHANNEL f2 ========
SFO2       700.3628014 MHz
NUC2                 1H
CPDPRG[2        waltz16
PCPD2            65.00 usec
PLW2        9.89999962 W
PLW12       0.15564001 W
PLW13       0.07837200 W

F2 - Processing parameters
SI               131072
SF          176.1056620 MHz
WDW                  EM
SSB      0
LB                 1.00 Hz
GB       0
PC                 1.40
```

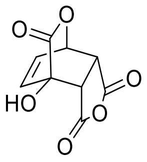

7

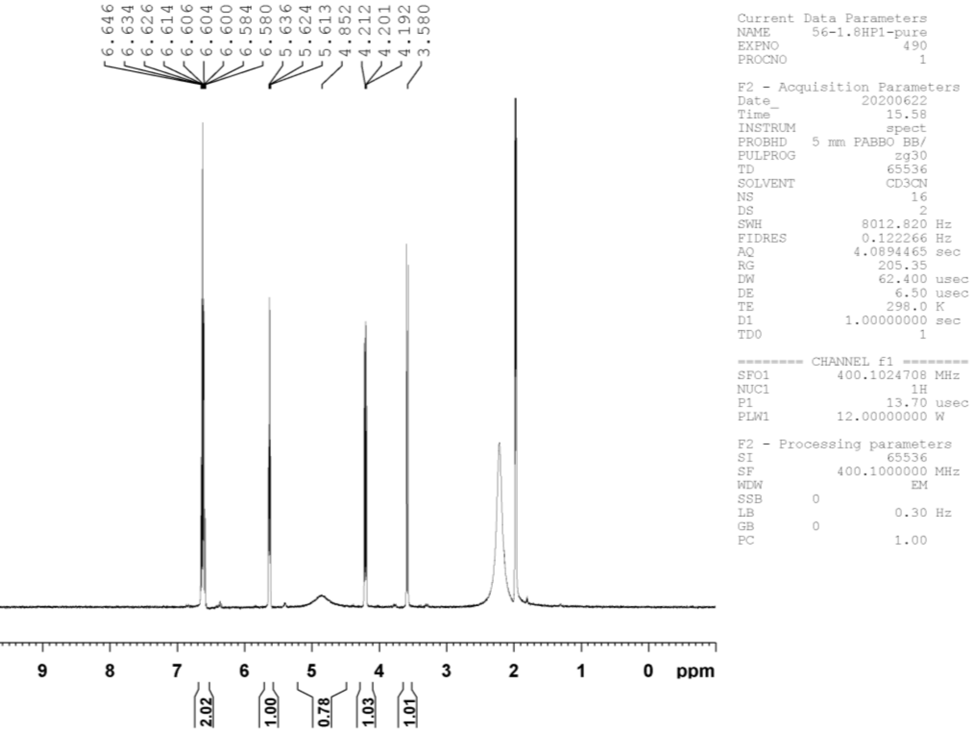

6.646
6.634
6.626
6.614
6.606
6.604
6.600
6.584
6.580
5.636
5.624
5.613
4.852
4.212
4.201
4.192
3.580
11 10 9 8 7 6 5 4 3 2 1 0 ppm
2.02
1.00
0.78
1.03
1.01
Current Data Parameters
NAME 56-1.8HP1-pure
EXPNO 490
PROCNO 1
F2 - Acquisition Parameters
Date_ 20200622
Time 15.58
INSTRUM spect
PROBHD 5 mm PABBO BB/
PULPROG zg30
TD 65536
SOLVENT CD3CN
NS 16
DS 2
SWH 8012.820 Hz
FIDRES 0.122266 Hz
AQ 4.0894465 sec
RG 205.35
DW 62.400 usec
DE 6.50 usec
TE 298.0 K
D1 1.00000000 sec
TD0 1
======== CHANNEL f1 ========
SFO1 400.1024708 MHz
NUC1 1H
P1 13.70 usec
PLW1 12.00000000 W
F2 - Processing parameters
SI 65536
SF 400.1000000 MHz
WDW EM
SSB 0
LB 0.30 Hz
GB 0
PC 1.00

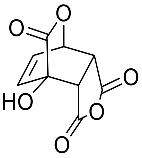

7

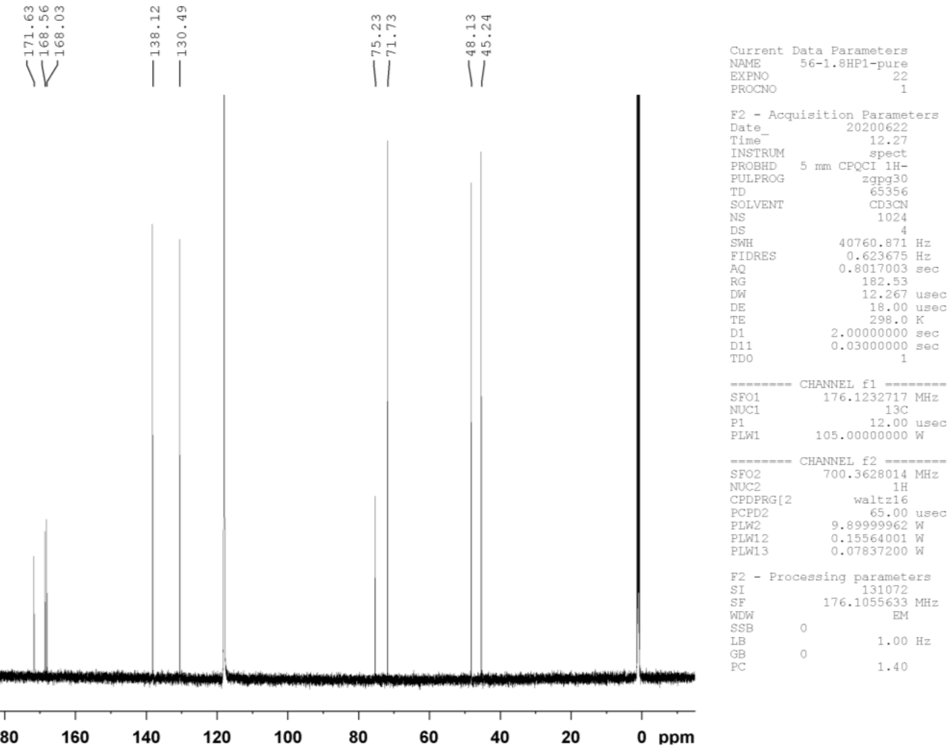

171.63
168.56
168.03
138.12
130.49
75.23
71.73
48.13
45.24
200 180 160 140 120 100 80 60 40 20 0 ppm
Current Data Parameters
NAME 56-1.8HP1-pure
EXPNO 22
PROCNO 1
F2 - Acquisition Parameters
Date_ 20200622
Time 12.27
INSTRUM spect
PROBHD 5 mm CPQCI 1H-
PULPROG zgpg30
TD 65356
SOLVENT CD3CN
NS 1024
DS 4
SWH 40760.871 Hz
FIDRES 0.623675 Hz
AQ 0.8017003 sec
RG 182.53
DW 12.267 usec
DE 18.00 usec
TE 298.0 K
D1 2.00000000 sec
D11 0.03000000 sec
TD0 1
======== CHANNEL f1 ========
SFO1 176.1232717 MHz
NUC1 13C
P1 12.00 usec
PLW1 105.00000000 W
======== CHANNEL f2 ========
SFO2 700.3628014 MHz
NUC2 1H
CPDPRG[2 waltz16
PCPD2 65.00 usec
PLW2 9.89999962 W
PLW12 0.15564001 W
PLW13 0.07837200 W
F2 - Processing parameters
SI 131072
SF 176.1055633 MHz
WDW EM
SSB 0
LB 1.00 Hz
GB 0
PC 1.40

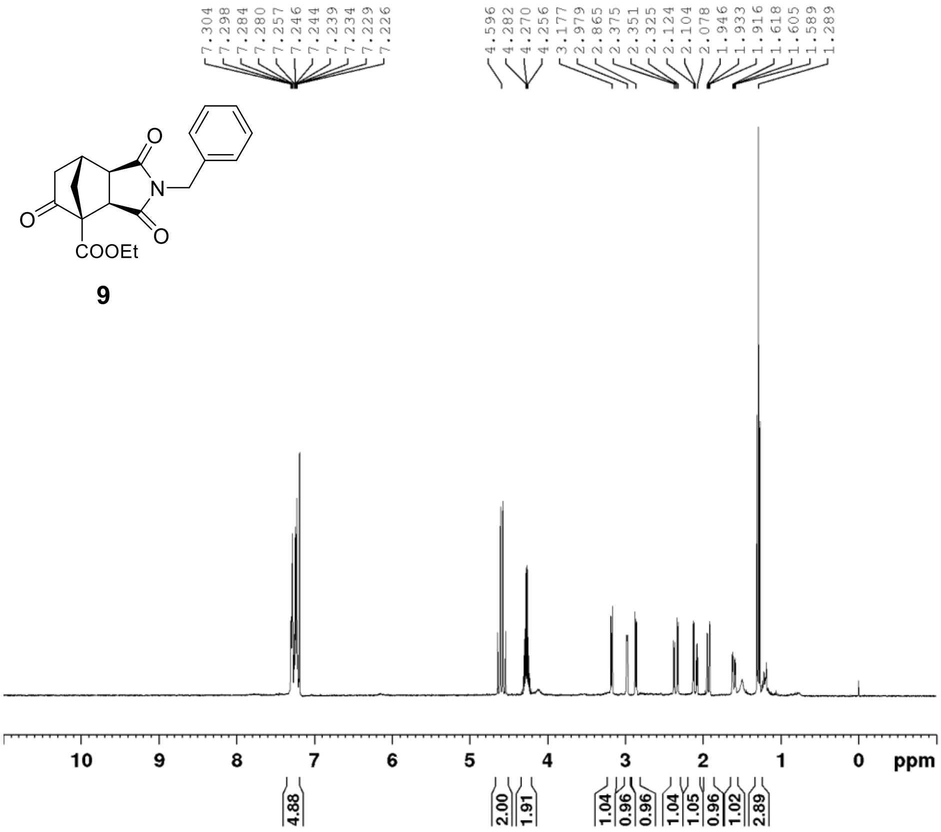


```
Current Data Parameters
NAME            56-1.8S6
EXPNO                470
PROCNO                 1

F2 - Acquisition Parameters
Date_           20200312
Time               17.06
INSTRUM            spect
PROBHD   5 mm PABBO BB/
PULPROG             zg30
TD                 65536
SOLVENT            CDCl3
NS                    16
DS                     2
SWH          8012.820 Hz
FIDRES       0.122266 Hz
AQ          4.0894465 sec
RG                205.35
DW            62.400 usec
DE              6.50 usec
TE               298.0 K
D1        1.00000000 sec
TD0                    1

======== CHANNEL f1 ========
SFO1      400.1024708 MHz
NUC1                  1H
P1             13.70 usec
PLW1      12.00000000 W

F2 - Processing parameters
SI                 65536
SF        400.1000365 MHz
WDW                   EM
SSB      0
LB               0.30 Hz
GB       0
PC                  1.00
```

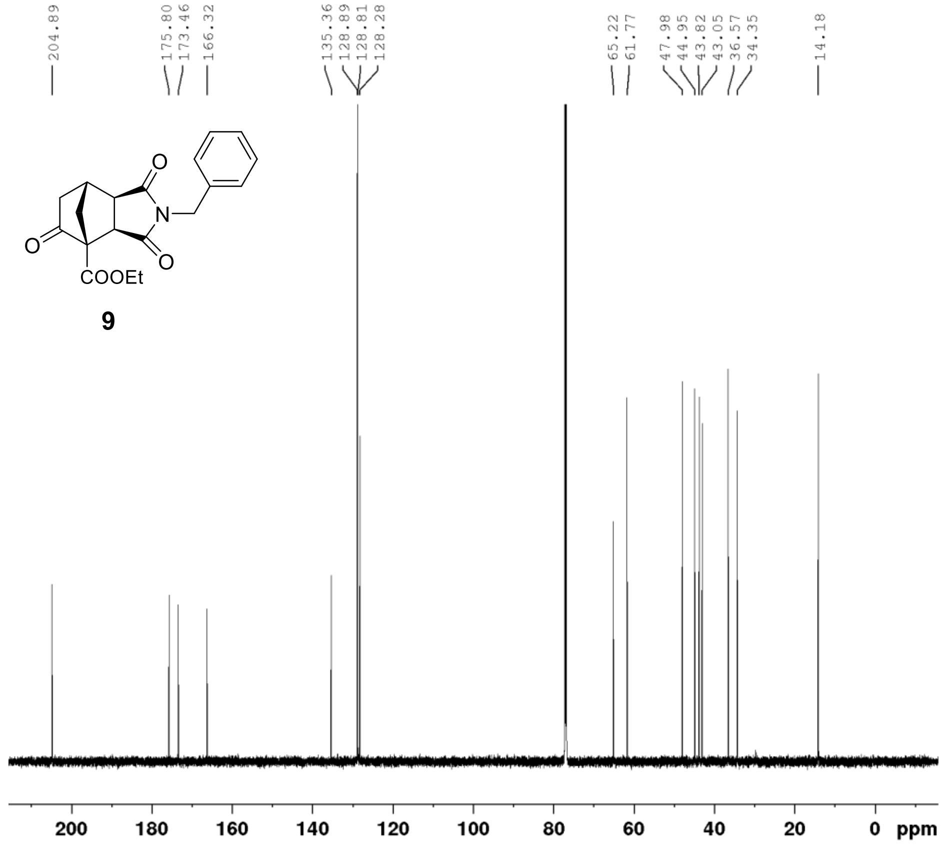


```
Current Data Parameters
NAME         56-1.8S6-_20
EXPNO                 31
PROCNO                 1

F2 - Acquisition Parameters
Date_           20200313
Time               13.13
INSTRUM            spect
PROBHD   5 mm CPQCI 1H-
PULPROG           zgpg30
TD                 65356
SOLVENT            CDCl3
NS                  2048
DS                     4
SWH         40760.871 Hz
FIDRES       0.623675 Hz
AQ          0.8017003 sec
RG                182.53
DW            12.267 usec
DE             18.00 usec
TE               298.0 K
D1        2.00000000 sec
D11       0.03000000 sec
TD0                    1

======== CHANNEL f1 ========
SFO1      176.1232717 MHz
NUC1                  13C
P1             12.00 usec
PLW1     105.00000000 W

======== CHANNEL f2 ========
SFO2      700.3628014 MHz
NUC2                  1H
CPDPRG[2         waltz16
PCPD2          65.00 usec
PLW2        9.89999962 W
PLW12       0.15564001 W
PLW13       0.07837200 W

F2 - Processing parameters
SI                131072
SF        176.1056620 MHz
WDW                   EM
SSB      0
LB               1.00 Hz
GB       0
PC                  1.40
```

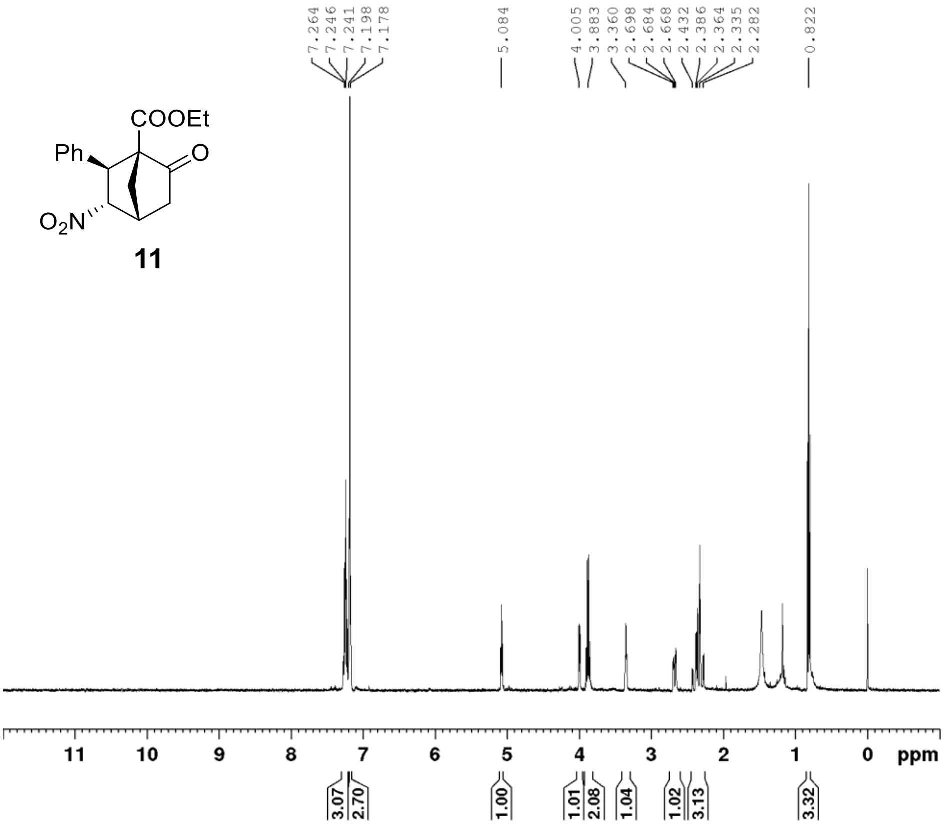


```
Current Data Parameters
NAME             56-1.3S6
EXPNO                 490
PROCNO                  1

F2 - Acquisition Parameters
Date_            20200312
Time                18.25
INSTRUM             spect
PROBHD   5 mm PABBO BB/
PULPROG              zg30
TD                  65536
SOLVENT             CDCl3
NS                     32
DS                      2
SWH              8012.820 Hz
FIDRES           0.122266 Hz
AQ              4.0894465 sec
RG                 205.35
DW                 62.400 usec
DE                   6.50 usec
TE                  298.0 K
D1             1.00000000 sec
TD0                     1

======== CHANNEL f1 ========
SFO1        400.1024708 MHz
NUC1                   1H
P1                  13.70 usec
PLW1          12.00000000 W

F2 - Processing parameters
SI                  65536
SF          400.1000368 MHz
WDW                    EM
SSB      0
LB                   0.30 Hz
GB       0
PC                   1.00
```

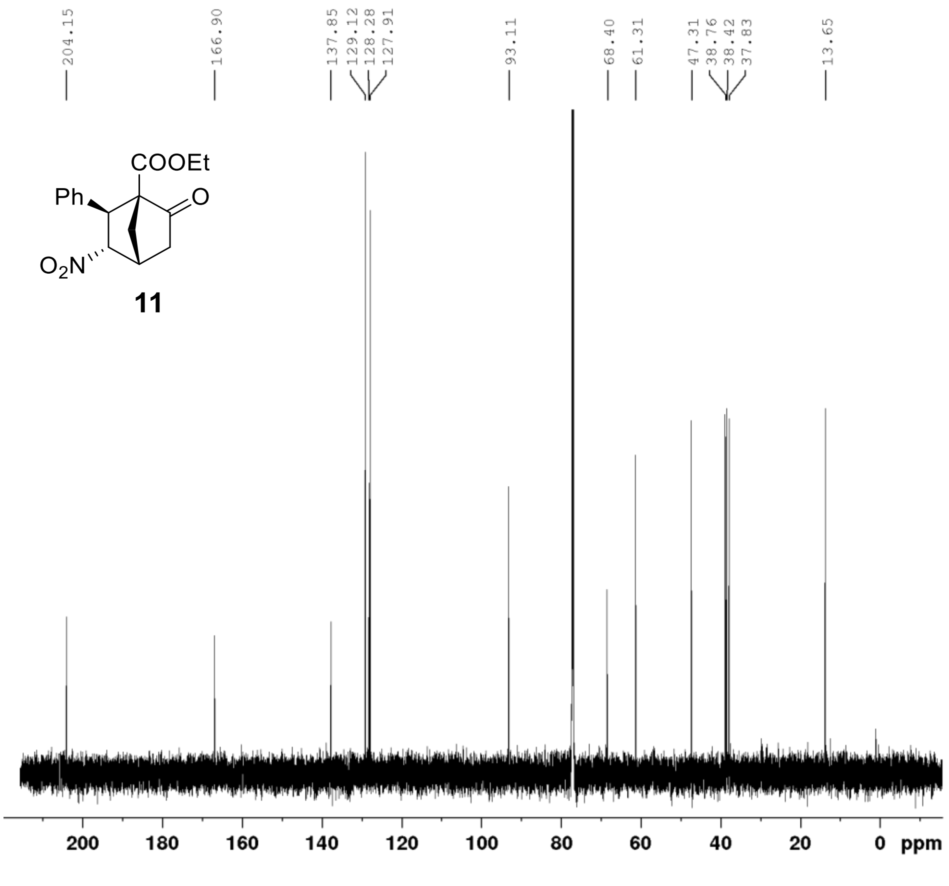


```
Current Data Parameters
NAME       56-NO2-pure_rac
EXPNO                  41
PROCNO                  1

F2 - Acquisition Parameters
Date_            20200313
Time                14.32
INSTRUM             spect
PROBHD   5 mm CPQCI 1H-
PULPROG            zgpg30
TD                  65356
SOLVENT             CDCl3
NS                   1024
DS                      4
SWH             40760.871 Hz
FIDRES           0.623675 Hz
AQ              0.8017003 sec
RG                 182.53
DW                 12.267 usec
DE                  18.00 usec
TE                  298.0 K
D1             2.00000000 sec
D11            0.03000000 sec
TD0                     1

======== CHANNEL f1 ========
SFO1        176.1232717 MHz
NUC1                  13C
P1                  12.00 usec
PLW1         105.00000000 W

======== CHANNEL f2 ========
SFO2        700.3628014 MHz
NUC2                   1H
CPDPRG[2          waltz16
PCPD2               65.00 usec
PLW2           9.89999962 W
PLW12          0.15564001 W
PLW13          0.07837200 W

F2 - Processing parameters
SI                 131072
SF          176.1056620 MHz
WDW                    EM
SSB      0
LB                   1.00 Hz
GB       0
PC                   1.40
```

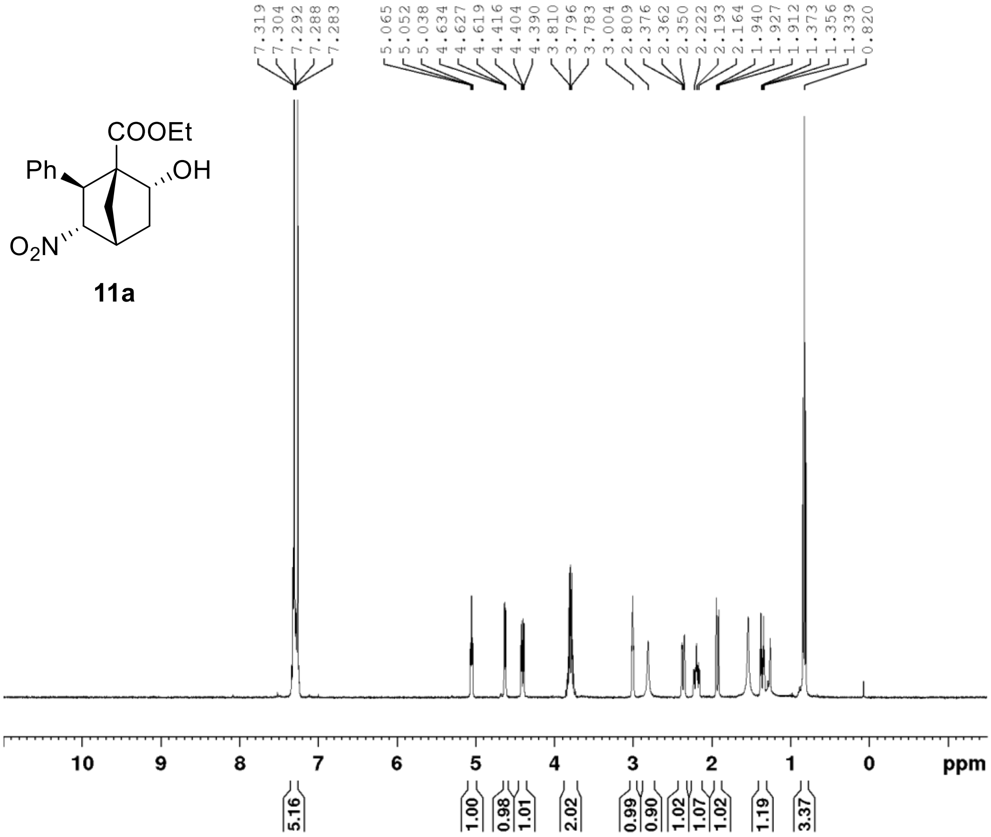


```
Current Data Parameters
NAME       56-1.3S6redB
EXPNO               180
PROCNO                1

F2 - Acquisition Parameters
Date_          20200421
Time              11.55
INSTRUM           spect
PROBHD   5 mm PABBO BB/
PULPROG            zg30
TD                65536
SOLVENT           CDC13
NS                   16
DS                    2
SWH          8012.820 Hz
FIDRES       0.122266 Hz
AQ          4.0894465 sec
RG               205.35
DW             62.400 usec
DE               6.50 usec
TE              298.0 K
D1         1.00000000 sec
TD0                   1

======== CHANNEL f1 ========
SFO1       400.1024708 MHz
NUC1                 1H
P1               13.70 usec
PLW1       12.00000000 W

F2 - Processing parameters
SI                65536
SF         400.1000097 MHz
WDW                  EM
SSB      0
LB                 0.30 Hz
GB       0
PC                 1.00
```

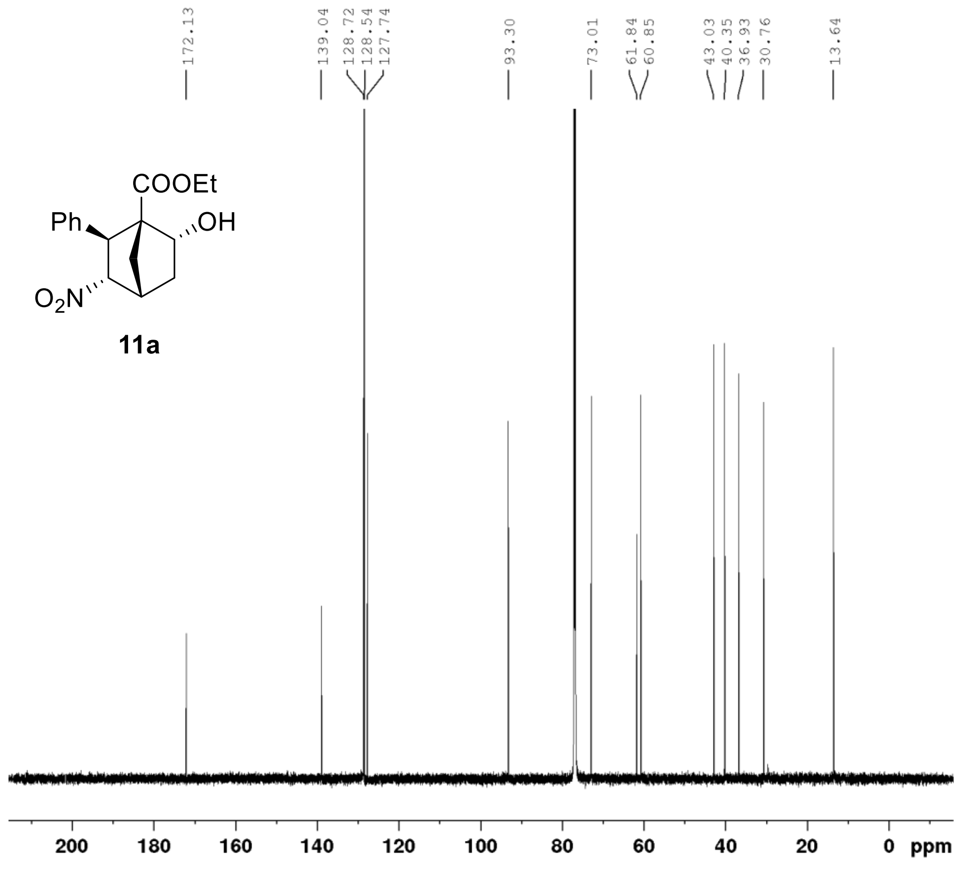


```
Current Data Parameters
NAME        56-1.4b-red
EXPNO                41
PROCNO                1

F2 - Acquisition Parameters
Date_          20200421
Time              15.22
INSTRUM           spect
PROBHD   5 mm CPQCI 1H-
PULPROG          zgpg30
TD                65356
SOLVENT           CDC13
NS                 2048
DS                    4
SWH         40760.871 Hz
FIDRES       0.623675 Hz
AQ          0.8017003 sec
RG               182.53
DW             12.267 usec
DE              18.00 usec
TE              298.0 K
D1         2.00000000 sec
D11        0.03000000 sec
TD0                   1

======== CHANNEL f1 ========
SFO1       176.1232717 MHz
NUC1                13C
P1               12.00 usec
PLW1      105.00000000 W

======== CHANNEL f2 ========
SFO2       700.3628014 MHz
NUC2                 1H
CPDPRG[2        waltz16
PCPD2            65.00 usec
PLW2         9.89999962 W
PLW12        0.15564001 W
PLW13        0.07837200 W

F2 - Processing parameters
SI               131072
SF         176.1056620 MHz
WDW                  EM
SSB      0
LB                 1.00 Hz
GB       0
PC                 1.40
```

Determination of Enantiomeric Excess

3aA

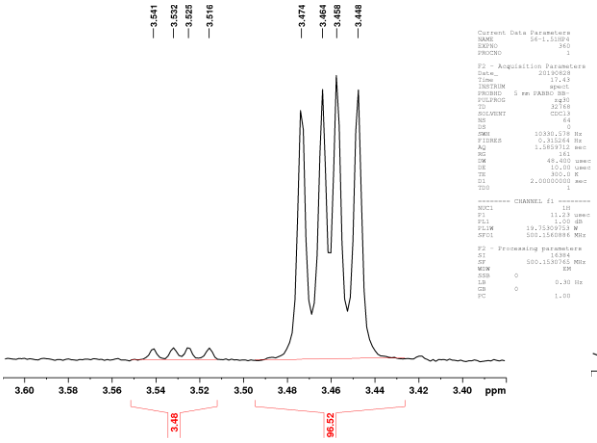

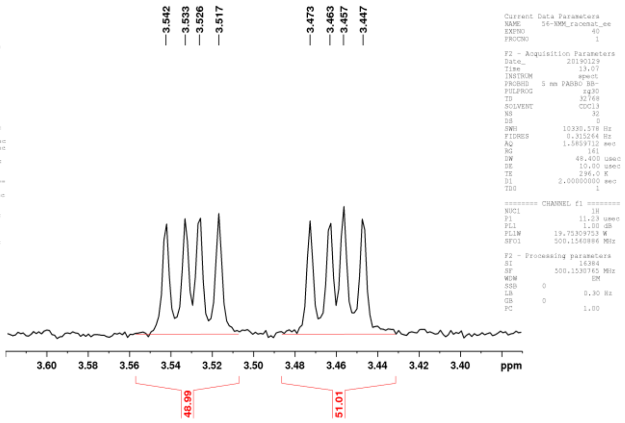

Racemic

3aB

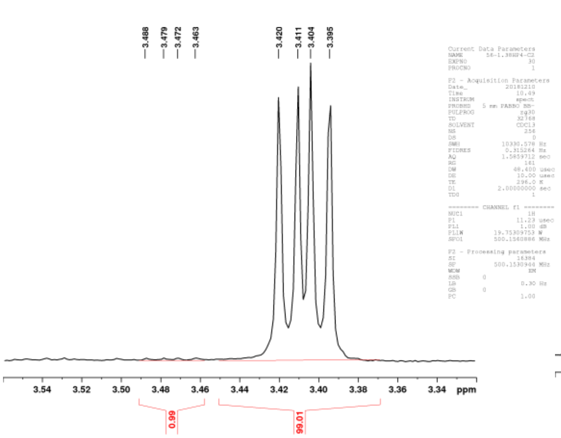

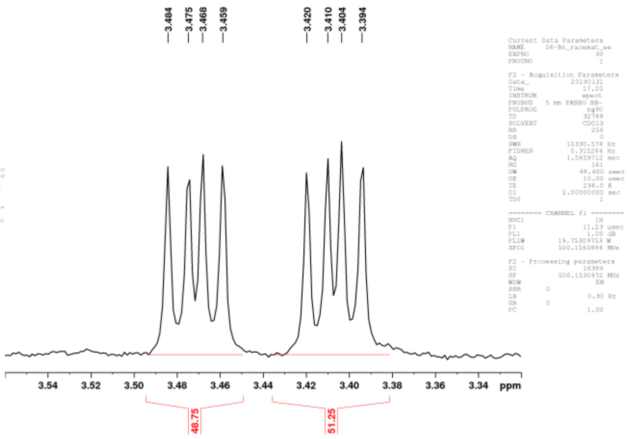

Racemic

3aC

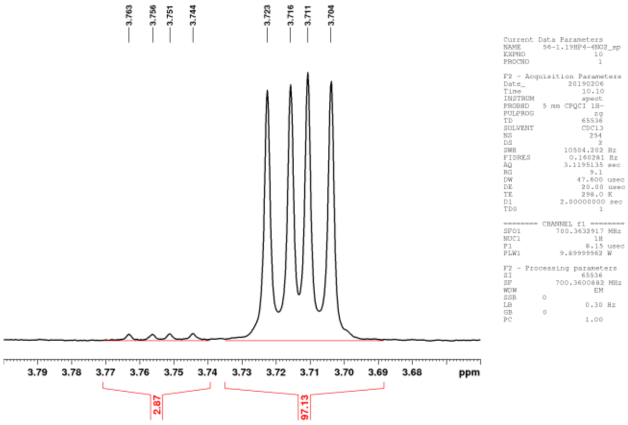

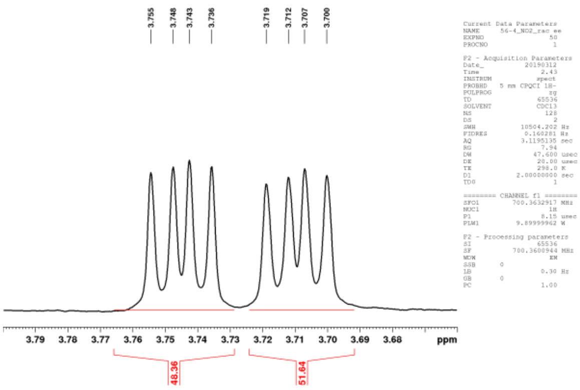

Racemic

3aD

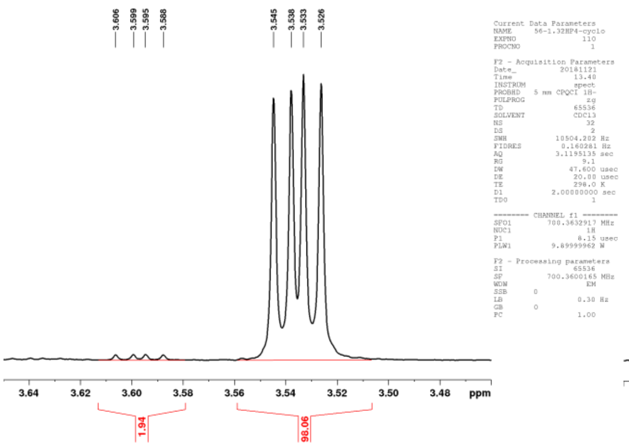

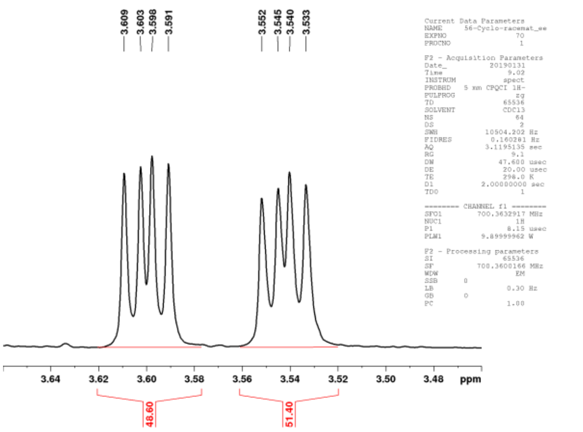

Racemic

3aE

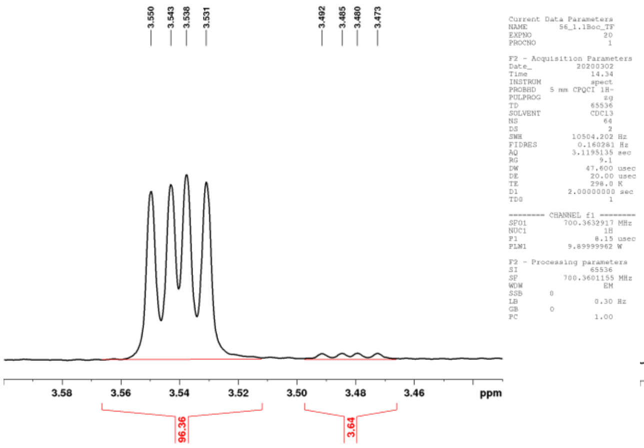

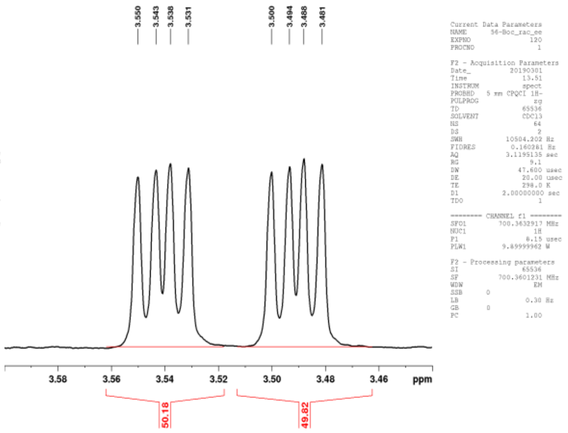

Racemic

3aF

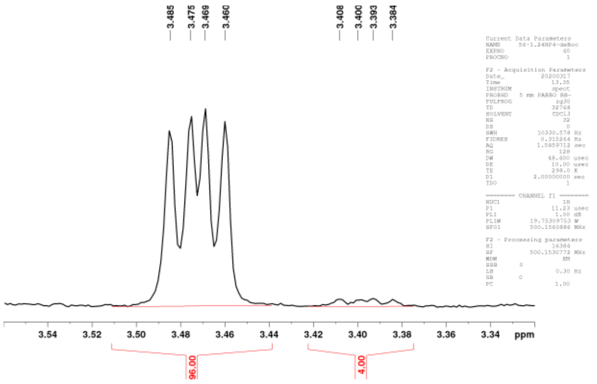

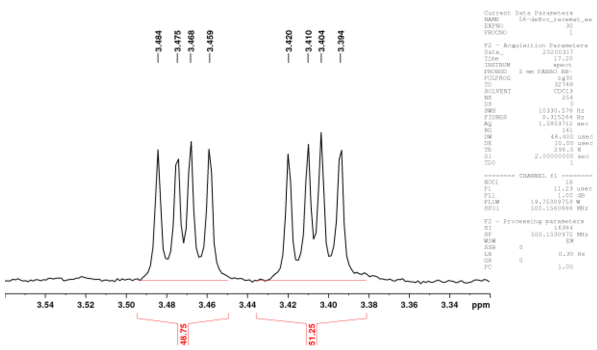

Racemic

3aG

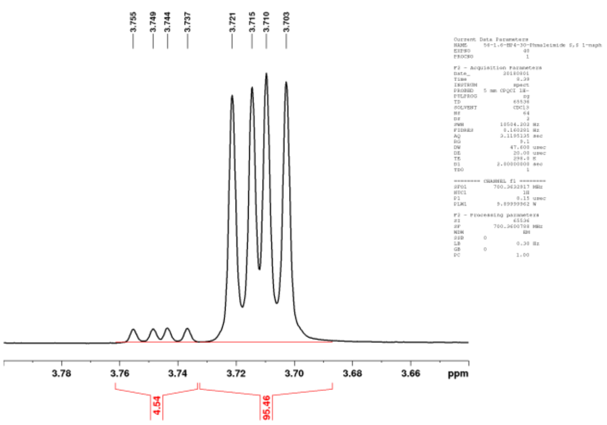

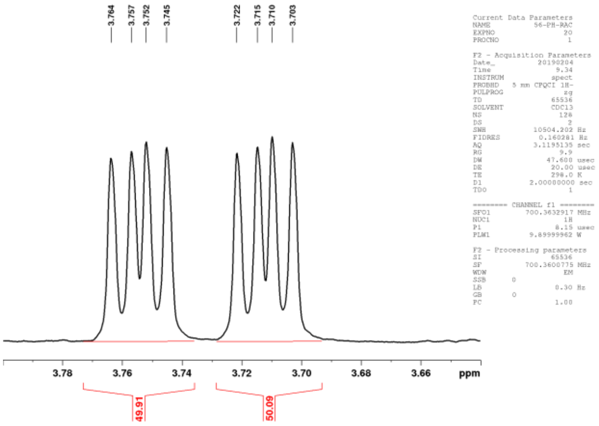

Racemic

3aH

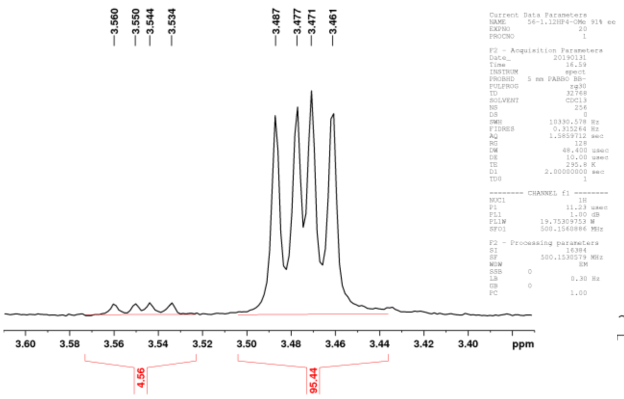

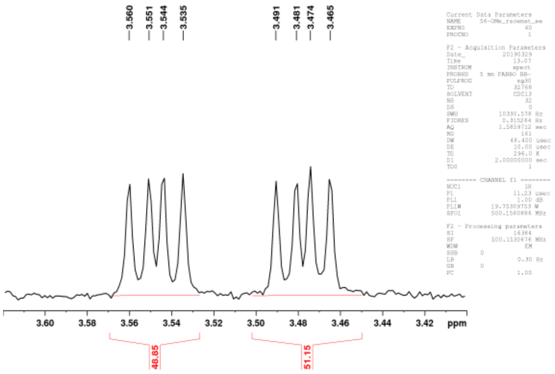

Racemic

3aI

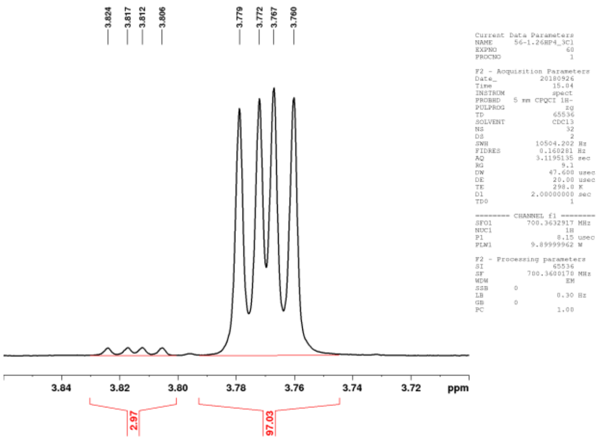

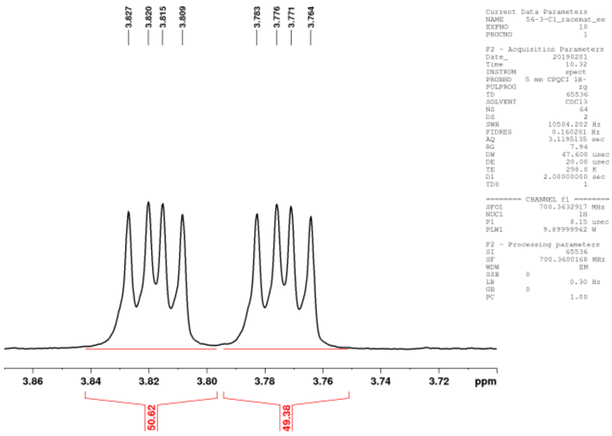

Racemic

3aJ

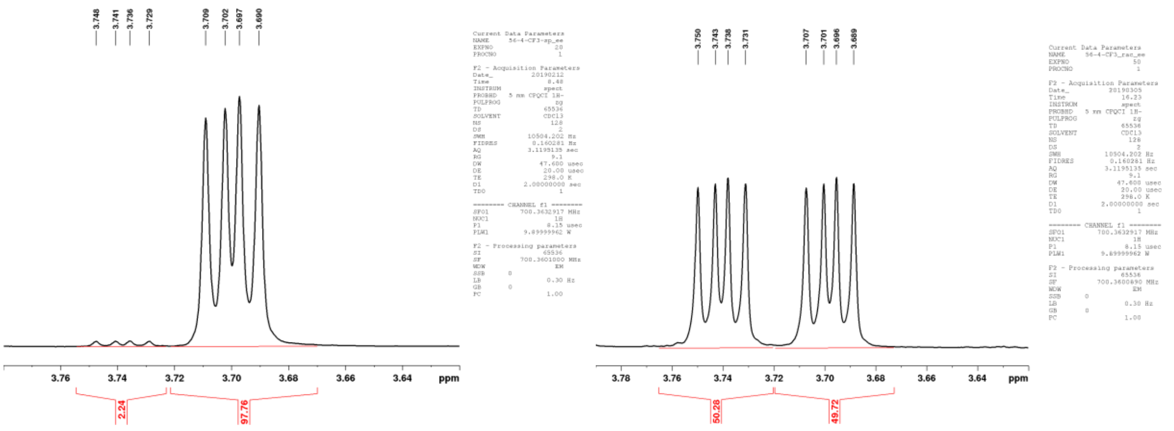

Racemic

3aK

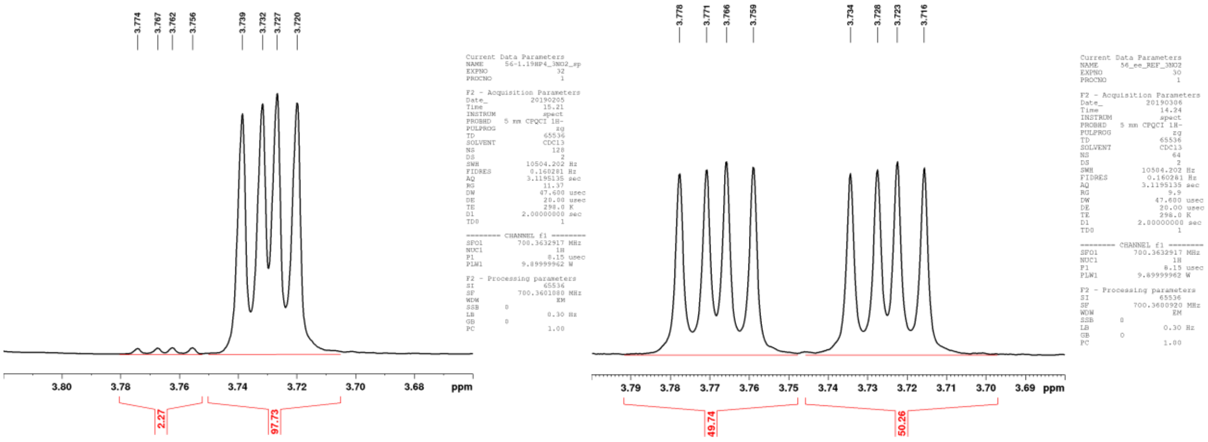

Racemic

3aL

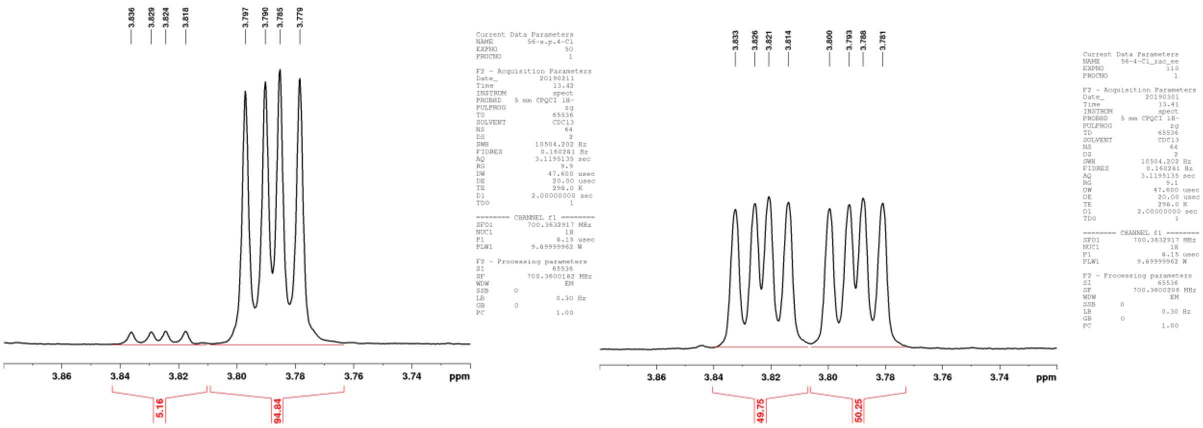

Racemic

3aM

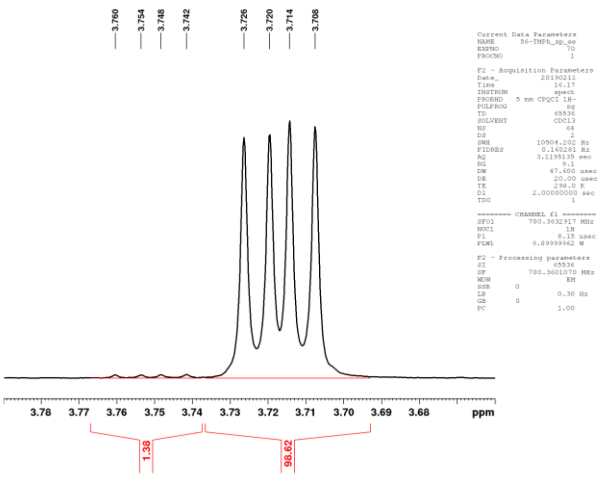

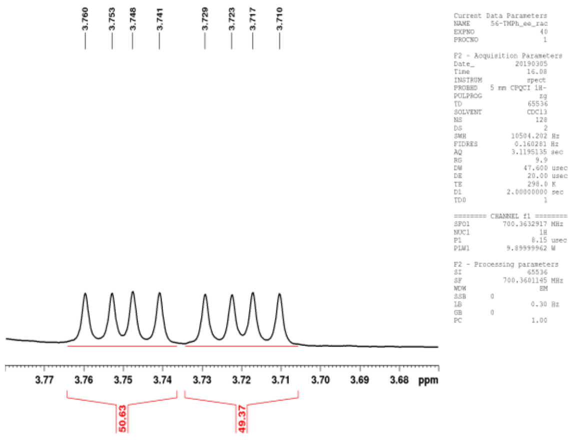

Racemic

3bB

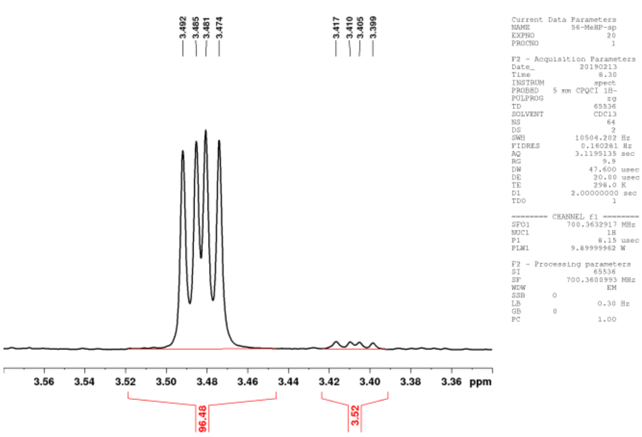

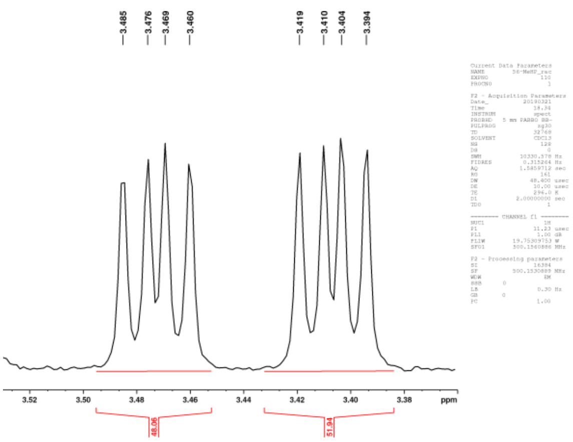

Racemic

3cB

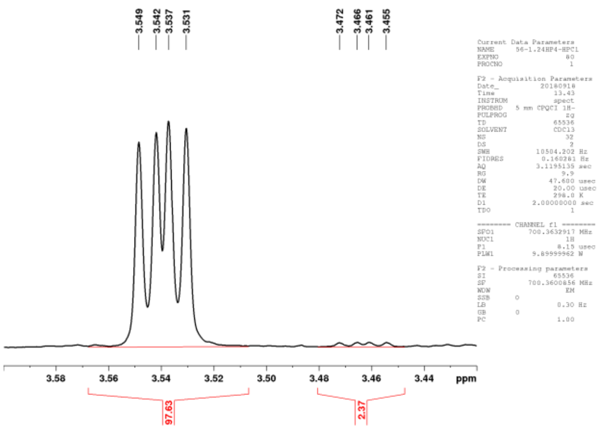

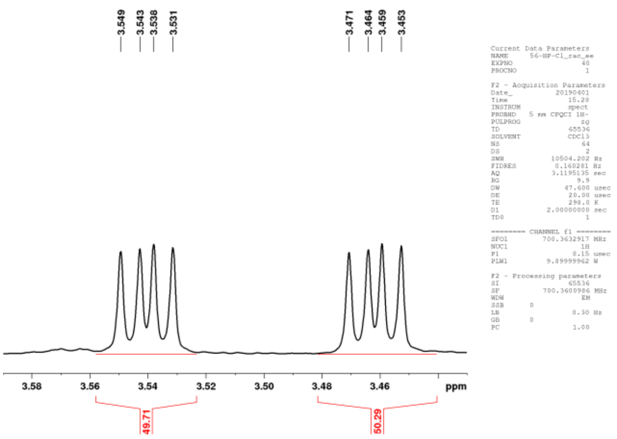

Racemic

3dB

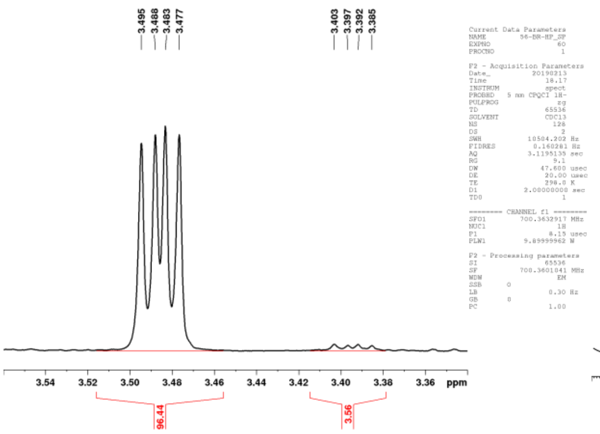

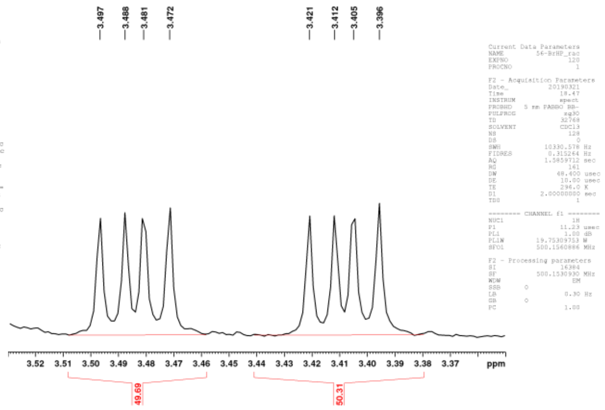

Racemic

5aB

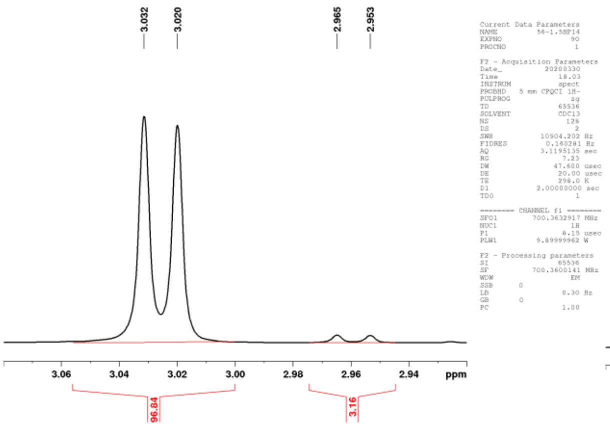

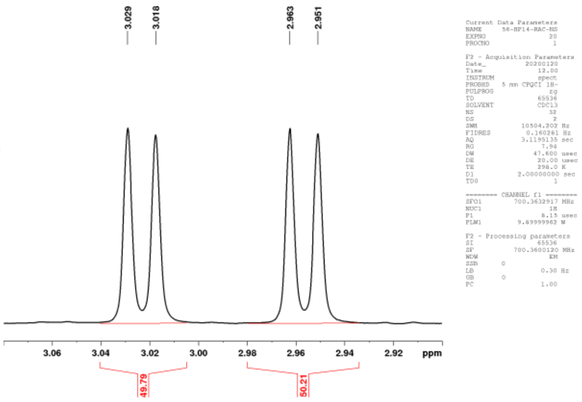

Racemic

5aC

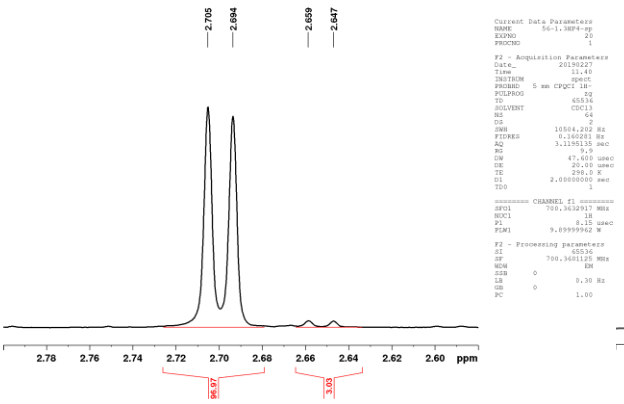

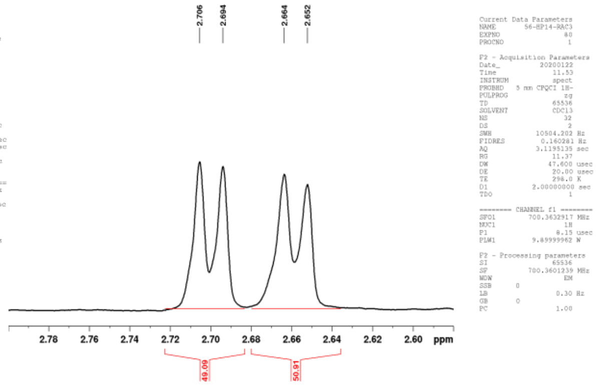

Racemic

5aF

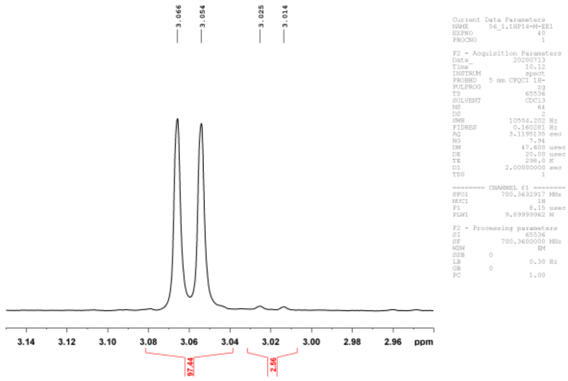

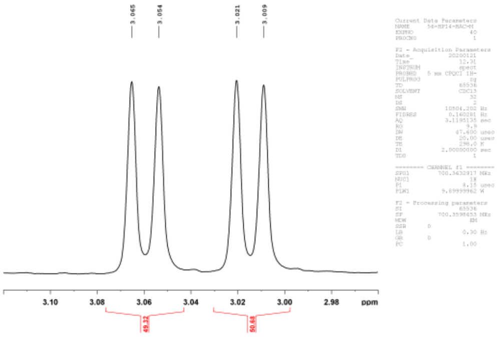

Racemic

5bB

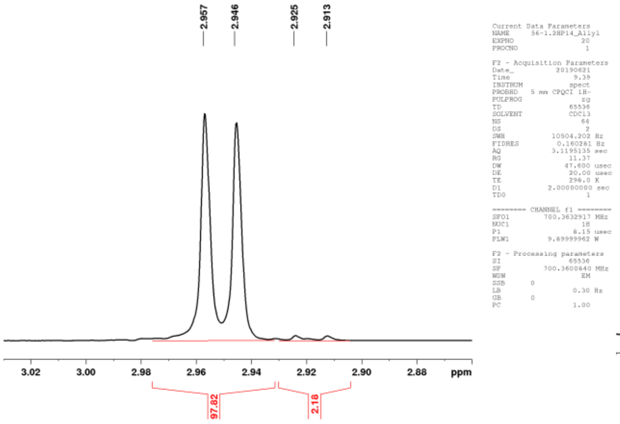

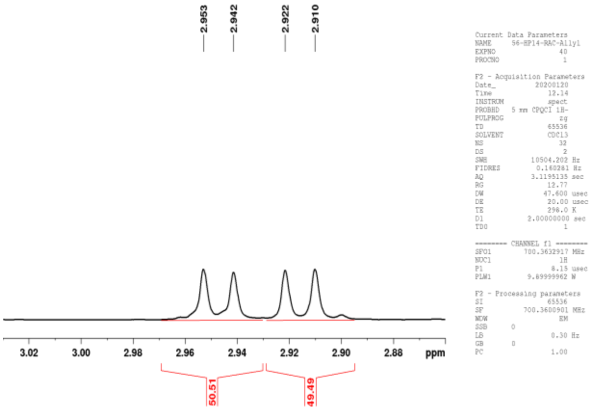

Racemic

5cB

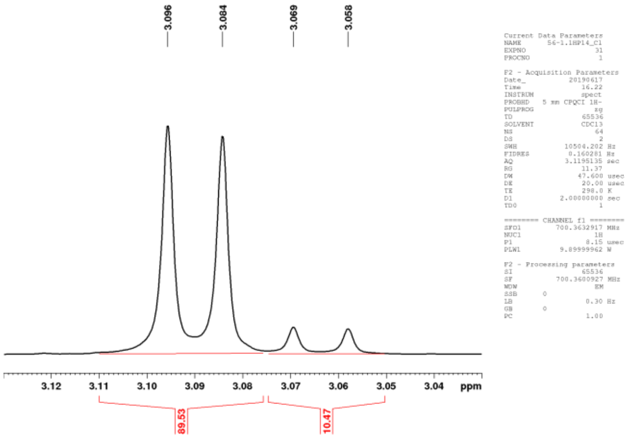

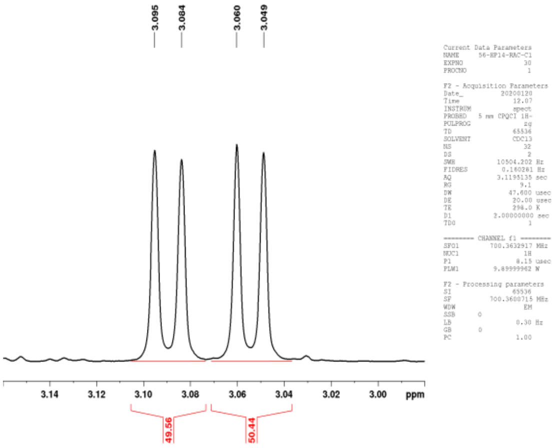

Racemic

5dB

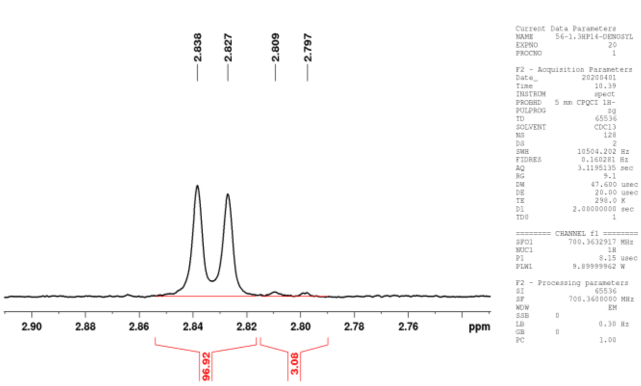

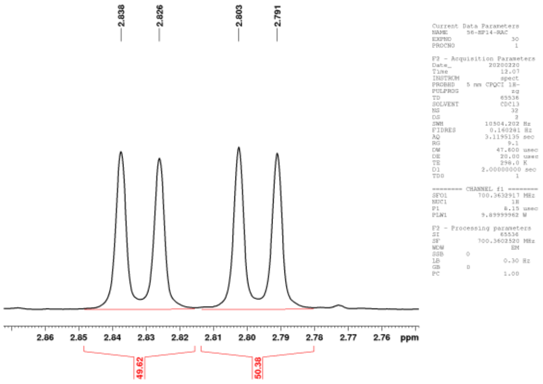

Racemic

7

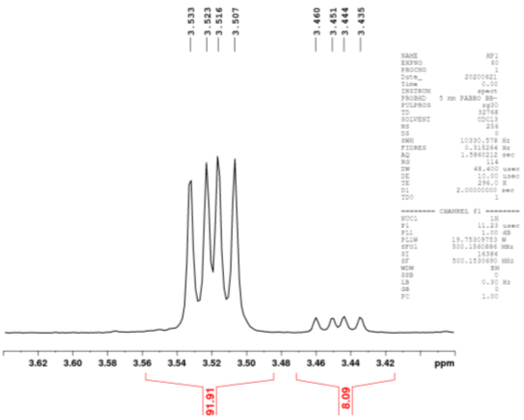

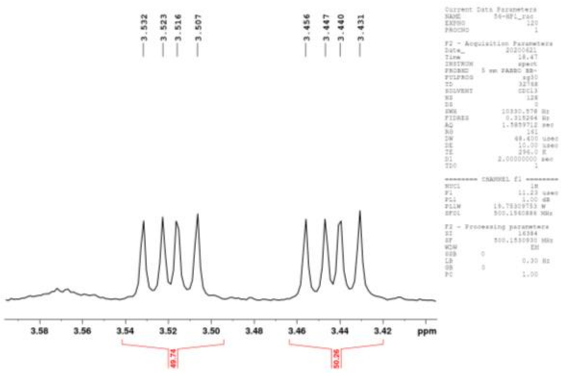

Racemic

9

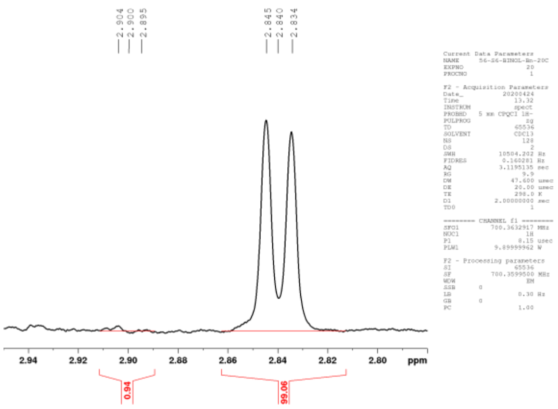

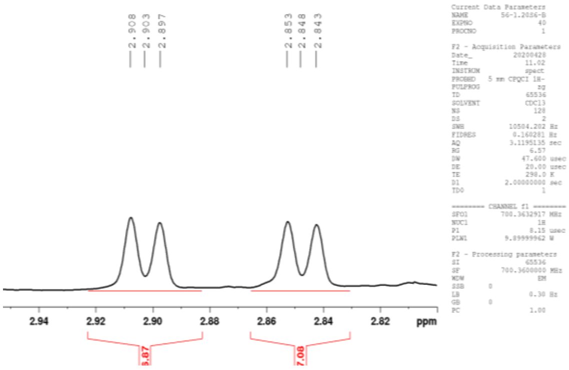

Racemic

HPLC Data

11

Column : ODH, *n*-heptane/iPrOH (90/10), 0.6 mL/min, 220 nm

Racemic:

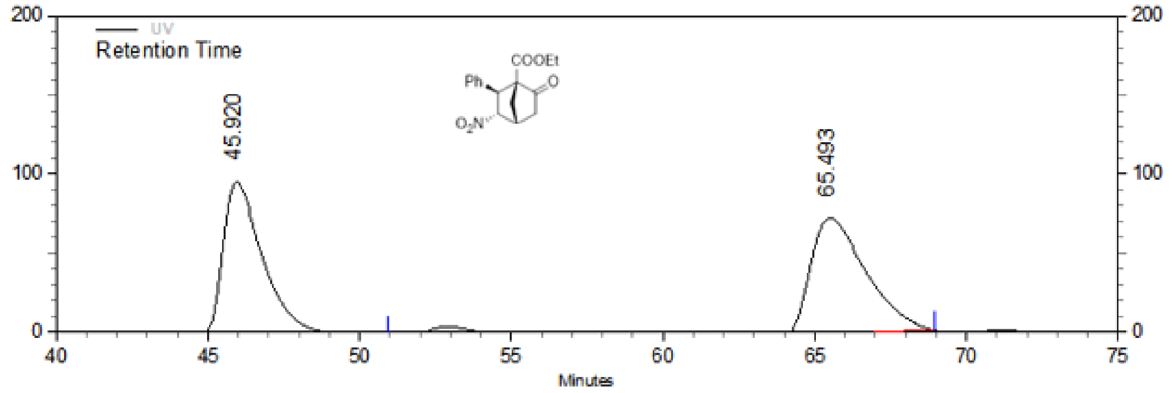

Retention time	Area	Area (%)
45.92	35288703	48.84
65.49	36969922	51.16

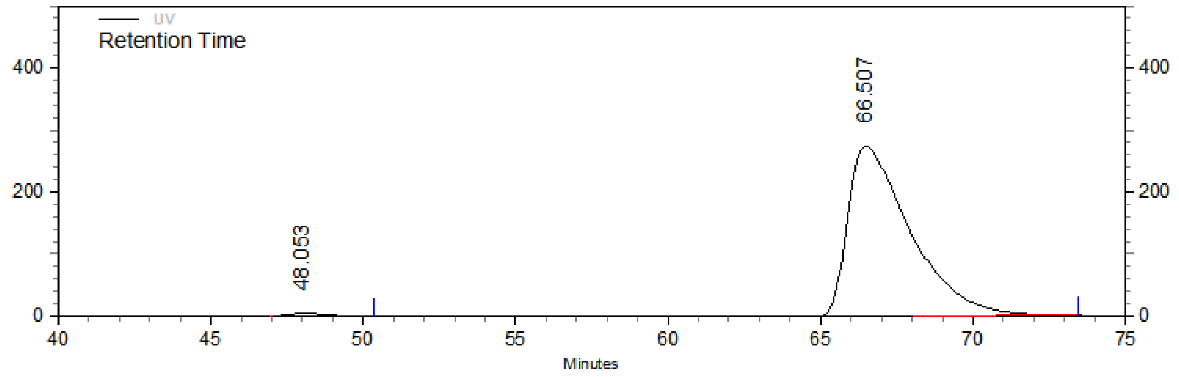

Retention time	Area	Area (%)
48.05	1582827	1.00
66.51	156967898	99.00

References

[1] (a) M. Sortino, V. Cechinel Filho, R. Corrêa, S. Zacchino *Bioorg. Med. Chem.* **2008**, *16,* 560–568; (b) Y. Lu, Y. Li, R. Zhang, K. Jin, C. Duan *Tetrahedron* **2013**, *69*, 9422-9427. (c) K. P. Haval, S. B. Mhaske, N. P. Argade, *Tetrahedron* **2006**, *62*, 937–942.

[2] J. A. Profitt, T. Jones, D. S. Watt *Synth. Commun.* **1975**, *5*, 457–460.

[3] T. Suzuki, S. Watanabe, S. Kobayashi, K. Tanino *Org. Lett*. **2017**, *19*, 922–925.

[4] T. Ishiyama, D. Urabe, H. Fujisawa, M. Inoue *Org. Lett.* **2013**, *15*, 4488–4491.

[5] N. T. Kipassa, H. Okamura, K. Kina, T. Hamada, T. Iwagawa *Org. Lett.* **2008**, *10*, 815–816.

[6] F. Willig, J. Lang, A. C. Hans, M. Ringenberg, D. Pfeffer, W. Frey, R. Peters *J. Am. Chem. Soc.* **2019**, *141*, 30, 12029–12043.

[7] H. Okamura, Y. Nakamura, T. Iwagawa, M. Nakatani *Chem. Lett.* **1996**, *25*, 193–194.

[8] (a) T. Komiyama, Y. Takaguchi, S. Tsuboi *Tetrahedron Lett*. **2004**, *45*, 6299–6301; (b) T. Komiyama, Y. Takaguchi, A. T. Gubaidullin, V. A. Mamedov, I. A. Litvinov, S. Tsuboi *Tetrahedron* **2005**, *61*, 2541–2547.

[9] J. Y.-T. Soh, C.-H. Tan *J. Am. Chem. Soc.* **2009**, *131*, 6904–6905.

[10] A. R. Chianese, R. H. Crabtree *Organometallics* **2005,** *24,* 4432–4436.

[11] S. Reymond, J. Cossy, *Chem. Rev.* **2008**, *108*, 5359–5406.

[12] H. Okamura, T. Iwagawa, M. Nakatani *Tetrahedron Lett.* **1995**, *36*, 5939–5942.

[13] H. Okamura, H. Nagaike, T. Iwagawa, M. Nakatani *Tetrahedron Lett.* **2000**, *41*, 8317–8321.

[14] (a) D. G. Blackmond *J. Am. Chem. Soc.* **2015**, *137*, 10852–10866; (b) R. D. Baxter, D. Sale, K. M. Engle, J.-Q. Yu, D. G. Blackmond *J. Am. Chem. Soc*. **2012**, *134*, *10*, 4600–4606.

[15] J. Burés *Angew. Chem. Int. Ed.* **2016**, *55*, 16084–16087.

[16] C. D.-T. Nielsen, J. Burés *Chem. Sci*. **2019**, *10*, 348–353.
